# Boron tribromide as a reagent for anti-Markovnikov addition of HBr to cyclopropanes†

**DOI:** 10.1039/d0sc02567d

**Published:** 2020-08-04

**Authors:** Matthew H. Gieuw, Shuming Chen, Zhihai Ke, K. N. Houk, Ying-Yeung Yeung

**Affiliations:** Department of Chemistry, State Key Laboratory of Synthetic Chemistry, The Chinese University of Hong Kong Shatin NT Hong Kong China yyyeung@cuhk.edu.hk; Department of Chemistry and Biochemistry, University of California Los Angeles California 90095 USA houk@chem.ucla.edu

## Abstract

Although radical formation from a trialkylborane is well documented, the analogous reaction mode is unknown for trihaloboranes. We have discovered the generation of bromine radicals from boron tribromide and simple proton sources, such as water or *tert*-butanol, under open-flask conditions. Cyclopropanes bearing a variety of substituents were hydro- and deuterio-brominated to furnish anti-Markovnikov products in a highly regioselective fashion. NMR mechanistic studies and DFT calculations point to a radical pathway instead of the conventional ionic mechanism expected for BBr_3_.

## Abstract 2

Anti-Markovnikov hydrobromination of cyclopropanes was achieved using boron tribromide and water as the bromine and proton sources, respectively.

## Graphical abstract

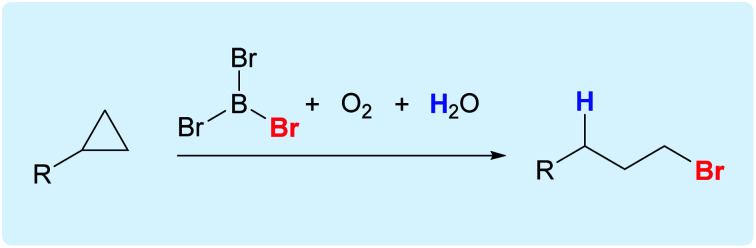

The Lewis acidic nature of organoboranes is well understood, but the participation of BR_3_ in free-radical processes was largely overlooked until 1966.^1^ Since the discovery of the potential of organoborane species to undergo radical reactions, many novel and synthetically useful transformations were developed.^2^ Trialkylboranes (BR_3_) can easily undergo bimolecular homolytic substitution (S_H_2) at the boron atom to generate alkyl radicals (Scheme 1A). It was found that alkoxyl, dialkylaminyl, alkylthiyl and carbon-centered radicals, triplet ketones, and triplet oxygen can all initiate the radical reaction by substituting one of the alkyl groups of trialkylboranes to liberate alkyl radicals.^3^ BEt_3_/O_2_ is arguably the most studied organoborane radical-initiating system, with the peroxyl radical being the key to propagate the reaction. Apart from being a radical initiator, BEt_3_, along with trace amount of O_2_, can also undergo conjugate addition to unsaturated ketones and aldehydes; addition to ethenyl- and ethynyloxiranes, azidoalkenes, and imines; and addition–elimination to nitroalkenes and nitroarenes, styryl sulfones, sulfoxides and sulfinimides.^3^ However, apart from changing BEt_3_ to other trialkylboranes or catecholborane to carry out similar radical reactions, the radical-reaction potential of other organoboranes remains underexplored, given the ease and mild conditions under which they initiate radical chains, often with trace amount of O_2_ in air at low temperature. The application of such a mild radical-initiation system to stereoselective radical reactions would drastically change the reaction outcome especially when intermediates and products are thermally unstable.^4^

**Scheme 1:**
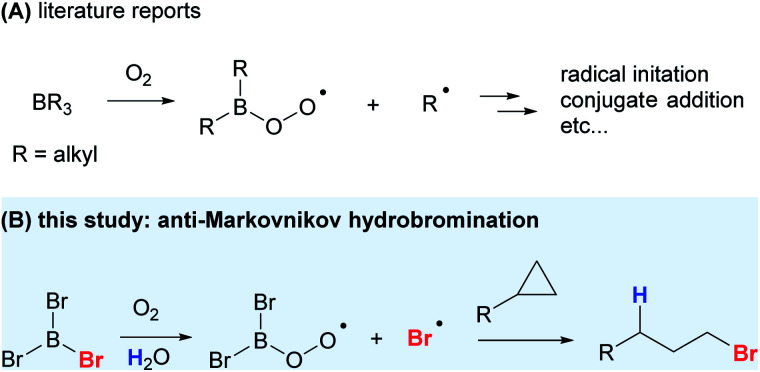
Classical radical reactions with trialkylboranes and our work on radical bromination using BBr_3_.

Halogenation is an important class of transformations and the resultant halogenated products can easily be manipulated to give a wide range of functional molecules.^5^ While trihaloboranes have been employed as halogenating or haloborating agents, their role in reactions is either ambiguous or thought to be exclusively Lewis acidic.^6^ To date, the use of trihaloboranes as a halogen radical donor has not been reported. With BR_3_/O_2_ being a versatile radical-initiator and conjugate-addition system, we envisioned that a suitable halogenated-borane might work similar to that of trialkylboranes in the generation of reactive, yet stable enough halogen radicals for selective halogenation reactions (Scheme 1B).

Trialkylboranes readily undergo S_H_2 reactions because the formation of stronger B–X (*e.g.* B–O) bonds *via* substitution is highly exothermic.^3^ The BDEs (B–C) of BMe_3_, BEt_3_, B^*n*^Pr_3_, B^i^Pr_3_, and B^*n*^Bu_3_ range from 344 to 354 kJ mol^−1^ at 298 K, while their typical autoxidation products, B(OH)_3_, B(OMe)_3_, and B(OEt)_3_, have BDEs (B–O) ranging from 519 to 522 kJ mol^−1^ at 298 K.^7^ We hypothesized that organohaloboranes (BX_*a*_R_3−*a*_, X = halogen) with BDEs (B–X) similar to trialkylboranes would be a halogen radical donor from a thermodynamic viewpoint. As the common trihaloboranes (BX_3_) BF_3_, BCl_3_ and BBr_3_ have BDEs (B–X) of 644.3, 442.3 and 367.1 kJ mol^−1^ at 298 K, respectively, BBr_3_ was the logical option for our purpose.^8^ Although the BDE (B–I) of BI_3_ is the lowest among all trihaloboranes and found to be 278.2 kJ mol^−1^ at 0 K,^9^ it was not considered suitable as I_2_ has proven to be a very efficient radical quencher in such reactions,^10^ and even rigorously purified BI_3_ invariably contains a trace amount of I_2_.^11^

Compared to activated cyclopropanes,^12^ oxidative functionalization of unactivated cyclopropanes gives a wide range of useful molecules that are otherwise not readily accessible, and protocols for the Markovnikov-selective functionalization of unactivated cyclopropanes have been reported.^13–20^ Halolyses of cyclopropanes to give 1,3-dihaloalkanes by molecular halogens are also documented although the reactions commonly suffer from the formation of side products *via* electrophilic aromatic halogenation.^21^ In contrast, obtaining products with anti-Markovnikov regioselectivity has been considered as one of the top challenges in industry.^22–30^ Anti-Markovnikov functionalization of unactivated cyclopropanes mostly relies on photo-initiated radical processes with generally poor regioselectivity and limited scope.^31–36^ To the best of our knowledge, anti-Markovnikov hydrohalogenation of cyclopropanes has not been reported.

Very recently, an anti-Markovnikov hydroboration for unactivated cyclopropanes has been reported using boron tribromide and phenylsilane.^37^ The reaction was carried out under inert and anhydrous conditions, and mechanistic studies pointed to an ionic mechanism with Lewis acid–base interactions. We show that with a simple twist in the reaction conditions, which is to introduce oxygen, a drastically different reaction outcome and mechanism could be realized. We now report the study and application of BBr_3_ as a radical Br donor for the anti-Markovnikov addition of HBr to cyclopropanes.

With all these considerations in mind, we initially envisioned that BBr_3_/O_2_ as a suitable system to generate bromine radicals, and cyclopropylbenzene (**1a**) as the model substrate to capture them. The radical reaction might then be terminated by another halogen radical from reagents such as *N*-chlorosuccinimide or *N*-iodosuccinimide. Unfortunately, messy mixtures were obtained for all entries (see the ESI, Scheme S1†). On the other hand, a simple proton source, H_2_O, was found to be effective in terminating the radical species. In the control experiment with only BBr_3_ and cyclopropylbenzene (**1a**) (Scheme 2, entry 1), the anti-Markovnikov hydrobrominated product **2a** was obtained in 24% yield, together with the formation of Markovnikov product **3a** (trace) and dibrominated cyclopropane **4a** (11%). We reasoned that the proton source was the trace amount of moisture in commercial BBr_3_ solution.

**Scheme 2:**
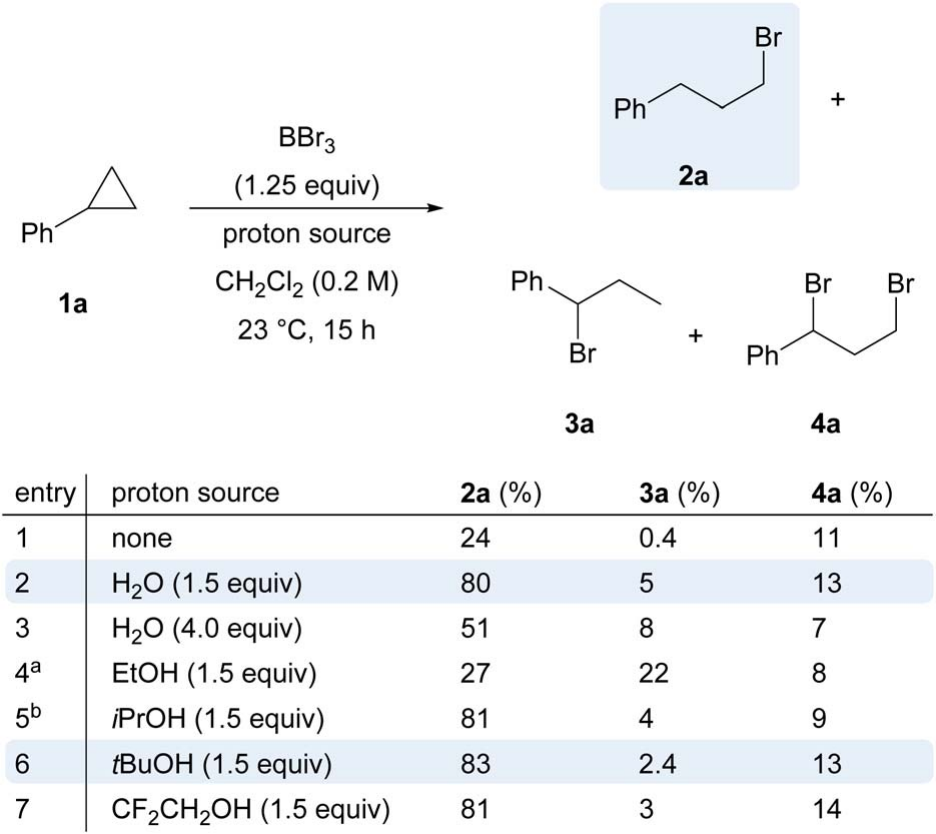
Reaction optimization. Conditions: reactions were carried out under ambient conditions and quenched by saturated NaHCO_3_ solution. Yields were measured by ^1^H NMR with CH_2_Br_2_ as the internal standard. ^a^24% of **1a** was recovered. ^b^6% of **1a** was recovered.

Although it is well known that boron-based Lewis acids are moisture sensitive,^38^ counter-intuitively, the addition of 1.5 equivalents of H_2_O had a positive impact on the yield of **2a**, which was dramatically improved to 80% (Scheme 2, entry 2). Excess water led to a reduction in the yield of **2a** and the regioselectivity (Scheme 2, entry 3). Replacing water with ethanol as the proton source resulted in a significant drop in reaction efficiency (Scheme 2, entry 4). In contrast, bulkier alcohols such as *i*-PrOH or *t*-BuOH (Scheme 2, entries 5 and 6) and less nucleophilic alcohols such as CF_3_CH_2_OH (Scheme 2, entry 7) gave comparable performance to that of water.

Further study revealed that achieving anti-Markovnikov addition of HBr to cyclopropanes in conventional systems is not a trivial task (Scheme 3). For instance, no reaction was observed when **1a** was treated with HBr in either aqueous or water/AcOH co-solvent systems at room temperature.^29^ Heating both reactions only yielded the Markovnikov product **3a** in 16–23% yield, and no anti-Markovnikov product **2a** was detected. The classical radical bromination protocol with BBr_3_/H_2_O_2_ only furnished dibrominated product **4a** in 29% yield. Similar to the uniqueness of BR_3_/O_2_ in several aforementioned radical reactions,^4^ the incapability of these control experiments in producing **2a** as a product contrasted starkly with our BBr_3_/O_2_ conditions, which generated a reactive yet selective bromine radical.

**Scheme 3:**
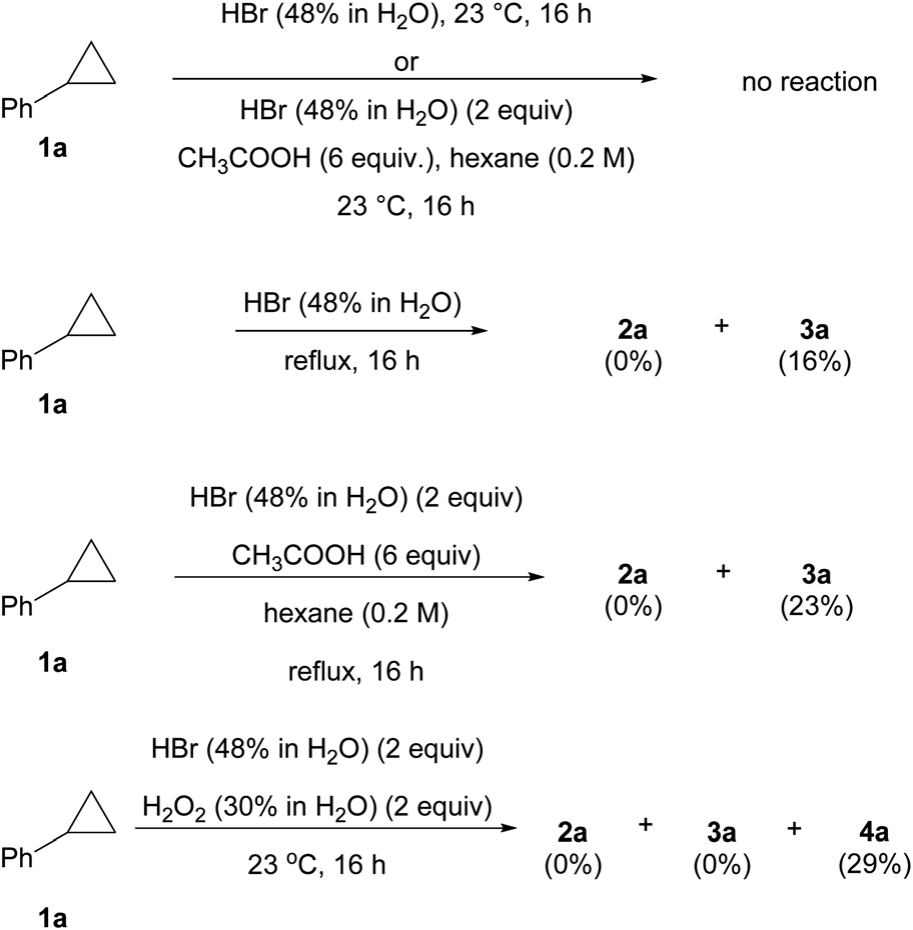
Reactions of cyclopropane (**1a**) with hydrobromic acid.

Next, we expanded the substrate scope to other unactivated cyclopropanes using either water or *t*-BuOH as the proton source (Scheme 4). Electron-neutral, deficient and sterically bulky substrates **1a–1g** gave the desired anti-Markovnikov products **2a–2g** in good yields and regioselectivity. Cyclopropanes with electron-deficient substituents including nitriles (**1j–n**) and ester (**1o**) also worked well with excellent regioselectivity. This protocol also exhibits high chemoselectivity towards cyclopropanes. Aryl methyl ether (**2i**), which is known to be easily cleaved by BBr_3_ even at low temperature, remained intact under our reaction conditions.^39^ Due to the tendency of aryl vinyl ketones to polymerize, they are known to be unsuitable for 1,4-conjugate additions mediated by trialkylboranes.^40^ Nevertheless, aryl cyclopropyl ketones (**1h–i**) were converted into the corresponding products in high yields, and polymerization was not observed. 1,1-Disubstituted (**1p**) and simple alkyl (**1q**) cyclopropanes were also compatible to give products **2p** and **2q**. When cyclopropyl carboxylic acid (**1r**) was used as the substrate, the unstable product **2r** was detected using HRMS and crude ^1^H NMR, and *γ*-butyrolactone was obtained ultimately through cyclization upon a basic work-up procedure. Indene-derived cyclopropyl substrate **1s** was also compatible to give **2s**. Scaled-up reactions were also performed on selected examples (**2a**, **2h**, **2o**, and **2r**) and excellent regioselectivities were still obtained.

**Scheme 4:**
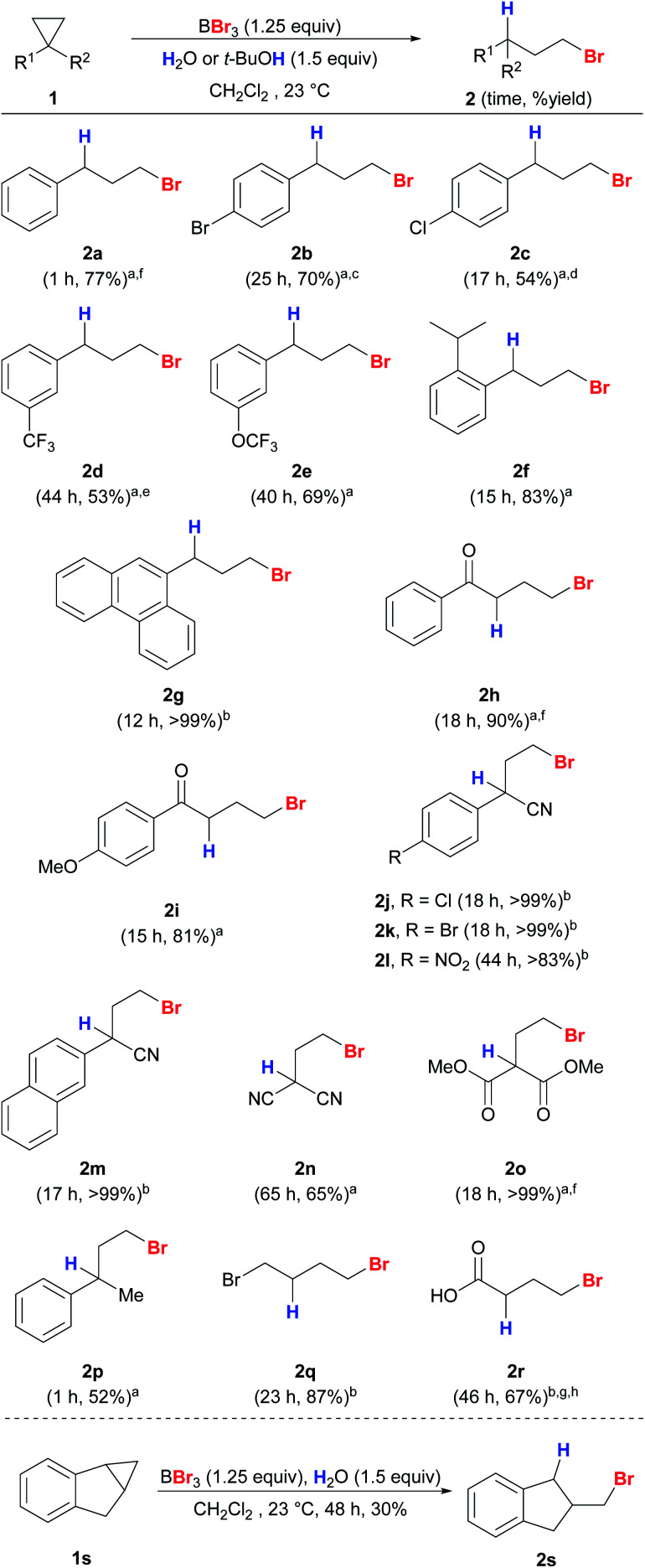
Reaction scope of anti-Markovnikov hydrobromination of cyclopropanes. Conditions: reactions were carried out with **1** (0.2 mmol) unless stated otherwise. Exact reaction conditions for each substrate are stated in the ESI.†^a^*t*-BuOH was used as the proton source. ^b^H_2_O was used as the proton source. ^c^4% of **3b** was detected. ^d^5% of **3c** was detected. ^e^7% of **3b** was detected. ^f^The reaction was conducted on a 1 mmol scale. ^g^The reaction was conducted on a 2 mmol scale. ^h^The product cyclized quickly upon work-up and the yield was measured on the basis of the cyclized product *γ*-butyrolactone.

Cyclopropanes **1t–1y** with secondary and tertiary alcohols also gave the corresponding anti-Markovnikov products in excellent yields and with high regioselectivities (Scheme 5). The structure of **2x** was confirmed unambiguously by X-ray crystallography.^41^ The hydroxyl groups in the substrates were converted into bromides simultaneously by the action of BBr_3_ to give a series of useful dibromides.^42^ We were interested in whether alcohol-containing substrates can be hydrobrominated in the absence of an external proton source. To our delight, **1t** was able to undergo anti-Markovnikov hydrobromination to give **2t** with only a slight drop in yield (76%), and **2u** was produced in quantitative yield. The hydroxyl groups in **1x** and **1y** appear to be crucial because a sluggish reaction was observed for 1-phenyl-2-methylcyclopropane that bears no hydroxyl substituent.

**Scheme 5:**
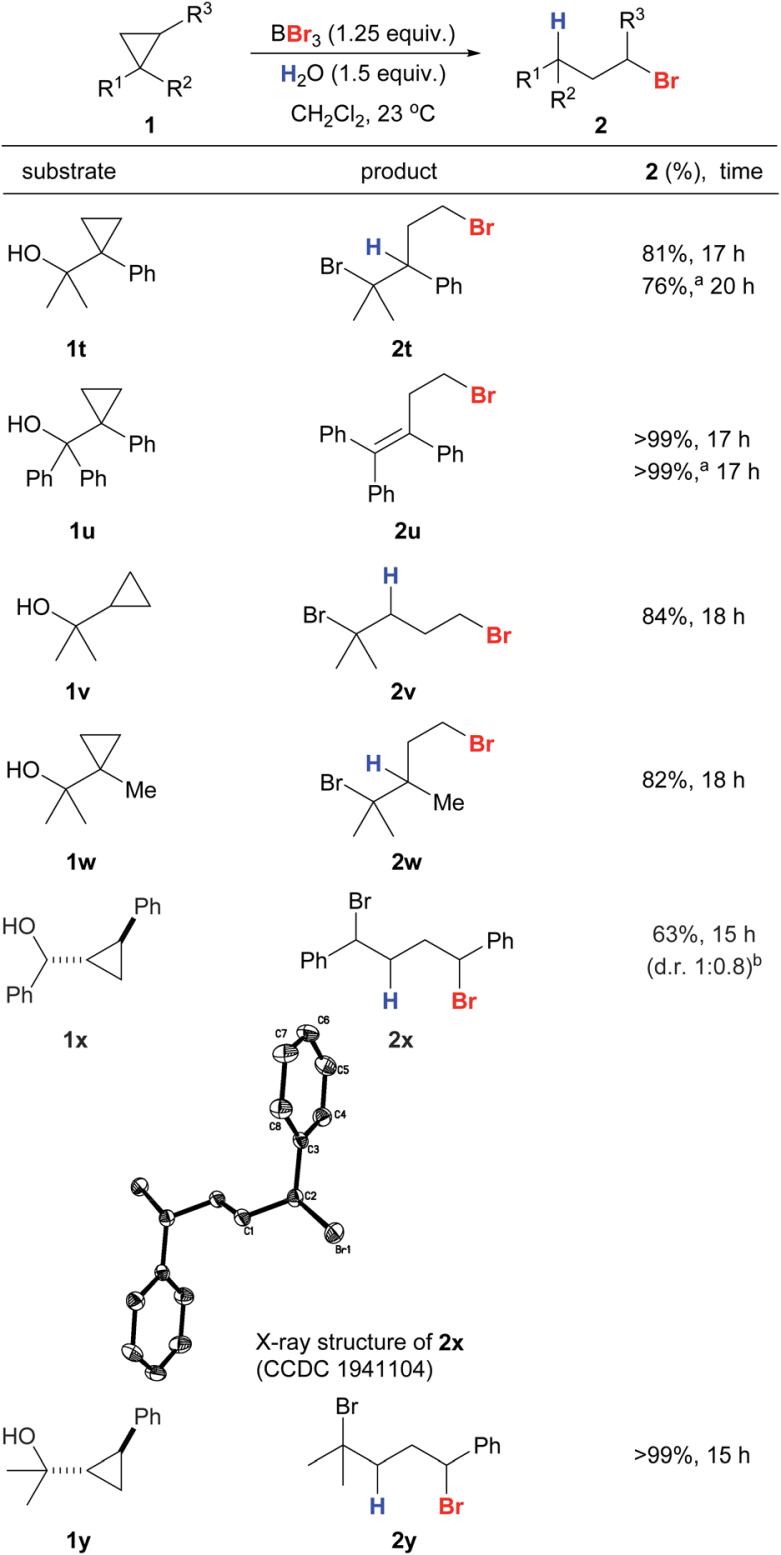
Reaction scope of anti-Markovnikov hydrobromination of cyclopropanes with hydroxyl substituents. Conditions: reactions were carried out with **1** (0.2 mmol). Exact reaction conditions for each substrate are stated in the ESI.†^a^Reaction was conducted in the absence of water. ^b^Diastereoselectivity was determined by a ^1^H NMR experiment on the crude mixture.

By substituting H_2_O and *t*-BuOH with D_2_O and *t*-BuOD, deuteriobrominations were also carried out and the corresponding mono-deuterium-labeled compounds were obtained smoothly (Scheme 6). Our protocol offered excellent regio-control in the mono-deuteriation to give **2-D**. Unactivated (**1a** and **1e**) and activated cyclopropanes (**1j–k**, **1m**, and **1o**) with various substituents worked well and excellent levels of deuterium incorporation were achieved.

**Scheme 6:**
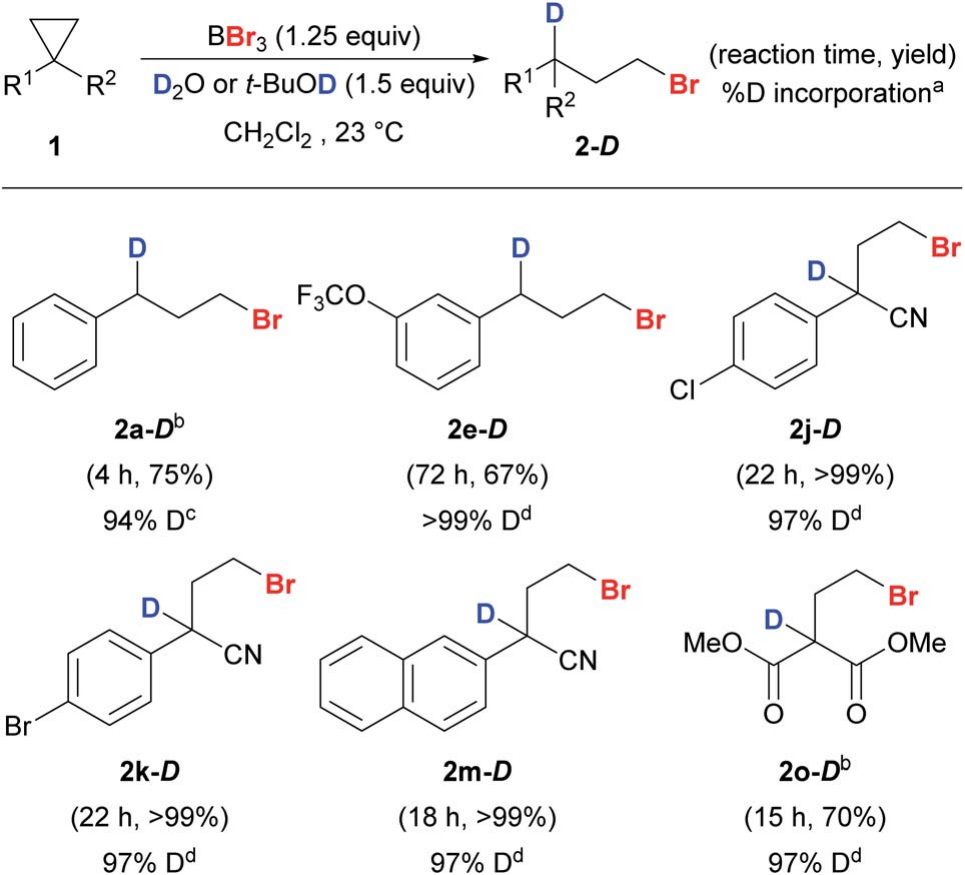
Reaction scope of anti-Markovnikov deuteriobromination of cyclopropanes. Conditions: reactions were carried out with **1** (0.2 mmol) unless stated otherwise. Exact reaction conditions for each substrate are stated in the ESI.†^a^The % D incorporation was determined based on the integration of the residual proton signal in ^1^H NMR. ^b^The reaction was conducted on a 1 mmol scale. ^c^*t*-BuOH was used as the deuterium source. ^d^D_2_O was used as the deuterium source.

The conversion of products **2** into primary alcohols and amines through nucleophilic substitution proved straightforward. For instance, alcohol **5a** and amine **5b** were readily prepared from **2a** with high conversion (Scheme 7). As the direct synthesis of primary alcohols and amines through anti-Markovnikov hydration and hydroamination has proven to be challenging,^22^ our protocol provides useful precursors for the synthesis of these highly desired compounds.

**Scheme 7:**
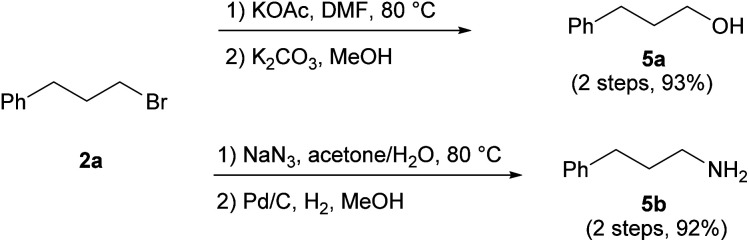
Synthetic utilities of **2a**.

We envision a radical reaction pathway between BBr_3_ and O_2_, but given the Lewis acidity of BBr_3_ and Lewis basicity of H_2_O and alcohols, an acid-mediated pathway cannot be ruled out.^38^ However, such a pathway appears highly unlikely, as the treatment of cyclopropanes with aqueous HBr yielded no anti-Markovnikov product **2** (Scheme 3). Several control experiments were performed to further probe the reaction mechanism.

The addition of a radical scavenger, BHT or TEMPO, in slight excess of BBr_3_ completely shut down the formation of anti-Markovnikov product **2a**, and a significant amount of Markovnikov product **3a** was detected (Scheme 8A). The addition of the acceptor olefin acrylonitrile completely suppressed the reaction. The absence of light had no impact on the reaction, thereby eliminating the possibility of a photo-triggered pathway. The presence of oxygen was crucial for both the yield and the regioselectivity. The reaction proceeded smoothly to give the desired product **2a** (80%) in open air. In contrast, the yield of anti-Markovnikov product **2a** dropped to 14% and that of the Markovnikov product **3a** increased to 17% when the reaction was conducted with degassed CH_2_Cl_2_ and **1a**. Deuteriobromination of **1g** was also conducted with *t*-BuOD as the deuterium source (Scheme 8B). Other than the benzylic deuteriation product **2g-D** (25%), a substantial amount of **2g-D′** (75%) was obtained. In contrast, no aromatic deuteriation was observed when phenanthrene (**6**) was used as the substrate under the same conditions. The formation of **2g-D′** could be attributed to the isomerization of benzylic radical species (also see the ESI, Fig. S1†). This preliminary evidence pointed at a radical mechanism, although a carbocation intermediate cannot be ruled out completely.

**Scheme 8:**
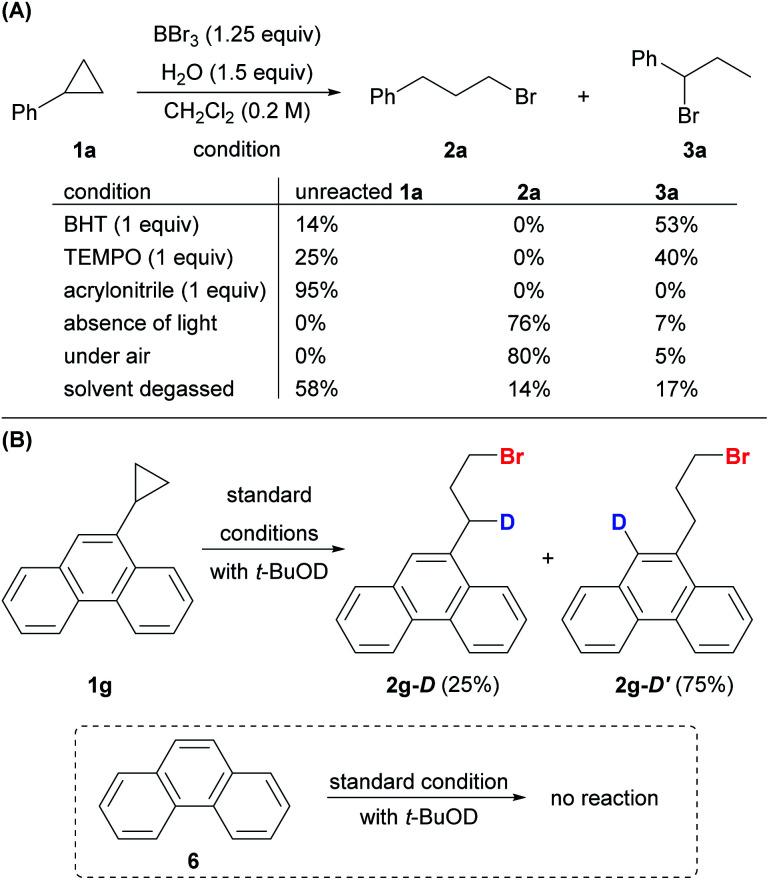
Control experiments.

Consistent with literature reports on BR_3_,^43,44^ the reactivity of BBr_3_ towards homolytic debromination decreases sharply along the series BBr_3_, BBr_2_OR, and BBr(OR)_2_ as a consequence of π-bonding between oxygen and boron. With 0.5 equiv BBr_3_, only 21% of **2a** was obtained even with a prolonged reaction time of 24 h. These data indicated that only the first equivalent of Br from BBr_3_ is crucial for the reactivity, and contribution from the possible BBr_*a*_(OR)_3−*a*_ byproducts should be insignificant.

A series of ^1^H and ^11^B NMR experiments were conducted to gain further insight. Upon mixing BBr_3_ with **1a** in the absence of O_2_ and a proton source, both **1a** and BBr_3_ were mostly consumed, and a new ^11^B signal at 64 ppm (see the ESI, Fig. S2†) emerged as a singlet, which is characteristic of an alkyldihaloborane species.^45,46^ From ^1^H NMR, it is clear that **1a** is ring-opened (see the ESI, Fig. S4†), and the species has a similar NMR pattern to a hydroborated cyclopropane, which has been reported as a reaction intermediate in literature examples (see the ESI, Fig. S3†).^37^ Direct bromoboration of alkynes or allenes with BBr_3_ is well documented.^47,48^ While this new species cannot be clearly identified, it is speculated that it could be the direct bromoboration product or hydroxyboration product. Nevertheless, it is clear that the interaction between BBr_3_ and **1a** does not lead to the anti-Markovnikov product **2a** in the absence of O_2_ and a proton source.

When *i*-PrOH and BBr_3_ were mixed in CD_2_Cl_2_ under air, the ^11^B signal of BBr_3_ (39 ppm) disappeared and a new signal at 25.0 ppm emerged. A new proton signal at −2.68 ppm also appeared in the ^1^H NMR study of the same sample. The two new signals (25.0 ppm in ^11^B NMR and −2.68 ppm in ^1^H NMR) diminished gradually upon the addition of **1a** and the amount of anti-Markovnikov product **2a** increased accordingly (see the ESI, Fig. S5†). On the other hand, a new ^11^B NMR signal at 18.9 ppm (but no signal at 25.0 ppm) was observed when the same mixture was prepared in the absence of O_2_ and attributed to the formation of the Lewis adduct between *i*-PrOH and BBr_3_ (see the ESI, Fig. S6†). Thus, it is reasonable to propose that the active species, responsible for initiating the anti-Markovnikov hydrobromination of cyclopropanes, was formed only in the presence of O_2_.

A DFT computational study was also performed to shed light on the mechanism (Fig. 1). While there are no reports on radical reactions triggered by BBr_3_/O_2_, we speculate that the reaction mechanism might be analogous to the classical BR_3_/O_2_ system in which the putative peroxy-boron species **A** is generated^49^ at the initiation stage of the radical process (Fig. 1A) and corresponds to the new NMR signals (25.0 ppm in ^11^B NMR and −2.68 ppm in ^1^H NMR).^3,50,51^ Based on the calculated energy profile, species **A** is capable of brominating cyclopropane **1a** through a radical mechanism to give **B** (Fig. 1B). It is also calculated that **A** and **A′** could be in equilibrium, but species **A** (Δ*G* = −7.6 kcal mol^−1^) was found to be a more competent Br donor than **A′** (Δ*G* = 0.6 kcal mol^−1^) in the halogen atom transfer (XAT), potentially due to the intramolecular hydrogen bond that stabilizes the by-product **I** (Fig. 1C). It was also calculated that BBr_3_ can reversibly react with water to give adduct **C** (^11^B NMR signal = 18.9 ppm). Species **C** is unable to serve as a Br radical donor to brominate cyclopropane **1a** (Δ*G* = 69.6 kcal mol^−1^).

**Fig. 1:**
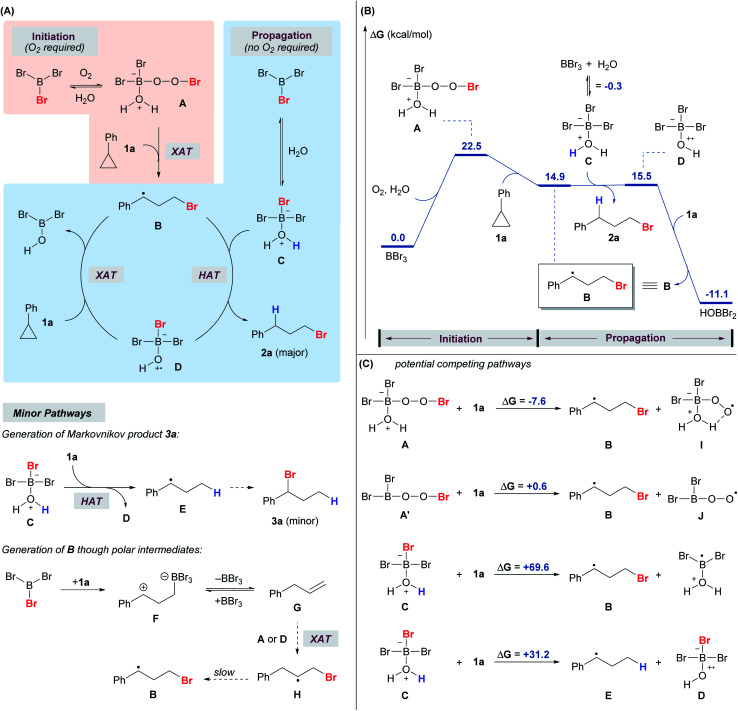
Reaction mechanism. (A) Plausible reaction pathways. (B) Calculated free energy profile of the anti-Markovnikov hydrobromination of **1a** at the ωB97X-D/6-311++G(d,p), SMD(CH_2_Cl_2_)//ωB97X-D/6-31+G(d,p) level of theory. (C) Potential competing pathways.

However, species **C** is capable of acting as a hydrogen radical donor to species **B**, furnishing the desired product **2a**. This result is in alignment with the proposal in the literature in which trialkylborane-ROH complexes (R = H, Me) might act as H-donors as a result of the weakened O–H bond.^52^ Species **D**, which is formed from species **C** after the hydrogen atom transfer (HAT), was calculated to be a competent Br radical donor to brominate cyclopropane **1a** to give **B**, thereby propagating the radical chain. Thus, we propose that oxygen is required only in the initiation stage for the generation of species **A**, while species **C** and **D** are responsible for propagation. Indeed, the reaction was sluggish under an inert atmosphere, while the re-introduction of oxygen into a system initially free of oxygen triggered the anti-Markovnikov hydrobromination (see the ESI, Scheme S2†). The HAT from species **A** to **1a** was also explored computationally, but species **A** could not be optimized as a stable energy minimum. Species **C** may also serve as a hydrogen radical donor and react with cyclopropane **1a** to give species **D** and **E**, which would go on to produce the Markovnikov product **3a**. However, this hydrogen atom transfer reaction is endergonic by 31.2 kcal mol^−1^ (Fig. 1C), making it a minor pathway compared to the competing hydrogen atom transfer from **C** to **B** that gives **D** and **2a** (Fig. 1A). This result is consistent with the experimental observation that the Markovnikov product **3a** became dominant when the reaction was conducted under an inert atmosphere (Scheme 8A).

In the ^1^H NMR study of the reaction using **1a**, apart from **2a**, **3a** and **4a** (Scheme 2), a trace amount of allylbenzene was detected initially and diminished over time. We speculate that the allylbenzene (Fig. 1A, species **G**) might be formed through the zwitterionic species **F** as proposed in the recent studies by Wang and Shi.^37,53^ The eventual disappearance of allylbenzene could be attributed to the radical bromination to give species **H** and the subsequent formation of **2a**.

In a deuterium labeling experiment with **1a** as the substrate and D_2_O as the deuterium source, we observed exclusive deuterium incorporation at the benzylic carbon to give product **2a-D**, potentially through the C(1) radical species **B1** (Scheme 9, eqn (1)). However, the deuterium incorporation pattern is vastly different when using allylbenzene instead of **1a**, for which C(2) deuterated product **2a-D′** was obtained predominately (Scheme 9, eqn (2)) (also see the ESI, Fig. S7†). The formation of **2a-D′** from allylbenzene may proceed through the C(2) radical species **H1**. A small amount of **2a-D** (9%) was also detected in the reaction with allylbenzene, attributed to the slow 1,2-hydrogen shift^54^ converting **H1** to the more stable benzylic radical **B1**. These results suggest that the 1,2-hydrogen shift between the radical species **H** and **B** (Fig. 1A) should be much slower than the radical protonation process, implying that allylbenzene is unlikely to be the key intermediate in the reaction.

**Scheme 9:**
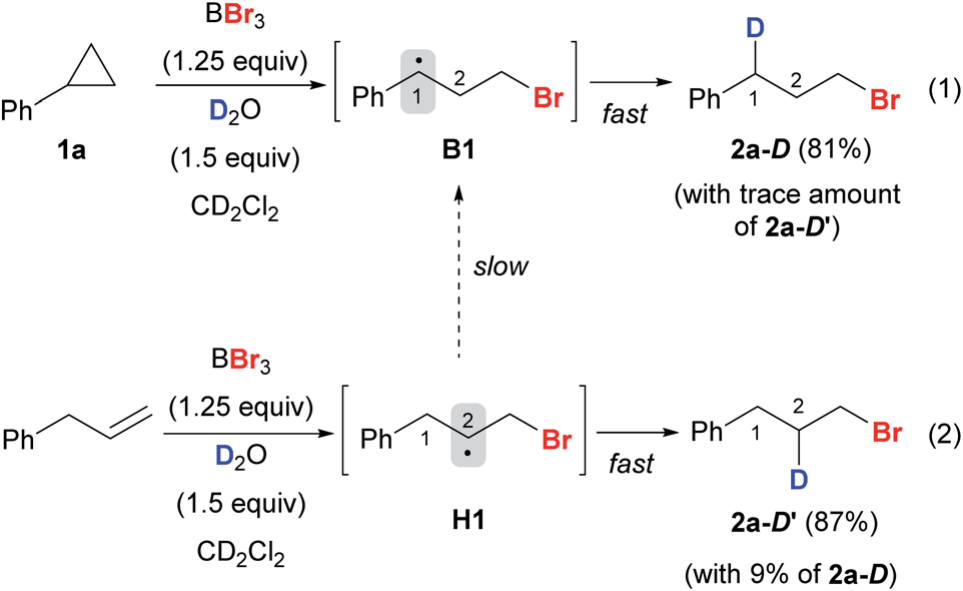
Mechanistic insights from deuteriobromination.

## Conclusions

In summary, we have discovered and exploited the potential of BBr_3_ to serve as a bromine radical donor in the presence of O_2_ and a proton source. Through our protocol, cyclopropanes are opened regioselectively to obtain anti-Markovnikov hydro- and deuteriobrominated products in high yields. Mechanistic studies and DFT calculations demonstrate the importance of O_2_ in the radical initiation process. Further efforts to utilize this reactivity mode of BBr_3_ on different classes of substrates are currently underway in our laboratory.

## Conflicts of interest

There are no conflicts to declare.

## Supplementary Material

SC-011-D0SC02567D-s001

SC-011-D0SC02567D-s002
