# Supplementary material for: Boron tribromide as a reagent for anti-Markovnikov addition of HBr to cyclopropanes
Source: Chem Sci. 2020 Aug 4;11(35):9426–33. doi: 10.1039/d0sc02567d (PMC8161534; doi:10.1039/d0sc02567d)

Electronic Supplementary Material (ESI) for Chemical Science.
This journal is © The Royal Society of Chemistry 2020

Electronic Supplementary Information

Boron Tribromide as Reagent for Anti-Markovnikov Addition of HBr to Cyclopropanes

Matthew H. Gieuw,[a] Shuming Chen,[b] Zhihai Ke,[a]
K. N. Houk,*,[b] Ying-Yeung Yeung*,[a]

[a]Department of Chemistry and State Key Laboratory of Synthetic Chemistry, The Chinese University of Hong Kong, Shatin, NT, Hong Kong, HKSAR

[b]Department of Chemistry and Biochemistry, University of California, Los Angeles, California 90095, United States

E-mail: houk@chem.ucla.edu, yyyeung@cuhk.edu.hk; Tel: +852-3943-6377

Table of Contents

**S2**	**A: General Information**
**S3 – S14**	**B: Preparation of Substrates**
**S15 – S33**	**C: Procedures of Hydro- and Deuteriobromination**
**S34 – S35**	**D: Further Transformations of 2a**
**S36 – S37**	**E: Control Experiments**
**S38– S45**	**F: NMR Studies**
**S46 – S51**	**G: Computational Studies**
**S52**	**H: References**
**S53 – S151**	**^{1}H and ^{13}C NMR Spectra**

(A) General Information

All reactions requiring anhydrous conditions were conducted by standard procedures under nitrogen atmosphere. The solvents were dried over a solvent purification system from Innovative Technology. Melting points were determined on a STUART SMP40 melting point apparatus. Infrared spectra (IR) were recorded on KBr plate with Nicolet iS10 FTIR with peaks reported in cm^{-1}. Relative intensities were indicated as s (strong, 0-33% T); m (34-66% T); w (weak 67-100% T). ^{1}H NMR, ^{11}B NMR, ^{13}C NMR, and ^{19}F NMR spectra were recorded on a Bruker AMX500 (500 MHz) spectrometer or a Bruker AMX400 (400 MHz) spectrometer. Proton and carbon chemical shifts are reported in parts per million (ppm) values downfield from TMS (δ 0.00) and referenced to residual protons in NMR solvents ($CDCl_3$ at δ 7.26, CD_2Cl_2 at δ 5.32) or carbon signals in NMR solvent ($CDCl_3$ at δ 77.16). High resolution mass spectra were obtained on a ThermoFinnigan MAT 95XL spectrometer (ionization mode: ESI or EI) or Thermo QEF mass spectrometer (ionization mode: ESI or APCI/DIP). Analytical thin layer chromatography (TLC) was performed with Merck pre-coated TLC plates, silica gel 60F-254, layer thickness 0.25 mm. Flash chromatography separations were performed on Merck 60 (0.040-0.063 mm) mesh silica gel. Cyclopropylbenzene **1a** (97%, Aldrich), cyclopropyl phenyl ketone **1h** (TCI chemical), cyclopropyl 4-methoxyphenyl ketone **1i** (TCI chemical), dimethyl-1,1-cyclopropanedicarboxylate **1o** (TCI chemical), (bromomethyl)cyclopropane **1q** (Accela), cyclopropylcarboxylic acid **1r** (J&K chemical), boron tribromide (J&K chemical), D_2O (99% D) (Cambridge), *t*-BuOD (99% D) (Aldrich) were used as received.

(B) Preparation of Substrates

(a) Preparation of aryl substrates **1b-g**

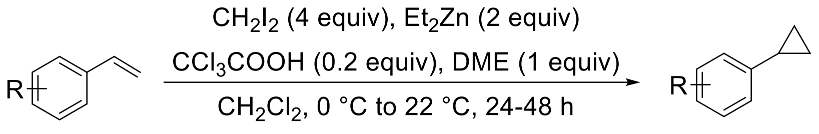

1

Method A: general procedure for the preparation of cyclopropane substrates 1 by Simmons-Smith cyclopropanation.[1] Anhydrous CH_2Cl_2 (10 mL) was added to a flame-dried round bottom flask equipped with a dropping funnel and a magnetic stir bar. The solution was cooled to 0 °C and Et_2Zn in hexanes (1.0 M, 10 mL, 10 mmol, 2 equiv) was added via syringe over 30 minutes. A solution of CH_2I_2 (1.6 mL, 20 mmol, 4 equiv) in CH_2Cl_2 (5 mL) was then added dropwise over 30 minutes into the solution and the resultant mixture was allowed to stir for 45 minutes at 0 °C. Next, a stock solution of CCl_3COOH (492 mg, 3 mmol, 0.6 equiv) and dimethoxyethane (1.56 mL, 15 mmol, 3 equiv) in CH_2Cl_2 (15 mL) was prepared, and 5 mL of this stock solution was added to the reaction mixture dropwise over 30 minutes. The resultant mixture was allowed to stir for another 45 minutes at 0 °C. A solution of substituted styrene (5 mmol, 1 equiv) in CH_2Cl_2 (5 mL) was then added into the solution at 0 °C and the resultant mixture was allowed to stir for 24-48 hours at 22 °C. The reaction was quenched by water (10 mL) and aqueous HCl (5 M, 4 mL). The organic layer was separated and the aqueous layer was extracted with CH_2Cl_2 (10 mL × 3). The combined organic layer was washed with brine (5 mL), dried with anhydrous Na_2SO_4, filtered, and concentrated under reduced pressure. The residue was purified by flash column chromatography on silica gel (diethyl ether in *n*-hexane) to provide **1**.

*Note: **1b, 1c, 1p** are volatile and was carefully dried with rotavap at ~10 mbar in a 25 °C water bath*

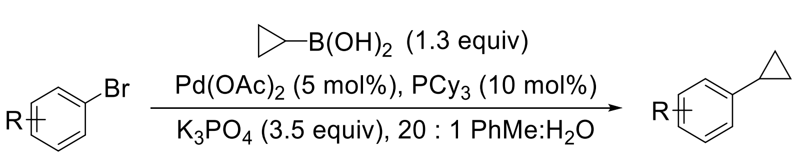

1

Method B: general procedure for the preparation of cyclopropane substrates 1 by Suzuki coupling.[2] To a 100 mL Schlenk tube, the substituted aromatic bromide (5 mmol, 1 equiv), K_3PO_4 (3.7 g, 17.5 mmol, 3.5 equiv), tricyclohexylphosphine (140 mg, 0.5 mmol, 0.1 equiv), cyclopropylboronic acid (558 mg, 6.5 mmol, 1.3 equiv), palladium(II) acetate (56 mg, 0.25 mmol, 0.05 equiv) and toluene:water (21 mL, 20:1 v/v) were added. The Schlenk tube was then placed in a pre-heated oil bath at 110 °C.

After 10 hours, the reaction mixture was cooled to room temperature, diluted with H_2O (50 mL) and extracted with ethyl acetate (50 mL × 3). The combined organic layer was then dried over Na_2SO_4, filtered and concentrated under reduced pressure. The residue was purified by flash column chromatography on silica gel (diethyl ether in *n*-hexane) to provide **1.**

*Note: **1d, 1e, 1f** are volatile and was carefully dried with rotavap at ~10 mbar in a 25 °C water bath*

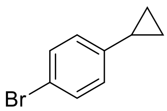

1b

Synthesized by Method **A**

1b: Colourless oil (90% yield, 887 mg)

^{1}H NMR (CDCl$_3$, 400 MHz) : δ 7.37 (d, *J* = 6.7 Hz, 2H), 6.95 (d, *J* = 8.4 Hz, 2H), 1.89–1.83 (m, 1H), 1.01–0.96 (m, 2H), 0.69–0.65 (m, 2H)

^{13}C NMR (CDCl$_3$, 100.6 MHz) : δ 143.1, 131.3, 127.5, 118.9, 15.1, 9.5

Data matches with literature reported values (D. Cheng, D. Huang, Y. Shi, *Org. Biomol. Chem*. 2013, **11**, 5588.)

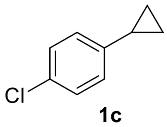

1c

Synthesized by Method **A**

1c: Colourless oil (82% yield, 626 mg)

^{1}H NMR (CDCl$_3$, 400 MHz) : δ 7.23 (d, *J* = 8.5 Hz, 2H), 7.01 (d, *J* = 8.4 Hz, 2H), 1.92–1.85 (m, 1H), 1.01–0.97 (m, 2H), 0.70–0.66 (m, 2H)

^{13}C NMR (CDCl$_3$, 100.6 MHz) : δ 142.6, 130.1, 128.4, 127.1, 15.0, 9.4

Data matches with literature reported values (Y.-Y. Zhou, C. Uyeda, *Angew. Chem.* 2016, **128**, 3223.)

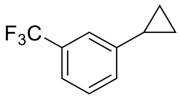

1d

Synthesized by Method **B**

1d: Colourless oil (92% yield, 856 mg)

R_f = 0.77 (*n*-hexane : diethyl ether = 40 :1)

IR (KBr): 3061 (w), 2360 (w), 1643 (w), 1076 (w)

^{1}H NMR (CDCl$_3$, 500 MHz) : δ 7.41–7.33 (m, 3H), 7.24 (d, *J* = 8.0 Hz, 1H), 1.96 (tt,

J = 8.5, 5.0 Hz, 1H), 1.05–1.01 (m, 2H), 0.76–0.73 (m, 2H)

^{13}C NMR (CDCl$_3$, 125.7 MHz) : δ 145.2, 130.7 (q, J = 31.8 Hz), 129.1, 128.8, 124.4 (q, J = 272.1 Hz), 122.6 (q, J = 3.8 Hz), 122.3 (q, J = 3.8 Hz), 15.5, 9.6

^{19}F NMR (CDCl$_3$, 470.6 MHz) : δ –62.6 (s)

HRMS (APCI/DIP) Calc'd for $C_{10}H_9F_3$ $[M]^+$: 186.06509; found 186.06524

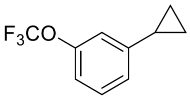

1e

Synthesized by Method **B**

1e: Colourless oil (95% yield, 960 mg)

R_f = 0.63 (*n*-hexane : diethyl ether = 40 :1)

IR (KBr): 3062 (w), 2360 (w), 1635 (m)

^{1}H NMR (CDCl$_3$, 500 MHz) : δ 7.26 (t, J = 7.5 Hz, 1H), 7.00 (dd, J = 8.0, 2.0 Hz, 2H), 6.91 (s, 1H), 1.91 (tt, J = 8.5, 5.0 Hz, 1H), 1.03–0.99 (m, 2H), 0.73–0.70 (m, 2H)

^{13}C NMR (CDCl$_3$, 125.7 MHz) : δ 149.6, 146.8, 129.6, 124.2, 120.7 (q, J = 256.7 Hz), 118.4, 117.9, 15.4, 9.7

^{19}F NMR (CDCl$_3$, 470.6 MHz) : δ –57.7 (s)

HRMS (APCI/DIP) Calc'd for $C_{10}H_9F_3O$ $[M+H]^+$: 203.06783; found 203.06776

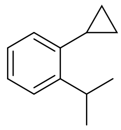

1f

Synthesized by Method **B**

1f: Colourless oil (78% yield, 625 mg)

R_f = 0.80 (*n*-hexane : diethyl ether = 40 :1)

IR (KBr): 3062 (w), 1642 (m), 1077 (m), 749 (w)

^{1}H NMR (CDCl$_3$, 500 MHz) : δ 7.29 (dd, J = 8.0, 1.5 Hz, 1H), 7.22 (td, J = 7.5, 1.0 Hz, 1H), 7.14 (td, J = 7.5, 1.0 Hz, 1H), 7.04 (d, J = 8.0 Hz, 1H), 3.62 (septet, J = 7.0 Hz, 1H), 2.03 (tt, J = 8.5, 5.5 Hz, 1H), 1.31 (d, J = 6.5 Hz, 6H), 0.98–0.94 (m, 2H), 0.71–0.68 (m, 2H)

^{13}C NMR (CDCl$_3$, 125.7 MHz) : δ 148.4, 139.9, 126.2, 126.1, 125.7, 124.8, 28.8, 23.7, 13.0, 7.2

HRMS (APCI/DIP) Calc'd for $C_{12}H_{16}$ $[M+H]^+$: 161.13248; found 161.13253

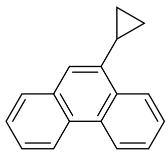

1g

Synthesized by Method **B**

1g: Light brown solid (86% yield, 939 mg)

m.p.: 132–133 °C

R_f = 0.67 (*n*-hexane : diethyl ether = 40 :1)

IR (KBr): 3060 (w), 1637 (m), 735 (w)

^{1}H NMR ($CDCl_3$, 500 MHz) : δ 8.77 (d, *J* = 9.0 Hz, 2H), 8.37 (s, 1H), 8.00 (d, *J* = 8.5 Hz, 2H), 7.54–7.45 (m, 4H), 2.52–2.47 (m, 1H), 1.49–1.45 (m, 2H), 0.85–0.82 (m, 2H)

^{13}C NMR ($CDCl_3$, 125.7 MHz) : δ 134.9, 131.8, 131.6, 129.0, 126.5, 126.1, 125.1, 124.9, 10.6, 9.5

HRMS (APCI/DIP) Calc'd for $C_{17}H_{14}$ $[M]^+$: 218.10900; found 218.10900

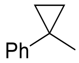

1p

Synthesized by Method **A**

1p: Colourless oil (74% yield, 489 mg)

^{1}H NMR ($CDCl_3$, 400 MHz) : δ 7.32–7.26 (m, 4H), 7.20–7.15 (m, 1H), 1.43 (s, 3H), 0.93–0.87 (m, 2H), 0.76–0.73 (m, 2H)

^{13}C NMR ($CDCl_3$, 100.6 MHz) : δ 147.2, 128.3, 126.8, 125.6, 25.9, 19.9, 15.8

Data matches with literature reported values (D. Cheng, D. Huang, Y. Shi, *Org. Biomol. Chem*. 2013, **11**, 5588.)

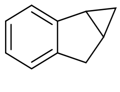

1s

Synthesized by Method **A**

1s: Light yellow oil (99% yield, 625 mg)

^{1}H NMR ($CDCl_3$, 400 MHz) : δ 7.33–7.26 (m, 1H), 7.18–7.09 (m, 3H), 3.21 (dd, *J* = 16.9, 6.7 Hz, 1H), 2.96 (d, *J* = 16.7 Hz, 1H), 2.41–2.36 (m, 1H), 1.90–1.84 (m, 1H), 1.08 (td, *J* = 8.0, 4.3 Hz, 1H), 0.06 (q, J = 3.9 Hz, 1H)

^{13}C NMR ($CDCl_3$, 100.6 MHz) : δ 147.2, 142.0, 126.0, 125.56, 125.46, 123.5, 35.6, 24.0, 16.9, 16.1

Data matches with literature reported values (J. C. Lorenz, J. Long, Z. Yang, S. Xue, Y. Xie, Y. Shi, *J. Org. Chem*. 2004, **69**, 327-334.)

(b) Preparation of nitrile substrates **1j-m**

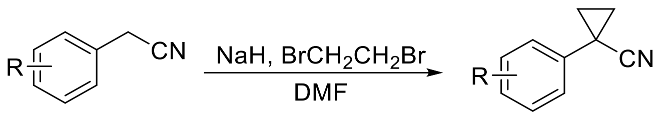

1j-m

This procedure follows the procedure of a previously reported literature.[3]

To a stirred suspension of NaH (60% in mineral oil, 2.4 g, 60 mmol, 3 equiv) in DMF (10 mL) at 0 °C was added a solution of benzyl cyanide (2.3 g, 20 mmol, 1 equiv) in DMF (10 mL) dropwise. The resultant mixture was stirred for 15 minutes at the same temperature. A solution of 1,2-dibromoethane (2.6 mL, 30 mmol, 1.5 equiv) in DMF (10 mL) was then added dropwise and the resultant mixture was stirred at the same temperature for an additional 18 hours. The reaction was quenched with H_2O (150 mL) and the aqueous layer was extracted with diethyl ether (3 × 100 mL). The combined organic layer was washed with brine (50 mL), dried over anhydrous $MgSO_4$, filtered, and concentrated under reduced pressure. The residue was purified by flash chromatography eluted with *n*-hexane/diethyl ether to yield **1j-m**.

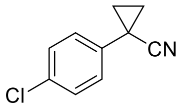

1j

1j: Light brown solid (89% yield, 3162 mg)

m.p.: 53–54 °C

R_f = 0.17 (*n*-hexane : diethyl ether = 8 :1)

IR (KBr): 3061 (w), 2237 (w), 1638 (m), 803 (w), 507 (w)

^{1}H NMR ($CDCl_3$, 500 MHz) : δ 7.31 (d, *J* = 8.5 Hz, 2H), 7.22 (d, *J* = 8.5 Hz, 2H), 1.74–1.72 (m, 2H), 1.39–1.36 (m, 2H)

^{13}C NMR ($CDCl_3$, 125.7 MHz) : δ 134.7, 133.7, 129.1, 127.3, 122.2, 18.3, 13.5

HRMS (APCI/DIP) Calc'd for $C_{10}H_8ClN$ $[M+H]^+$: 178.04180; found 178.04209

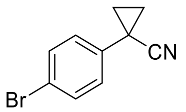

1k

1k: Light yellow solid (89% yield, 3953 mg)

m.p.: 84–86 °C

R_f = 0.17 (*n*-hexane : diethyl ether = 8 :1)

IR (KBr): 3061 (w), 2361 (w), 2234 (w), 1641 (m), 1490 (w), 1073 (m)

^{1}H NMR ($CDCl_3$, 500 MHz) : δ 7.47 (d, *J* = 8.5 Hz, 2H), 7.16 (d, *J* = 8.5 Hz, 2H),

1.75–1.72 (m, 2H), 1.39–1.37 (m, 2H)

^{13}C NMR ($CDCl_3$, 125.7 MHz) : δ 135.3, 132.1, 127.6, 122.2, 121.7, 18.4, 13.6

HRMS (APCI/DIP) Calc'd for $C_{10}H_8BrN$ $[M+H]^+$: 221.99129; found 221.99130

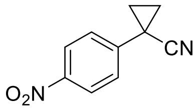

1l

1l: yellow solid (83% yield, 3124 mg)

m.p.: 153–155 °C

R_f = 0.16 (*n*-hexane : diethyl ether = 2 :1)

IR (KBr): 3060 (w), 2361 (w), 1637 (m), 1514 (w), 1347 (w)

^{1}H NMR ($CDCl_3$, 500 MHz) : δ 8.22 (d, *J* = 9.0 Hz, 2H), 7.43 (d, *J* = 8.5 Hz, 2H), 1.92–1.90 (m, 2H), 1.55–1.53 (m, 2H)

^{13}C NMR ($CDCl_3$, 125.7 MHz) : δ 147.3, 143.7, 126.1, 124.3, 121.3, 20.1, 14.2

HRMS (APCI/DIP) Calc'd for $C_{10}H_8N_2O_2$ $[M+H]^+$: 189.06584; found 189.06584

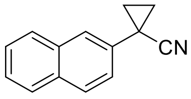

1m

1m: Light yellow solid (86% yield, 3324 mg)

m.p.: 42–44 °C

R_f = 0.20 (*n*-hexane : diethyl ether = 8 :1)

IR (KBr): 3061 (w), 2361 (w), 2340 (w), 1639 (m), 1083 (w)

^{1}H NMR ($CDCl_3$, 500 MHz) : δ 7.84–7.82 (m, 4H), 7.54–7.48 (m, 2H), 7.32 (dd, *J* = 9.0, 2.0 Hz, 1H), 1.80–1.78 (m, 2H), 1.52–1.50 (m, 2H)

^{13}C NMR ($CDCl_3$, 125.7 MHz) : δ 133.3, 133.2, 132.6, 129.0, 127.8, 127.7, 126.8, 126.4, 125.2, 123.1, 122.7, 18.2, 14.1

HRMS (APCI/DIP) Calc'd for $C_{14}H_{11}N$ $[M+H]^+$: 194.09643; found 194.09634

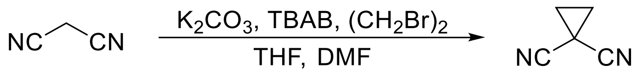

1n

To a solution of THF (6 mL) and DMF (0.2 mL), K_2CO_3 (2.1 g, 15 mmol, 1 equiv), malononitrile (990 mg, 15 mmol, 1 equiv), 1,2-dibromoethane (1.3 mL, 15 mmol, 1 equiv) and tetrabutylammonium bromide (193 mg, 0.6 mmol, 0.04 equiv) were added and the resultant mixture was stirred for 3 days. The reaction mixture was then diluted with H_2O (20 mL) and ethyl acetate (20 mL). The aqueous solution was extracted with ethyl acetate (3 × 20 mL). The combined organic layer was washed with brine (20 mL), dried over Na_2SO_4, filtered, and concentrated under reduced pressure. The residue was purified by flash chromatography eluted with dichloromethane/*n*-hexane to yield **1n** as light yellow oil (21% yield, 290 mg).

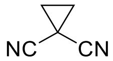

1n

R_f = 0.33 (dichloromethane/*n*-hexane = 2 : 1)

IR (KBr): 3059 (w), 1642 (m), 1079 (m)

^{1}H NMR ($CDCl_3$, 400 MHz) : δ 1.82 (s)

^{13}C NMR ($CDCl_3$, 100.6 MHz) : δ 115.3, 18.7, −1.2

HRMS (EI) Calc'd for $C_5H_4N_2$ $[M]^+$: 92.0369; found 92.03698

(c) Preparation of hydroxyl substrates **1t-y**

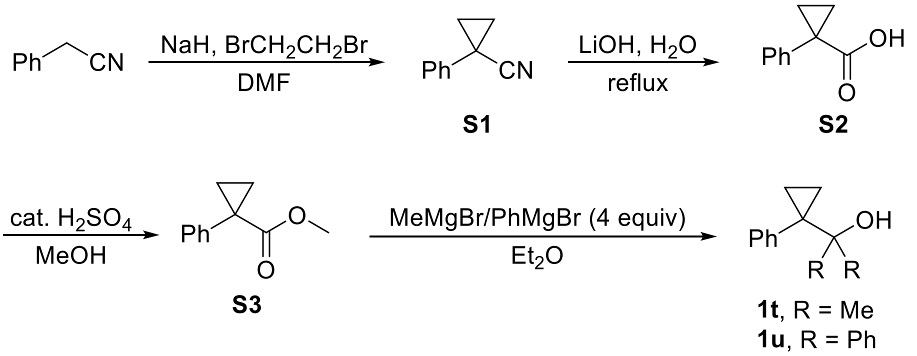

This procedure follows the procedure of a previously reported literature.[3]
To a stirred suspension of NaH (60% in mineral oil, 2.4 g, 60 mmol, 3 equiv) in DMF (10 mL) at 0 ˚C was added a solution of benzyl cyanide (2.3 g, 20 mmol, 1 equiv) in DMF (10 mL) dropwise. The resultant mixture was stirred for 15 minutes at the same temperature. A solution of 1,2-dibromoethane (2.6 mL, 30 mmol, 1.5 equiv) in DMF (10 mL) was then added dropwise and the resulting mixture was stirred at the same temperature for an additional 18 hours. The reaction was quenched with H_2O (150 mL) and the aqueous layer was extracted with diethyl ether (3 × 100 mL). The combined organic layer was washed with brine (50 mL), dried over anhydrous $MgSO_4$, filtered, and concentrated under reduced pressure. The residue was purified by flash chromatography eluted with *n*-hexane/diethyl ether (5:1) to yield **S1** as colourless oil.

1-phenylcyclopropanecarbonitrile **S1** was refluxed in aqueous LiOH (4 M) for 18 hours. The reaction mixture was cooled and aqueous HCl (5 M) was added until the pH < 1. The aqueous layer was extracted with ethyl acetate (3 × 20 mL). The combined organic layer was washed with brine (30 mL), dried over anhydrous $MgSO_4$, filtered, and concentrated under reduced pressure. The crude product **S2** was used directly in the next step without further purification.

To a solution of 1-phenylcyclopropanecarboxylic acid **S2** in MeOH (20 mL) was added catalytic amount of H_2SO_4 (0.1 mL) and the resultant mixture was heated at reflux for 18 hours. Upon completion, saturated aqueous $NaHCO_3$ was added until pH = 8. The aqueous layer was extracted with ethyl acetate (3 × 20 mL). The combined organic layer was washed with brine (30 mL), dried over $MgSO_4$, filtered, and concentrated under reduced pressure. The crude product **S3** was used directly in the next step without further purification.

To a solution of mixture of **S2** and **S3** (~3 mmol combined) in diethyl ether (15 mL)

was added the corresponding Grignard reagent (MeMgBr or PhMgBr) (3 M in diethyl ether, 4 mL, 12 mmol, 4 equiv) at 0 ˚C. The mixture was allowed to stir overnight at the same temperature. The reaction was then quenched by saturated aqueous NH_4Cl solution (20 mL) and the aqueous layer was extracted by diethyl ether (3 × 20 mL). The combined organic layer was washed with brine (20 mL), dried over Na_2SO_4, filtered, and concentrated under reduced pressure. The residue was purified by flash chromatography eluted with *n*-hexane/diethyl ether to yield **1t** and **1u**.

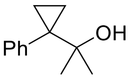

1t

1t: White solid

m.p.: 50–51 °C

R_f = 0.33 (*n*-hexane : diethyl ether = 2 :1)

IR (KBr): 3427 (s), 2361 (w), 1640 (m), 1083 (m)

^{1}H NMR ($CDCl_3$, 500 MHz) : δ 7.40–7.39 (m, 2H), 7.29–7.26 (m, 2H), 7.23–7.20 (m, 1H), 1.20 (s, 6H), 1.03–1.01 (m, 2H), 0.71–0.69 (m, 2H)

^{13}C NMR ($CDCl_3$, 125.7 MHz) : δ 144.1, 132.1, 127.8, 126.7, 71.5, 35.3, 27.9, 9.3

HRMS (ESI) Calc'd for $C_{12}H_{16}O$ $[M+Na]^+$: 199.10934; found 199.10941

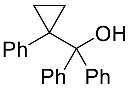

1u

1u: White solid

m.p.: 112–114 °C

R_f = 0.47 (*n*-hexane : diethyl ether = 8 :1)

IR (KBr): 3452 (s), 1637 (m), 701 (w)

^{1}H NMR ($CDCl_3$, 500 MHz) : δ 7.61 (d, *J* = 7.5 Hz, 4H), 7.39 (d, *J* = 7.0 Hz, 2H), 7.30–7.26 (m, 4H), 7.23–7.20 (m, 2H), 7.16–7.10 (m, 3H), 2.46 (s, 1H), 0.98–0.89 (m, 4H)

^{13}C NMR ($CDCl_3$, 125.7 MHz) : δ 144.5, 142.6, 132.8, 128.2, 127.9, 127.6, 127.2, 126.9, 80.8, 33.7, 10.0

HRMS (ESI) Calc'd for $C_{22}H_{20}O$ $[M+Na]^+$: 323.14064; found 323.14064

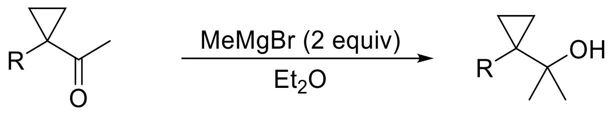

1v, R = H
1w, R = Me

To a solution of the corresponding cyclopropyl methyl ketone (5 mmol) in diethyl ether (20 mL) was added MeMgBr (3 M in diethyl ether, 3.3 mL, 10 mmol, 2 equiv) at 0 ˚C. The resultant mixture was allowed to stir overnight at the same temperature. The reaction was then quenched by saturated aqueous NH_4Cl solution (20 mL) and the aqueous layer was extracted by diethyl ether (3 × 20 mL). The combined organic layer was washed with brine (20 mL), dried over Na_2SO_4, filtered, and *carefully dried with rotavap at ~10 mbar in a 25 °C water bath* due to the volatility of the product. The products **1v** and **1w** were then used directly in the next step without further purification.

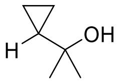

1v

1v: Colourless oil (83% yield, 416 mg)
R_f = 0.50 (*n*-hexane : ethyl acetate = 3 :1)
IR (KBr): 3426 (s), 1642 (m), 1079 (m)
^{1}H NMR ($CDCl_3$, 500 MHz) : δ 1.18 (s, 6H), 0.95 (tt, *J* = 8.5, 5.5 Hz, 1H), 0.39–0.36 (m, 2H), 0.32–0.29 (m, 2H)
^{13}C NMR ($CDCl_3$, 125.7 MHz) : δ 69.9, 28.6, 22.5, 1.1
HRMS (APCI) Calc'd for $C_6H_{12}O$ $[M+H]^+$: 101.09638; found 101.09609

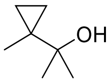

1w

1w: Colourless oil (84% yield, 480 mg)
R_f = 0.57 (*n*-hexane : ethyl acetate = 3 :1)
IR (KBr): 3444 (s), 2360 (w), 1638 (m)
^{1}H NMR ($CDCl_3$, 500 MHz) : δ 1.21 (s, 6H), 1.07 (s, 3H), 0.60 (br, 2H), 0.17 (br, 2H)
^{13}C NMR ($CDCl_3$, 125.7 MHz) : δ 71.7, 27.2, 24.1, 21.6, 10.1
HRMS (ESI) Calc'd for $C_{12}H_{16}O$ $[M+Na+H_2O]^+$: 155.10425; found 155.10423

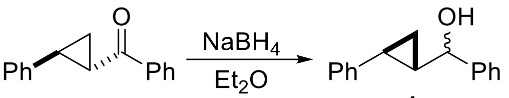

1x

The synthesis of cyclopropyl ketone starting material follows a literature reported procedure.[4]

The cyclopropyl ketone (4.3 mmol, 1 equiv) was dissolved in diethyl ether (20 mL), and $NaBH_4$ (325 mg, 2 equiv) was added at 0 ˚C and stir at room temperature overnight. The reaction was then quenched by saturated aqueous NH_4Cl (20 mL), and the aqueous solution was extracted with ethyl acetate (3 × 20 mL). The combined organic layer was washed with brine (20 mL), dried over Na_2SO_4, filtered, and concentrated under reduced pressure. The residue was purified by flash chromatography eluted with *n*-hexane/ethyl acetate (3:1) to yield the diastereomers **1x** and **1x'** as colourless gel (combined 88% yield, 849 mg)

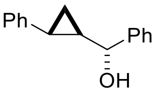

1x

R_f = 0.50 (*n*-hexane : ethyl acetate = 3 :1)

IR (KBr): 3396 (s), 1603 (w), 1495 (w), 698 (w)

^{1}H NMR (CDCl$_3$, 500 MHz) : δ 7.45 (d, *J* = 7.5 Hz, 2H), 7.39 (t, *J* = 7.5 Hz, 2H), 7.34–7.28 (m, 3H), 7.17 (t, *J* = 7.5 Hz, 1H), 7.10 (d, *J* = 7.5 Hz, 2H), 4.31 (d, *J* = 8.0 Hz, 1H), 2.12–2.08 (m, 1H), 1.57 (tt, *J* = 8.0, 5.0 Hz, 1H), 1.09 (dt, *J* = 9.0, 5.5 Hz, 1H), 1.00 (dt, *J* = 9.0, 5.5 Hz, 1H)

^{13}C NMR (CDCl$_3$, 125.7 MHz) : δ 143.5, 142.4, 128.6, 128.5, 127.8, 126.2, 126.0, 125.8, 77.6, 30.8, 22.0, 13.7

HRMS (ESI) Calc'd for $C_{16}H_{16}O$ $[M+Na]^+$: 247.10934; found 247.10933

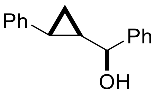

1x'

R_f = 0.40 (*n*-hexane : ethyl acetate = 3 :1)

IR (KBr): 3398 (s), 1600 (w), 1490 (w), 705 (w)

^{1}H NMR (CDCl$_3$, 500 MHz) : δ 7.46–7.44 (m, 2H), 7.38–7.35 (m, 2H), 7.29 (tt, *J* = 7.5, 1.5 Hz, 1H), 7.25–7.22 (m, 2H), 7.15 (tt, *J* = 7.0, 1.5 Hz, 1H), 7.03–7.02 (m, 2H), 4.38 (d, *J* = 7.5 Hz, 1H), 2.07 (br, 1H), 2.03–1.99 (m, 1H), 1.58–1.53 (m, 1H), 1.20 (dt, *J* = 9.0, 5.5 Hz, 1H), 1.07 (dt, *J* = 8.5, 5.0, 1H)

^{13}C NMR (CDCl$_3$, 125.7 MHz) : δ 143.6, 142.2, 128.6, 128.4, 127.8, 126.2, 126.1, 125.8, 76.9, 30.2, 21.1, 13.7

HRMS (ESI) Calc'd for $C_{16}H_{16}O$ [M+Na]$^+$: 247.10934; found 247.10932

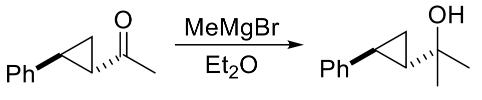

1y

The synthesis of cyclopropyl ketone starting material follows a literature reported procedure.[4]

The cyclopropyl ketone (2.1 mmol, 1 equiv) was dissolved in diethyl ether (10 mL), and MeMgBr (3 M in diethyl ether, 1.4 mL, 4.2 mmol, 2 equiv) was added at 0 ˚C. The resultant mixture was stirred at room temperature overnight. The reaction was then quenched by saturated aqueous NH_4Cl (20 mL), and the aqueous solution was extracted with ethyl acetate (3 × 20 mL). The combined organic layer was washed with brine (20 mL), dried over Na_2SO_4, filtered, and concentrated under reduced pressure. The residue was purified by flash chromatography eluted with *n*-hexane/ethyl acetate (1:1) to yield **1y** as a colourless thick oil. (92% yield, 341 mg)

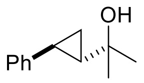

1y

R_f = 0.33 (*n*-hexane : diethyl ether = 1 :1)

IR (KBr): 3442 (s), 1642 (m), 1076 (m)

^{1}H NMR (CDCl$_3$, 500 MHz) : δ 7.27 (t, *J* = 7.5 Hz, 2H), 7.16 (t, *J* = 7.5 Hz, 1H), 7.11 (d, *J* = 7.5, 2H), 1.98–1.94 (m, 1H), 1.43 (br, 1H), 1.31–1.25 (m, 7H), 1.05 (dt, *J* = 9.0, 5.5 Hz, 1H), 0.89–0.85 (m, 1H)

^{13}C NMR (CDCl$_3$, 125.7 MHz) : δ 143.3, 128.4, 126.1, 125.6, 69.6, 34.2, 29.2, 29.1, 19.3, 11.8

HRMS (ESI) Calc'd for $C_{12}H_{16}O$ [M+Na]$^+$: 199.10934; found 199.10953

(C) Procedures for Hydro- and Deuterio-bromination

Note and caution: While the reaction can be conducted in ambient condition, the reaction flask must be placed in the fume cupboard at all time before quenched by saturated $NaHCO_3$ solution. For better reproducibility of results, BBr_3 should be added at last, and the reaction flask had to be placed in a 0 ˚C ice bath when adding BBr_3. The BBr_3 solution (1 M in CH_2Cl_2) was added along the inner wall of the reaction flask in one portion either by a 1 mL or 5 mL autopipette regardless of the scale. The use of apparatus with stainless steel parts (e.g. syringe) is highly discouraged as BBr_3 can dissolve the stainless steel needle quickly, which will affect the reproducibility. It is critical to ensure the reaction mixture is being vigorously stirred.

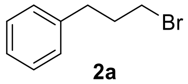

2a

To a solution of CH_2Cl_2 (3.75 mL) in a 25 mL round-bottom flask with a magnetic stirrer bar, **1a** (122 mg, 130 μL, 1 mmol, 1 equiv) and *t*-BuOH (143 μL, 1.5 mmol, 1.5 equiv) were added. The solution was cooled to 0 ˚C and BBr_3 (1 M in CH_2Cl_2, 1.25 mL, 1.25 mmol, 1.25 equiv) was added into the mixture. The resultant mixture was allowed to stir for 1 h at 22 ˚C and then quenched at 0 ˚C by saturated aqueous $NaHCO_3$ solution. The organic layer was separated, and the aqueous layer was extracted by CH_2Cl_2 (3 × 20 mL). The combined organic layer was dried over Na_2SO_4, filtered, and concentrated under reduced pressure. The residue was purified by flash chromatography eluted with *n*-hexane/diethyl ether (50:1) to yield **2a** as colourless oil (77% yield, 153 mg).

R_f = 0.57 (*n*-hexane : diethyl ether = 50 :1)
IR (KBr): 3062 (w), 3026 (m), 2938 (w), 1495 (w), 1435 (w), 1242 (w), 744 (s), 699 (s)
^{1}H NMR ($CDCl_3$, 500 MHz) : δ 7.33–7.30 (m, 2H), 7.24–7.21 (m, 3H), 3.41 (t, J = 7.0 Hz, 2H), 2.80 (t, J = 7.0 Hz, 2H), 2.19 (quint, J = 7.0 Hz)
^{13}C NMR ($CDCl_3$, 125.7 MHz) : δ 140.7, 128.7, 128.6, 126.3, 34.3, 34.1, 33.2
HRMS (EI) Calc'd for $C_9H_{11}Br$ $[M]^+$: 198.0039; found 198.0037
NMR data matches with authentic sample.

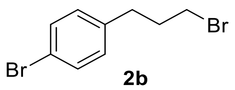

2b

To a solution of CH_2Cl_2 (1.75 mL) in a 4 mL scintillation vial with a magnetic stirrer bar, **1b** (39.4 mg, 0.2 mmol, 1 equiv) and *t*-BuOH (28.5 μL, 0.3 mmol, 1.5 equiv) were added. The solution was cooled to 0 ˚C and BBr_3 (1 M in CH_2Cl_2, 250 μL, 0.25 mmol, 1.25 equiv) was added into the mixture. The resultant mixture was allowed to stir for 25 h at 22 ˚C and then quenched at 0 ˚C by saturated aqueous $NaHCO_3$ solution. The organic layer was separated, and the aqueous layer was extracted by CH_2Cl_2 (3 × 5 mL). The combined organic layer was dried over Na_2SO_4, filtered, and concentrated under reduced pressure. The residue was purified by flash chromatography eluted with *n*-hexane/diethyl ether (40:1) to yield **2b** as light yellow oil (70% yield, 38.9 mg).

R_f = 0.70 (*n*-hexane : diethyl ether = 40 :1)
IR (KBr): 3451 (s), 2069 (w), 1637 (m)
^{1}H NMR ($CDCl_3$, 500 MHz) : δ 7.41 (d, *J* = 8.0 Hz, 2H), 7.08 (d, *J* = 8.0 Hz, 2H), 3.38 (t, *J* = 6.5 Hz, 2H), 2.74 (t, *J* = 7.5 Hz, 2H), 2.14 (quint, *J* = 7.0 Hz, 2H)
^{13}C NMR ($CDCl_3$, 125.7 MHz) : δ 139.6, 131.7, 130.5, 120.1, 34.0, 33.5, 32.9
HRMS (EI) Calc'd for $C_9H_{10}Br_2$ $[M]^+$: 277.9123; found 277.9119

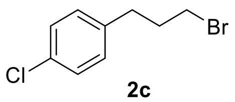

2c

To a solution of CH_2Cl_2 (1.75 mL) in a 4 mL scintillation vial with a magnetic stirrer bar, **1c** (30.5 mg, 0.2 mmol, 1 equiv) and *t*-BuOH (28.5 μL, 0.3 mmol, 1.5 equiv) were added. The solution was cooled to 0 ˚C and BBr_3 (1 M in CH_2Cl_2, 250 μL, 0.25 mmol, 1.25 equiv) was added into the mixture. The resultant mixture was allowed to stir for 17 h at 22 ˚C and then quenched at 0 ˚C by saturated aqueous $NaHCO_3$ solution. The organic layer was separated, and the aqueous layer was extracted by CH_2Cl_2 (3 × 5 mL). The combined organic layer was dried over Na_2SO_4, filtered, and concentrated under reduced pressure. The residue was purified by flash chromatography eluted with *n*-hexane/diethyl ether (50:1) to yield **2c** as colourless oil (54% yield, 25.2 mg).

R_f = 0.53 (*n*-hexane : diethyl ether = 50 :1)
IR (KBr): 3061 (w), 2084 (w), 1644 (m), 1087 (m)
^{1}H NMR ($CDCl_3$, 500 MHz) : δ 7.27 (d, *J* = 8.5 Hz, 2H), 7.13 (d, *J* = 8.5 Hz, 2H), 3.38 (t, *J* = 6.5 Hz, 2H), 2.76 (t, *J* = 7.5 Hz, 2H), 2.14 (quint, *J* = 7.0 Hz, 2H)

^{13}C NMR ($CDCl_3$, 125.7 MHz) : δ 139.1, 132.1, 130.0, 128.8, 34.1, 33.4, 32.9
HRMS (EI) Calc'd for $C_9H_{10}BrCl$ $[M]^+$: 233.9627; found 233.9627

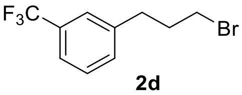

2d

To a solution of CH_2Cl_2 (1.75 mL) in a 4 mL scintillation vial with a magnetic stirrer bar, **1d** (37.2 mg, 0.2 mmol, 1 equiv) and *t*-BuOH (28.5 μL, 0.3 mmol, 1.5 equiv) were added. The solution was cooled to 0 ˚C and BBr_3 (1 M in CH_2Cl_2, 250 μL, 0.25 mmol, 1.25 equiv) was added into the mixture. The mixture was allowed to stir for 44 h at 22 ˚C and then quenched at 0 ˚C by saturated aqueous $NaHCO_3$ solution. The organic layer was separated, and the aqueous layer was extracted by CH_2Cl_2 (3 × 5 mL). The combined organic layer was dried over Na_2SO_4, filtered, and concentrated under reduced pressure. The residue was purified by flash chromatography eluted with *n*-hexane/diethyl ether (40:1) to yield **2d** as colourless oil (53% yield, 28.3 mg).

R_f = 0.60 (*n*-hexane : diethyl ether = 40 :1)
IR (KBr): 3060 (w), 2076 (w), 1637 (m), 1328 (w)
^{1}H NMR ($CDCl_3$, 500 MHz) : δ 7.48–7.45 (m, 2H), 7.43–7.39 (m, 2H), 3.40 (t, *J* = 6.5 Hz, 2H), 2.85 (t, *J* = 7.5 Hz, 2H), 2.19 (quint, *J* = 7.0 Hz, 2H)
^{13}C NMR ($CDCl_3$, 125.7 MHz) : δ 141.6, 132.1, 131.0 (q, *J* = 32.2 Hz), 129.1, 125.3 (q, *J* = 3.9 Hz), 124.3 (q, *J* = 272.4 Hz), (Two of the peaks are overlapped, only the peak at 127.5 and 121.0 ppm are observed), 123.3 (q, *J* = 3.6 Hz), 34.0, 33.9, 32.8
^{19}F NMR ($CDCl_3$, 470.6 MHz) : δ –62.6 (s)
HRMS (APCI/DIP) Calc'd for $C_{10}H_{10}BrF_3$ $[M+H]^+$: 265.99125; found 265.99113

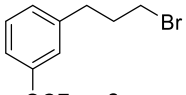

2e

To a solution of CH_2Cl_2 (1.75 mL) in a 4 mL scintillation vial with a magnetic stirrer bar, **1e** (40.4 mg, 0.2 mmol, 1 equiv) and *t*-BuOH (28.5 μL, 0.3 mmol, 1.5 equiv) were added. The solution was cooled to 0 ˚C and BBr_3 (1 M in CH_2Cl_2, 250 μL, 0.25 mmol, 1.25 equiv) was added into the mixture. The resultant mixture was allowed to stir for 40 h at 22 ˚C and then quenched at 0 ˚C by saturated aqueous $NaHCO_3$ solution. The organic layer was separated, and the aqueous layer was extracted by CH_2Cl_2 (3 × 5 mL). The combined organic layer was dried over Na_2SO_4, filtered, and concentrated under reduced pressure. The residue was purified by flash chromatography eluted with *n*-hexane/diethyl ether (40:1) to yield **2e** as colourless oil (69% yield, 39.1 mg).

R_f = 0.43 (*n*-hexane : diethyl ether = 40 :1)

IR (KBr): 3062 (w), 2084 (w), 1643 (m), 1259 (m), 1161 (w), 1083 (w)

^{1}H NMR ($CDCl_3$, 500 MHz) : δ 7.32 (t, *J* = 7.5 Hz, 1H), 7.14 (d, *J* = 7.5 Hz, 1H), 7.08–7.06 (m, 2H), 3.39 (t, *J* = 6.5 Hz, 2H), 2.81 (t, *J* = 7.5 Hz, 2H), 2.17 (quint, *J* = 7.0 Hz, 2H)

^{13}C NMR ($CDCl_3$, 125.7 MHz) : δ 149.6, 143.0, 129.9, 127.1, 121.2, 120.6 (q, *J* = 256.9 Hz), 118.8, 33.9, 33.8, 32.8

^{19}F NMR ($CDCl_3$, 470.6 MHz) : δ –57.7 (s)

HRMS (EI) Calc'd for $C_{10}H_{10}BrF_3O$ $[M]^+$: 281.9862; found 281.98602

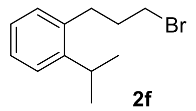

2f

To a solution of CH_2Cl_2 (1.75 mL) in a 4 mL scintillation vial with a magnetic stirrer bar, **1f** (32.1 mg, 0.2 mmol, 1 equiv) and *t*-BuOH (28.5 μL, 0.3 mmol, 1.5 equiv) were added. The solution was cooled to 0 ˚C and BBr_3 (1 M in CH_2Cl_2, 250 μL, 0.25 mmol, 1.25 equiv) was added into the mixture. The resultant mixture was allowed to stir for 15 h at 22 ˚C and then quenched at 0 ˚C by saturated aqueous $NaHCO_3$ solution. The organic layer was separated, and the aqueous layer was extracted by CH_2Cl_2 (3 × 5 mL). The combined organic layer was dried over Na_2SO_4, filtered, and concentrated under reduced pressure to yield **2f** as yellow oil (83% yield, 40.0 mg).

R_f = 0.70 (*n*-hexane : diethyl ether = 40 :1)

IR (KBr): 3060 (w), 2962 (s), 1634 (m), 1459 (m), 758 (m), 562 (w)

^{1}H NMR ($CDCl_3$, 500 MHz) : δ 7.31–7.30 (m, 1H), 7.25–7.21 (m, 1H), 7.17–7.12 (m, 2H), 3.47 (t, *J* = 6.5 Hz, 2H), 3.20 (septet, *J* = 7.0 Hz, 1H), 2.84 (t, *J* = 7.5 Hz, 2H), 2.15 (quint, *J* = 7.5 Hz, 2H), 1.27 (d, *J* = 7.0 Hz, 6H)

^{13}C NMR ($CDCl_3$, 125.7 MHz) : δ 146.8, 137.4, 129.6, 126.8, 125.8, 125.6, 34.5, 33.6, 31.2, 28.7, 24.2

HRMS (EI) Calc'd for $C_{12}H_{17}Br$ $[M]^+$: 240.0508; found 240.0505

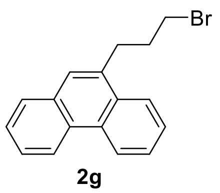

2g

To a solution of CH_2Cl_2 (0.75 mL) in a 4 mL scintillation vial with a magnetic stirrer bar, **1g** (43.7 mg, 0.2 mmol, 1 equiv) and H_2O (5.4 μL, 0.3 mmol, 1.5 equiv) were

added. The solution was cooled to 0 ˚C and BBr_3 (1 M in CH_2Cl_2, 250 μL, 0.25 mmol, 1.25 equiv) was added into the mixture. The resultant mixture was allowed to stir for 12 h at 22 ˚C and then quenched at 0 ˚C by saturated aqueous $NaHCO_3$ solution. The organic layer was separated, and the aqueous layer was extracted by CH_2Cl_2 (3 × 5 mL). The combined organic layer was dried over Na_2SO_4, filtered, and concentrated under reduced pressure to yield **2g** as a brown solid (quantitative yield, 59.8 mg).

m.p.: 72–74 °C
R_f = 0.60 (*n*-hexane : diethyl ether = 40 :1)
IR (KBr): 3060 (w), 2084 (w), 1643 (m), 1079 (m)
^{1}H NMR ($CDCl_3$, 500 MHz) : δ 8.38 (s, 1H), 8.31 (d, *J* = 9.0 Hz, 2H), 8.03 (d, *J* = 8.5 Hz, 2H), 7.57–7.54 (m, 2H), 7.51–7.48 (m, 2H), 3.80 (t, *J* = 8.0 Hz, 2H), 3.62 (t, *J* = 6.5 Hz, 2H), 2.42–2.36 (m, 2H)
^{13}C NMR ($CDCl_3$, 125.7 MHz) : δ 133.1, 131.7, 129.8, 129.4, 126.3, 125.9, 125.0, 124.2, 34.2, 34.0, 26.5
HRMS (APCI/DIP) Calc'd for $C_{17}H_{15}Br$ $[M+H]^+$: 299.04299; found 299.04306

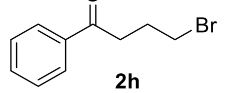

2h

To a solution of CH_2Cl_2 (3.75 mL) in a 25 mL round-bottom flask with a magnetic stirrer bar, **1h** (146.1 mg, 1.0 mmol, 1 equiv) and *t*-BuOH (143 μL, 1.5 mmol, 1.5 equiv) were added. The solution was cooled to 0 ˚C and BBr_3 (1 M in CH_2Cl_2, 1.25 mL, 1.25 mmol, 1.25 equiv) was added into the mixture. The resultant mixture was allowed to stir for 18 h at 22 ˚C and then quenched at 0 ˚C by saturated aqueous $NaHCO_3$ solution. The organic layer was separated, and the aqueous layer was extracted by CH_2Cl_2 (3 × 20 mL). The combined organic layer was dried over Na_2SO_4, filtered, and concentrated under reduced pressure. The residue was purified by flash chromatography eluted with *n*-hexane/diethyl ether (8:1) to yield **2h** as yellow oil (90% yield, 204 mg)

R_f = 0.37 (*n*-hexane : diethyl ether = 8 :1)
IR (KBr): 3060 (w), 2084 (m), 1644 (m), 1225 (w), 1077 (m)
^{1}H NMR ($CDCl_3$, 500 MHz) : δ 7.99–7.97 (m, 2H), 7.57 (tt, *J* = 7.5, 1.5 Hz, 1H), 7.47 (t, *J* = 7.5 Hz, 2H), 3.55 (t, *J* = 6.5 Hz, 2H), 3.19 (t, *J* = 7.0 Hz, 2H), 2.31 (quint, *J* = 6.5 Hz, 2H)
^{13}C NMR ($CDCl_3$, 125.7 MHz) : δ 199.0, 136.8, 133.4, 128.8, 128.1, 36.7, 33.8, 27.0
HRMS (APCI/DIP) Calc'd for $C_{10}H_{11}BrO$ $[M+H]^+$: 227.00660; found 227.00668

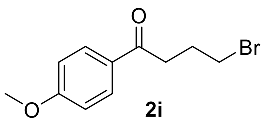

To a solution of CH_2Cl_2 (1.75 mL) in a 4 mL scintillation vial with a magnetic stirrer bar, **1i** (35.2 mg, 0.2 mmol, 1 equiv) and *t*-BuOH (28.5 µL, 0.3 mmol, 1.5 equiv) were added. The solution was cooled to 0 ˚C and BBr_3 (1 M in CH_2Cl_2, 250 µL, 0.25 mmol, 1.25 equiv) was added into the mixture. The mixture was allowed to stir for 15 h at 22 ˚C and then quenched at 0 ˚C by saturated aqueous $NaHCO_3$ solution. The organic layer was separated, and the aqueous layer was extracted by CH_2Cl_2 (3 × 5 mL). The combined organic layer was dried over Na_2SO_4, filtered, and concentrated under reduced pressure. The residue was purified by flash chromatography eluted with *n*-hexane/diethyl ether (8:1) to yield **2i** as yellow oil (81% yield, 41.7 mg).

R_f = 0.27 (*n*-hexane : diethyl ether = 8 :1)
IR (KBr): 3060 (w), 2092 (w), 1643 (m), 1078 (w)
^{1}H NMR ($CDCl_3$, 500 MHz) : δ 7.97 (dt, *J* = 9.5, 2.5 Hz, 2H), 6.96–6.93 (m, 2H), 3.88 (s, 3H), 3.55 (t, *J* = 6.5 Hz, 2H), 3.13 (t, *J* = 7.0 Hz, 2H), 2.30 (quint, *J* = 6.5 Hz, 2H)
^{13}C NMR ($CDCl_3$, 125.7 MHz) : δ 197.5, 163.7, 130.5, 130.0, 113.9, 55.6, 36.3, 33.9, 27.2
HRMS (APCI/DIP) Calc'd for $C_{11}H_{13}BrO_2$ $[M+H]^+$: 257.01717; found 257.01712

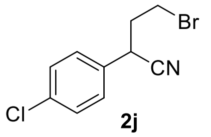

To a solution of CH_2Cl_2 (0.75 mL) in a 4 mL scintillation vial with a magnetic stirrer bar, **1j** (35.5 mg, 0.2 mmol, 1 equiv) and H_2O (5.4 µL, 0.3 mmol, 1.5 equiv) were added. The solution was cooled to 0 ˚C and BBr_3 (1 M in CH_2Cl_2, 250 µL, 0.25 mmol, 1.25 equiv) was added into the mixture. The mixture was allowed to stir for 18 h at 22 ˚C and then quenched at 0 ˚C by saturated $NaHCO_3$ solution. The organic layer was separated, and the aqueous layer was extracted by CH_2Cl_2 (3 × 5 mL). The combined organic layer was dried over Na_2SO_4, filtered, and concentrated under reduced pressure to yield **2j** as yellow oil (quantitative yield, 51.9 mg).

R_f = 0.33 (*n*-hexane : diethyl ether = 3 :1)
IR (KBr): 3060 (w), 2243 (w), 1643 (w), 1492 (m), 1249 (m), 1093 (m), 823 (m)
^{1}H NMR ($CDCl_3$, 500 MHz) : δ 7.38 (dt, *J* = 9.0, 2.5 Hz, 2H), 7.31 (dt, *J* = 9.0, 2.5 Hz, 2H), 4.12 (t, *J* = 7.5 Hz, 1H), 3.54 (ddd, *J* = 10.5, 8.0, 5.0 Hz, 1H), 3.35 (ddd, *J* =

10.5, 6.5, 5.0 Hz, 1H), 2.51–2.44 (m, 1H), 2.34–2.27 (m, 1H)
^{13}C NMR ($CDCl_3$, 125.7 MHz) : δ 134.7, 132.8, 129.7, 128.9, 119.6, 38.2, 35.1, 29.2
HRMS (APCI/DIP) Calc'd for $C_{10}H_9BrClN$ $[M+H]^+$: 259.96573; found 259.96577

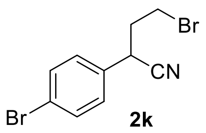
2k

To a solution of CH_2Cl_2 (0.75 mL) in a 4 mL scintillation vial with a magnetic stirrer bar, **1k** (44.4 mg, 0.2 mmol, 1 equiv) and H_2O (5.4 µL, 0.3 mmol, 1.5 equiv) were added. The solution was cooled to 0 ˚C and BBr_3 (1 M in CH_2Cl_2, 250 µL, 0.25 mmol, 1.25 equiv) was added into the mixture. The mixture was allowed to stir for 18 h at 22 ˚C and then quenched at 0 ˚C by saturated aqueous $NaHCO_3$ solution. The organic layer was separated, and the aqueous layer was extracted by CH_2Cl_2 (3 × 5 mL). The combined organic layer was dried over Na_2SO_4, filtered, and concentrated under reduced pressure to yield **2k** as colourless oil (quantitative yield, 60.6 mg).

R_f = 0.33 (*n*-hexane : diethyl ether = 3 :1)
IR (KBr): 3056 (w), 2243 (w), 1642 (m), 1488 (w), 1073 (m), 817 (w)
^{1}H NMR ($CDCl_3$, 500 MHz) : δ 7.54 (dt, *J* = 9.0, 2.5 Hz, 2H), 7.25 (dt, *J* = 9.0, 2.0 Hz, 2H), 4.11 (t, *J* = 7.5 Hz, 1H), 3.54 (ddd, *J* = 10.5, 7.5, 5.0 Hz, 1H), 3.35 (ddd, *J* = 10.5, 7.0, 5.0 Hz, 1H), 2.50–2.44 (m, 1H), 2.34–2.27 (m, 1H)
^{13}C NMR ($CDCl_3$, 125.7 MHz) : δ 133.3, 132.6, 129.2, 122.8, 119.5, 38.1, 35.2, 29.2
HRMS (APCI/DIP) Calc'd for $C_{10}H_9Br_2N$ $[M+H]^+$: 303.91543; found 303.91536

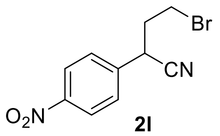
2l

To a solution of CH_2Cl_2 (0.75 mL) in a 4 mL scintillation vial with a magnetic stirrer bar, **1l** (37.6 mg, 0.2 mmol, 1 equiv) and H_2O (5.4 µL, 0.3 mmol, 1.5 equiv) were added. The solution was cooled to 0 ˚C and BBr_3 (1 M in CH_2Cl_2, 250 µL, 0.25 mmol, 1.25 equiv) was added into the mixture. The mixture was allowed to stir for 44 h at 22 ˚C and then quenched at 0 ˚C by saturated aqueous $NaHCO_3$ solution. The organic layer was separated, and the aqueous layer was extracted by CH_2Cl_2 (3 × 5 mL). The combined organic layer was dried over Na_2SO_4, filtered, and concentrated under reduced pressure. The residue was purified by flash chromatography eluted with *n*-hexane/ethyl acetate (3:1) to yield **2l** as light yellow thick oil (83% yield, 44.7 mg).

R_f = 0.37 (*n*-hexane : ethyl acetate = 3 :1)
IR (KBr): 3059 (w), 1643 (w), 1519 (m), 1344 (m), 849 (w), 695 (w)
^{1}H NMR (CDCl$_3$, 500 MHz) : δ 8.28 (dt, *J* = 9.5, 2.5 Hz, 2H), 7.59 (dt, *J* = 9.0, 2.5 Hz, 2H), 4.3 (dd, *J* = 8.5, 7.0 Hz, 1H), 3.59 (ddd, *J* = 10.5, 8.5, 5.0 Hz, 1H), 3.42–3.38 (m, 1H), 2.57–2.50 (m, 1H), 2.39–2.32 (m, 1H)
^{13}C NMR (CDCl$_3$, 125.7 MHz) : δ 148.1, 141.4, 128.7, 124.7, 118.7, 38.1, 35.6, 29.0
HRMS (APCI/DIP) Calc'd for $C_{10}H_9BrN_2O_2$ $[M+H]^+$: 268.99202; found 268.99220

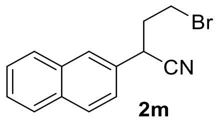

2m

To a solution of CH_2Cl_2 (0.75 mL) in a 4 mL scintillation vial with a magnetic stirrer bar, **1m** (38.7 mg, 0.2 mmol, 1 equiv) and H_2O (5.4 μL, 0.3 mmol, 1.5 equiv) were added. The solution was cooled to 0 ˚C and BBr_3 (1 M in CH_2Cl_2, 250 μL, 0.25 mmol, 1.25 equiv) was added into the mixture. The mixture was allowed to stir for 17 h at 22 ˚C and then quenched at 0 ˚C by saturated aqueous $NaHCO_3$ solution. The organic layer was separated, and the aqueous layer was extracted by CH_2Cl_2 (3 × 5 mL). The combined organic layer was dried over Na_2SO_4, filtered, and concentrated under reduced pressure to yield **2m** as yellow oil (quantitative yield, 54.9 mg).

R_f = 0.33 (*n*-hexane : diethyl ether = 3 :1)
IR (KBr): 3057 (w), 2241 (w), 1508 (w), 1432 (w), 818 (m), 749 (m), 478 (m)
^{1}H NMR (CDCl$_3$, 500 MHz) : δ 7.90–7.85 (m, 4H), 7.57–7.52 (m, 2H), 7.43 (dd, *J* = 8.5, 1.5 Hz, 1H), 4.30 (t, *J* = 7.5 Hz, 1H), 3.57 (ddd, *J* = 10.5, 8.0, 5.5 Hz, 1H), 3.38 (ddd, *J* = 10.5, 6.5, 5.5 Hz, 1H), 2.60–2.53 (m, 1H), 2.47–2.40 (m, 1H)
^{13}C NMR (CDCl$_3$, 125.7 MHz) : δ 133.3, 133.1, 131.5, 129.5, 128.0, 127.9, 127.0, 126.9, 126.8, 124.7, 120.0, 38.2, 35.8, 29.5
HRMS (APCI/DIP) Calc'd for $C_{14}H_{12}BrN$ $[M+H]^+$: 274.02259; found 274.02246

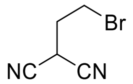

2n

To a solution of CH_2Cl_2 (1.75 mL) in a 4 mL scintillation vial with a magnetic stirrer bar, **1n** (18.4 mg, 0.2 mmol, 1 equiv) and *t*-BuOH (28.5 μL, 0.3 mmol, 1.5 equiv) were added. The solution was cooled to 0 ˚C and BBr_3 (1 M in CH_2Cl_2, 250 μL, 0.25 mmol, 1.25 equiv) was added into the mixture in one portion by autopipette. The mixture was allowed to stir for 65 h at 22 ˚C and then quenched at 0 ˚C by saturated aqueous $NaHCO_3$ solution. The organic layer was separated, and the aqueous layer

was extracted by CH_2Cl_2 (3 × 5 mL). The combined organic layer was dried over Na_2SO_4, filtered, and concentrated under reduced pressure. The residual solid was washed with *n*-hexane (3 × 5 mL) and dried under vacuum to yield **2n** as light yellow solid (65% yield, 22.5 mg).

m.p.: 83–85 °C
IR (KBr): 3060 (w), 1638 (w)
^{1}H NMR (CDCl$_3$, 500 MHz) : δ 4.16 (t, *J* = 7.5 Hz, 1H), 3.61 (t, *J* = 6.0 Hz, 2H), 2.57 (dt, *J* = 7.5, 6.0 Hz, 2H)
^{13}C NMR (CDCl$_3$, 125.7 MHz) : δ 111.7, 33.5, 27.2, 21.7
HRMS (EI) Calc'd for $C_5H_5BrN_2$ $[M]^+$: 171.9631; found 171.96311

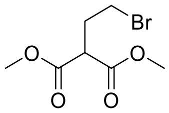

2o

To a solution of CH_2Cl_2 (8.75 mL) in a 25 mL round-bottom flask with a magnetic stirrer bar, **1o** (158.2 mg, 1.0 mmol, 1 equiv) and *t*-BuOH (143 µL, 1.5 mmol, 1.5 equiv) were added. The solution was cooled to 0 ˚C and BBr_3 (1 M in CH_2Cl_2, 1.25 mL, 1.25 mmol, 1.25 equiv) was added into the mixture. The resultant mixture was allowed to stir for 18 h at 22 ˚C and then quenched at 0 ˚C by saturated $NaHCO_3$ solution. The organic layer was separated, and the aqueous layer was extracted by CH_2Cl_2 (3 × 20 mL). The combined organic layer was dried over Na_2SO_4, filtered, and concentrated under reduced pressure to yield **2o** as light yellow oil (quantitative yield, 239.0 mg).

R_f = 0.53 (*n*-hexane : diethyl ether = 1 :1)
IR (KBr): 3058 (w), 1734 (m), 1646 (m), 1216 (w), 1086 (m)
^{1}H NMR (CDCl$_3$, 500 MHz) : δ 3.75 (s, 6H), 3.69 (t, *J* = 7.5 Hz, 1H), 3.44 (t, *J* = 6.5 Hz, 2H), 2.43 (q, *J* = 6.5 Hz, 2H)
^{13}C NMR (CDCl$_3$, 125.7 MHz) : δ 169.1, 52.9, 49.9, 31.6, 30.4
HRMS (ESI) Calc'd for $C_7H_{11}BrO_4$ $[M+Na]^+$: 260.97329; found 260.97329

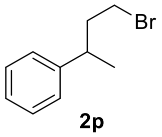

2p

To a solution of CH_2Cl_2 (1.75 mL) in a 4 mL scintillation vial with a magnetic stirrer bar, **1p** (26.4 mg, 0.2 mmol, 1 equiv) and *t*-BuOH (28.5 µL, 0.3 mmol, 1.5 equiv) were added. The solution was cooled to 0 ˚C and BBr_3 (1 M in CH_2Cl_2, 250 µL, 0.25

mmol, 1.25 equiv) was added into the mixture. The resultant mixture was allowed to stir for 1 h at 22 ˚C and then quenched at 0 ˚C by saturated aqueous $NaHCO_3$ solution. The organic layer was separated, and the aqueous layer was extracted by CH_2Cl_2 (3 × 5 mL). The combined organic layer was dried over Na_2SO_4, filtered, and concentrated under reduced pressure. The residue was purified by flash chromatography eluted with *n*-hexane/diethyl ether (40:1) to yield **2p** as colourless oil (52% yield, 22.2 mg).

R_f = 0.70 (*n*-hexane: diethyl ether = 40 :1)
IR (KBr): 3060 (w), 1641 (m), 1083 (m), 762 (w), 700 (w)
^{1}H NMR ($CDCl_3$, 500 MHz) : δ 7.33–7.31 (m, 2H), 7.24–7.21 (m, 3H), 3.35–3.31 (m, 1H), 3.23–3.18 (m, 1H), 2.97 (sextet, *J* = 7.0 Hz, 1H), 2.13 (q, *J* = 7.0 Hz, 2H), 1.3 (d, *J* = 7.0 Hz, 3H)
^{13}C NMR ($CDCl_3$, 125.7 MHz) : δ 145.6, 128.7, 127.2, 126.5, 41.2, 38.4, 32.3, 21.9
HRMS (EI) Calc'd for $C_{10}H_{13}Br$ $[M]^+$: 212.0195; found 212.0191

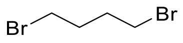

2q

To a solution of CH_2Cl_2 (1.75 mL) in a 4 mL scintillation vial with a magnetic stirrer bar, **1q** (27.0 mg, 0.2 mmol, 1 equiv) and H_2O (5.4 µL, 0.3 mmol, 1.5 equiv) were added. The solution was cooled to 0 ˚C and BBr_3 (1 M in CH_2Cl_2, 250 µL, 0.25 mmol, 1.25 equiv) was added into the mixture. The resultant mixture was allowed to stir for 23 h at 22 ˚C. Due to the low boiling point of **2q**, CH_2Br_2 (10 µL, 0.143 mmol) was added as internal standard, and 100 µL of reaction mixture was extracted and added to 400 µL of CD_2Cl_2 to perform the ^{1}H crude NMR analysis. By comparing with an authentic sample of **2q** in CD_2Cl_2, the yield of **2q** was determined to be 87%.

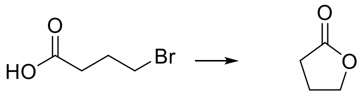

2r **γ-butyrolactone**

To a solution of CH_2Cl_2 (7.5 mL) in a 25 mL round-bottom flask with a magnetic stirrer bar, **1r** (172 mg, 2 mmol, 1 equiv) and H_2O (54 µL, 3 mmol, 1.5 equiv) were added. The solution was cooled to 0 ˚C and BBr_3 (1 M in CH_2Cl_2, 2.5 mL, 2.5 mmol, 1.25 equiv) was added into the mixture. The resultant mixture was allowed to stir for 46 h at 22 ˚C. Product **2r** was formed as indicated by HMRS analysis on the crude mixture. The reaction was then quenched at 0 ˚C by saturated aqueous $NaHCO_3$ solution. The organic layer was separated, and the aqueous layer was extracted by ethyl acetate (3 × 20 mL). The combined organic layer was dried over Na_2SO_4, filtered, and concentrated under reduced pressure to yield γ-butylrolactone as a yellow oil (67% yield, 115 mg).

IR (KBr): 3057 (w), 1760 (s), 1641 (m), 1186 (s), 1037 (m)
^{1}H NMR (CDCl$_3$, 500 MHz) : δ 4.28 (t, *J* = 7.5 Hz, 2H), 2.42 (t, *J* = 8.5 Hz, 2H), 2.20 (quint, *J* = 7.5 Hz, 2H)
^{13}C NMR (CDCl$_3$, 125.7 MHz) : δ 177.9, 68.6, 27.7, 22.1
HRMS (APCI/DIP) Calc'd for $C_4H_6O_2$ $[M+H]^+$: 87.04406; found 87.04395

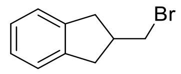

2s

To a solution of CH_2Cl_2 (0.75 mL) in a 4 mL scintillation vial with a magnetic stirrer bar, **1s** (26.0 mg, 0.2 mmol, 1 equiv) and H_2O (5.4 µL, 0.3 mmol, 1.5 equiv) were added. The solution was cooled to 0 ˚C and BBr_3 (1 M in CH_2Cl_2, 250 µL, 0.25 mmol, 1.25 equiv) was added into the mixture. The resultant mixture was allowed to stir for 15 h at 22 ˚C and then quenched at 0 ˚C by saturated aqueous $NaHCO_3$ solution. The organic layer was separated, and the aqueous layer was extracted by CH_2Cl_2 (3 × 5 mL). The combined organic layer was dried over Na_2SO_4, filtered, and concentrated under reduced pressure to yield **2s** as yellow oil (30% yield, 12.7 mg).

R_f = 0.50 (*n*-hexane : diethyl ether = 20 :1)
^{1}H NMR (CDCl$_3$, 500 MHz) : δ 7.21–7.19 (m, 2H), 7.17–7.14 (m, 2H), 3.52 (d, *J* = 7.0 Hz, 2H), 3.15 (dd, *J* = 15.0, 7.5 Hz, 2H), 2.96–2.87 (m, 1H), 2.81 (dd, *J* = 16.0, 7.0 Hz, 2H)
^{13}C NMR (CDCl$_3$, 125.7 MHz) : δ 142.2, 126.6, 124.8, 42.1, 38.5, 38.4
Data matches with literature reported values (Y. Bekkali, et al. *Bioorg. Med. Chem. Lett.* 2007, **17**, 2465)

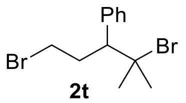

2t

To a solution of CH_2Cl_2 (0.75 mL) in a 4 mL scintillation vial with a magnetic stirrer bar, **1t** (35.5 mg, 0.2 mmol, 1 equiv) and H_2O (5.4 µL, 0.3 mmol, 1.5 equiv) were added. The solution was cooled to 0 ˚C and BBr_3 (1 M in CH_2Cl_2, 250 µL, 0.25 mmol, 1.25 equiv) was added into the mixture. The resultant mixture was allowed to stir for 17 h at 22 ˚C and then quenched at 0 ˚C by saturated aqueous $NaHCO_3$ solution. The organic layer was separated, and the aqueous layer was extracted by CH_2Cl_2 (3 × 5 mL). The combined organic layer was dried over Na_2SO_4, filtered, and concentrated under reduced pressure to yield **2t** as colourless oil (81% yield, 51.7 mg).

R_f = 0.63 (*n*-hexane : diethyl ether = 20 :1)
IR (KBr): 3059 (w), 2969 (w), 1641 (m), 1453 (w), 1102 (w), 702 (m)
^{1}H NMR (CDCl$_3$, 500 MHz) : δ 7.35–7.28 (m, 5H), 3.36–3.32 (m, 1H), 3.05–2.97 (m, 2H), 2.67–2.60 (m, 1H), 2.52–2.45 (m, 1H), 1.79 (s, 3H), 1.65 (s, 3H)
^{13}C NMR (CDCl$_3$, 125.7 MHz) : δ 138.6, 129.7, 128.5, 127.7, 70.7, 57.2, 35.4, 34.7, 32.3, 32.2
**Difficulties with obtaining HRMS for this compound were addressed below.*

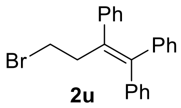

2u

To a solution of CH_2Cl_2 (0.75 mL) in a 4 mL scintillation vial with a magnetic stirrer bar, **1u** (60.1 mg, 0.2 mmol, 1 equiv) and H_2O (5.4 μL, 0.3 mmol, 1.5 equiv) were added. The solution was cooled to 0 ˚C and BBr_3 (1 M in CH_2Cl_2, 250 μL, 0.25 mmol, 1.25 equiv) was added into the mixture. The resultant mixture was allowed to stir for 17 h at 22 ˚C and then quenched at 0 ˚C by saturated aqueous $NaHCO_3$ solution. The organic layer was separated, and the aqueous layer was extracted by CH_2Cl_2 (3 × 5 mL). The combined organic layer was dried over Na_2SO_4, filtered, and concentrated under reduced pressure to yield **2u** as a light yellow solid (quantitative yield, 75.3 mg).

R_f = 0.60 (*n*-hexane : diethyl ether = 40 :1)
m.p.: 96–98 °C
IR (KBr): 3059 (w), 2085 (w), 1644 (m), 1442 (w), 1076 (m), 698 (w)
^{1}H NMR (CDCl$_3$, 500 MHz) : δ 7.42–7.31 (m, 5H), 7.23–7.16 (m, 5H), 7.08–7.02 (m, 3H), 6.96–6.94 (m, 2H), 3.33 (t, *J* = 7.5 Hz, 2H), 3.09 (t, *J* = 7.5 Hz, 2H)
^{13}C NMR (CDCl$_3$, 125.7 MHz) : δ 142.7, 142.4, 142.3, 140.6, 137.0, 130.5, 129.7, 129.4, 128.5, 128.3, 127.6, 127.2, 126.8, 126.3, 38.7, 31.3
HRMS (APCI/DIP) Calc'd for $C_{22}H_{19}Br$ $[M+H]^+$: 365.07249; found 365.07233

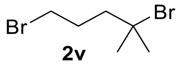

2v

To a solution of CH_2Cl_2 (0.75 mL) in a 4 mL scintillation vial with a magnetic stirrer bar, **1v** (20.0 mg, 0.2 mmol, 1 equiv) and H_2O (5.4 μL, 0.3 mmol, 1.5 equiv) were added. The solution was cooled to 0 ˚C and BBr_3 (1 M in CH_2Cl_2, 250 μL, 0.25 mmol, 1.25 equiv) was added into the mixture. The resultant mixture was allowed to stir for 18 h at 22 ˚C and then quenched at 0 ˚C by saturated aqueous $NaHCO_3$ solution. The

organic layer was separated, and the aqueous layer was extracted by CH_2Cl_2 (3 × 5 mL). The combined organic layer was dried over Na_2SO_4, filtered, and concentrated under reduced pressure carefully (~200 mbar, 25 °C water bath) to yield **2v** as light yellow oil (84% yield, 41.0 mg).

R_f = 0.60 (*n*-hexane : diethyl ether = 10 :1)
IR (KBr): 3059 (w), 2085 (w), 1637 (m)
^{1}H NMR (CDCl$_3$, 500 MHz) : δ 3.45 (t, *J* = 6.5 Hz, 2H), 2.15–2.09 (m, 2H), 1.94–1.90 (m, 2H), 1.77 (s, 6H)
^{13}C NMR (CDCl$_3$, 125.7 MHz) : δ 66.8, 46.0, 34.4, 33.6, 29.9
**Difficulties with obtaining HRMS for this compound were addressed below.*

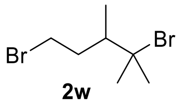

2w

To a solution of CH_2Cl_2 (0.75 mL) in a 4 mL scintillation vial with a magnetic stirrer bar, **1w** (22.8 mg, 0.2 mmol, 1 equiv) and H_2O (5.4 μL, 0.3 mmol, 1.5 equiv) were added. The solution was cooled to 0 ˚C and BBr_3 (1 M in CH_2Cl_2, 250 μL, 0.25 mmol, 1.25 equiv) was added into the mixture. The resultant mixture was allowed to stir for 18 h at 22 ˚C and then quenched at 0 ˚C by saturated aqueous $NaHCO_3$ solution. The organic layer was separated, and the aqueous layer was extracted by CH_2Cl_2 (3 × 5 mL). The combined organic layer was dried over Na_2SO_4, filtered, and concentrated under reduced pressure carefully (~200 mbar, 25 °C water bath) to yield **2w** as light yellow oil (82% yield, 42.3 mg).

R_f = 0.63 (*n*-hexane : diethyl ether = 10 :1)
IR (KBr): 3057 (w), 2091 (w), 1640 (m), 1079 (m)
^{1}H NMR (CDCl$_3$, 500 MHz) : δ 3.60–3.56 (m, 1H), 3.42–3.36 (m, 1H), 2.33–2.28 (m, 1H), 1.79 (s, 3H), 1.75–1.72 (m, 5H), 1.05 (d, *J* = 6.0 Hz, 3H)
^{13}C NMR (CDCl$_3$, 125.7 MHz) : δ 73.0, 44.9, 36.4, 32.9, 32.4, 32.3, 15.3
**Difficulties with obtaining HRMS for this compound were addressed below.*

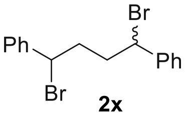

2x

To a solution of CH_2Cl_2 (0.75 mL) in a 4 mL scintillation vial with a magnetic stirrer bar, **1x** or **1x’** (44.9 mg, 0.2 mmol, 1 equiv) and H_2O (5.4 μL, 0.3 mmol, 1.5 equiv)

were added. The solution was cooled to 0 ˚C and BBr_3 (1 M in CH_2Cl_2, 250 µL, 0.25 mmol, 1.25 equiv) was added into the mixture. The resultant mixture was allowed to stir for 15 h at 22 ˚C and then quenched at 0 ˚C by saturated aqueous $NaHCO_3$ solution. The organic layer was separated, and the aqueous layer was extracted by CH_2Cl_2 (3 × 5 mL). The combined organic layer was dried over Na_2SO_4, filtered, and concentrated under reduced pressure. The residue was purified by flash chromatography eluted with *n*-hexane/diethyl ether (40:1) to yield **2x** as a mixture of diastereomers (1:0.8, 63% yield, 46.7 mg). Recrystallization from $CHCl_3$/hexane afforded the major diastereomer **2x** as a colourless crystal.

R_f = 0.33 (*n*-hexane : diethyl ether = 40 :1)
m.p.: 120-121 °C
IR (KBr): 3059 (w), 2360 (w), 1636 (m), 759 (w)
^{1}H NMR ($CDCl_3$, 500 MHz) : δ 7.39–7.34 (m, 8H), 7.31–7.29 (m, 2H), 4.96 (t, *J* = 5.0 Hz, 2H), 2.45–2.38 (m, 2H), 2.32–2.26 (m, 2H)
^{13}C NMR ($CDCl_3$, 125.7 MHz) : δ 141.7, 129.0, 128.7, 127.3, 54.4, 38.8
HRMS (EI) Calc'd for $C_{16}H_{16}Br_2$ $[M]^+$: 367.9594; found 367.95902

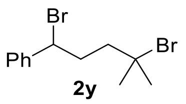

2y

To a solution of CH_2Cl_2 (0.75 mL) in a 4 mL scintillation vial with a magnetic stirrer bar, **1y** (35.3 mg, 0.2 mmol, 1 equiv) and H_2O (5.4 µL, 0.3 mmol, 1.5 equiv) were added. The solution was cooled to 0 ˚C and BBr_3 (1 M in CH_2Cl_2, 250 µL, 0.25 mmol, 1.25 equiv) was added into the mixture. The resultant mixture was allowed to stir for 15 h at 22 ˚C and then quenched at 0 ˚C by saturated aqueous $NaHCO_3$ solution. The organic layer was separated, and the aqueous layer was extracted by CH_2Cl_2 (3 × 5 mL). The combined organic layer was dried over Na_2SO_4, filtered, and concentrated under reduced pressure to yield **2y** as yellow oil (83% yield, 53.0 mg).

R_f = 0.60 (*n*-hexane : diethyl ether = 40 :1)
IR (KBr): 3059 (w), 2084 (w), 1643 (m), 1454 (w), 1102 (w)
^{1}H NMR ($CDCl_3$, 500 MHz) : δ 7.43 (d, *J* = 7.0 Hz, 2H), 7.37 (t, *J* = 8.0 Hz, 2H), 7.31 (t, *J* = 7.5 Hz, 1H), 4.96 (dd, *J* = 8.5, 6.5 Hz, 1H), 2.58–2.50 (m, 1H), 2.47–2.40 (m, 1H), 2.04 (ddd, *J* = 14.5, 11.5, 4.0 Hz, 1H), 1.79–1.73 (m, 7H)
^{13}C NMR ($CDCl_3$, 125.7 MHz) : δ 141.9, 128.9, 128.6, 127.3, 66.5, 55.1, 45.9, 37.0, 34.7, 34.2
**Difficulties with obtaining HRMS for this compound were addressed below.*

Issues with HRMS: Multiple attempts were made to obtain the HRMS of **2t**, **2v**, **2w,** **2y** by APCI/DIP, ESI and EI but none of the target molecules could be traced. We rationalized that the tertiary bromide could be easily eliminated (TLC also showed the partial elimination of **2t**, **2v**, **2w**, **2y** to their corresponding alkenes after left standing in $CHCl_3$). As a representative example, hydrobromination of **1t** was repeated. After working up the reaction, DBU (36.5 mg, 0.24 mmol, 1.2 equiv) and THF (2 mL) were added and the resultant mixture was refluxed for 16 h. H_2O (5 mL) and CH_2Cl_2 (5 mL) were then added to the reaction mixture, and the organic layer was separated. Aqueous layer was extracted by CH_2Cl_2 (3 × 5 mL). The combined organic layer was dried over Na_2SO_4, filtered, and concentrated under reduced pressure. The residue was purified by flash chromatography eluted with *n*-hexane/diethyl ether (40:1) to yield **2t'** as a colourless oil (64% yield over 2 steps, 30.6 mg).

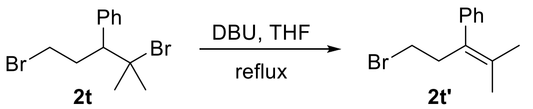

R_f = 0.66 (*n*-hexane : diethyl ether = 40 :1)
IR (KBr): 3057 (w), 2360 (m), (2340 (w), 1636 (w)
^{1}H NMR ($CDCl_3$, 500 MHz) : δ 7.32 (t, *J* = 7.5 Hz, 2H), 7.23 (t, *J* = 7.5 Hz, 1H), 7.10 (d, *J* = 8.0 Hz, 2H), 3.26 (t, *J* = 7.5 Hz, 2H), 2.93 (t, *J* = 7.5 Hz, 2H), 1.86 (s, 3H), 1.56 (s, 3H)
^{13}C NMR ($CDCl_3$, 125.7 MHz) : δ 142.5, 132.1, 131.3, 129.1, 128.3, 126.5, 37.9, 31.5, 22.4, 20.5
HRMS (APCI/DIP) Calc'd for $C_{12}H_{15}Br$ $[M+H]^+$: 239.04299; found 239.04282

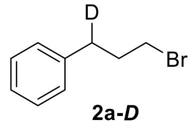

2a-*D*

To a solution of CH_2Cl_2 (3.75 mL) in a 25 mL round-bottom flask with a magnetic stirrer bar, **1a** (118 mg, 1.0 mmol, 1 equiv) and *t*-BuOD (120 μL, 1.25 mmol, 1.25 equiv) were added. The solution was cooled to 0 °C and BBr_3 (1 M in CH_2Cl_2, 1.25 mL, 1.25 mmol, 1.25 equiv) was added into the mixture. The resultant mixture was allowed to stir for 4 h at 22 °C and then quenched at 0 °C by saturated aqueous $NaHCO_3$ solution. The organic layer was separated, and the aqueous layer was extracted by CH_2Cl_2 (3 × 20 mL). The combined organic layer was dried over Na_2SO_4, filtered, and concentrated under reduced pressure. The residue was purified by flash chromatography eluted with *n*-hexane/diethyl ether (50:1) to yield **2a-*D*** as colourless oil (75% yield, 94% D incorporation, 150.0 mg). The % D incorporation is

determined based on the integration of the residual peak relative to other peaks in ^{1}H NMR.

R_f = 0.57 (*n*-hexane: diethyl ether = 50 :1)
IR (KBr): 3060 (w), 2096 (w), 1644 (m), 1494 (w), 1270 (w), 1214 (m), 1076 (m)
^{1}H NMR ($CDCl_3$, 500 MHz) : δ 7.33–7.30 (m, 2H), 7.24–7.21 (m, 3H), 3.41 (t, *J* = 6.5 Hz, 2H), 2.81–2.76 (m, 1H), 2.21–2.16 (m, 2H)
^{13}C NMR ($CDCl_3$, 125.7 MHz) : δ 140.7, 128.7, 128.6, 126.3, 34.2, 33.7 (*J* = 19.6 Hz), 33.2
HRMS (EI) Calc'd for $C_9H_{10}DBr$ $[M]^+$: 199.0101; found 199.0103

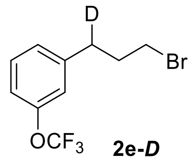

2e-*D*

To a solution of CH_2Cl_2 (1.75 mL) in a 4 mL scintillation vial with a magnetic stirrer bar, **1e** (40.4 mg, 0.2 mmol, 1 equiv) and D_2O (5.4 μL, 0.3 mmol, 1.5 equiv) were added. The solution was cooled to 0 ˚C and BBr_3 (1 M in CH_2Cl_2, 250 μL, 0.25 mmol, 1.25 equiv) was added into the mixture. The resultant mixture was allowed to stir for 72 h at 22 ˚C and then quenched at 0 ˚C by saturated aqueous $NaHCO_3$ solution. The organic layer was separated, and the aqueous layer was extracted by CH_2Cl_2 (3 × 5 mL). The combined organic layer was dried over Na_2SO_4, filtered, and concentrated under reduced pressure. The residue was purified by flash chromatography eluted with *n*-hexane/diethyl ether (40:1) to yield **2e-*D*** as colourless oil (65% yield, >99% D incorporation, 36.9 mg). The % D incorporation is determined based on the integration of the residual peak relative to other peaks in ^{1}H NMR. 6% of di-deuterated product on the benzylic carbon was detected.

R_f = 0.63 (*n*-hexane : diethyl ether = 40 :1)
IR (KBr): 3059 (w), 2099 (w), 1641 (m), 1489 (w), 1257 (m), 1161 (w)
^{1}H NMR ($CDCl_3$, 500 MHz) : δ 7.32 (t, *J* = 7.5 Hz, 1H), 7.14 (d, *J* = 7.5 Hz, 1H), 7.08–7.06 (m, 2H), 3.39 (t, *J* = 6.5 Hz, 2H), 2.83–2.78 (m, 1H), 2.20–2.14 (m, 2H)
^{13}C NMR ($CDCl_3$, 125.7 MHz) : δ 149.6, 143.0, 129.9, 127.1, 121.2, 120.6 (q, *J* = 241.2 Hz), 118.8, 33.8, 33.5 (t, *J* = 19.4 Hz), 32.8
^{19}F NMR ($CDCl_3$, 470.6 MHz) : δ –57.7 (s)
HRMS (APCI/DIP) Calc'd for $C_{10}H_9DBrF_3O$ $[M+H]^+$: 284.00027; found 284.00059

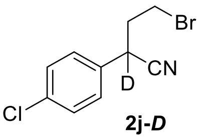

2j-*D*

To a solution of CH_2Cl_2 (0.75 mL) in a 4 mL scintillation vial with a magnetic stirrer bar, **1j** (35.5 mg, 0.2 mmol, 1 equiv) and D_2O (5.4 µL, 0.3 mmol, 1.5 equiv) were added. The solution was cooled to 0 ˚C and BBr_3 (1 M in CH_2Cl_2, 250 µL, 0.25 mmol, 1.25 equiv) was added into the mixture. The resultant mixture was allowed to stir for 22 h at 22 ˚C, then quenched at 0 ˚C by saturated aqueous $NaHCO_3$ solution. The organic layer was separated, and the aqueous layer was extracted by CH_2Cl_2 (3 × 5 mL). The combined organic layer was dried over Na_2SO_4, filtered, and concentrated under reduced pressure to yield **2j-*D*** as yellow oil (quantitative yield, 97% D incorporation, 52.0 mg). The product is analytically pure as shown in ^{1}H and ^{13}C NMR. The % D incorporation is determined based on the integration of the residual peak relative to other peaks in ^{1}H NMR.

R_f = 0.33 (*n*-hexane : diethyl ether = 3 :1)
IR (KBr): 3060 (w), 1640 (m), 1492 (m), 1284 (w), 1093 (m), 1014 (w), 823 (w)
^{1}H NMR ($CDCl_3$, 500 MHz) : δ 7.38 (dt, *J* = 8.5, 2.0 Hz, 2H), 7.31 (dt, *J* = 8.5, 2.0 Hz, 2H), 3.54 (ddd, *J* = 10.5, 8.0, 5.0 Hz, 1H), 3.35 (ddd, *J* = 11.0, 7.0, 5.0 Hz, 1H), 2.49–2.44 (m, 1H), 2.33–2.27 (m, 1H)
^{13}C NMR ($CDCl_3$, 125.7 MHz) : δ 134.8, 132.8, 129.7, 128.9, 119.6, 38.1, 34.8 (t, *J* = 20.1 Hz), 29.2
HRMS (APCI/DIP) Calc'd for $C_{10}H_8DBrClN$ $[M+H]^+$: 260.97201; found 260.97215

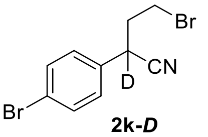

2k-*D*

To a solution of CH_2Cl_2 (0.75 mL) in a 4 mL scintillation vial with a magnetic stirrer bar, **1k** (44.4 mg, 0.2 mmol, 1 equiv) and D_2O (5.4 µL, 0.3 mmol, 1.5 equiv) were added. The solution was cooled to 0 ˚C and BBr_3 (1 M in CH_2Cl_2, 250 µL, 0.25 mmol, 1.25 equiv) was added into the mixture. The resultant mixture was allowed to stir for 22 h at 22 ˚C and then quenched at 0 ˚C by saturated aqueous $NaHCO_3$ solution. The organic layer was separated, and the aqueous layer was extracted by CH_2Cl_2 (3 × 5 mL). The combined organic layer was dried over Na_2SO_4, filtered, and concentrated under reduced pressure to yield **2k-*D*** as a colourless oil (quantitative yield, 97% D incorporation, 60.9 mg). The product is analytically pure as shown in ^{1}H and ^{13}C NMR. The % D incorporation is determined based on the integration of the residual peak relative to other peaks in ^{1}H NMR.

R_f = 0.33 (*n*-hexane : diethyl ether = 3 :1)

IR (KBr): 3343, 2943, 1453, 1049, 758, 700 cm^{-1}

^{1}H NMR (CDCl$_3$, 500 MHz) : δ 7.54 (dt, *J* = 8.5, 2.0 Hz, 2H), 7.25 (dt, *J* = 8.5, 2.0 Hz, 2H), 3.53 (ddd, *J* = 11.0, 8.0, 5.0 Hz, 1H), 3.34 (ddd, *J* = 11.0, 7.0, 5.0 Hz, 1H), 2.49–2.44 (m, 1H), 2.32–2.27 (m, 1H)

^{13}C NMR (CDCl$_3$, 125.7 MHz) : δ 133.3, 132.6, 129.2, 122.8, 119.5, 38.0, 34.9 (t, *J* = 21.4 Hz), 29.2

HRMS (APCI/DIP) Calc'd for $C_{10}H_8DBr_2N$ [M+H]$^+$: 304.92171; found 304.92193

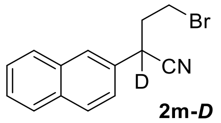

2m-*D*

To a solution of CH_2Cl_2 (1.75 mL) in a 4 mL scintillation vial with a magnetic stirrer bar, **1m** (38.7 mg, 0.2 mmol, 1 equiv) and D_2O (5.4 μL, 0.3 mmol, 1.5 equiv) were added. The solution was cooled to 0 ˚C and BBr_3 (1 M in CH_2Cl_2, 250 μL, 0.25 mmol, 1.25 equiv) was added into the mixture. The resultant mixture was allowed to stir for 18 h at 22 ˚C and then quenched at 0 ˚C by saturated aqueous $NaHCO_3$ solution. The organic layer was separated, and the aqueous layer was extracted by CH_2Cl_2 (3 × 5 mL). The combined organic layer was dried over Na_2SO_4, filtered, and concentrated under reduced pressure to yield **2m-*D*** as yellow oil (quantitative yield, 97% D incorporation, 55.0 mg). The product is analytically pure as shown in ^{1}H and ^{13}C NMR. The % D incorporation is determined based on the integration of the residual peak relative to other peaks in ^{1}H NMR.

R_f = 0.33 (*n*-hexane : diethyl ether = 3 :1)

IR (KBr): 3062 (w), 2241 (w), 1635 (m), 1280 (w), 817 (w), 749 (m), 478 (w)

^{1}H NMR (CDCl$_3$, 500 MHz) : δ 7.90–7.85 (m, 4H), 7.56–7.52 (m, 2H), 7.43 (dd, *J* = 8.5, 1.5 Hz, 1H), 4.30 (t, *J* = 7.5 Hz, 0.03H), 3.57 (ddd, *J* = 11.0, 7.5, 5.0 Hz, 1H), 3.38 (ddd, *J* = 11.0, 6.5, 5.0 Hz, 1H), 2.59–2.54 (m, 1H), 2.46–2.40 (m, 1H)

^{13}C NMR (CDCl$_3$, 125.7 MHz) : δ 133.3, 133.1, 131.5, 129.5, 128.0, 127.9, 127.0, 126.87, 126.85, 124.6, 120.0, 38.1, 35.5 (*J* = 20.9 Hz), 29.5

HRMS (APCI/DIP) Calc'd for $C_{14}H_{11}DBrN$ [M+H]$^+$: 275.02887; found 275.02974

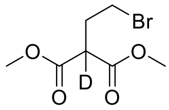

2o-*D*

To a solution of CH_2Cl_2 (8.75 mL) in a 25 mL round-bottom flask with a magnetic

stirrer bar, **1o** (158.2 mg, 1.0 mmol, 1 equiv) and D_2O (27.0 μL, 1.5 mmol, 1.5 equiv) were added. The solution was cooled to 0 ˚C and BBr_3 (1 M in CH_2Cl_2, 1.25 mL, 1.25 mmol, 1.25 equiv) was added into the mixture in one portion by autopipette. The mixture was allowed to stir for 15 h at 22 ˚C and then quenched at 0 ˚C by saturated aqueous $NaHCO_3$ solution. The organic layer was separated, and the aqueous layer was extracted by CH_2Cl_2 (3 × 20 mL). The combined organic layer was dried over Na_2SO_4, filtered, and concentrated under reduced pressure to yield **2o-*d*** as light yellow oil (70% yield using CH_2Br_2 as internal standard, >97% D incorporation). All deuterium was exchanged to hydrogen upon treating the crude mixture with silica gel. The assignment of ^1H and ^{13}C NMR peaks of **2o-*D*** was based on that of **1o**. The % D incorporation was determined based on the integration of the residual peak relative to other peaks in crude ^1H NMR.

IR (KBr): 3060 (w), 1734 (m), 1647 (m), 1218 (w), 1079 (m), 816 (w)
^{1}H NMR ($CDCl_3$, 500 MHz) : δ 3.70 (s, 6H), 3.40 (t, *J* = 6.5 Hz, 2H), 2.37 (t, *J* = 6.5 Hz, 2H)
^{13}C NMR ($CDCl_3$, 125.7 MHz) : δ 168.9, 52.8, 49.5 (t, *J* = 20.4), 31.4, 30.3
HRMS (ESI) Calc'd for $C_7H_{10}DBrO_4$ $[M+Na]^+$: 261.97957; found 261.97957

(D) Further Transformations of 2a

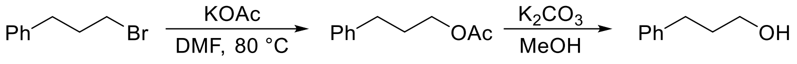

To a solution of DMF (10 mL) and KOAc (197 mg, 2 mmol, 2 equiv) was added **1a** (199 mg, 1 mmol, 1 equiv) and the resultant mixture was heated at 80 ˚C for 12 h. The reaction was cooled to room temperature and diluted with H_2O (50 mL). The aqueous layer was extracted by diethyl ether (3 × 20 mL). The combined organic layer was dried over Na_2SO_4, filtered, and concentrated under reduced pressure to yield **5a-OAc** which was used in the next step without further purification.

To a solution of **5a-OAc** in MeOH (8 mL) was added K_2CO_3 (346 mg, 2.5 mmol, 2.5 equiv) and the resultant mixture was allowed to stir for 12 h at 23 ˚C. The reaction was diluted with H_2O (30 mL) and the aqueous layer was extracted by ethyl acetate (3 × 20 mL). The combined organic layer was dried over Na_2SO_4, filtered, and concentrated under reduced pressure. The residue was purified by flash chromatography eluted with *n*-hexane/ethyl acetate (1:1) to yield **5a** as colourless oil (93%, 127 mg).

R_f = 0.57 (*n*-hexane : ethyl acetate = 1 :1)
^{1}H NMR ($CDCl_3$, 500 MHz) : δ 7.35–7.32 (m, 2H), 7.25–7.22 (m, 3H), 3.68 (t, *J* = 6.5 Hz, 2H), 2.75–2.72 (m, 3H), 1.95–1.90 (m, 2H)
^{13}C NMR ($CDCl_3$, 125.7 MHz) : δ 141.9, 128.42, 128.38, 125.8, 62.0, 34.2, 32.1
Data matches with literature reported values (G. Chen, C. Fu, S. Ma, *Tetrahedron* 2006, **62**, 4444)

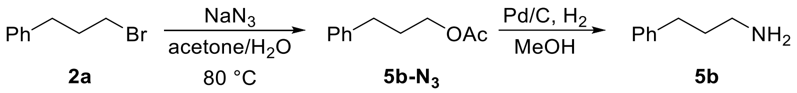

To a solution of acetone/H_2O (1:1, 10 mL) and **1a** (199 mg, 1 mmol, 1 equiv) was added NaN_3 (195 mg, 3 mmol, 3 equiv) and the resultant mixture was heated at 80 ˚C for 12 h. The reaction was cooled to room temperature and concentrated under reduced pressure to remove most of the acetone. The remaining aqueous layer was the extracted by diethyl ether (3 × 20 mL). The combined organic layer was dried over Na_2SO_4, filtered, and concentrated under reduced pressure to yield **5b-N_3** which was used in the next step without further purification.

To a solution of **5b-N$_3$** in MeOH (10 mL) was added catalytic amount of Pd/C (~10 wt %). The flask was evacuated and backfilled with H_2 for three times. The resultant mixture was then allowed to stir for 12 h under H_2 (with a balloon) at 23 ˚C. The mixture was filtered through a thin plug of celite and the residue was washed with ethyl acetate (20 mL). The filtrate was concentrated under reduced pressure and the residue was purified by flash chromatography eluted with $CHCl_3$/methanol (9:1) and 1% NH_3 solution (28% w/w) to yield **5b** as a colourless oil (92%, 124 mg).

R_f = 0.30 ($CHCl_3$: MeOH : NH_3 solution (28% w/w) = 90 : 9 : 1)
^{1}H NMR ($CDCl_3$, 500 MHz) : δ 7.29–7.26 (m, 2H), 7.19–7.16 (m, 3H), 2.72 (t, *J* = 7.0 Hz, 2H), 2.65 (t, *J* = 7.5 Hz, 2H), 1.77 (quint, *J* = 7.5 Hz, 2H), 1.65 (br, 2H)
^{13}C NMR ($CDCl_3$, 125.7 MHz) : δ 142.1, 128.39, 128.37, 125.8, 41.7, 35.3, 33.3
Data matches with literature reported values (L. Benati, G. Bencivenni, R. Leardini, D. Nanni, M. Minozzi, P. Spagnolo, R. Scialpi, G. Zanardi, *Org. Lett.* 2006, **8**, 2499)

(E) Control Experiments

Scheme S1. Preliminary trials with different halogen sources as radical quenchers.

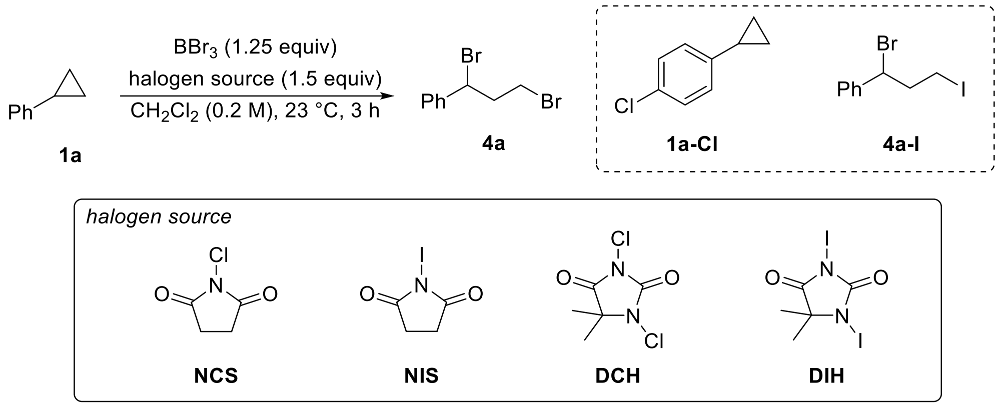

halogen source	4a	
NCS	49%	+ **1a-Cl** (15%)
DCH	26%	+ messy mixture
NIS	0%	+ unidentified products
DIH	12%	+ **4a**-I (45%)

Scheme S2. Effect of reintroducing O_2 to the reaction

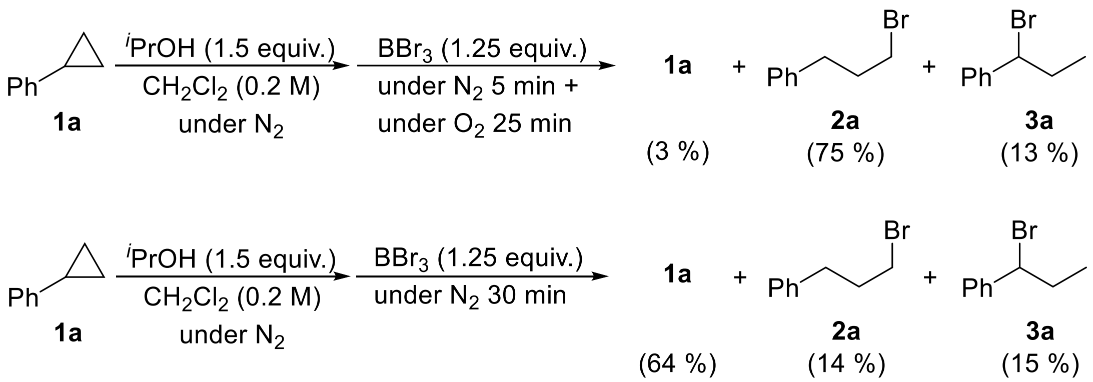

Scheme S3. Effect of the hydroxyl substituent on substrate **1y**.

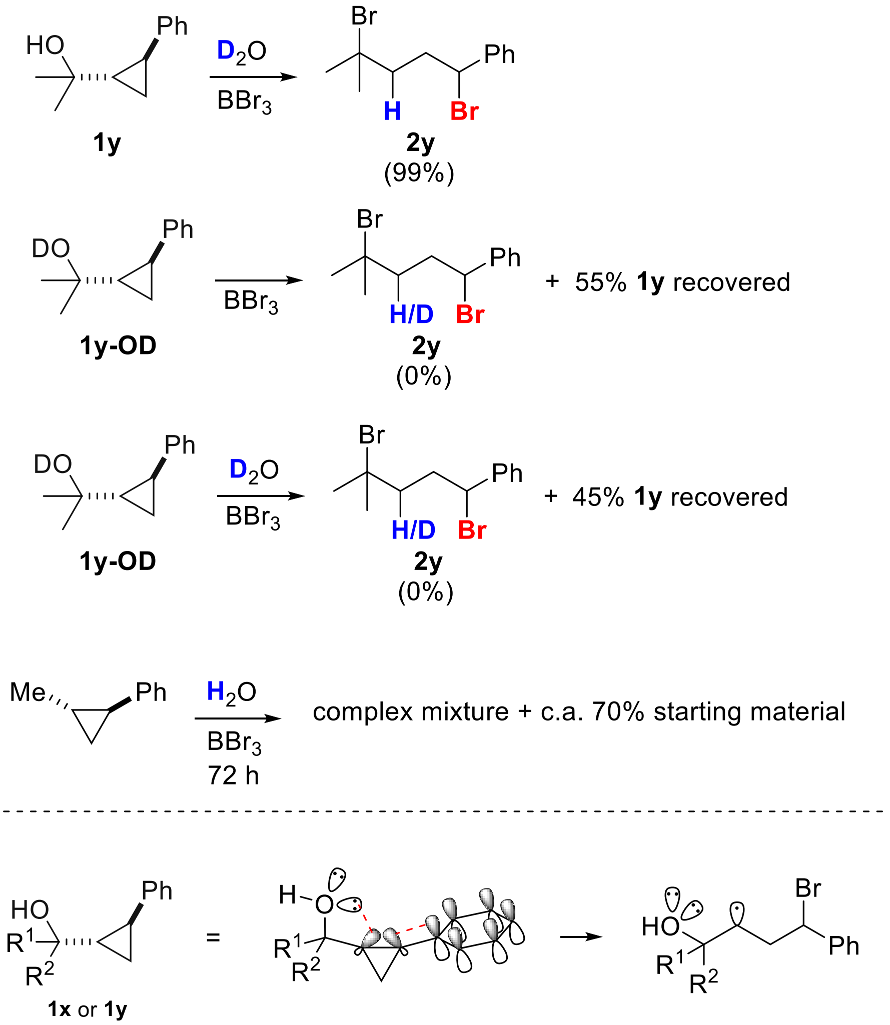

*Note: The reaction of **1y** was carried out with D_2O as the proton source. The hydrobromination product **2y** was obtained in excellent yield but there was no deuterium incorporation in the product. When the deuterated **1y** (i.e. **1y-OD**) was used as the substrate in the absence or absence of D_2O, no **2y** was detected and significant amount of **1y** was recovered. These results indicate that the OH group in **1y** is crucial for the hydrobromination. It is known that cyclopropanes have a bisected conformation with π-character σ-bonds.[5] A possible explanation is that the phenyl ring (via π-conjugation)[6-7] and the OH group (via neighboring group effect)[8-9] might activate the C-C bond in cyclopropane synergistically. This might also explain the low reactivity of 1-phenyl-2-methylcyclopropane that has no hydroxyl substituent.*

(F) NMR Studies

For all NMR experiments that were done under N_2, NMR tubes were repeatedly dried under high vacuum and then refilled with N_2. All reagents and solvent (except BBr_3) were added to an oven-dried Schlenk equipped with stirrer bar under N_2, then degassed with three freeze-pump-thaw cycles under N_2. The solution was cooled to 0 °C with an ice bath, and BBr_3 (1M in CH_2Cl_2) was then added with autopipette to the stirring solution. The reaction mixture was quickly transferred to the NMR tube with cannula or syringe.

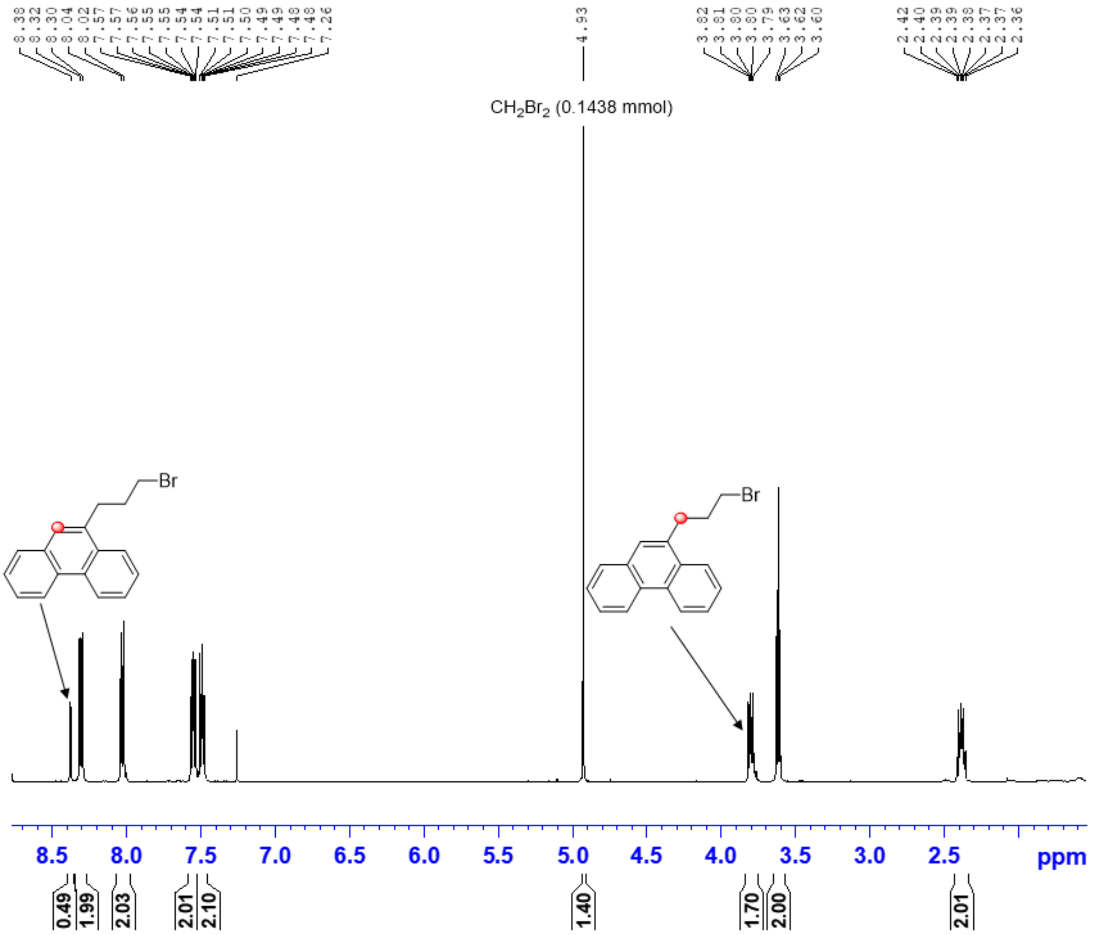

Figure S1. Deuteriobromination of **1g**

*Note: the carbons in which protons were exchanged with deuteriums are marked with a red dot. The reaction was carried out with **1g** (0.2 mmol), BBr_3 (0.25 mmol) and t-BuOD (0.3 mmol) in CD_2Cl_2 for 2 hours. The reaction was then quenched with saturated $NaHCO_3$ solution and ^1H NMR (500 MHz) was carried out on the crude mixture with CH_2Br_2 (0.1438 mmol) as internal standard.*

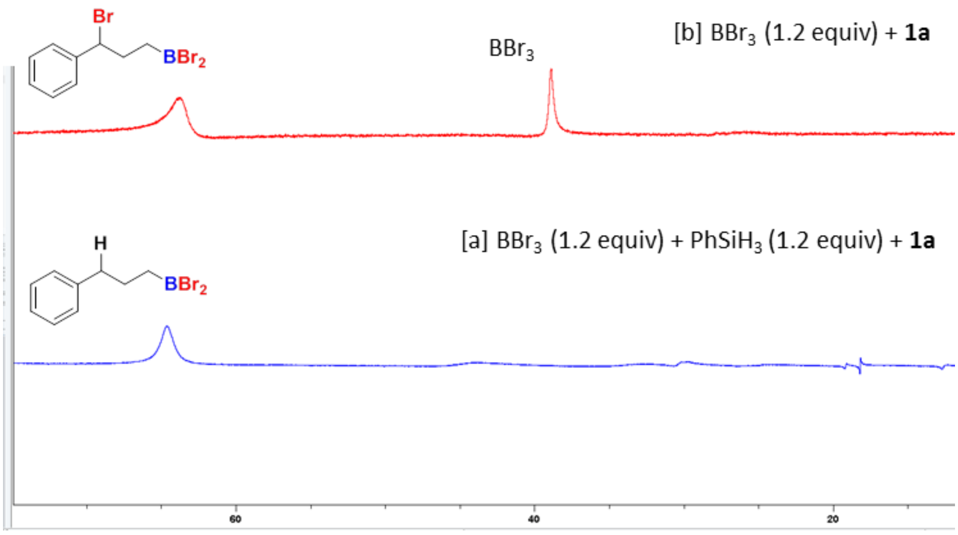

Figure S2. ^{11}B NMR experiment of BBr_3 and **1a** under N_2

Note: ^{11}B *NMR experiments in* CD_2Cl_2 *under* N_2 *were conducted with [a]* BBr_3 *(1.2 equiv),* $PhSiH_3$ *(1.2 equiv) and* ***1a*** *(1.0 equiv) for 20 min; [b]* BBr_3 *(1.2 equiv) and* ***1a*** *(1.0 equiv) for 20 min.*

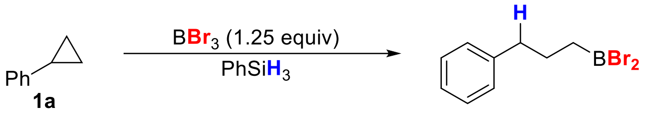

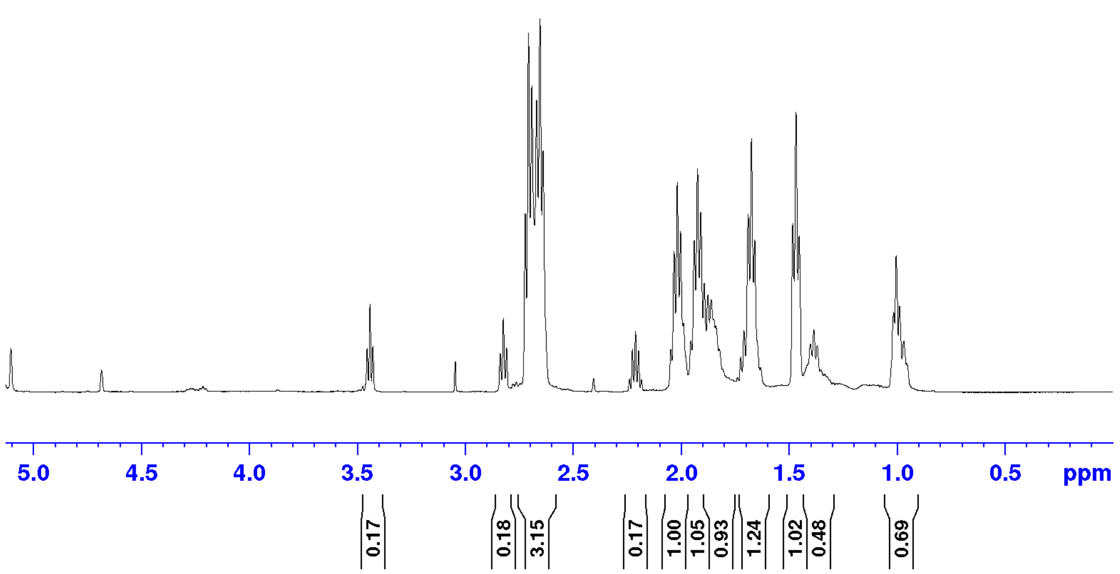

Figure S3. ^{1}H NMR experiment of BBr_3, $PhSiH_3$ and **1a**

*Note: ^{1}H NMR (500 MHz) study on a mixture of BBr_3 (1.2 equiv), $PhSiH_3$ (1.2 equiv) and **1a** (1.0 equiv) in CD_2Cl_2 under N_2 at 23 °C for 20 min was conducted.*

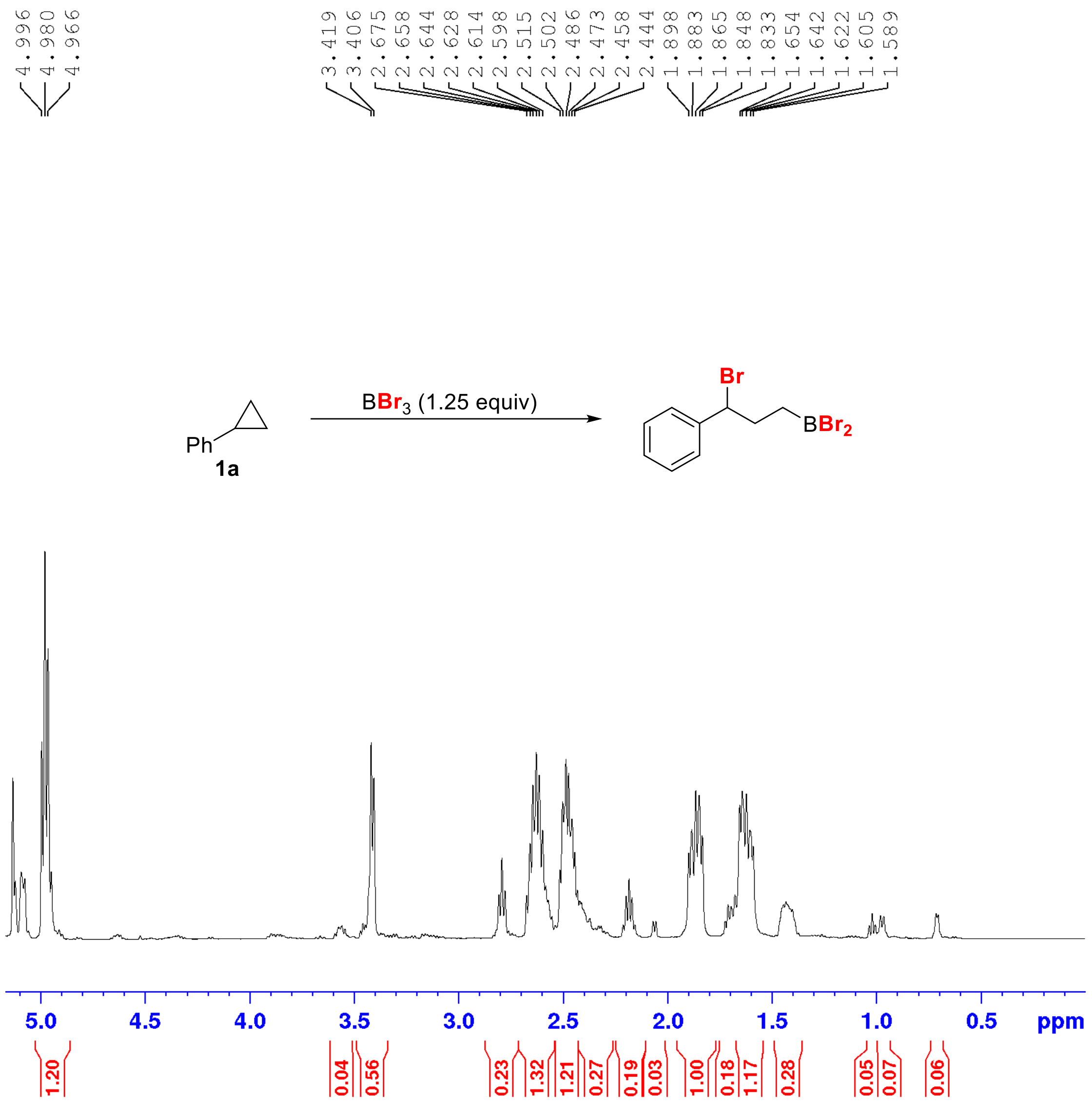

Figure S4. ^{1}H NMR experiment of BBr_3 and **1a**

Note: ^{1}H NMR (500 MHz) study on a mixture of BBr_3 (1.2 equiv) and ***1a*** *(1.0 equiv) in CD_2Cl_2 under N_2 at 23 °C for 20 min was conducted.*

^{1}H NMR (500 MHz)

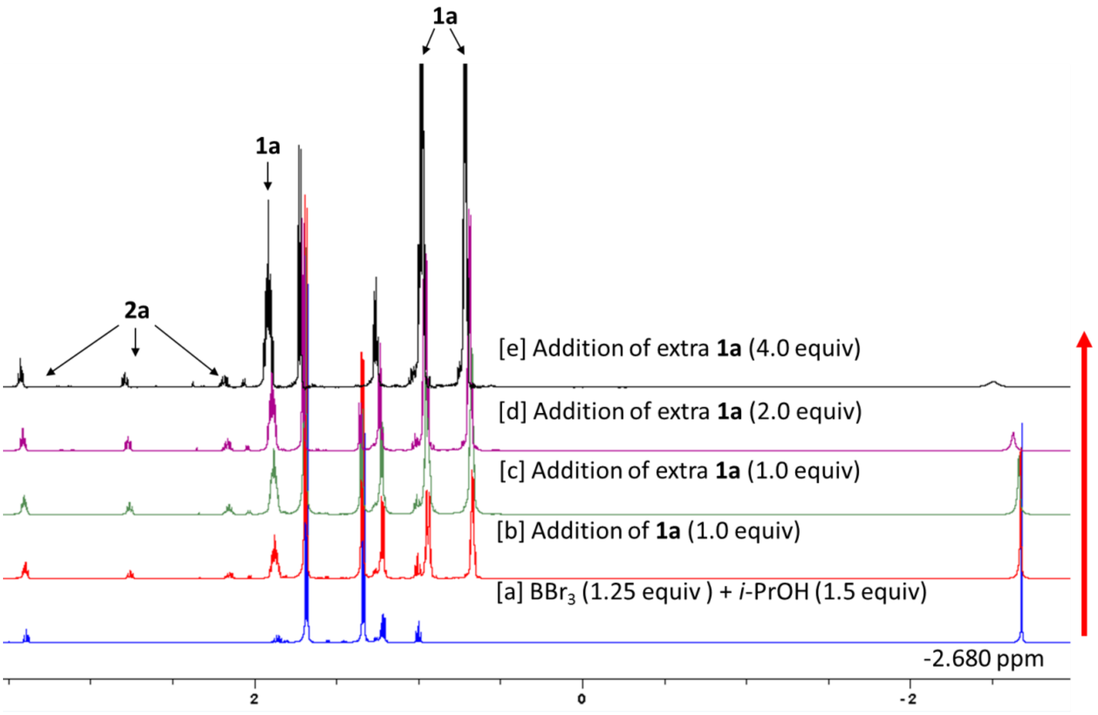

^{11}B NMR (128 MHz)

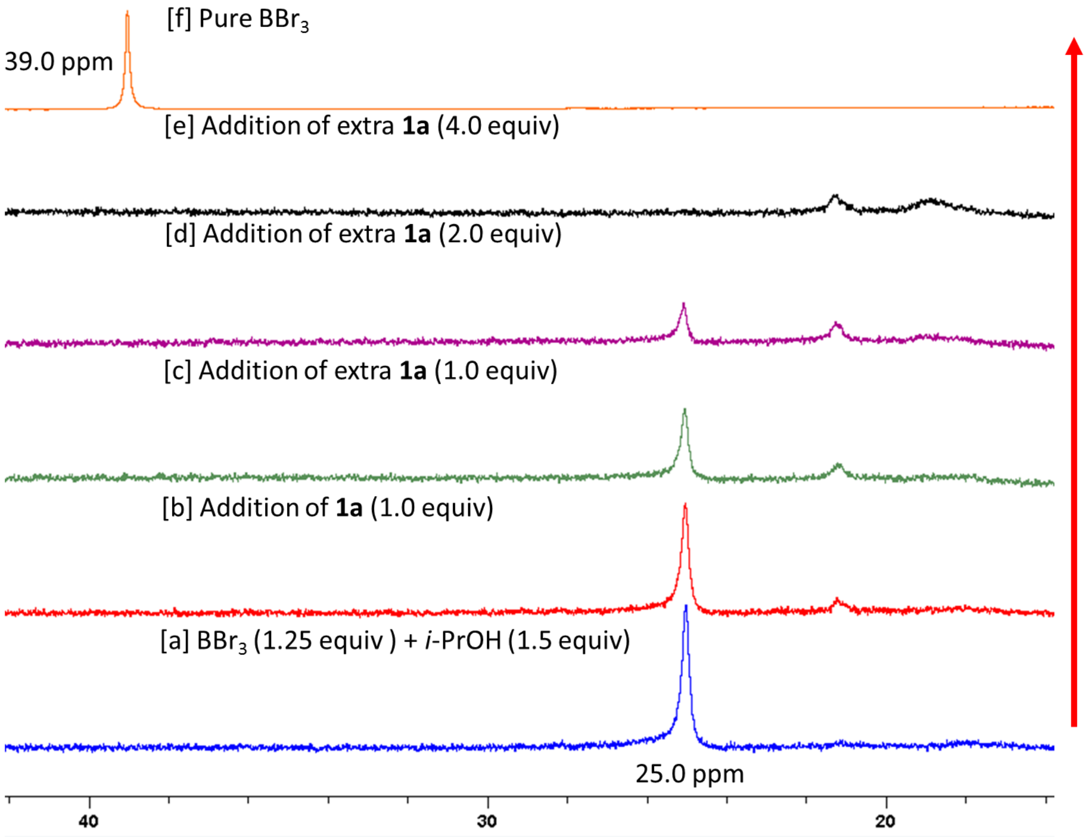

Figure S5. NMR experiments on the reactions among BBr_3, *i*-PrOH and **1a**

*Note: ^{11}B NMR experiments on a mixture of BBr_3 and i-PrOH in CD_2Cl_2 at 23 ^oC under air were conducted. Different species were added to the same sample, in the order from bottom to top for each set of spectra: [a] BBr_3 (1.25 equiv) + i-PrOH (1.5 equiv); [b] addition of **1a** (1.0 equiv); [c] addition of extra **1a** (1.0 equiv); [d] addition of extra **1a** (2.0 equiv); [e] addition of extra **1a** (4.0 equiv); [f] reference signal: pure BBr_3 (1M in CH_2Cl_2) for ^{11}B NMR. It was noted that the signal 25.0 ppm diminished gradually upon the addition of cyclopropane substrate **1a**.*

^{11}B NMR (128 MHz)

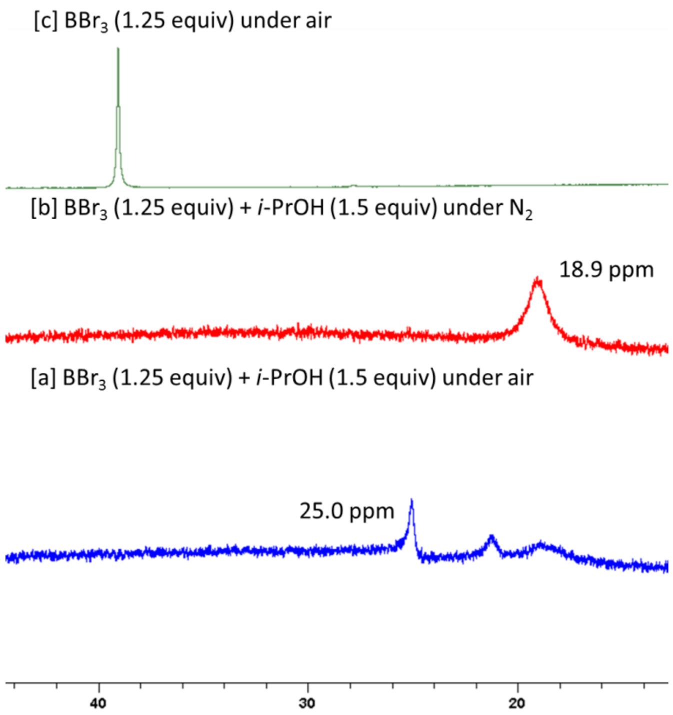

Figure S6. ^{11}B NMR experiments to study the role of O_2

Note: ^{11}B NMR experiments on a mixture of BBr_3 and i-PrOH in CD_2Cl_2 at 23 ^oC in sealed NMR tubes were conducted under different conditions. [a] under air; [b] under N_2; [c] under air and in the absence of i-PrOH. The signal at 25.0 ppm was formed only when all the three components (BBr_3, i-PrOH and oxygen) were present in the reaction system. The signal at 18.9 ppm could be assigned to the complex formed between BBr_3 and i-PrOH.

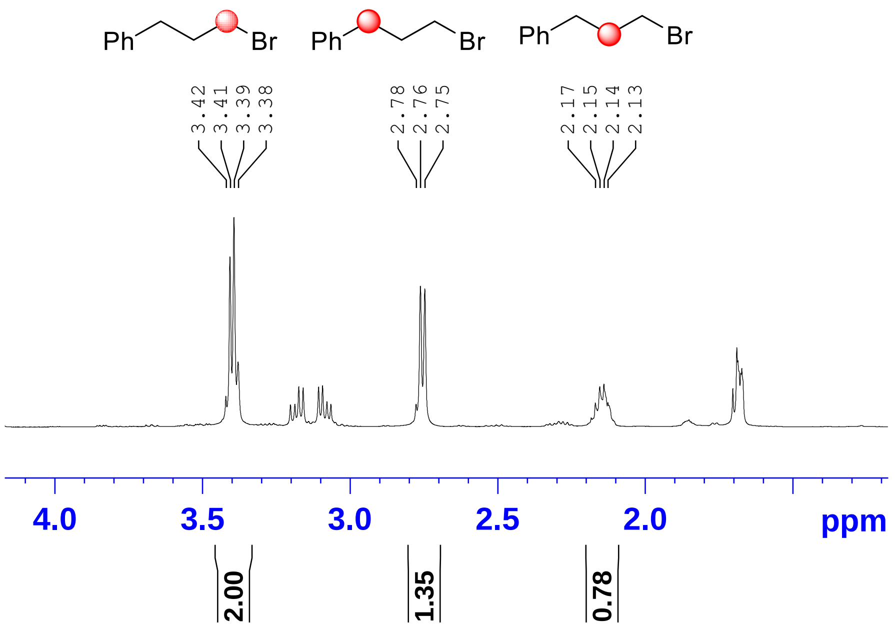

Note: ^{1}H NMR (500 MHz) study on a mixture of allylbenzene (1 equiv), BBr_3 (1.25 equiv) and D_2O (1.5 equiv) in CD_2Cl_2 at 23 °C was conducted. Allylbenzene was added in one portion. Chemical shift of each proton is labelled accordingly.

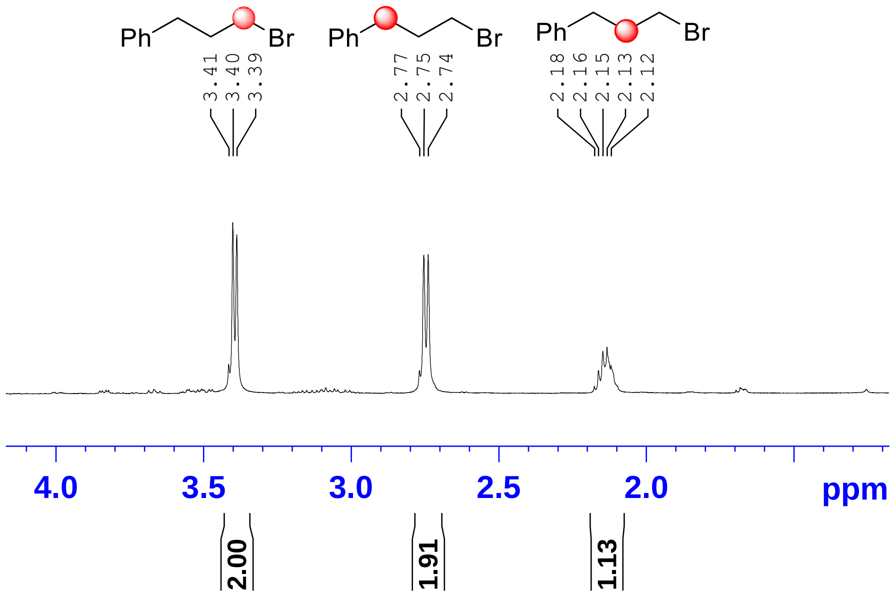

Note: ^{1}H NMR (500 MHz) study on a mixture of allylbenzene (1 equiv), BBr_3 (1.25 equiv) and D_2O (1.5 equiv) in CD_2Cl_2 at 23 °C was conducted. Allylbenzene was added portion-wise with each potion c.a. 0.1 equiv over 40 minutes to mimic the slow generation of allylbenzene in the system. Chemical shift of each proton is labelled accordingly.

Figure S7. Control experiments with allylbenzene

(G) Computational Studies

DFT calculations were performed using Gaussian 16.[10] Geometry optimizations and frequency calculations were performed at the ωB97X-D[11]/6-31+G(d,p) level of theory. A pruned (99,590) grid (default in Gaussian 16) was used in geometry optimizations to minimize orientational variations in calculated free energy corrections.[12] Thermal contributions to free energies were calculated from vibrational frequencies using the quasi-rigid rotor-harmonic oscillator (RRHO) approach of Grimme.[13] Optimized geometries were confirmed by frequency computations as minima (no imaginary frequency) or first-order saddle-point (one imaginary frequency) structures. Single-point energy calculations were performed at the ωB97X-D/6-311+G(d,p), SMD[14] (CH_2Cl_2) level of theory. Conformational searches were carried out in Spartan '18[15] using the MMFFs force field.

The computed free energy diagram of the proposed reaction pathway is shown in the manuscript (Figure S1). The reaction free energies (kcal/mol) of some key competing processes are shown below.

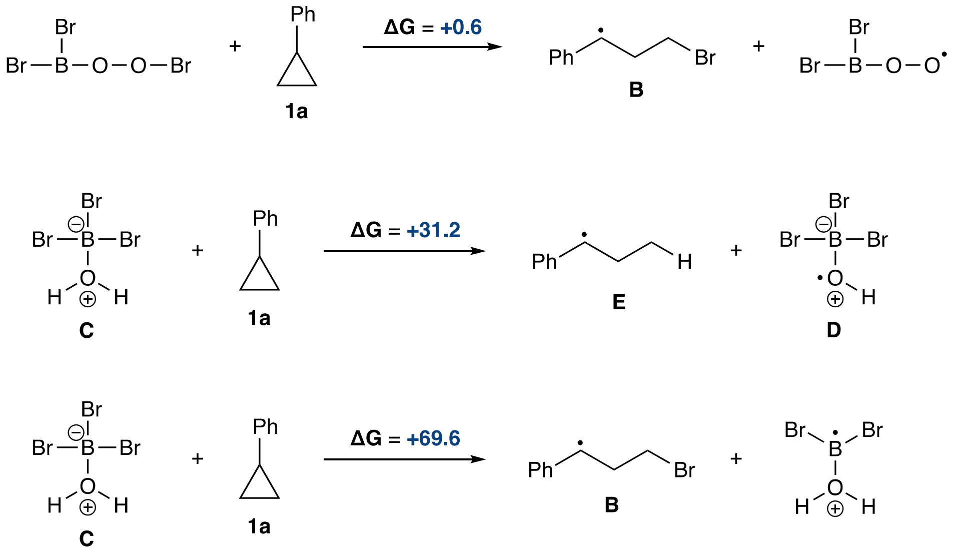

Cartesian Coordinates and Energies of Calculated Structures

BBr_3

B	0.00000000	0.00000000	0.00000000
Br	0.00000000	1.89381800	0.00000000
Br	1.64009500	-0.94690900	0.00000000
Br	-1.64009500	-0.94690900	0.00000000

O_2

O	0.00000000	0.00000000	0.60271200
O	0.00000000	0.00000000	-0.60271200

H_2O

O	0.00000000	0.00000000	0.11561600
H	0.00000000	0.76650100	-0.46246200
H	0.00000000	-0.76650100	-0.46246200

A

B	-0.76385500	0.08848400	0.32024100
Br	-2.37494500	0.92232900	-0.44511800
Br	-0.34098500	-1.75152200	-0.21578900
O	0.25219400	1.06729200	0.32684100
O	1.41421100	0.54412800	0.96212800
Br	2.79122400	0.47522600	-0.25750400
O	-1.11666800	-0.11014200	1.96991400
H	-1.42241400	0.72461100	2.35599100
H	-1.79151000	-0.78842500	2.11611500

1a

C	-0.56064000	-1.20179700	0.15394400
C	0.14355700	-0.00016000	0.27209900
C	-0.56029000	1.20165900	0.15382300
C	-1.93428300	1.20449900	-0.07597200
C	-2.62592800	0.00020800	-0.19128900
C	-1.93462600	-1.20427500	-0.07583400
H	-0.02204800	-2.14180800	0.23962000
H	-0.02144500	2.14152800	0.23944800
H	-2.46474300	2.14757900	-0.16763600
H	-3.69622600	0.00035400	-0.37320800
H	-2.46535400	-2.14721300	-0.16740400
C	1.61476300	-0.00043400	0.55573500

C	2.56964100	-0.75385000	-0.33353000
C	2.56976000	0.75411200	-0.33241100
H	1.86241000	-0.00126100	1.61582700
H	2.15319200	-1.25215000	-1.20376700
H	3.40441900	-1.27026600	0.12889200
H	3.40462600	1.26970500	0.13077000
H	2.15343600	1.25376100	-1.20193500

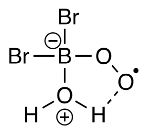

B	-0.00242600	0.29557700	0.28238300
Br	-1.66431000	-0.69758300	-0.04735100
Br	1.70357800	-0.61706500	-0.08846400
O	-0.08782500	1.41261100	-0.91986200
O	-0.15319800	2.62893000	-0.43555000
O	-0.01097100	1.09817900	1.45127100
H	0.75942700	0.96340800	2.01047700
H	-0.10572300	2.45363500	0.56427400

B

C	3.54806400	-1.48933800	-0.00000500
C	2.23815900	-1.03574800	-0.00000200
C	1.94820200	0.35468200	0.00003900
C	3.04946900	1.25264300	-0.00001800
C	4.35329500	0.78881900	-0.00001300
C	4.61430600	-0.58585300	-0.00000300
H	3.74501700	-2.55709700	-0.00003200
H	1.42522200	-1.75515200	-0.00001300
H	2.85410800	2.32174400	-0.00003600
H	5.17660000	1.49656700	-0.00003100
H	5.63722400	-0.94795300	-0.00000500
C	0.62389700	0.85155600	0.00001400
H	0.48653300	1.93129300	-0.00011400
C	-0.59626600	-0.01818300	0.00003700
H	-0.60137600	-0.67958200	0.87787900
H	-0.60139300	-0.67965400	-0.87772000
C	-1.85177700	0.83207500	0.00002100
H	-1.90472400	1.46288700	0.88806200
H	-1.90472700	1.46289000	-0.88801700
Br	-3.48218800	-0.22171000	-0.00001100

C

B	0.00003600	-0.02570800	0.23544100
Br	-0.00465700	1.90754000	-0.20724000
Br	-1.67017100	-0.96052200	-0.19393900
Br	1.67488700	-0.95255900	-0.19388200
O	0.00003600	-0.05527500	1.89391800
H	0.78846200	0.38468200	2.24934100
H	-0.79101100	0.38003400	2.24926300

2a

C	3.45237600	-1.53702900	-0.00004800
C	2.16549600	-0.99635300	-0.00023300
C	1.97127800	0.38577700	-0.00002200
C	3.10133800	1.21282000	0.00016900
C	4.38460900	0.67905300	0.00027300
C	4.56563400	-0.70409200	0.00023500
H	3.57937300	-2.61534000	-0.00019000
H	1.31499900	-1.67036800	-0.00055900
H	2.96913200	2.29246700	0.00015600
H	5.24488500	1.34145600	0.00043100
H	5.56582500	-1.12558100	0.00034200
C	0.60015400	1.03196900	-0.00038600
H	0.52934300	1.68981500	-0.87669300
C	-0.59049700	0.07163500	-0.00048300
H	-0.55814600	-0.57675600	0.88168400
H	-0.55849400	-0.57641200	-0.88292500
C	-1.88374500	0.86095400	-0.00004600
H	-1.97143100	1.48941800	0.88732300
H	-1.97183500	1.48978400	-0.88712400
Br	-3.46493200	-0.27019100	0.00013900
H	0.52911400	1.68978900	0.87591300

D

B	-0.33557900	0.00317200	0.64705900
Br	1.07246500	1.39635200	-0.17616100
Br	-2.02860200	-0.00254800	-0.26976000
Br	1.07687300	-1.39515700	-0.17424300
O	-0.37719900	0.00296000	2.00192700
H	0.46972500	0.00785400	2.45502200

$HOBBr_2$

B	-0.01135000	0.62752800	0.00033600

Br	1.66114700	-0.31694100	-0.00001000
Br	-1.67502900	-0.29080200	-0.00002400
O	-0.03615100	1.96863300	0.00005600
H	0.83185000	2.38427900	-0.00095600

E

C	1.95071000	1.32683700	0.00000500
C	0.58640700	1.07911100	0.00000400
C	0.08434900	-0.25005800	-0.00000100
C	1.03637800	-1.30547000	0.00000100
C	2.39617900	-1.04721000	0.00000200
C	2.86628900	0.27091000	0.00000400
H	2.30893200	2.35204200	0.00001000
H	-0.10741000	1.91401100	0.00000600
H	0.67945300	-2.33208300	0.00000300
H	3.10087000	-1.87335900	0.00000300
H	3.93278500	0.47154000	0.00000000
C	-1.30225800	-0.53676000	-0.00001200
H	-1.60119400	-1.58369800	-0.00003500
C	-2.38482800	0.49632500	-0.00002600
H	-2.27079100	1.15445000	-0.87486100
H	-2.27075100	1.15451500	0.87475300
C	-3.78816700	-0.10981200	0.00002600
H	-4.55494800	0.66987500	0.00001300
H	-3.94366400	-0.73529300	-0.88503800
H	-3.94362900	-0.73523200	0.88513700

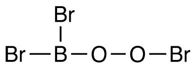

B	-0.83527200	-0.06305700	-0.00018500
Br	-2.15683400	-1.42278500	0.00003400
Br	-1.18941200	1.79320400	0.00003400
O	0.43028800	-0.56411300	-0.00022800
O	1.41692600	0.48528400	-0.00021400
Br	3.04335000	-0.34339300	0.00006000

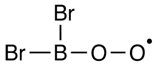

B	0.00005200	0.46750000	0.00000000
Br	0.00005200	-0.45108500	-1.68284400
Br	0.00005200	-0.45108500	1.68284400
O	0.66759500	1.82752700	0.00000000

O	-0.66807900	1.82728200	0.00000000

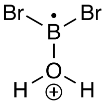

B	0.00767100	0.43992900	-0.57360800
Br	-1.69570600	-0.32452300	0.03067200
Br	1.69806800	-0.31541100	0.02493900
O	0.03736700	1.97700200	0.00045900
H	-0.85836300	2.34721800	0.03274400
H	0.43842500	2.03481800	0.88524500

Table S1. Calculated energies (in Hartrees). ΔG values provided are corrected values after applying the quasi-rigid rotor-harmonic oscillator (RRHO) approach of Grimme.[13]

	**ΔG** [ωB97X-D/6-31+G(d,p)]	**E** [ωB97X-D/6-311+G(d,p), SMD(CH_2Cl_2)// ωB97X-D/6-31+G(d,p)]	**G** (**E** + **ΔG**)
**BBr_3**	-0.02441	-7747.482615	-7747.507025
**O_2**	-0.016044	-150.3188962	-150.334940
**H_2O**	0.004123	-76.44045641	-76.436333
**A**	0.000775	-7974.24318	-7974.242405
**1a**	0.131582	-348.9291567	-348.797575
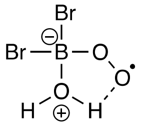	0.003644	-5400.081053	-5400.077409
**B**	0.12743	-2923.102132	-2922.974702
**C**	-0.00245	-7823.941437	-7823.943887
**2a**	0.142247	-2923.753324	-2923.611077
**D**	-0.015861	-7823.290662	-7823.306523
**$HOBBr_2$**	-0.009874	-5249.16193	-5249.171804
**E**	0.13831	-349.5234664	-349.385156
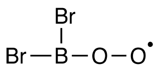	-0.00262	-5249.653175	-5249.655795
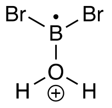	-0.020965	-5323.608436	-5323.629401

(H) References

1. D. Cheng, D. Huang, Y. Shi, *Org. Biomol. Chem*. 2013, **11**, 5588.
2. D. J. Wallace, C. Chen, *Tetrahedron Lett.* 2002, **43**, 6987.
3. M. H. Gieuw, V. M-Y. Leung, Z. Ke, Y.-Y. Yeung, *Adv. Synth. Cat.* 2018, **360**, 4306.
4. J. Xu, N. B. Samsuri, H. A. Duong, *Chem. Commun.* 2016, **52**, 3372.
5. A. D. Walsh, *Trans. Faraday Soc.* 1970, **45**, 179.
6. J. W. Lauher, J. A. Ibers, *J. Am. Chem. Soc.* 1975, **97**, 561.
7. S. Rosenfeld, C. W. Tingle, *J. Am. Chem. Soc.* 1994, **116**, 12049.
8. H. Zipse, *Top. Curr. Chem.* 2006, **236**, 163.
9. J. Berges, N. Varmenot, A. Scemama, Z. Abedinzadeh, K. Bobrowski, *J. Phys. Chem. A* 2008, **112**, 7015.
10. Gaussian 16, Revision A.03, M. J. Frisch, G. W. Trucks, H. B. Schlegel, G. E. Scuseria, M. A. Robb, J. R. Cheeseman, G. Scalmani, V. Barone, G. A. Petersson, H. Nakatsuji, X. Li, M. Caricato, A. V. Marenich, J. Bloino, B. G. Janesko, R. Gomperts, B. Mennucci, H. P. Hratchian, J. V. Ortiz, A. F. Izmaylov, J. L. Sonnenberg, D. Williams-Young, F. Ding, F. Lipparini, F. Egidi, J. Goings, B. Peng, A. Petrone, T. Henderson, D. Ranasinghe, V. G. Zakrzewski, J. Gao, N. Rega, G. Zheng, W. Liang, M. Hada, M. Ehara, K. Toyota, R. Fukuda, J. Hasegawa, M. Ishida, T. Nakajima, Y. Honda, O. Kitao, H. Nakai, T. Vreven, K. Throssell, J. A. Montgomery, Jr., J. E. Peralta, F. Ogliaro, M. J. Bearpark, J. J. Heyd, E. N. Brothers, K. N. Kudin, V. N. Staroverov, T. A. Keith, R. Kobayashi, J. Normand, K. Raghavachari, A. P. Rendell, J. C. Burant, S. S. Iyengar, J. Tomasi, M. Cossi, J. M. Millam, M. Klene, C. Adamo, R. Cammi, J. W. Ochterski, R. L. Martin, K. Morokuma, O. Farkas, J. B. Foresman, and D. J. Fox, Gaussian, Inc., Wallingford CT, 2016.
11. J. D. Chai, M. Head-Gordon, *Phys. Chem. Chem. Phys.* 2008, **10**, 6615.
12. A. N. Bootsma, S. Wheeler, *ChemRxiv.* 2019, Preprint.
13. S. Grimme, *Chem. Eur. J.* 2012, **18**, 9955.
14. A. V. Marenich, C. J. Cramer, D. G. Truhlar, *J. Phys. Chem. B* 2009, **113**, 6378.
15. Spartan ’18, Wavefunction, Inc.

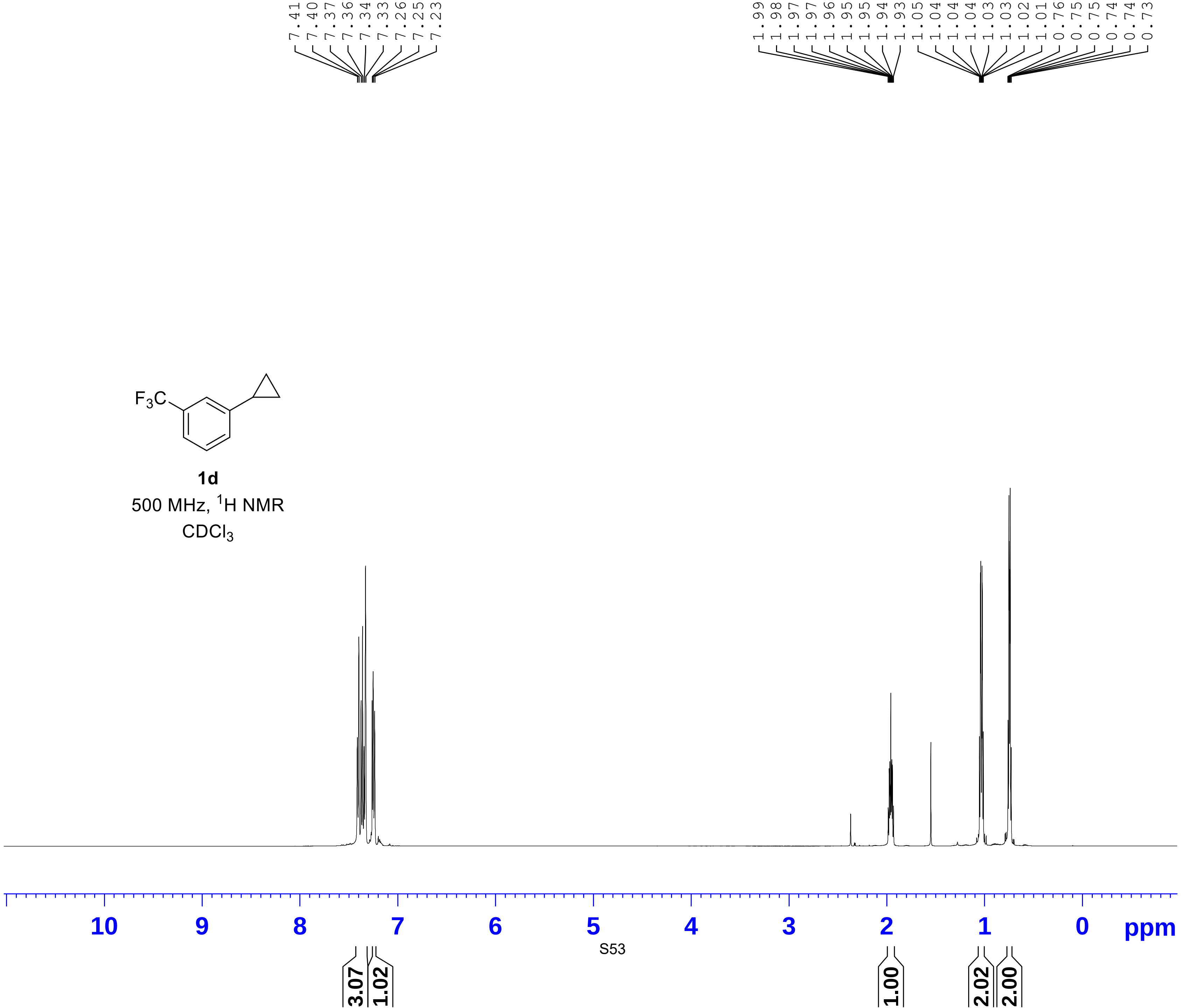

1d

500 MHz, ^{1}H NMR

$CDCl_3$

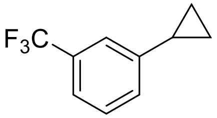

1d

125.7 MHz, ^{13}C NMR

$CDCl_3$

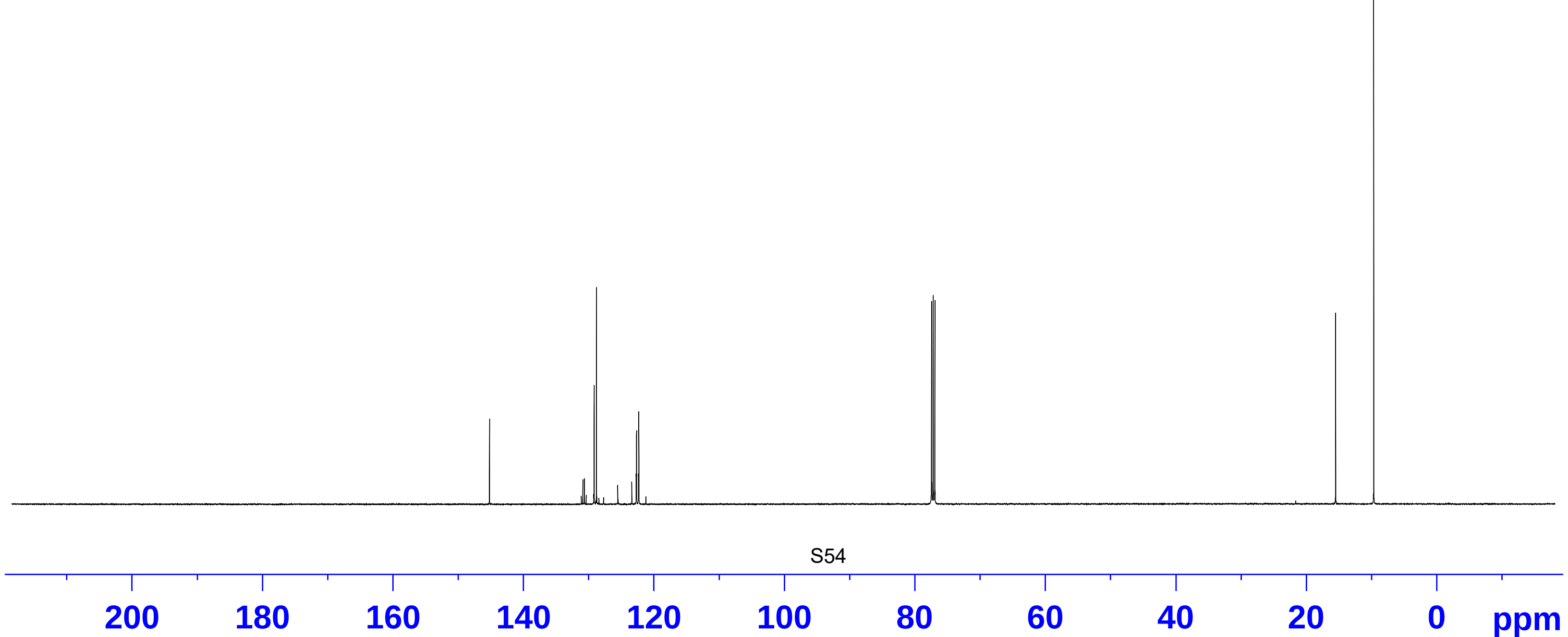

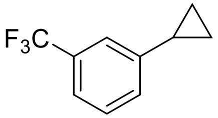

1d
470.6 MHz, ^{19}F NMR
$CDCl_3$

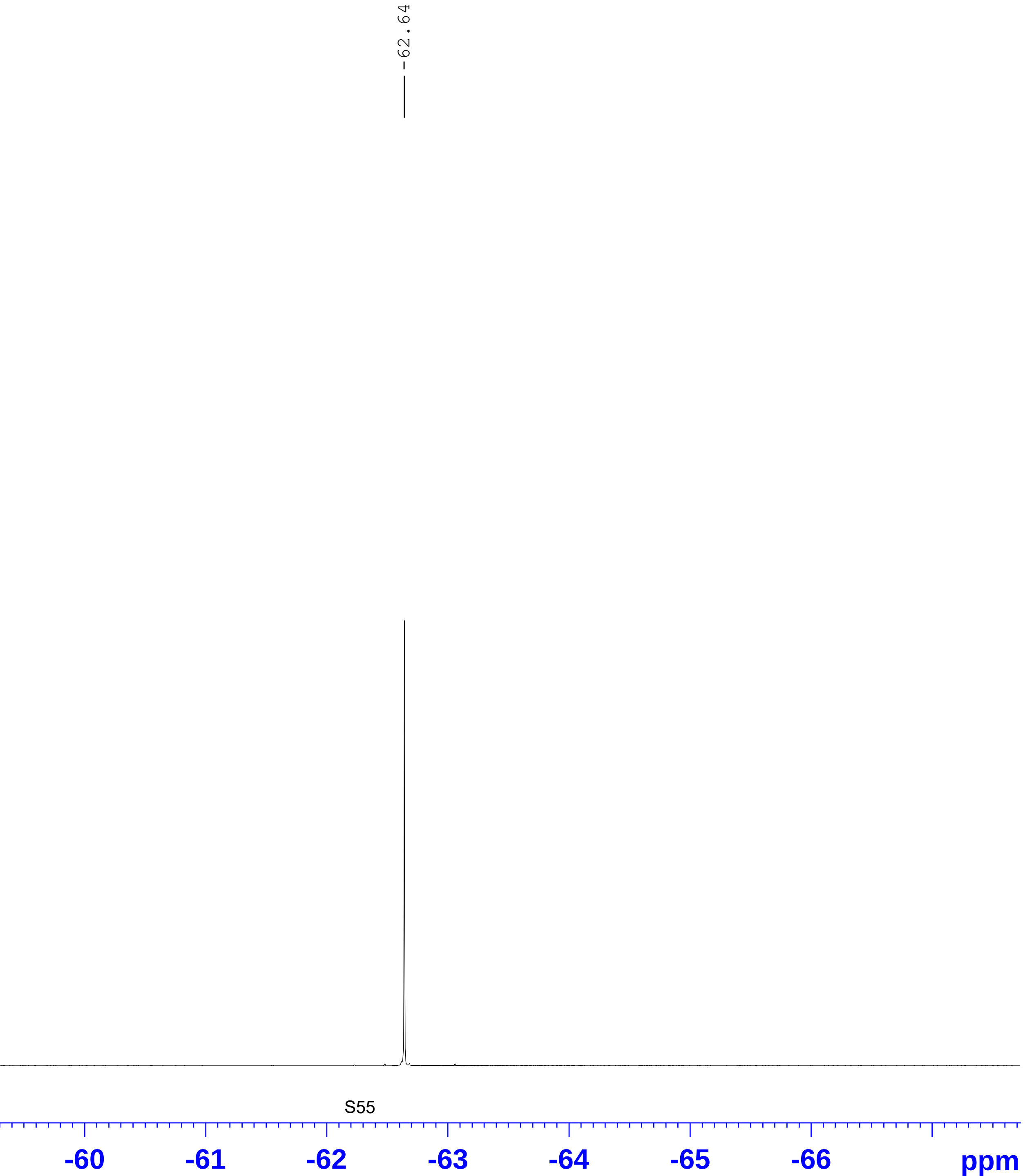

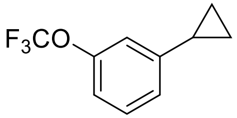

1e

500 MHz, ^{1}H NMR

$CDCl_3$

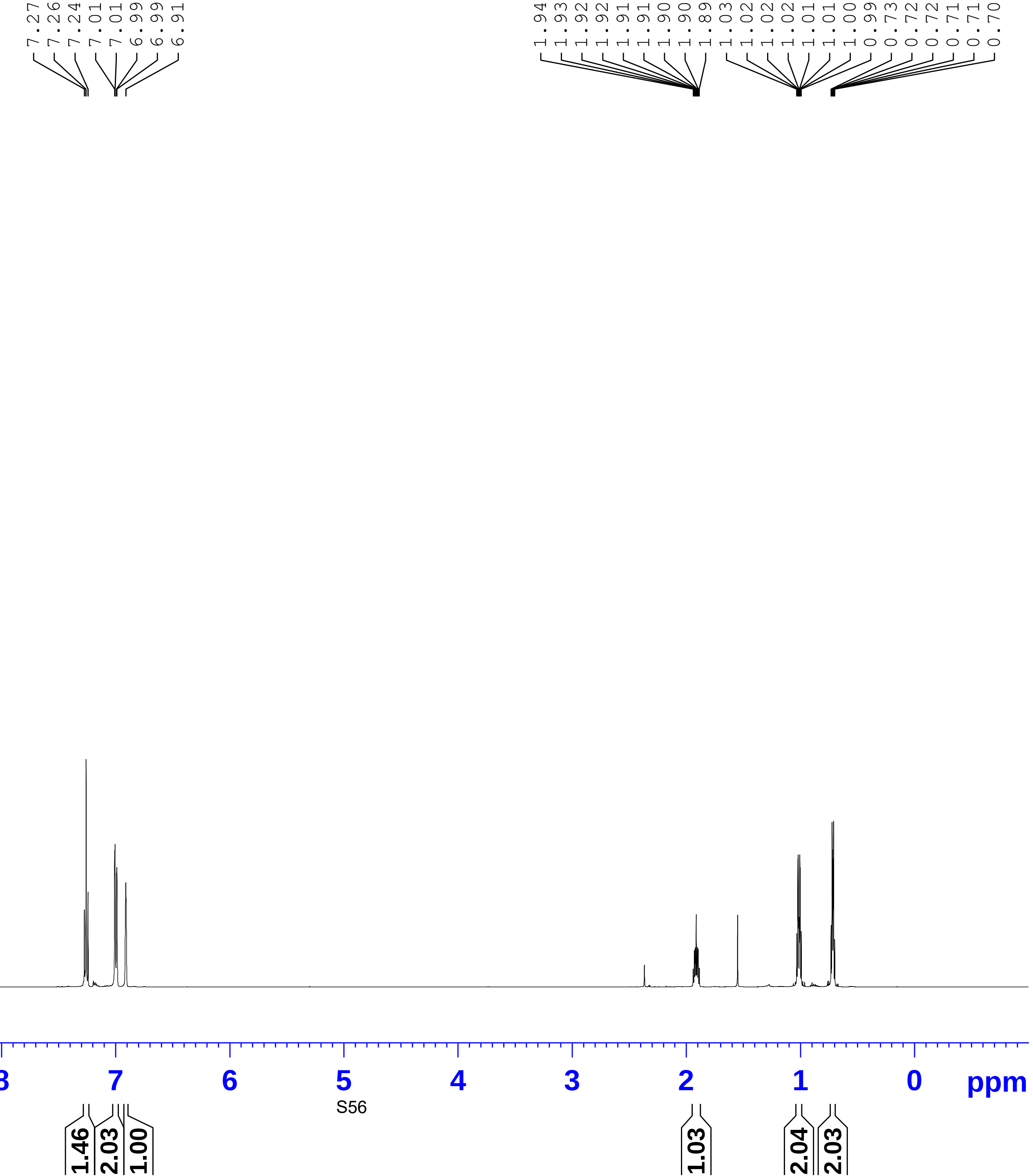

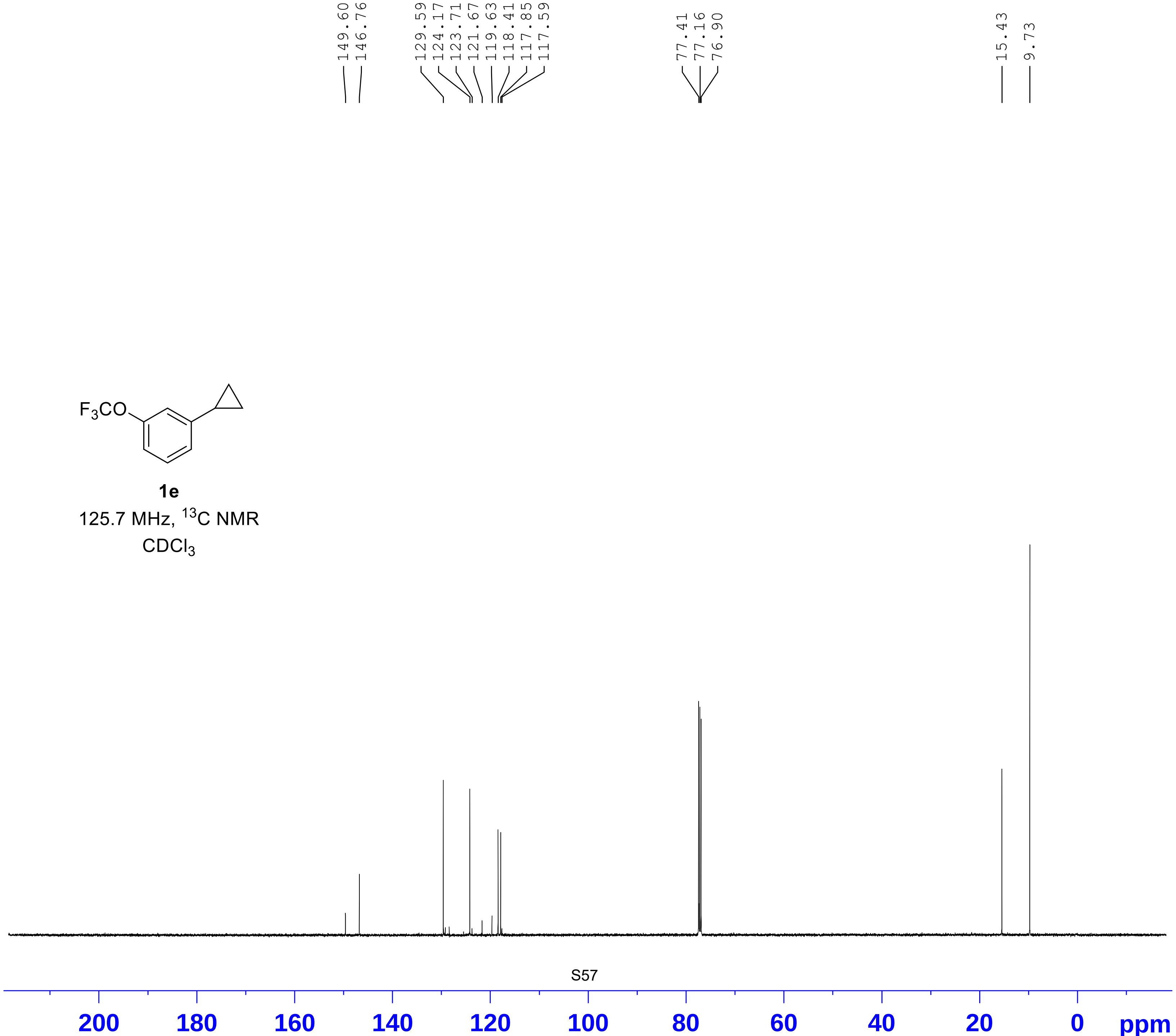

1e

125.7 MHz, ^{13}C NMR

$CDCl_3$

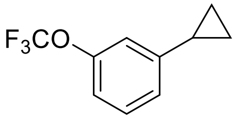

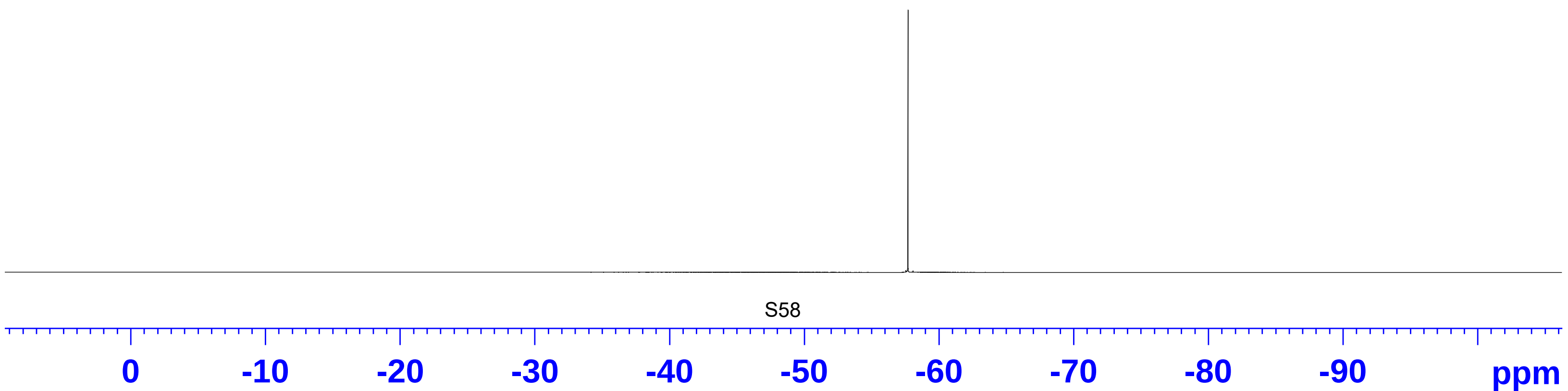

1e

470.6 MHz, ^{19}F NMR

$CDCl_3$

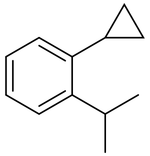

1f
500 MHz, ^{1}H NMR
$CDCl_3$

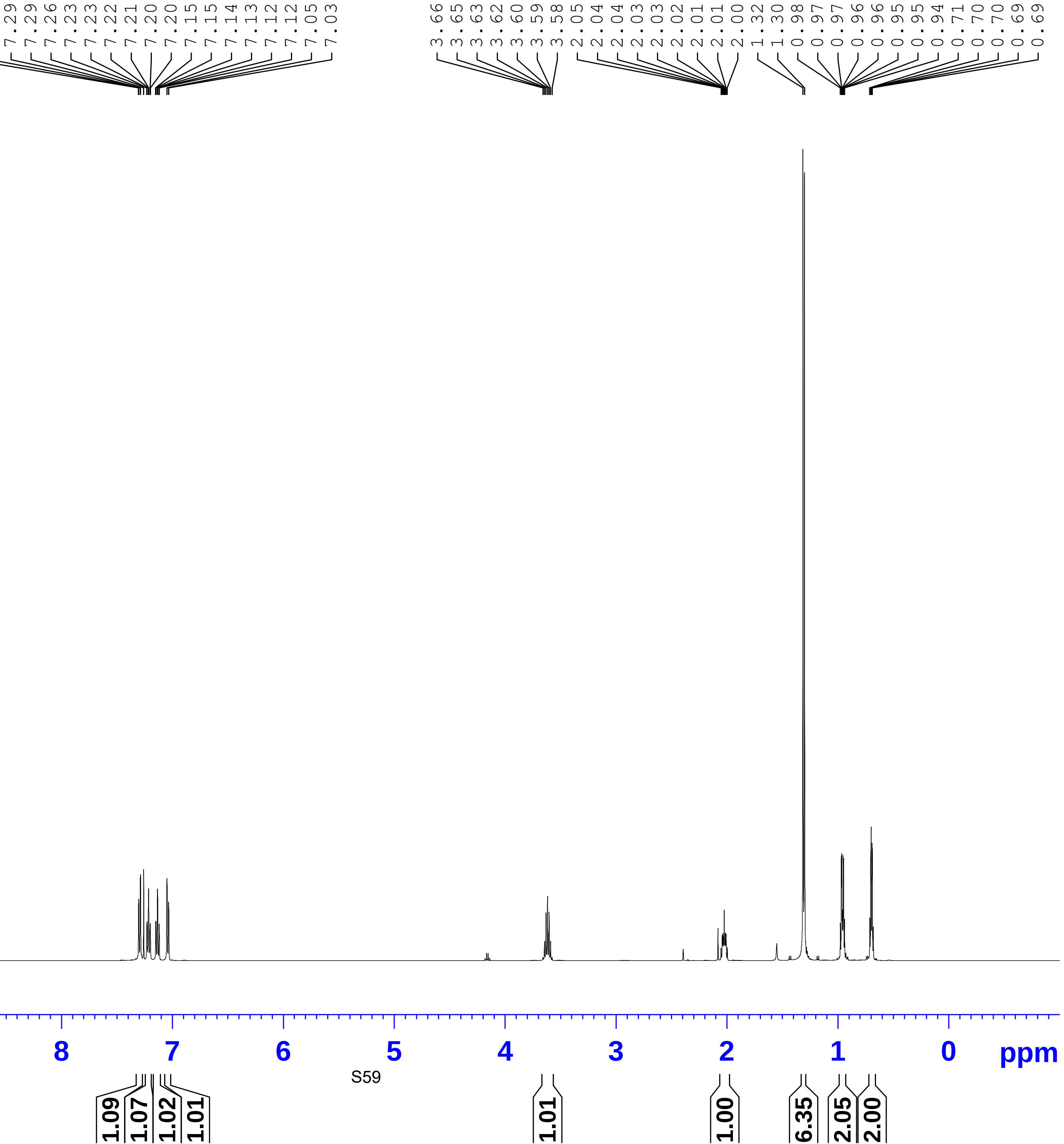

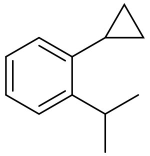

1f

125.7 MHz, ^{13}C NMR

$CDCl_3$

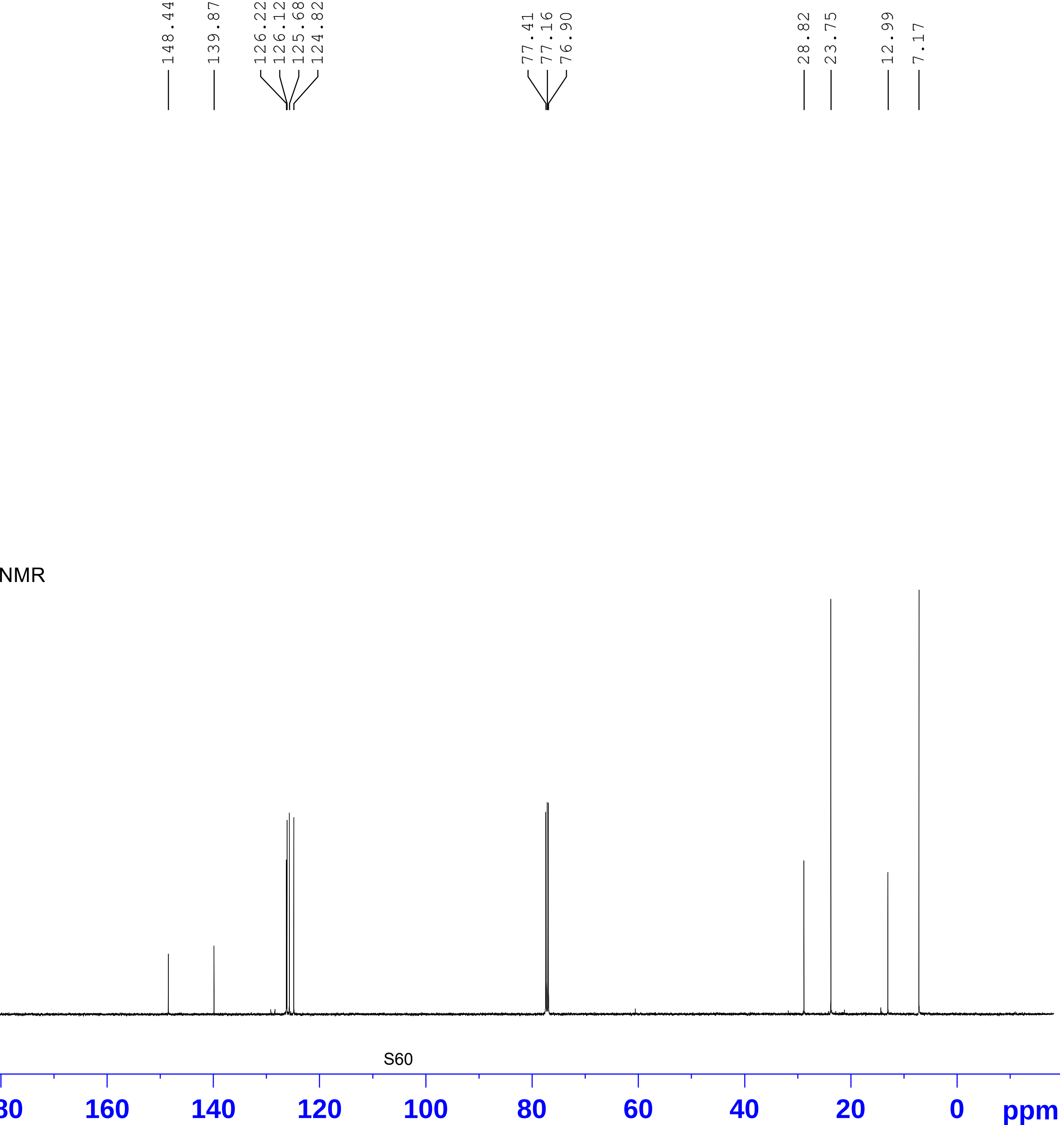

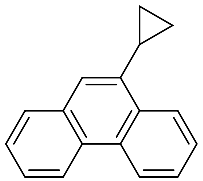

1g

500 MHz, ^{1}H NMR

$CDCl_3$

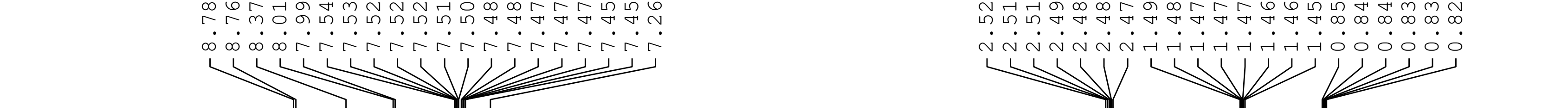

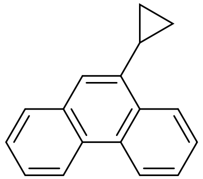

1g
125.7 MHz, ^{13}C NMR
$CDCl_3$

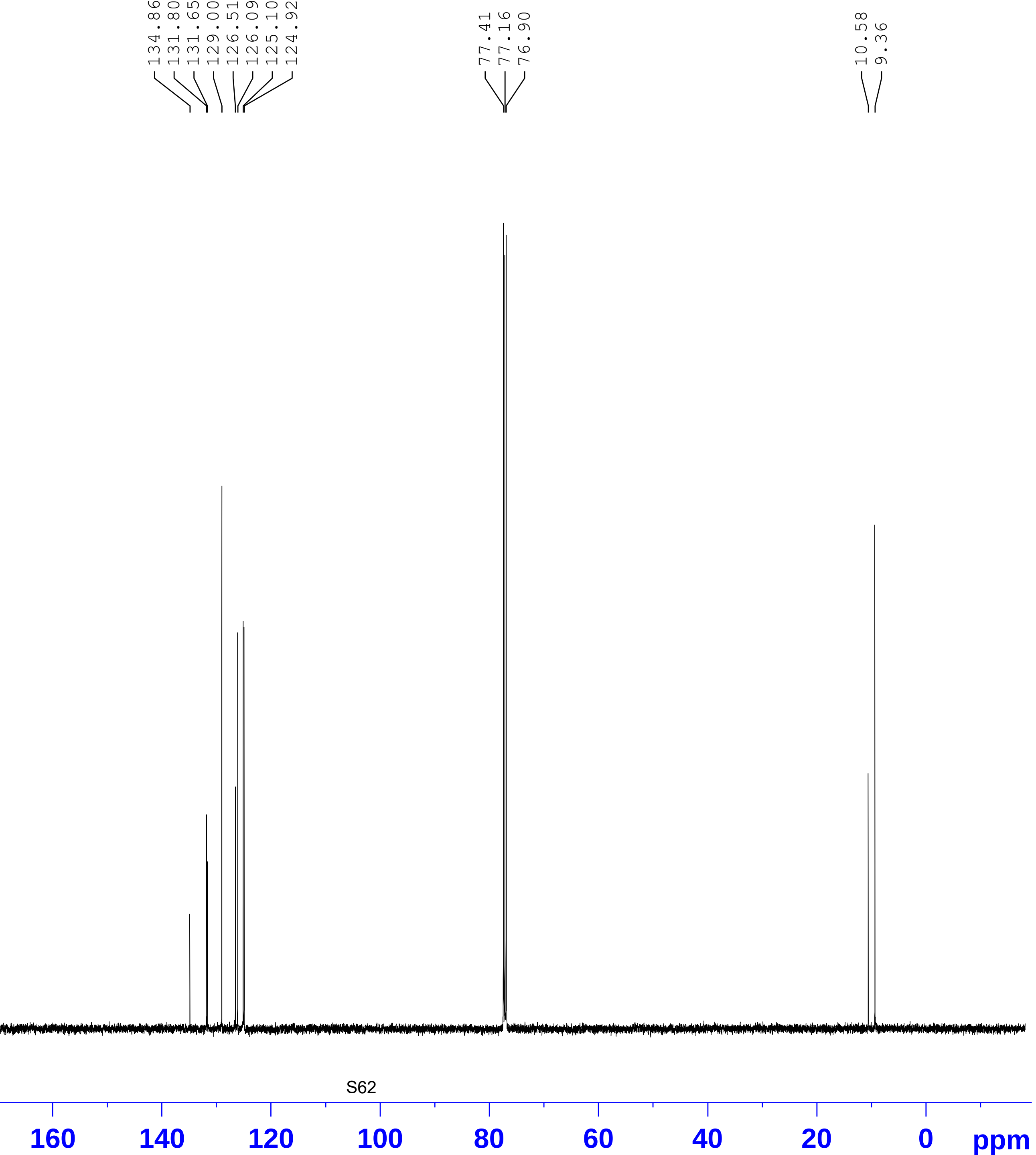

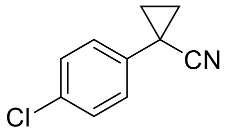

1j
500 MHz, ^{1}H NMR
$CDCl_3$

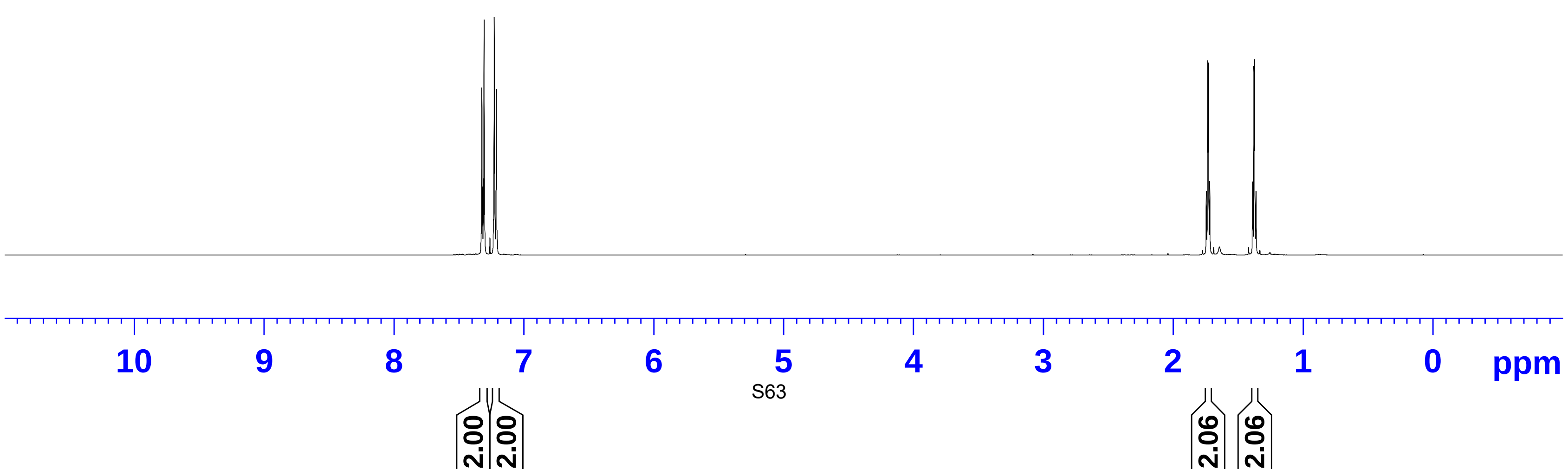

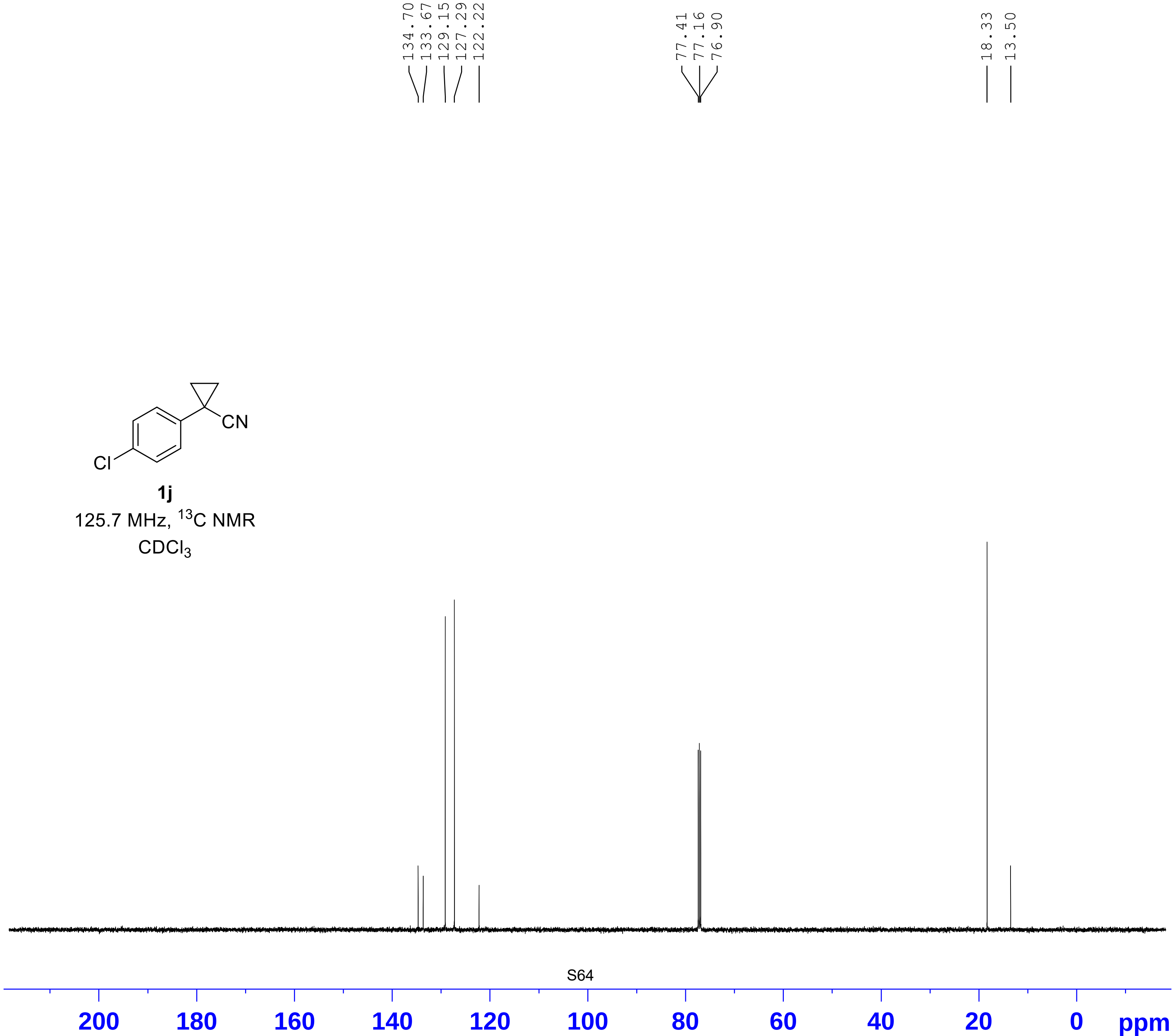
134.70
133.67
129.15
127.29
122.22
77.41
77.16
76.90
18.33
13.50
CN
Cl
1j
125.7 MHz, 13C NMR
CDCl3
200
180
160
140
120
100
80
60
40
20
0
ppm

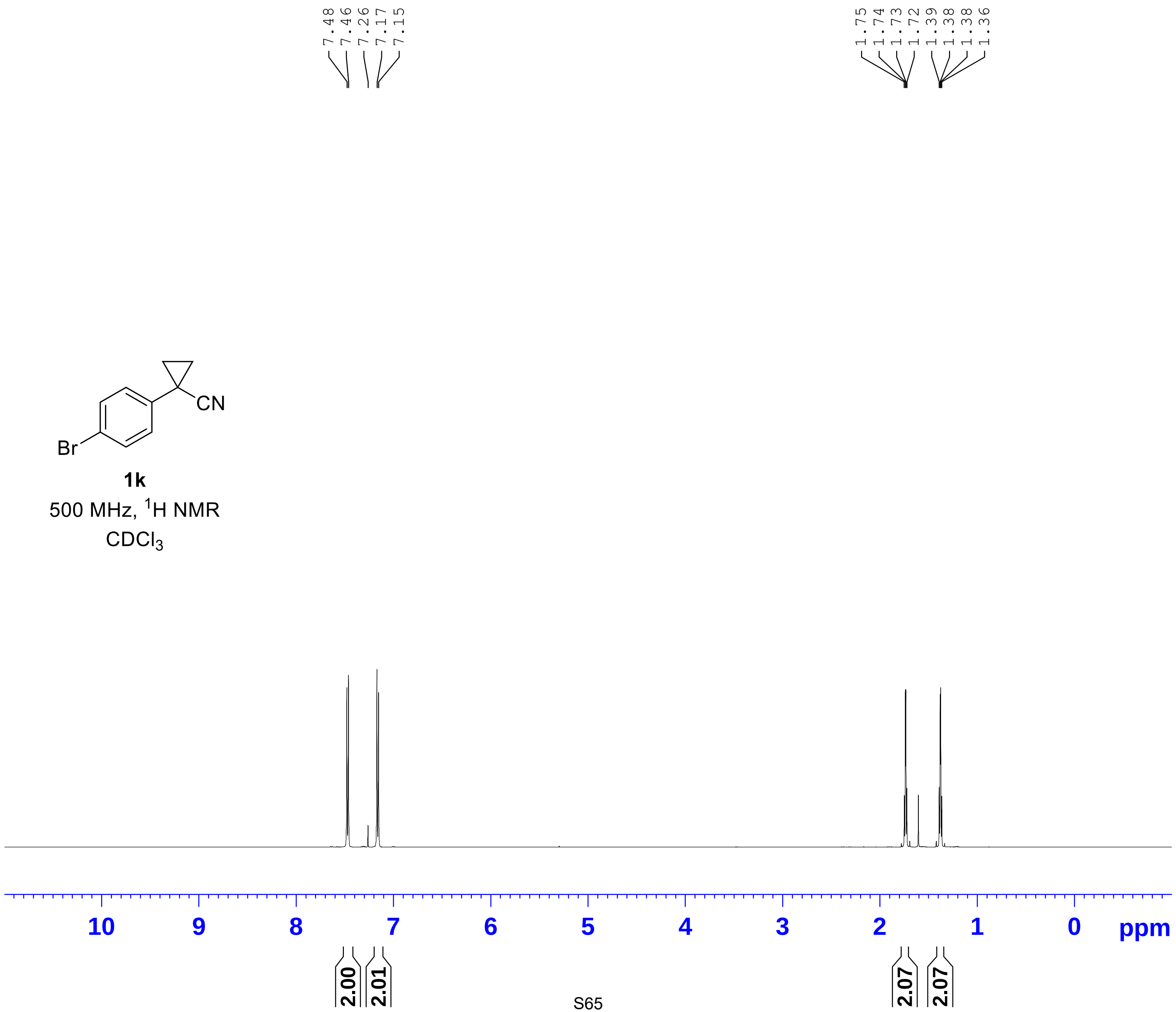

1k
500 MHz, ¹H NMR
$CDCl_3$

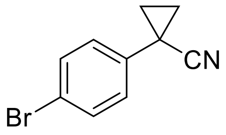

1k
125.7 MHz, ^{13}C NMR
$CDCl_3$

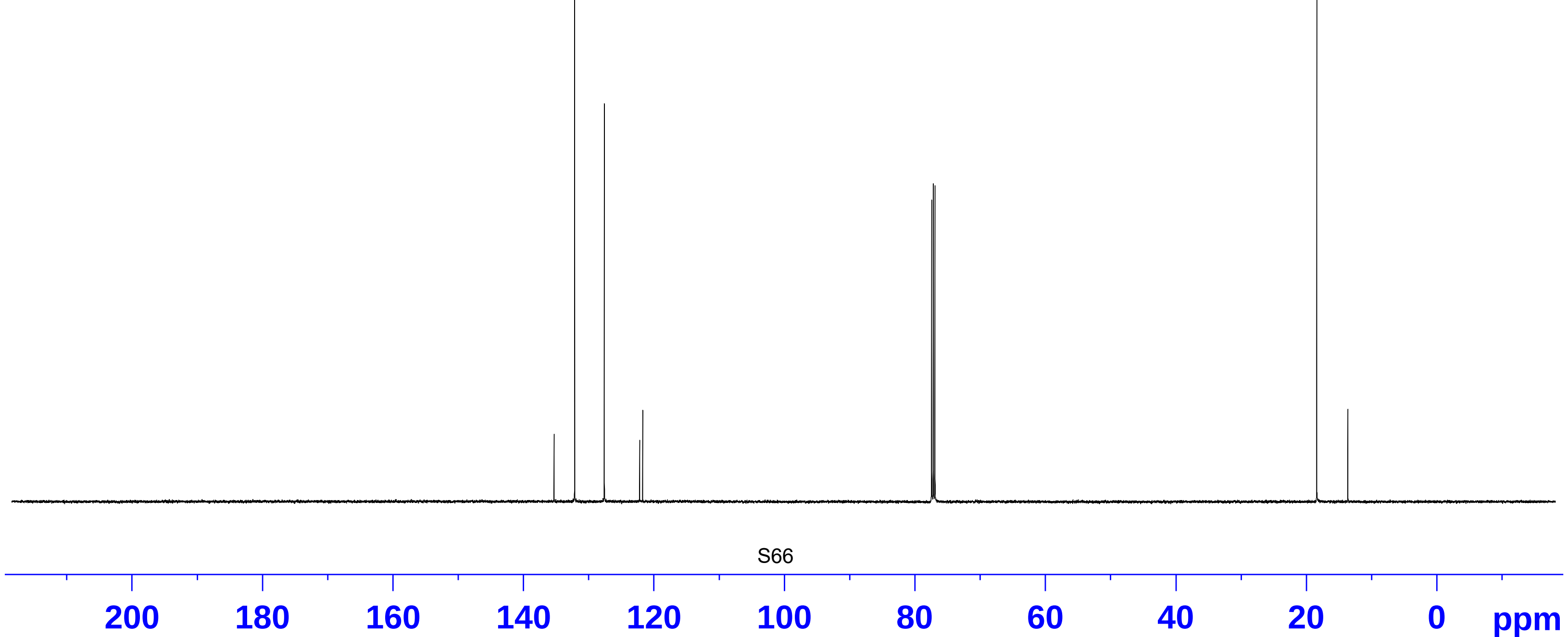

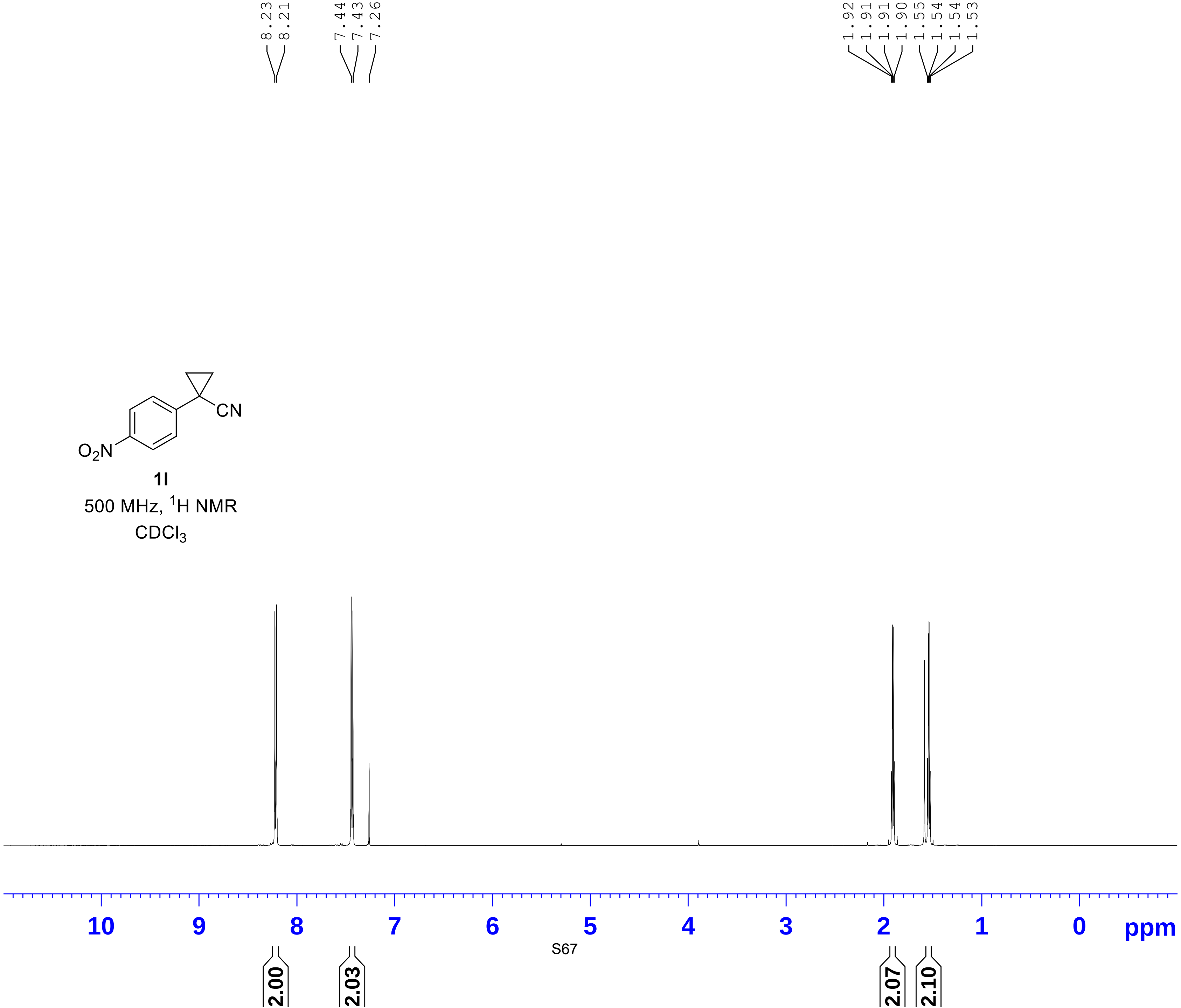
8.23
8.21
7.44
7.43
7.26
1.92
1.91
1.91
1.90
1.55
1.54
1.54
1.53
CN
O_2N
1l
500 MHz, ^{1}H NMR
$CDCl_3$
10
9
8
7
6
5
4
3
2
1
0
ppm
2.00
2.03
2.07
2.10

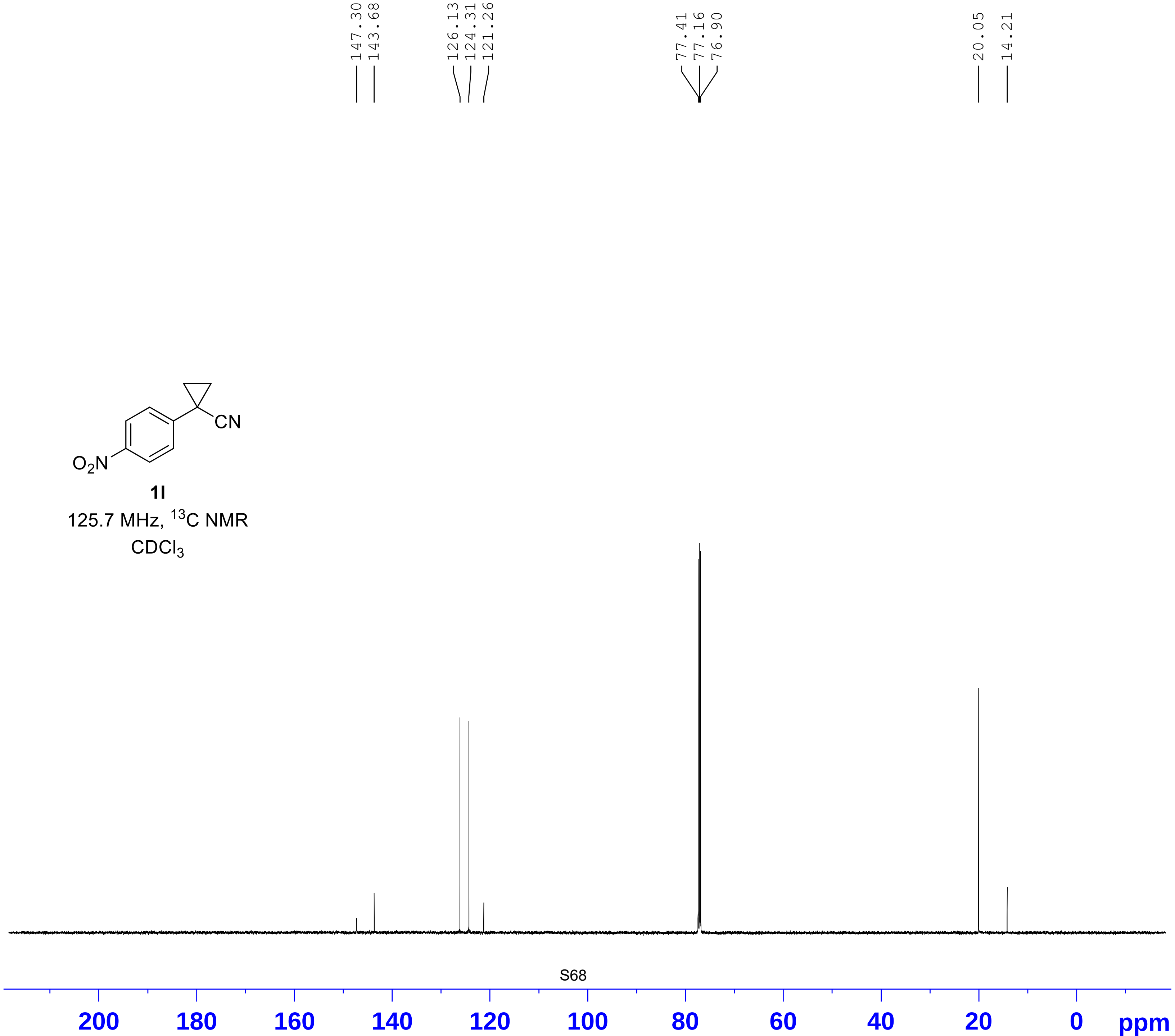
147.30
143.68
126.13
124.31
121.26
77.41
77.16
76.90
20.05
14.21
CN
O2N
1l
125.7 MHz, 13C NMR
CDCl3
200
180
160
140
120
100
80
60
40
20
0
ppm

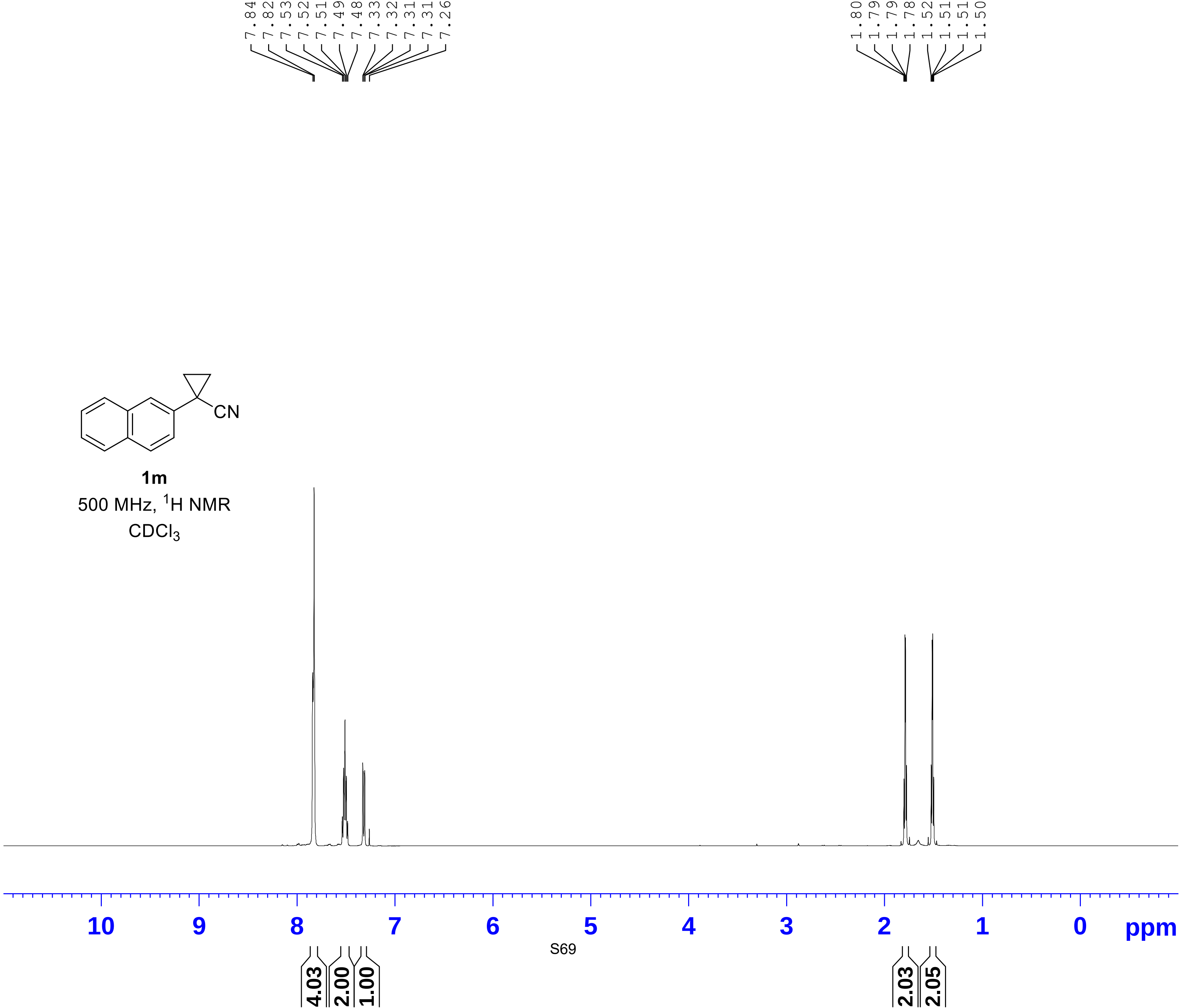
7.84
7.82
7.53
7.52
7.51
7.49
7.48
7.33
7.32
7.31
7.31
7.26
1.80
1.79
1.79
1.78
1.52
1.51
1.51
1.50
CN
1m
500 MHz, ^{1}H NMR
$CDCl_3$
10
9
8
7
6
5
4
3
2
1
0
ppm
4.03
2.00
1.00
2.03
2.05

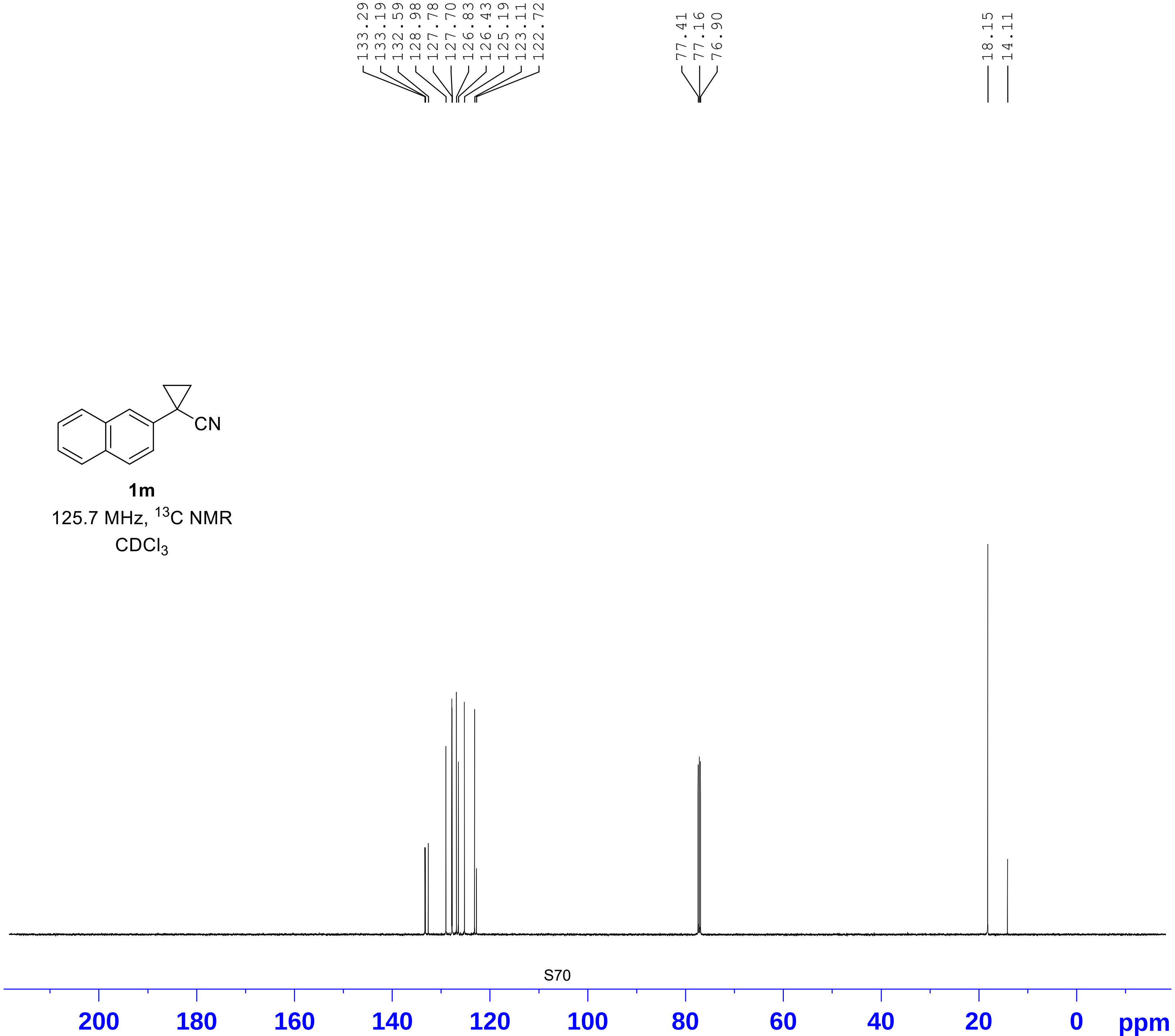

1m

125.7 MHz, ^{13}C NMR

$CDCl_3$

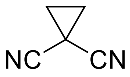

1n
400 MHz, ^{1}H NMR
$CDCl_3$

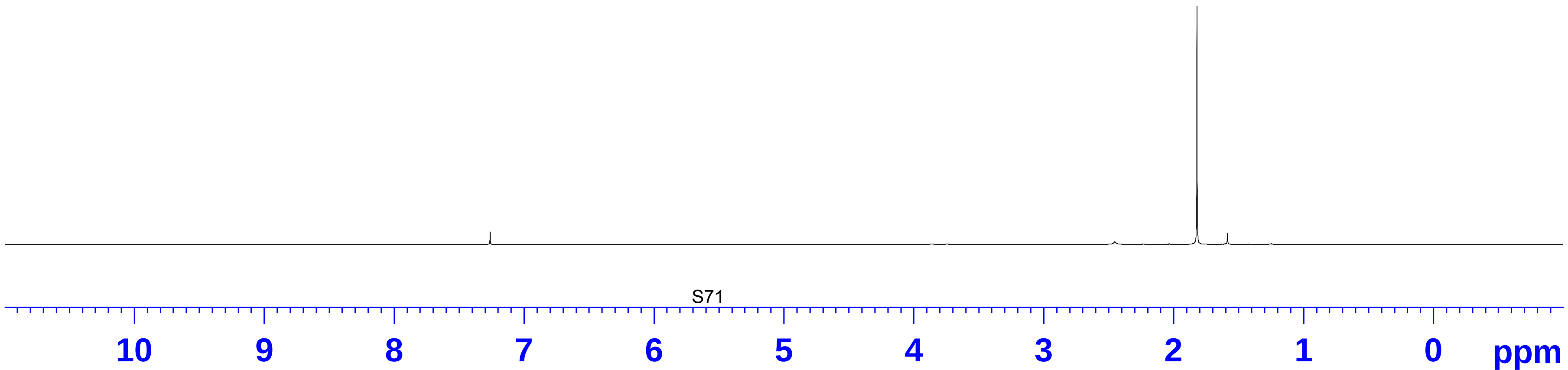

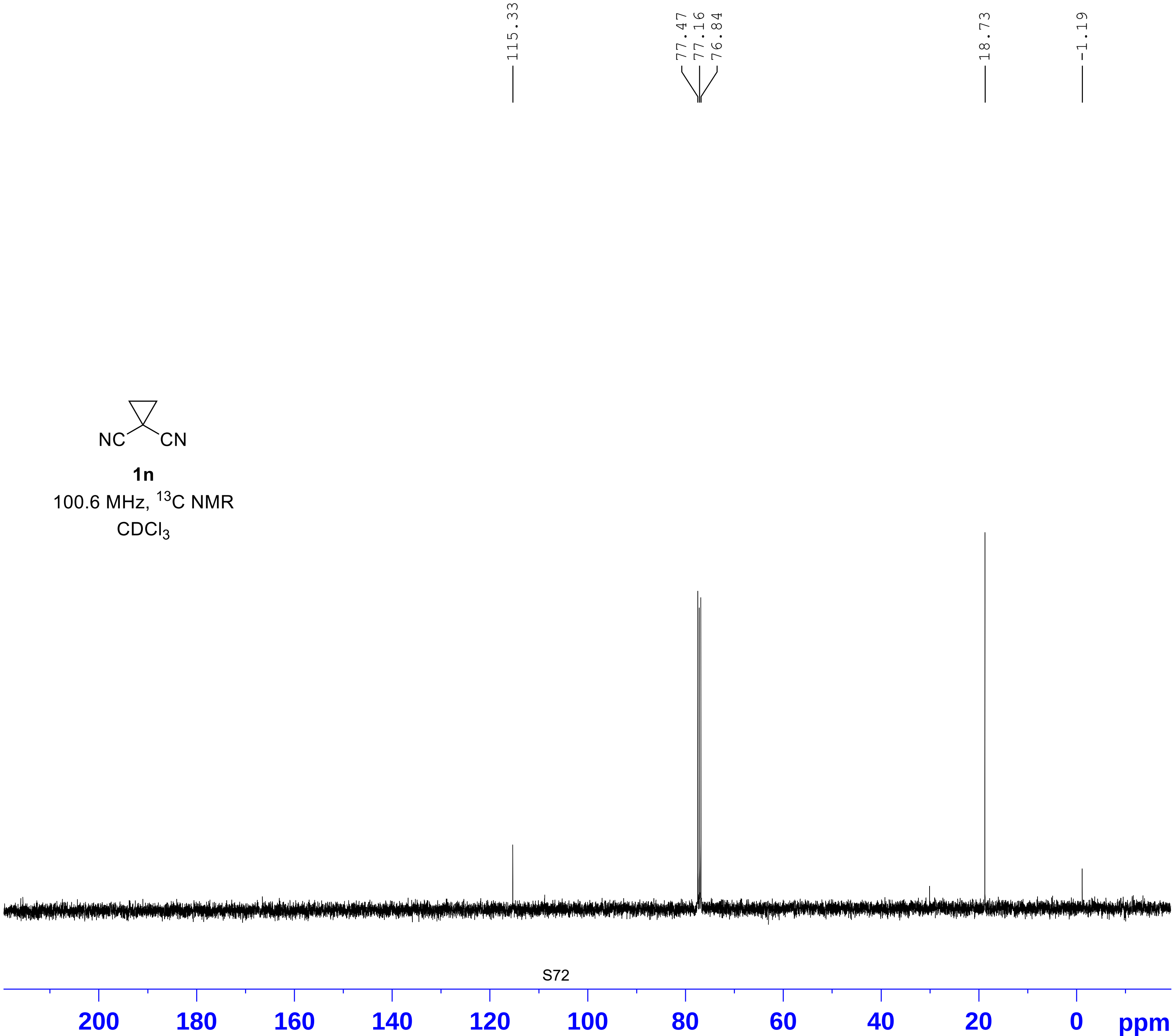

NC
CN
1n
100.6 MHz, ^{13}C NMR
$CDCl_3$
115.33
77.47
77.16
76.84
18.73
-1.19
200
180
160
140
120
100
80
60
40
20
0
ppm

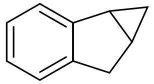

1s
400 MHz, ^{1}H NMR
$CDCl_3$

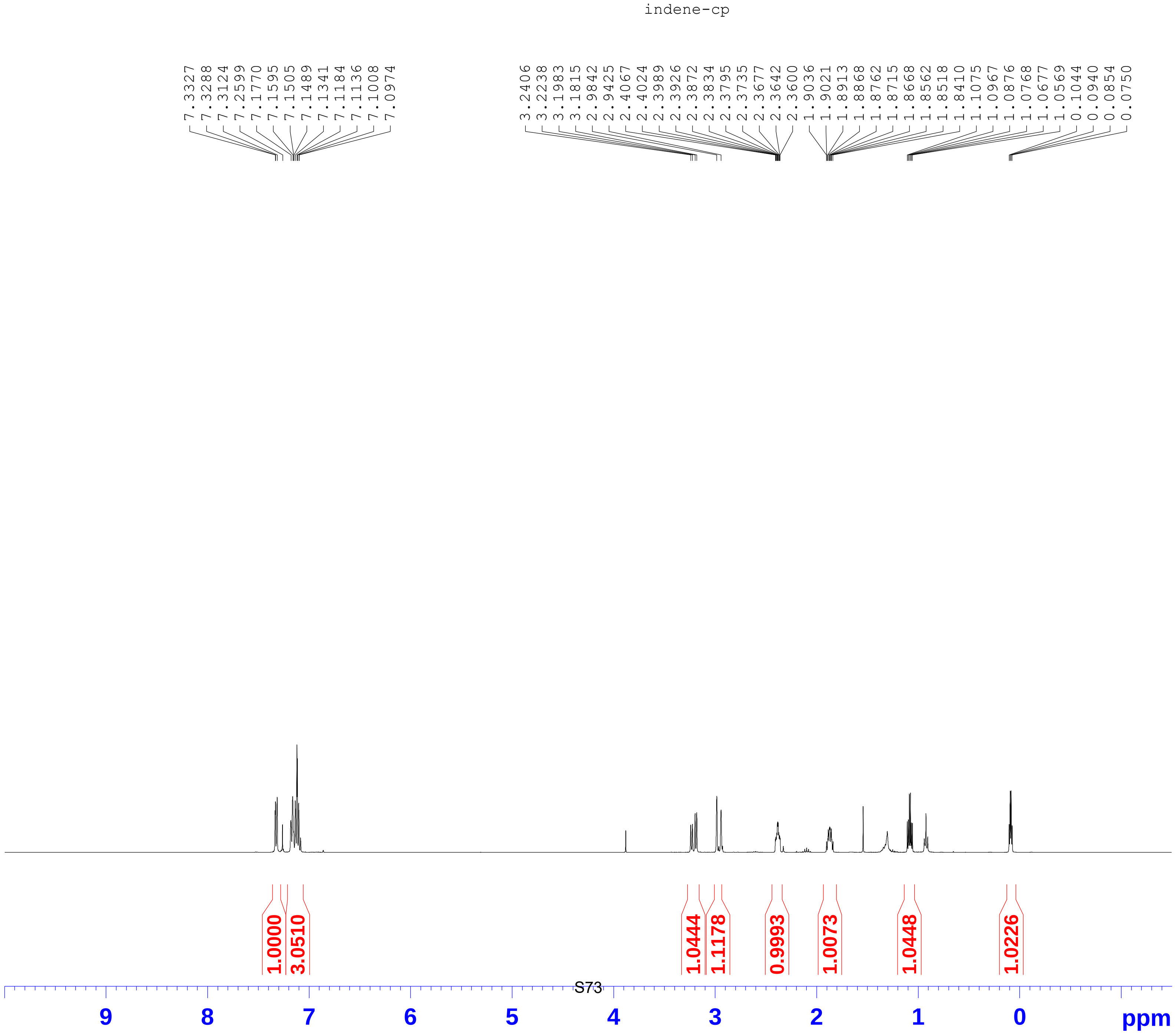

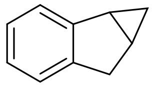

1s
100.6 MHz, ^{13}C NMR
$CDCl_3$

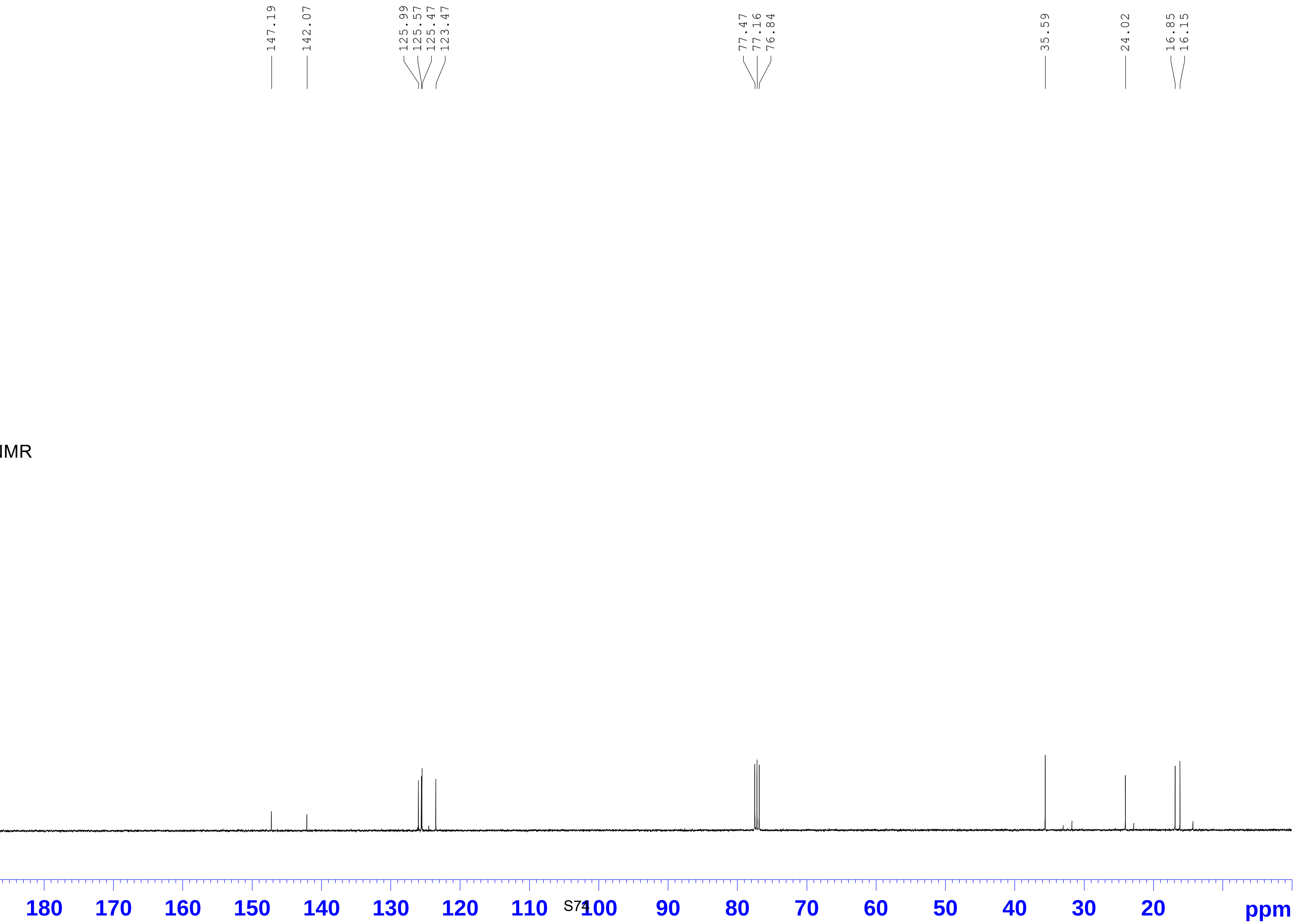

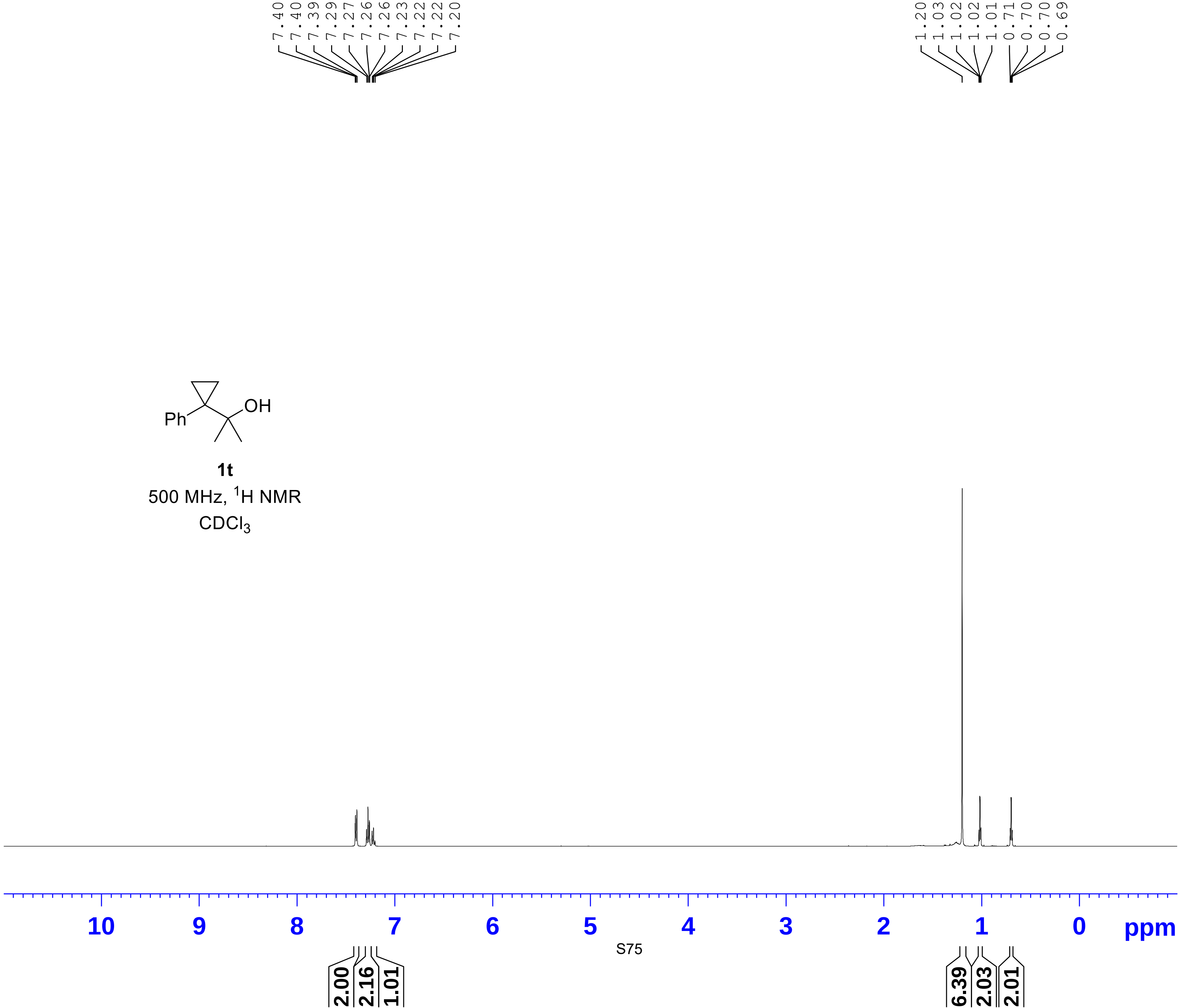
7.40
7.40
7.39
7.29
7.27
7.26
7.26
7.23
7.22
7.22
7.20
1.20
1.03
1.02
1.02
1.01
0.71
0.70
0.70
0.69
Ph
OH
1t
500 MHz, ^{1}H NMR
$CDCl_3$
10
9
8
7
6
5
4
3
2
1
0
ppm
2.00
2.16
1.01
6.39
2.03
2.01

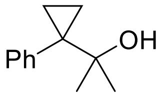

1t
125.7 MHz, ^{13}C NMR
$CDCl_3$

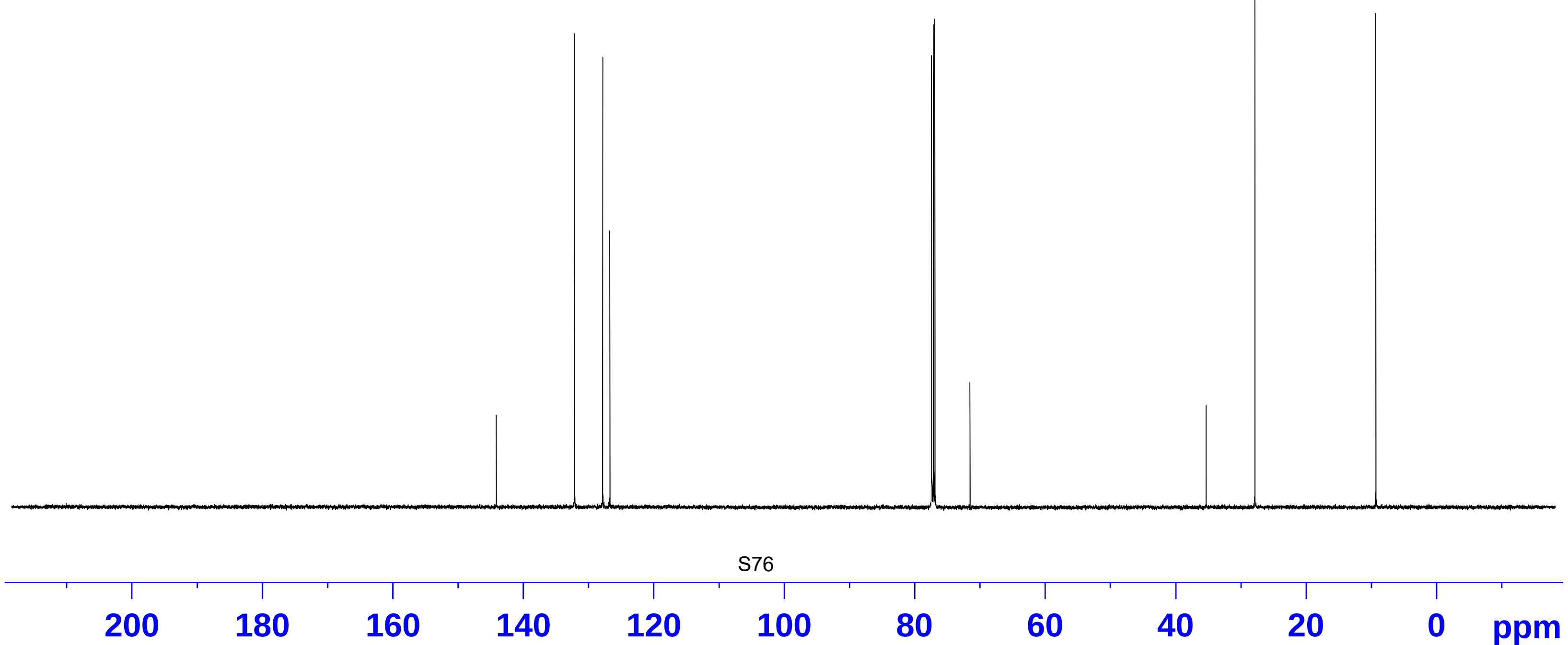

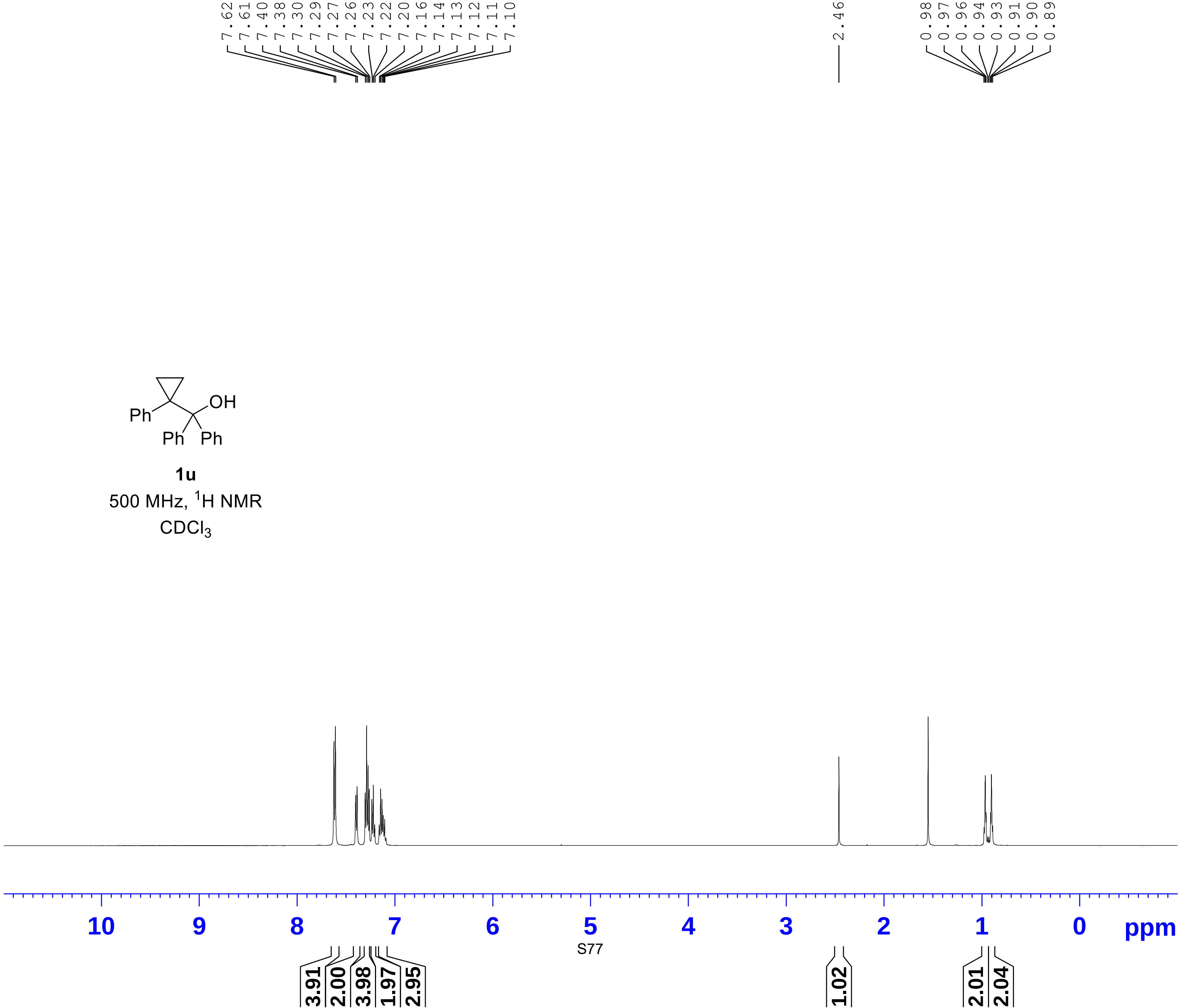
7.62 7.61 7.40 7.38 7.30 7.29 7.27 7.26 7.23 7.22 7.20 7.16 7.14 7.13 7.12 7.11 7.10
2.46
0.98 0.97 0.96 0.94 0.93 0.91 0.90 0.89
Ph
OH
Ph
Ph
1u
500 MHz, ¹H NMR
CDCl3
10 9 8 7 6 5 4 3 2 1 0 ppm
3.91 2.00 3.98 1.97 2.95
1.02
2.01 2.04

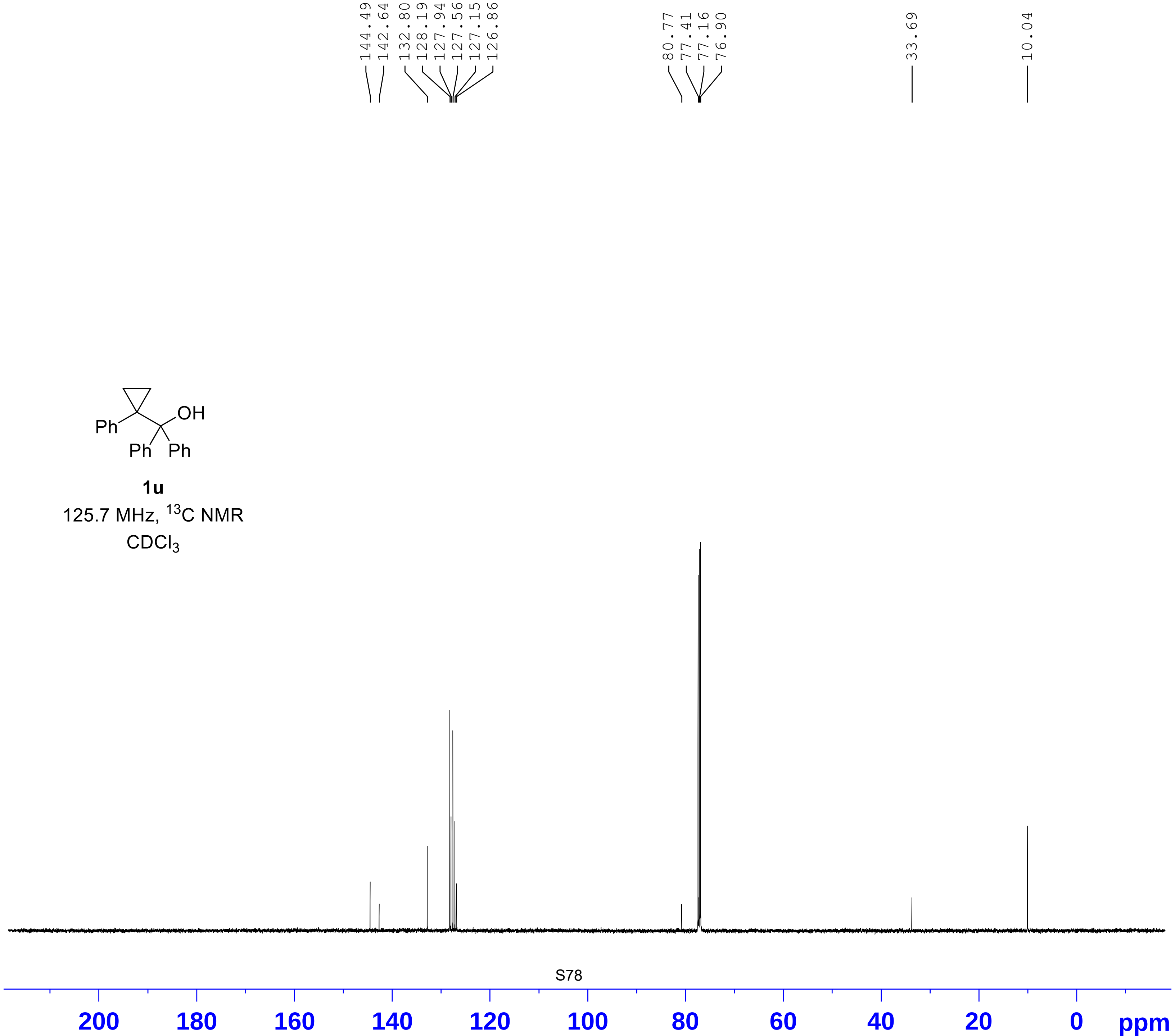
144.49
142.64
132.80
128.19
127.94
127.56
127.15
126.86
80.77
77.41
77.16
76.90
33.69
10.04
Ph
OH
Ph
Ph
1u
125.7 MHz, ^{13}C NMR
$CDCl_3$
200
180
160
140
120
100
80
60
40
20
0
ppm

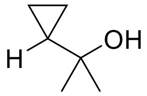

1v
500 MHz, ^{1}H NMR
$CDCl_3$

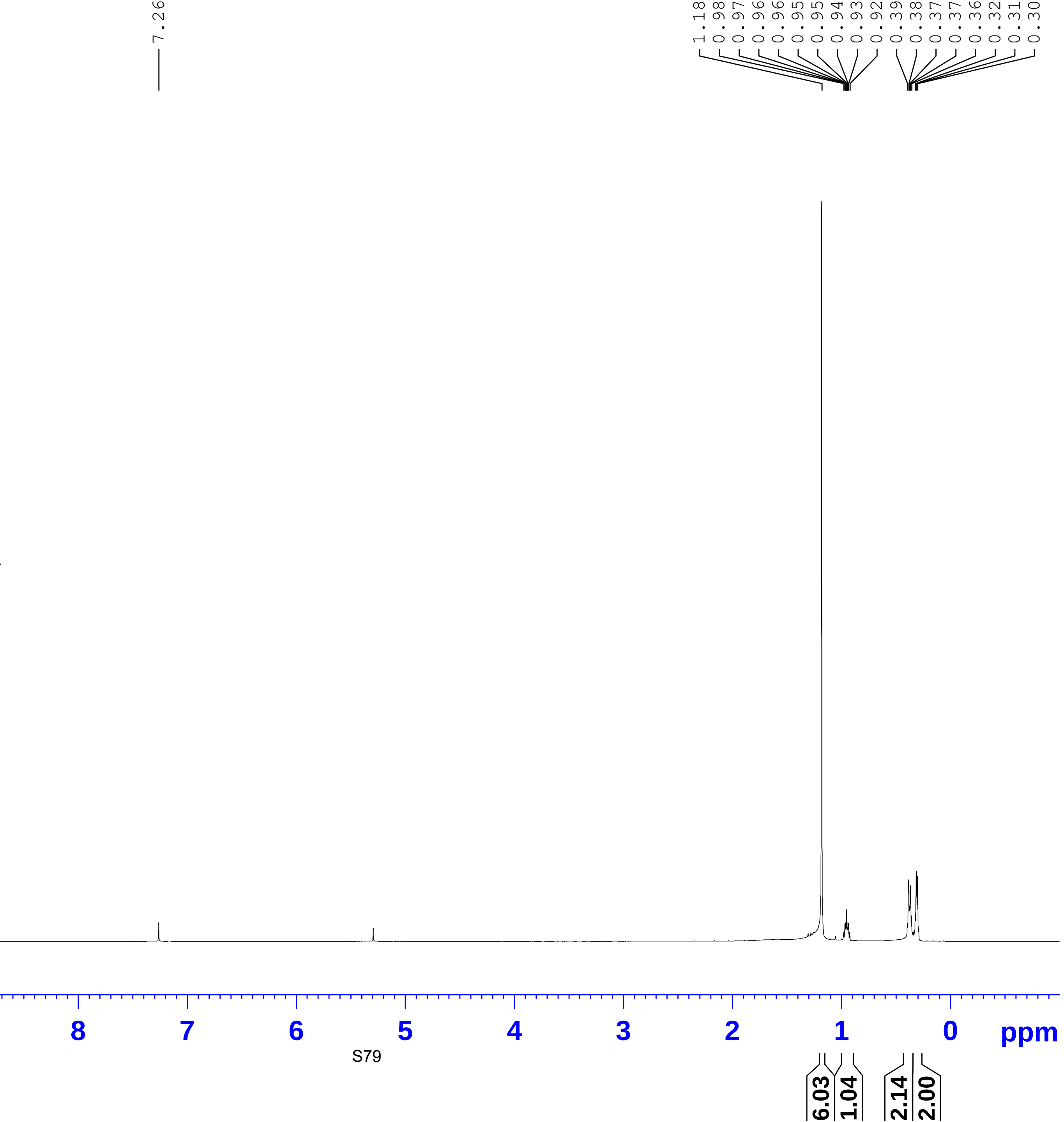

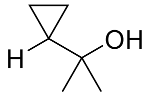

1v
125.7 MHz, ^{13}C NMR
$CDCl_3$

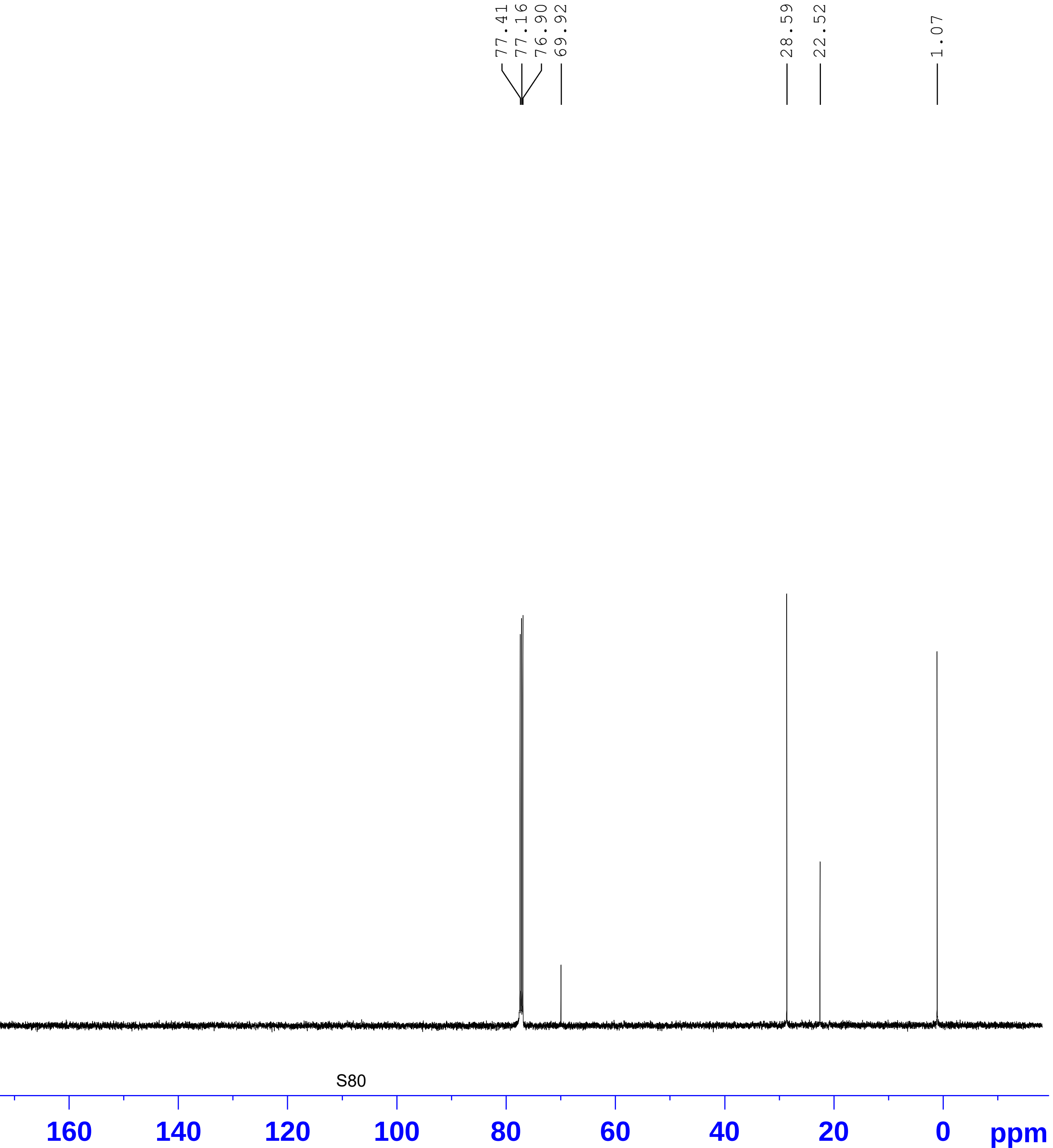

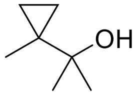

1w
500 MHz, ^{1}H NMR
$CDCl_3$

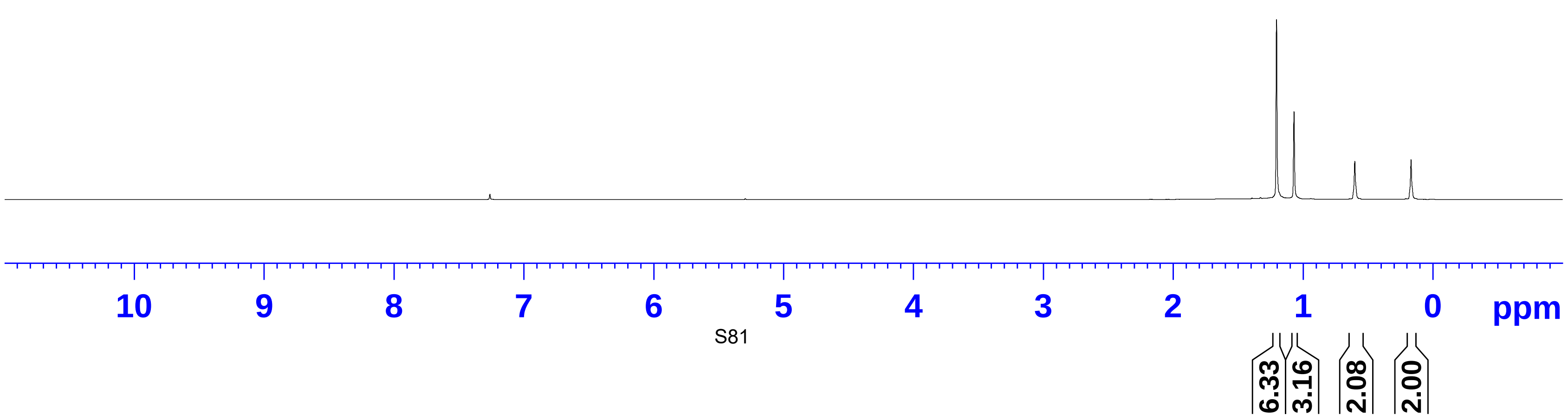

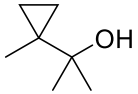

1w
125.7 MHz, ^{13}C NMR
$CDCl_3$

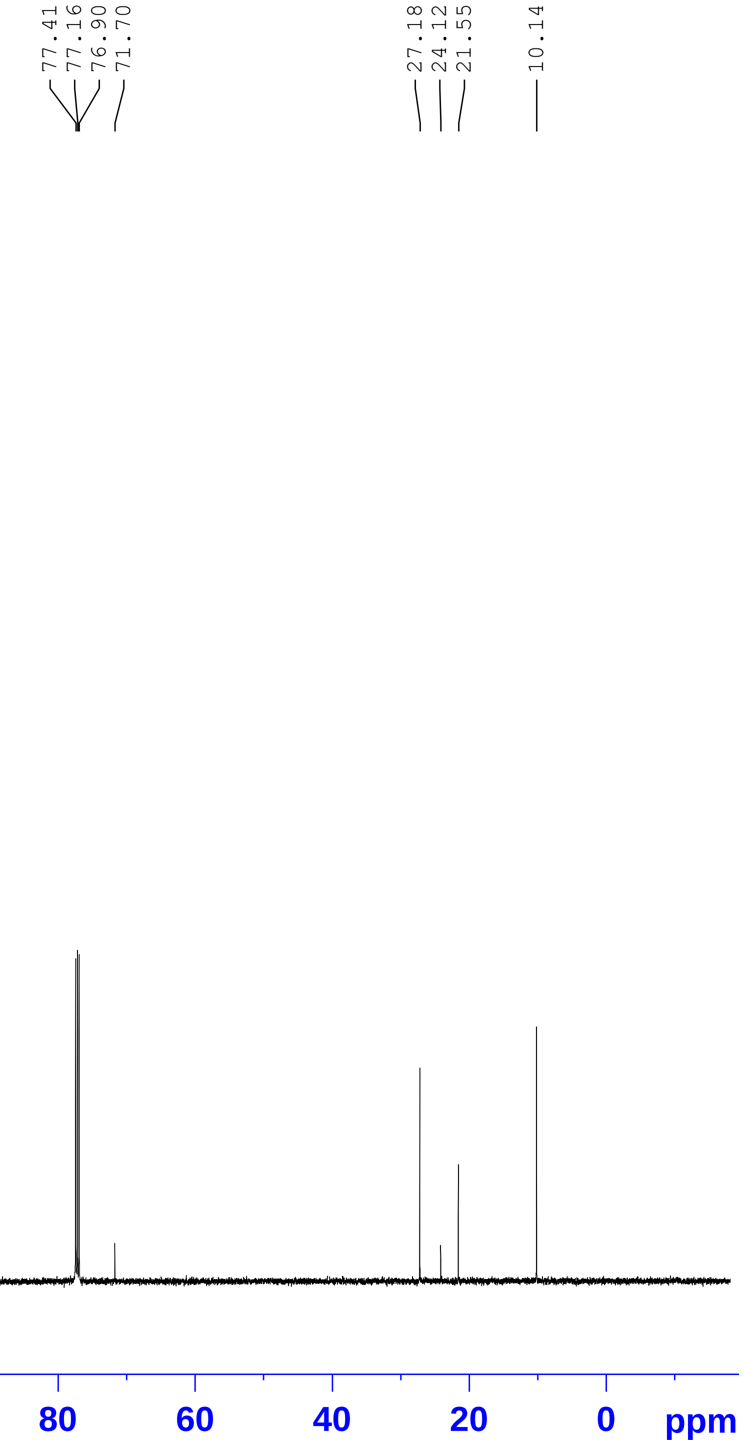

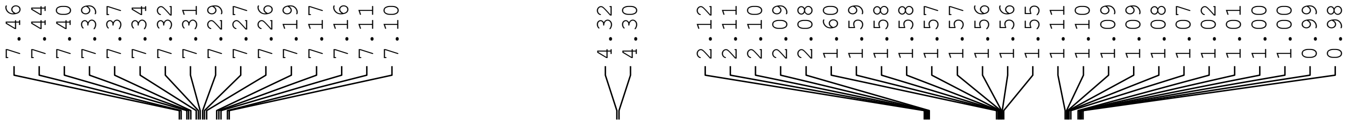

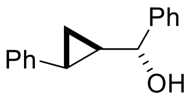

1x
500 MHz, ^{1}H NMR
$CDCl_3$

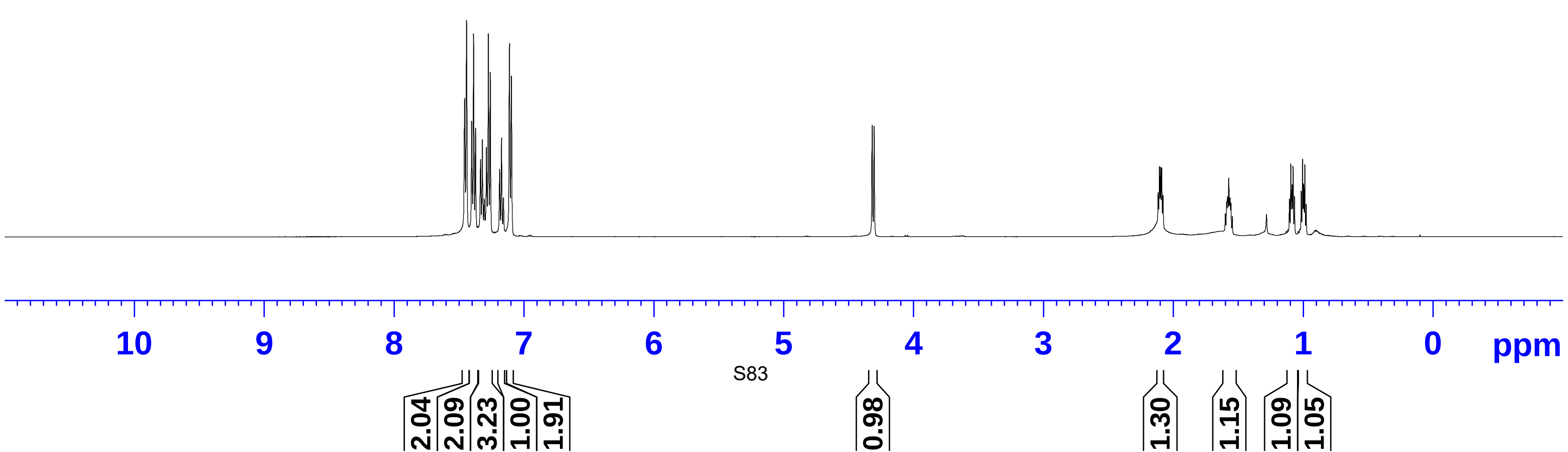

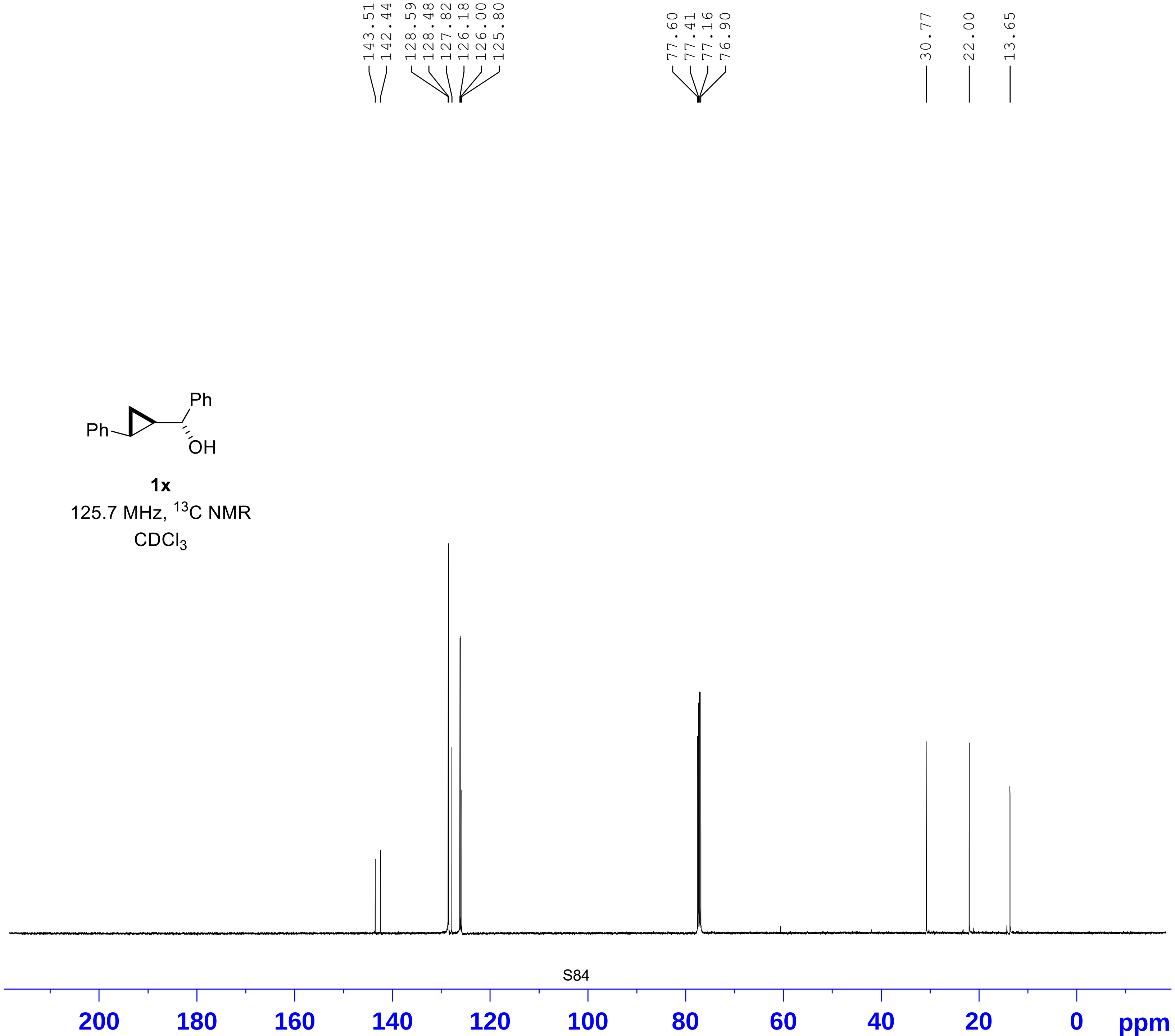
Ph
Ph
OH
1x
125.7 MHz, 13C NMR
CDCl3
143.51
142.44
128.59
128.48
127.82
126.18
126.00
125.80
77.60
77.41
77.16
76.90
30.77
22.00
13.65
200
180
160
140
120
100
80
60
40
20
0
ppm

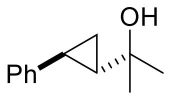

1y
500 MHz, ^{1}H NMR
$CDCl_3$

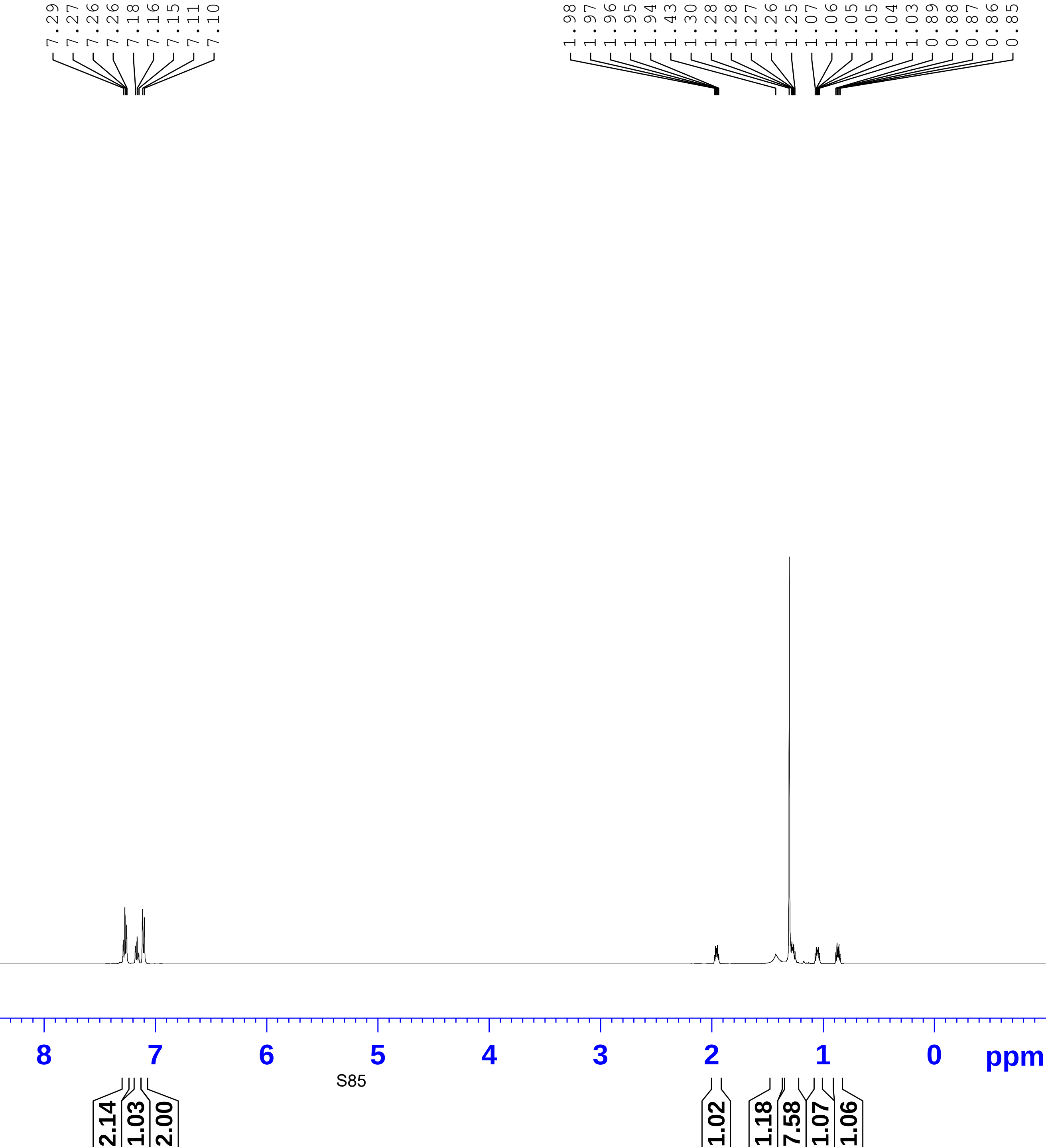

1y

125.7 MHz, ^{13}C NMR

$CDCl_3$

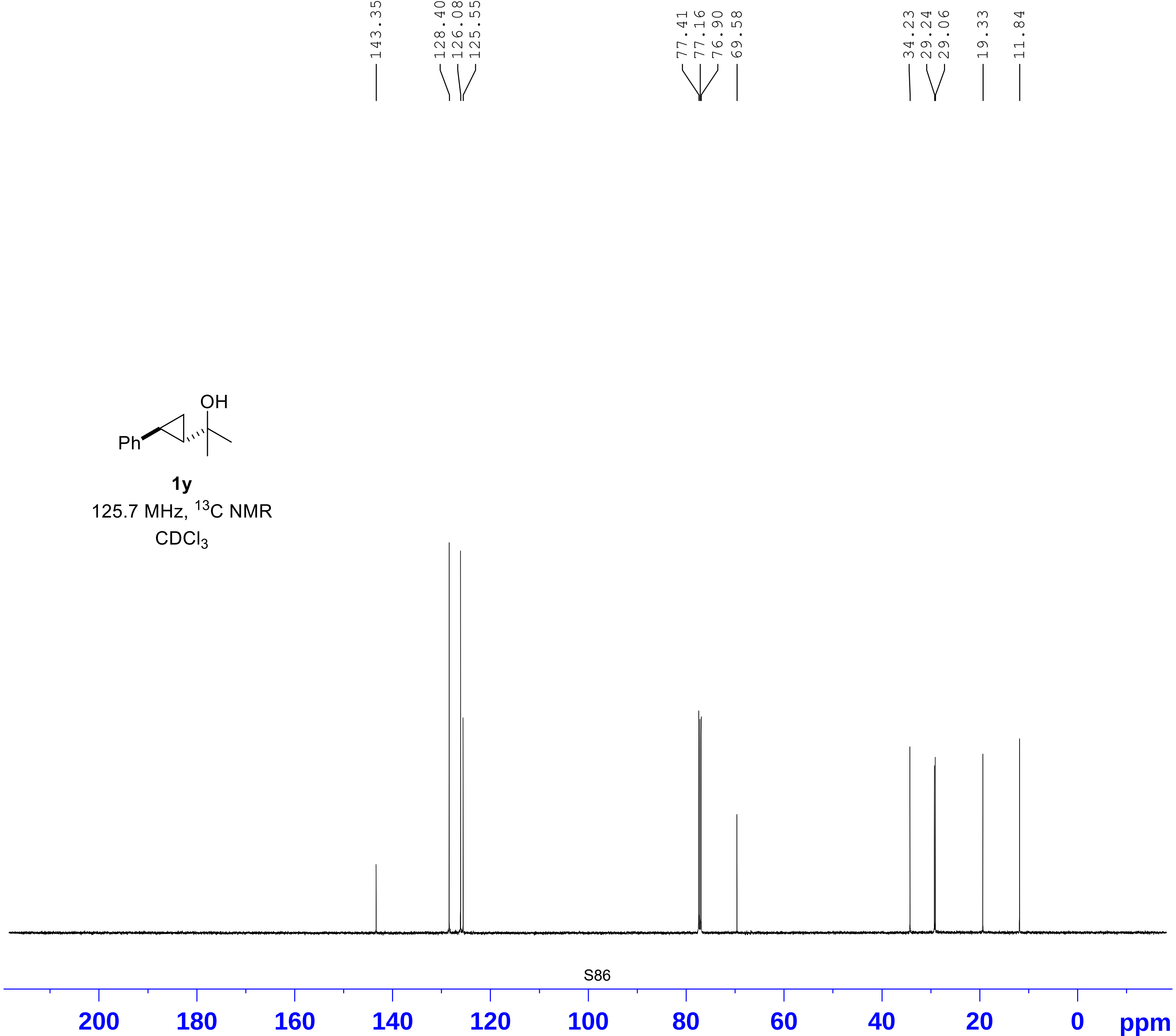

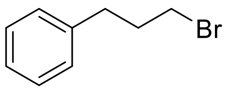

2a
500 MHz, ¹H NMR
$CDCl_3$

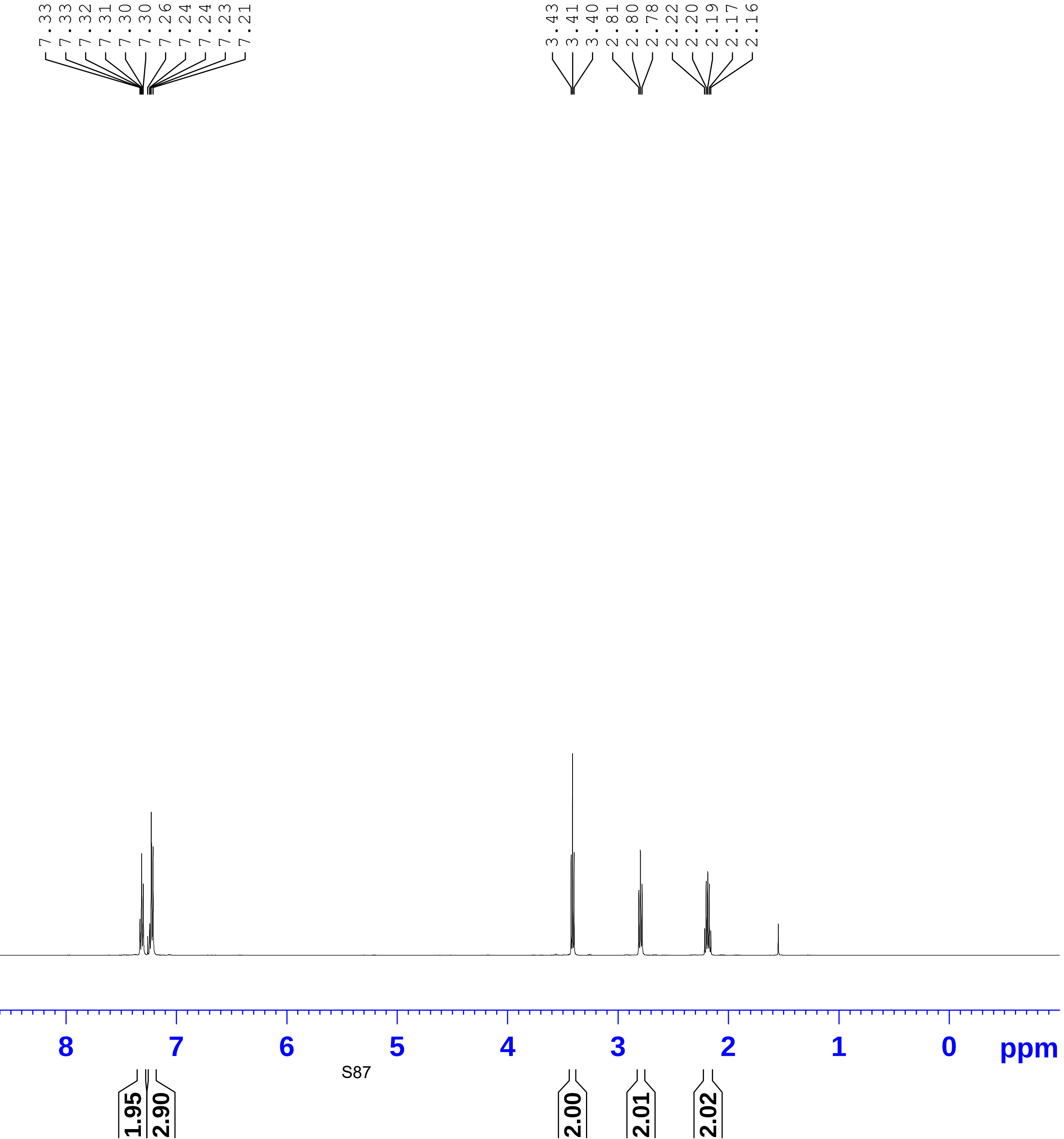

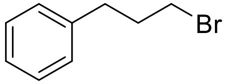

2a

125.7 MHz, ^{13}C NMR

$CDCl_3$

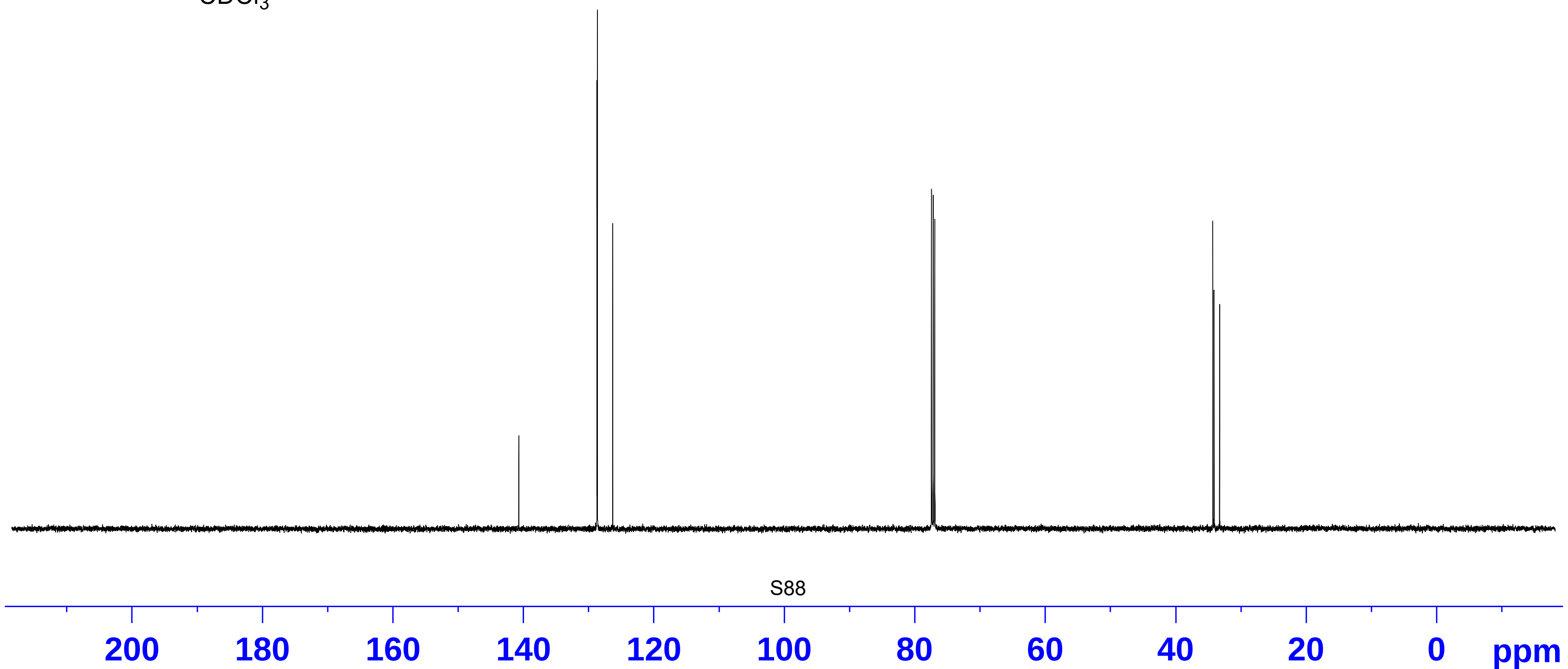

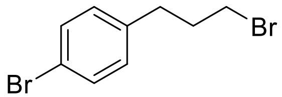

2b

500 MHz, ^{1}H NMR

$CDCl_3$

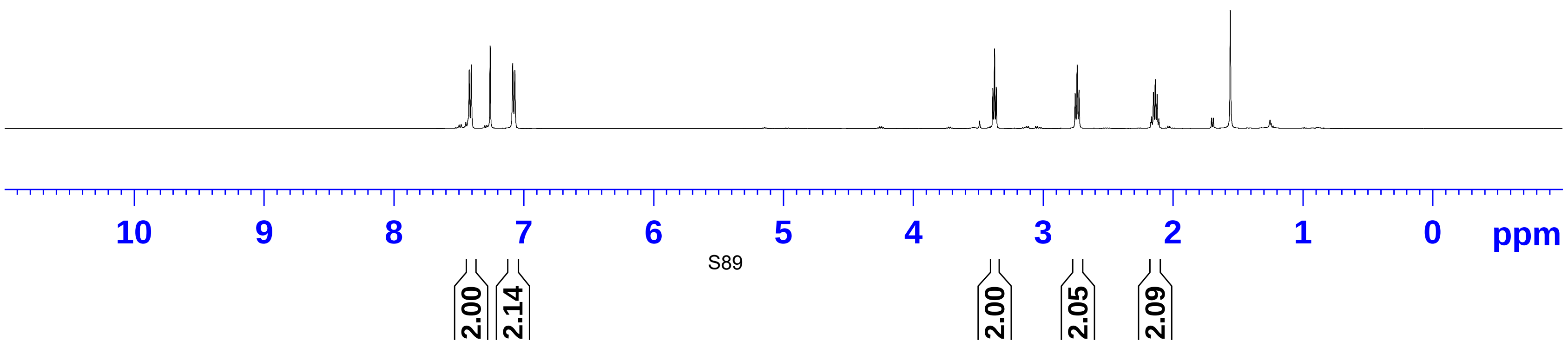

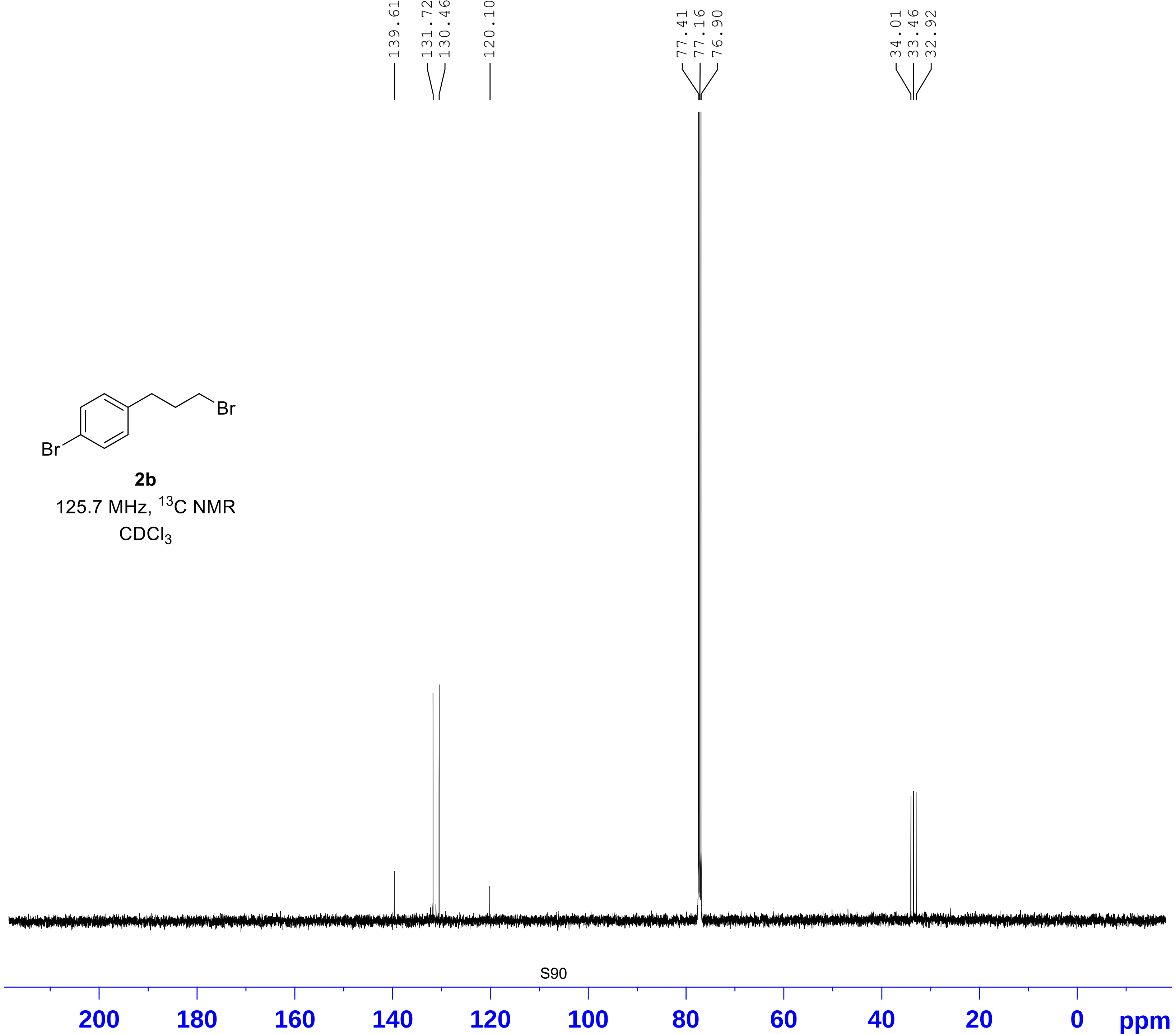

2b

125.7 MHz, ^{13}C NMR

$CDCl_3$

2c
500 MHz, ^{1}H NMR
$CDCl_3$

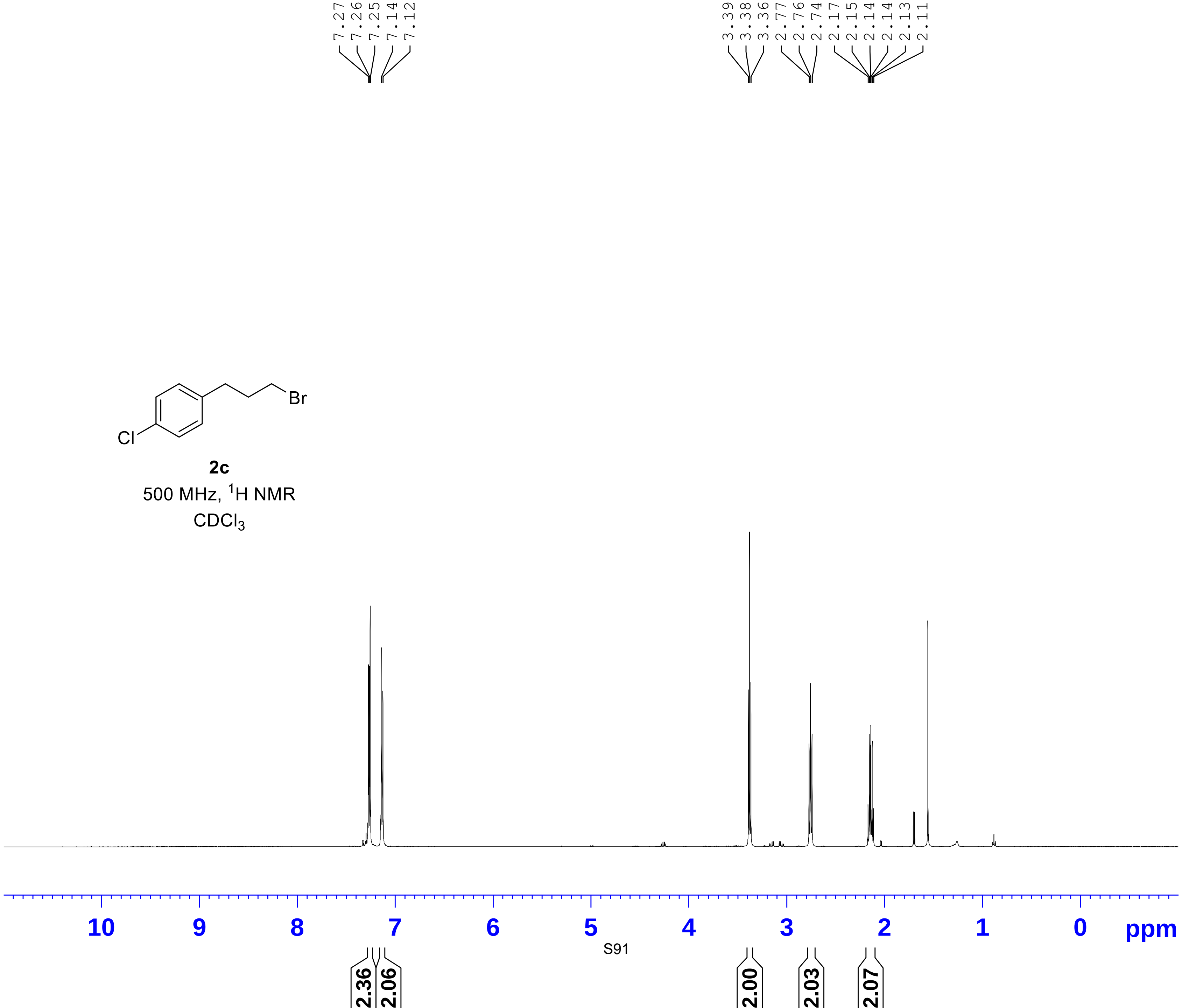

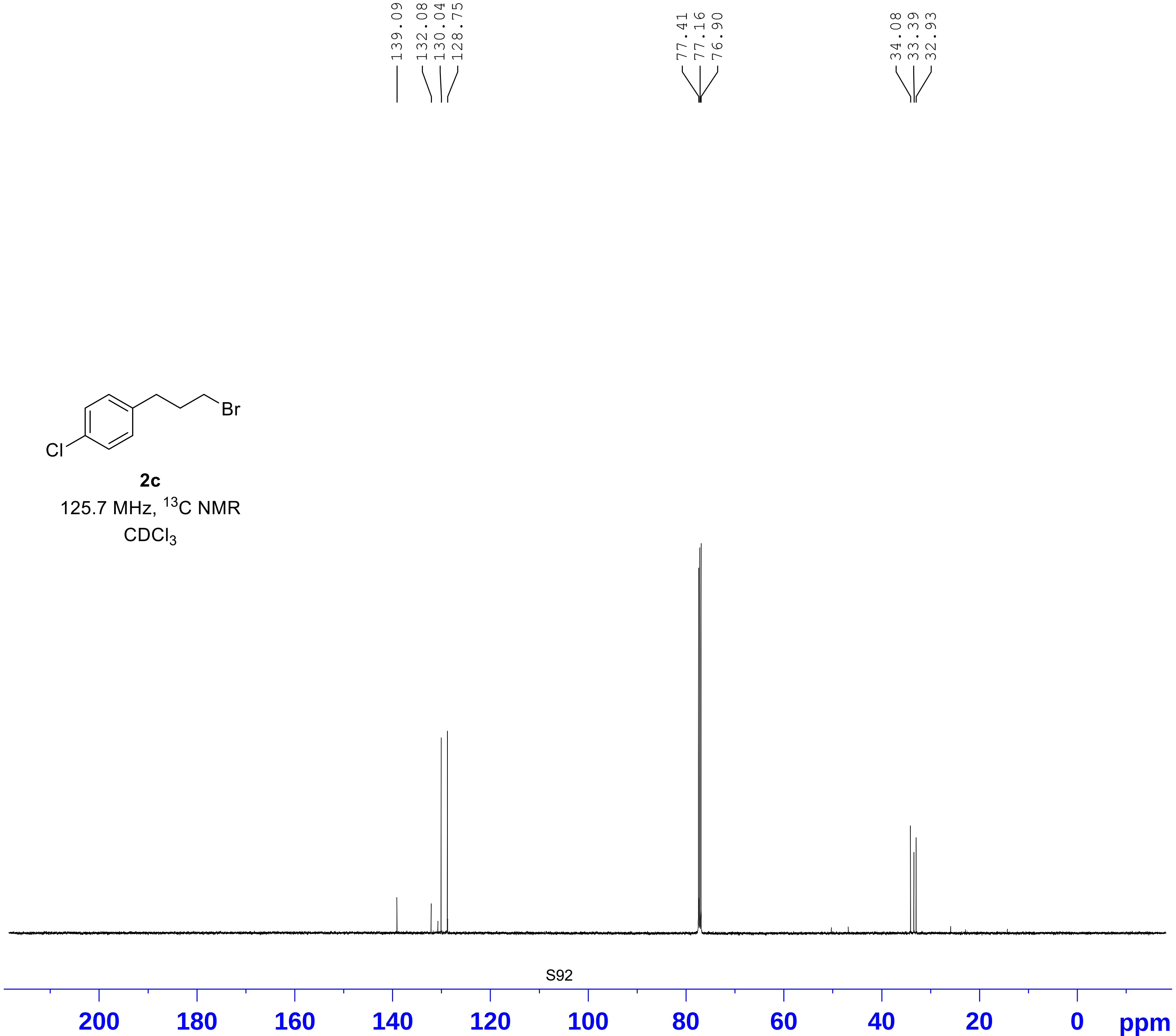

2c

125.7 MHz, ^{13}C NMR

$CDCl_3$

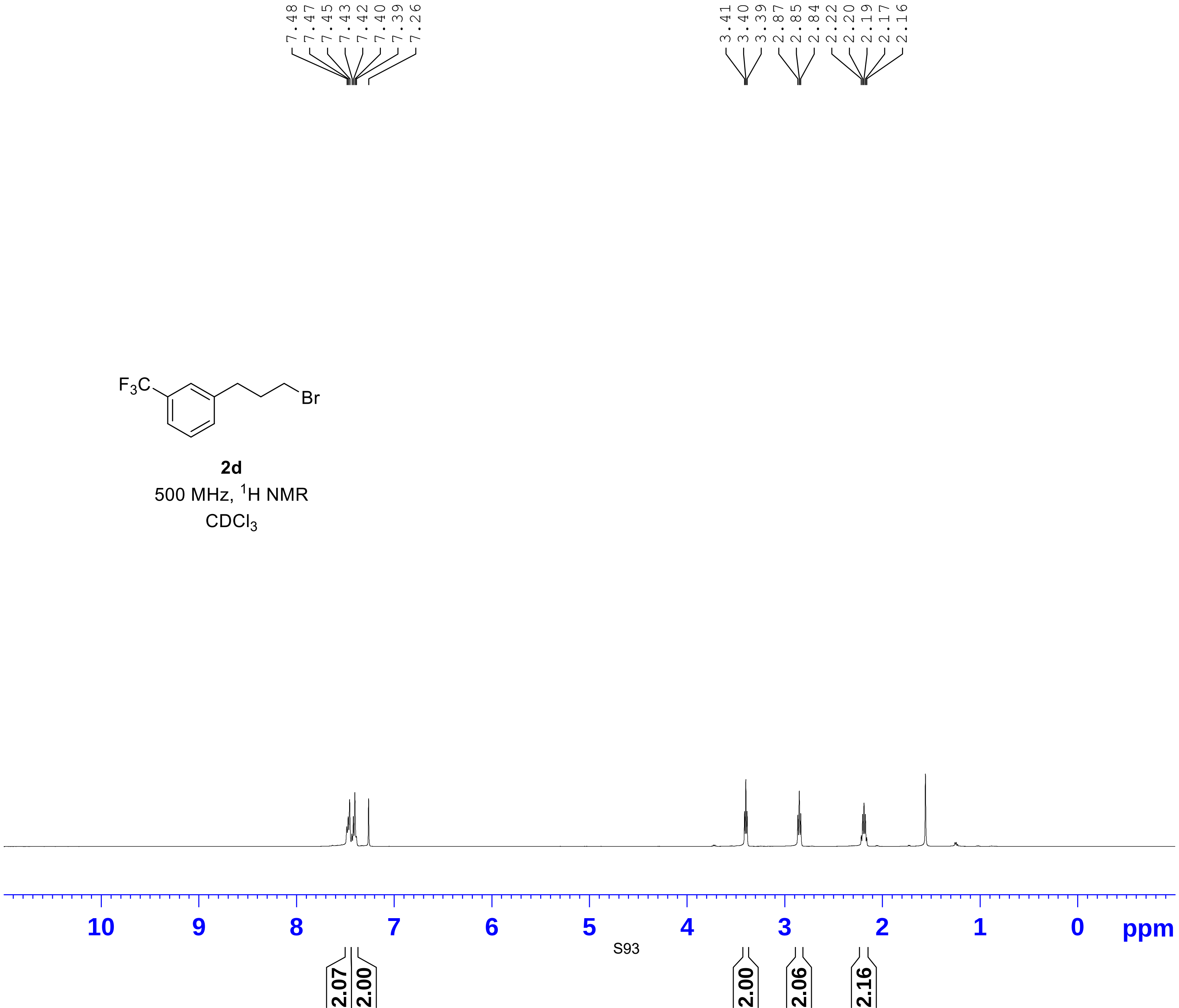
7.48 7.47 7.45 7.43 7.42 7.40 7.39 7.26
3.41 3.40 3.39 2.87 2.85 2.84 2.22 2.20 2.19 2.17 2.16
F_3C
Br
2d
500 MHz, ^{1}H NMR
$CDCl_3$
10 9 8 7 6 5 4 3 2 1 0 ppm
2.07 2.00 2.00 2.06 2.16

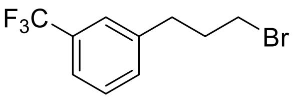

2d

125.7 MHz, ^{13}C NMR

$CDCl_3$

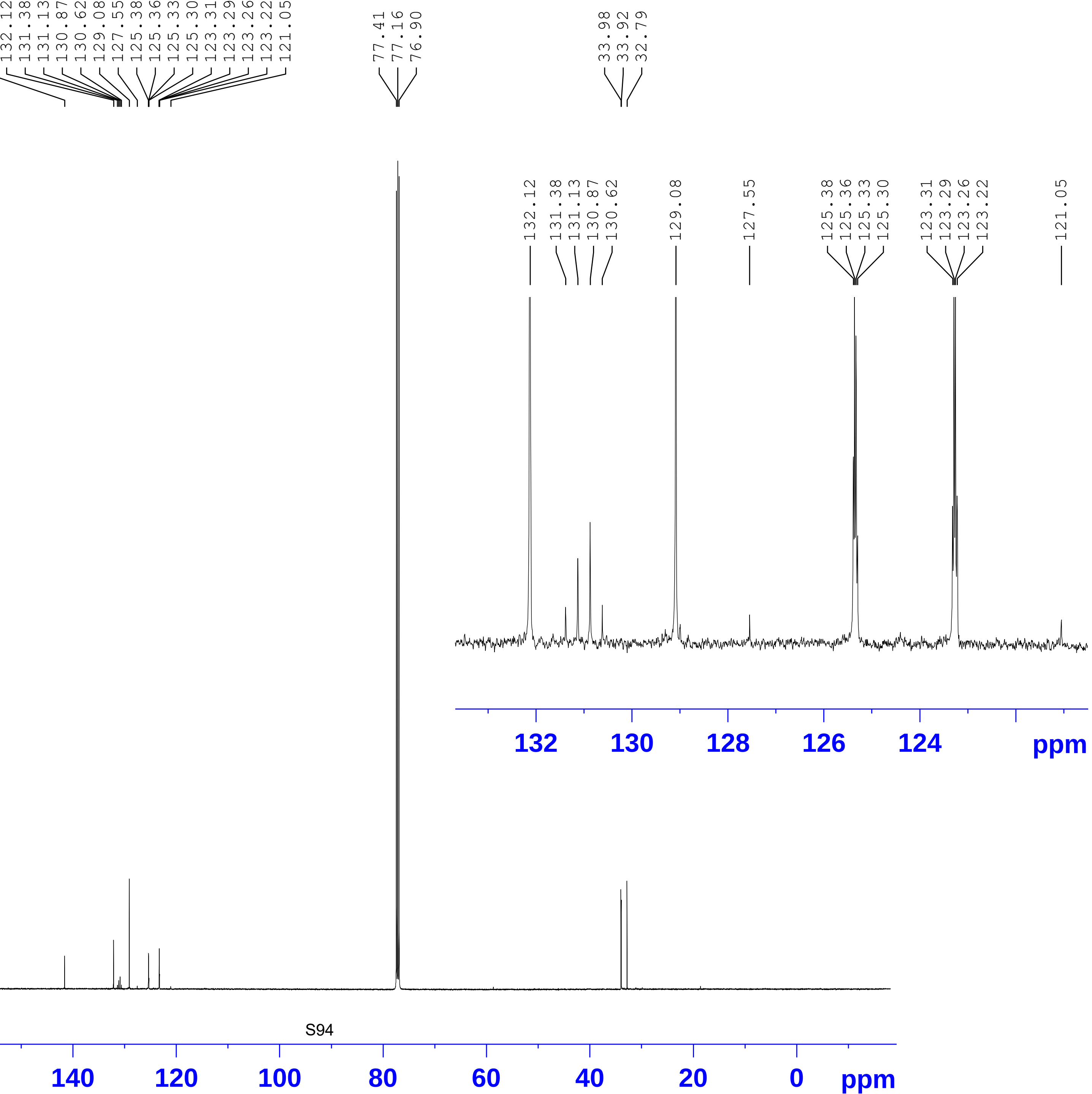

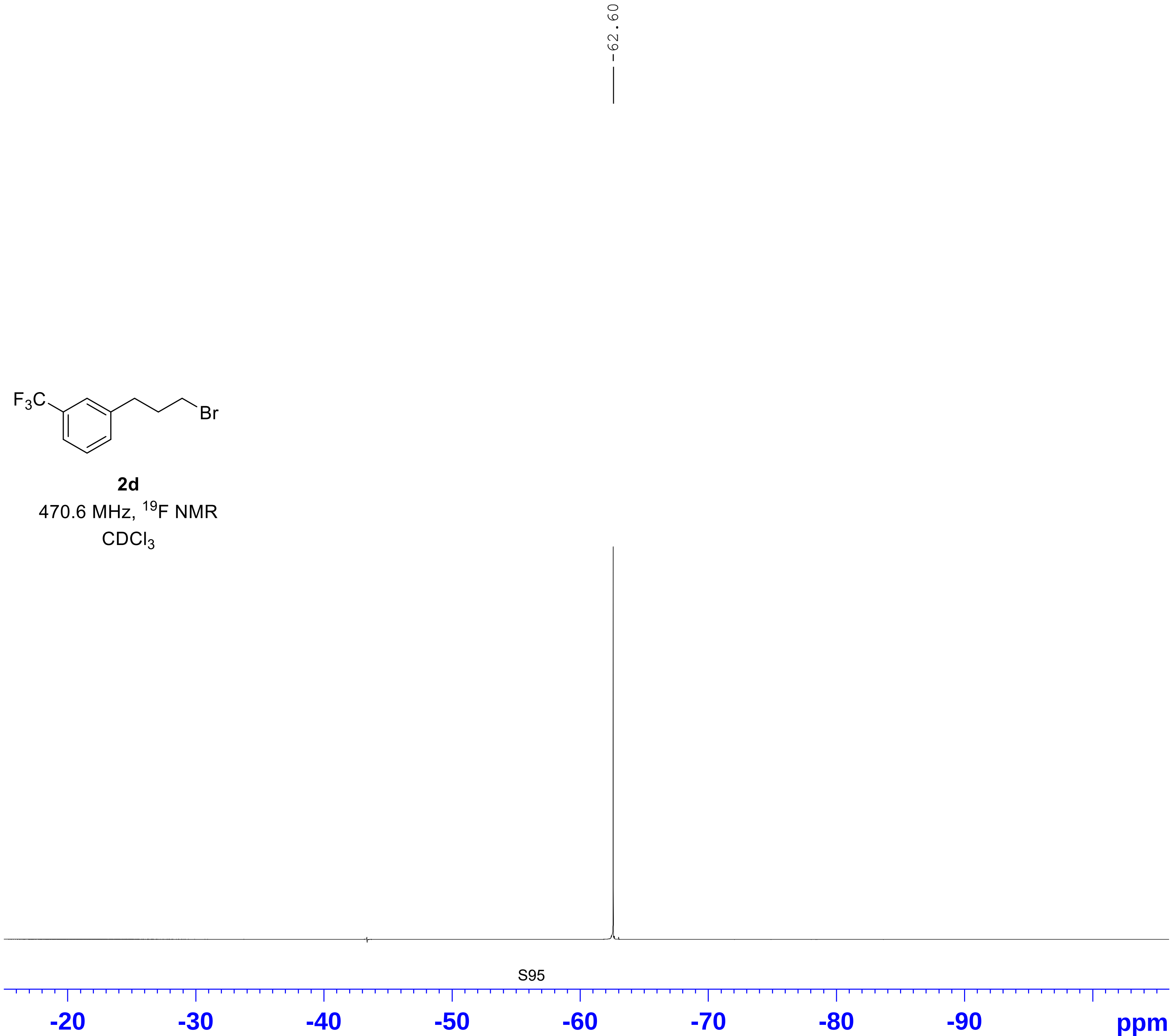

2d

470.6 MHz, ^{19}F NMR

$CDCl_3$

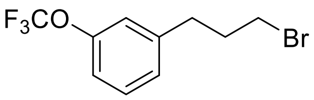

2e
500 MHz, ^1H NMR
$CDCl_3$

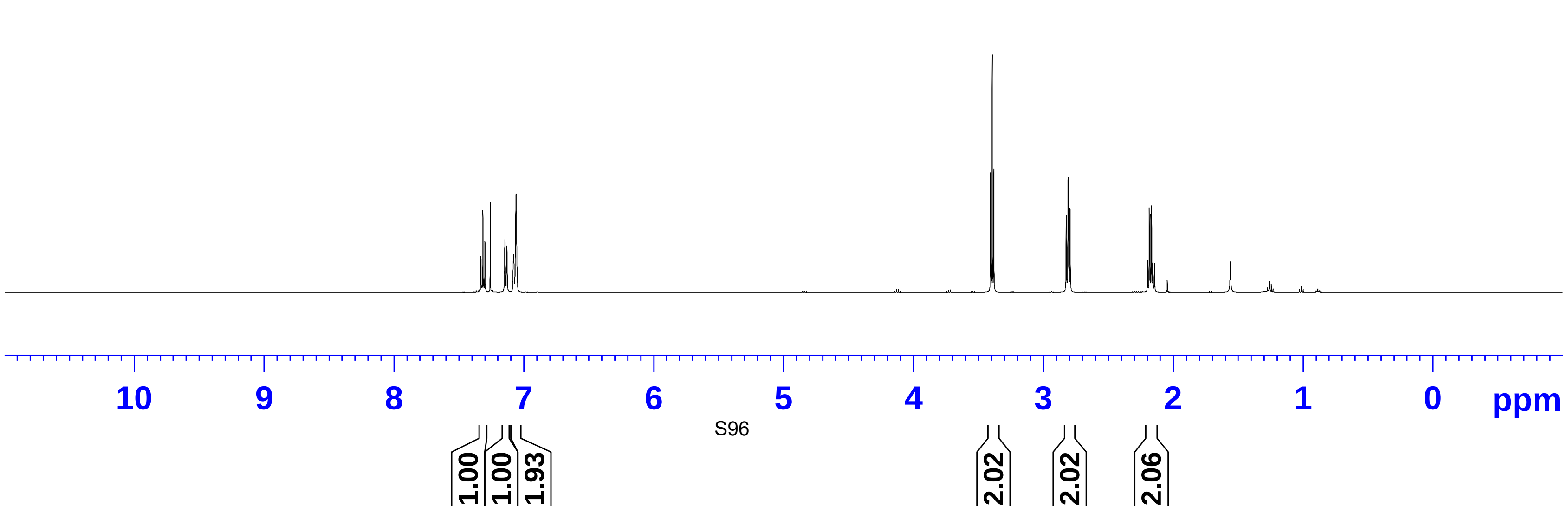

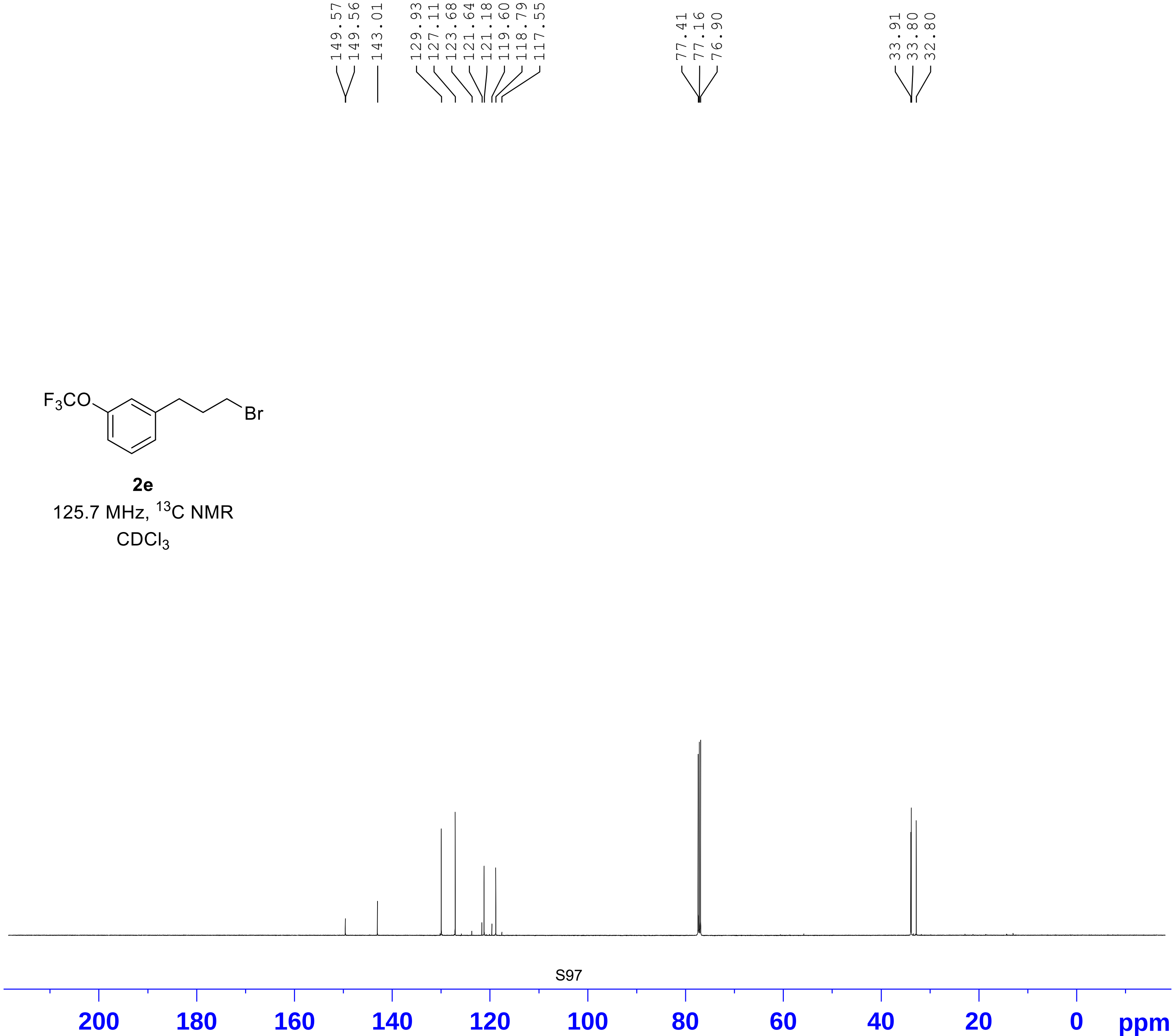

2e

125.7 MHz, ^{13}C NMR

$CDCl_3$

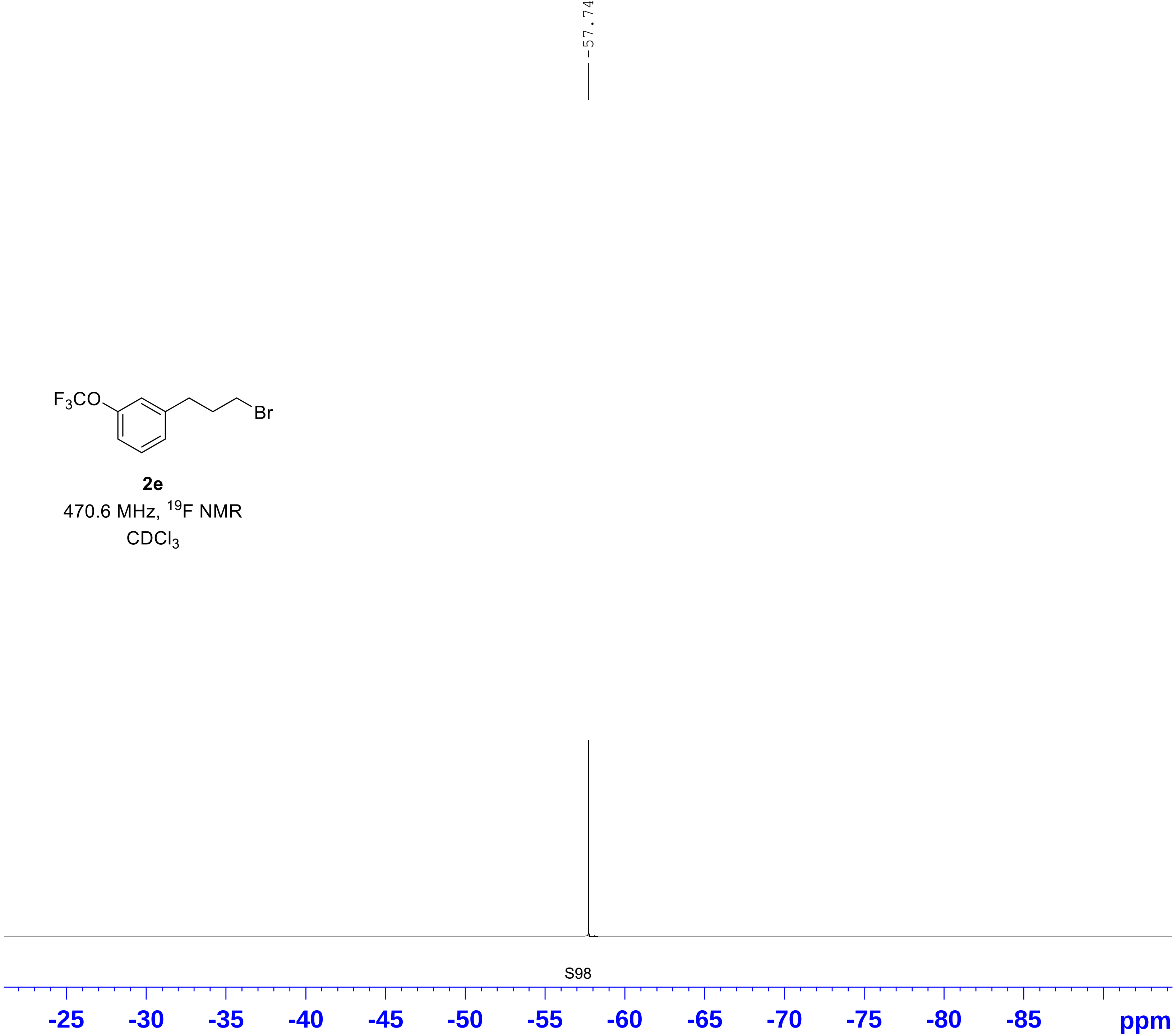
-57.74
F3CO
Br
2e
470.6 MHz, 19F NMR
CDCl3
-25 -30 -35 -40 -45 -50 -55 -60 -65 -70 -75 -80 -85 ppm

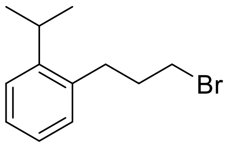

2f

500 MHz, ^{1}H NMR

$CDCl_3$

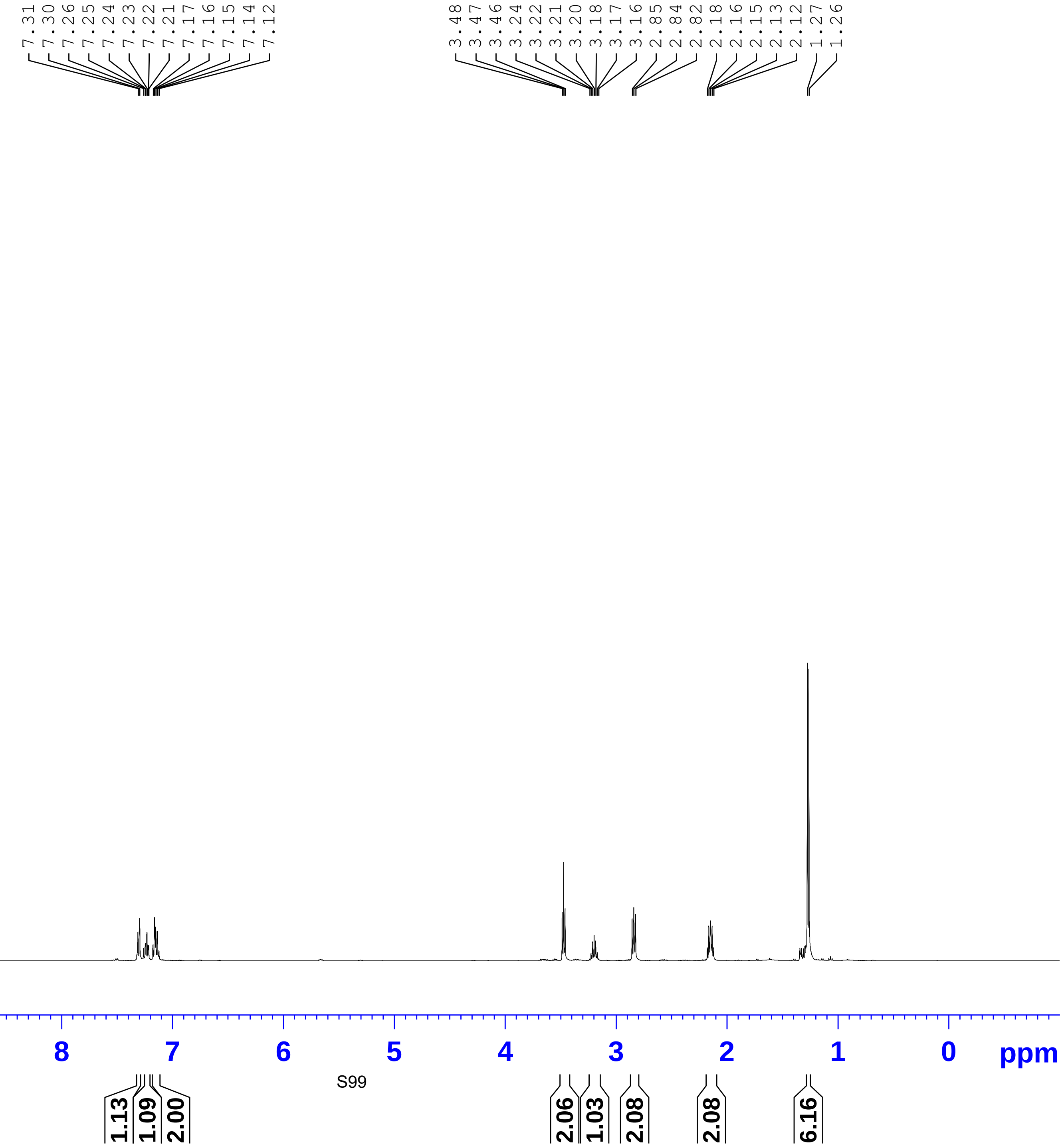

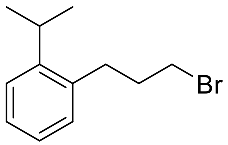

2f

125.7 MHz, ^{13}C NMR

$CDCl_3$

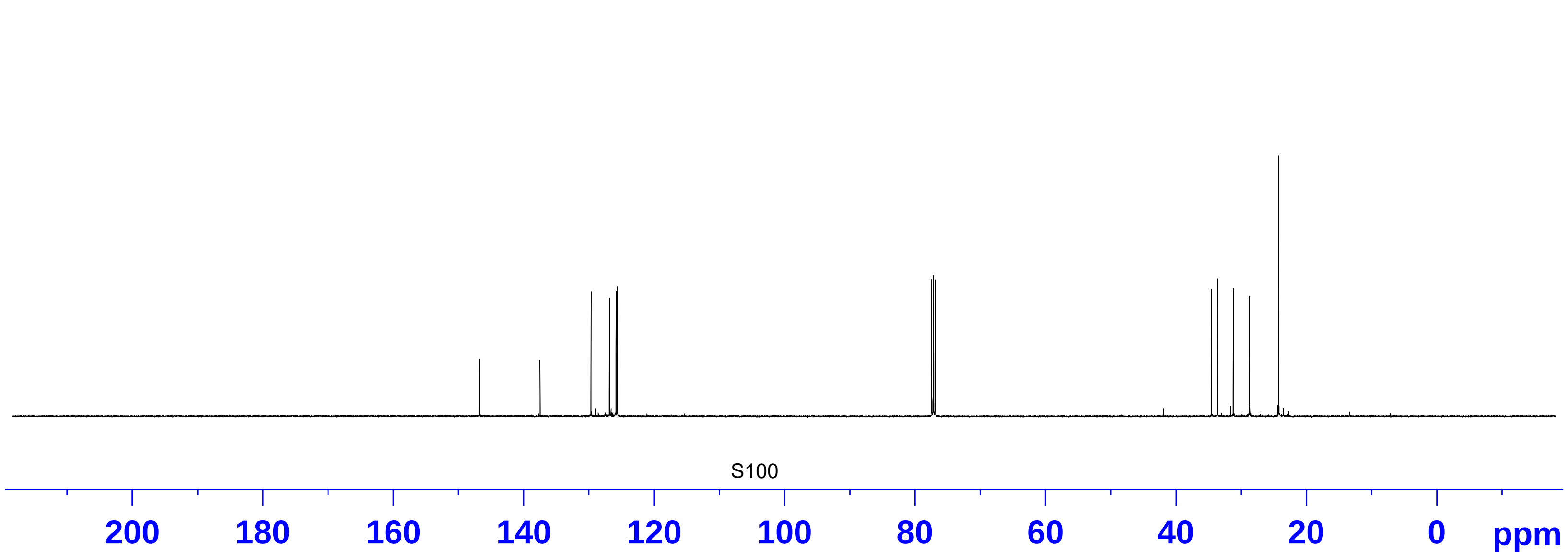

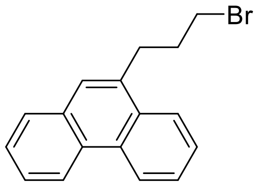

2g
500 MHz, ^{1}H NMR
$CDCl_3$

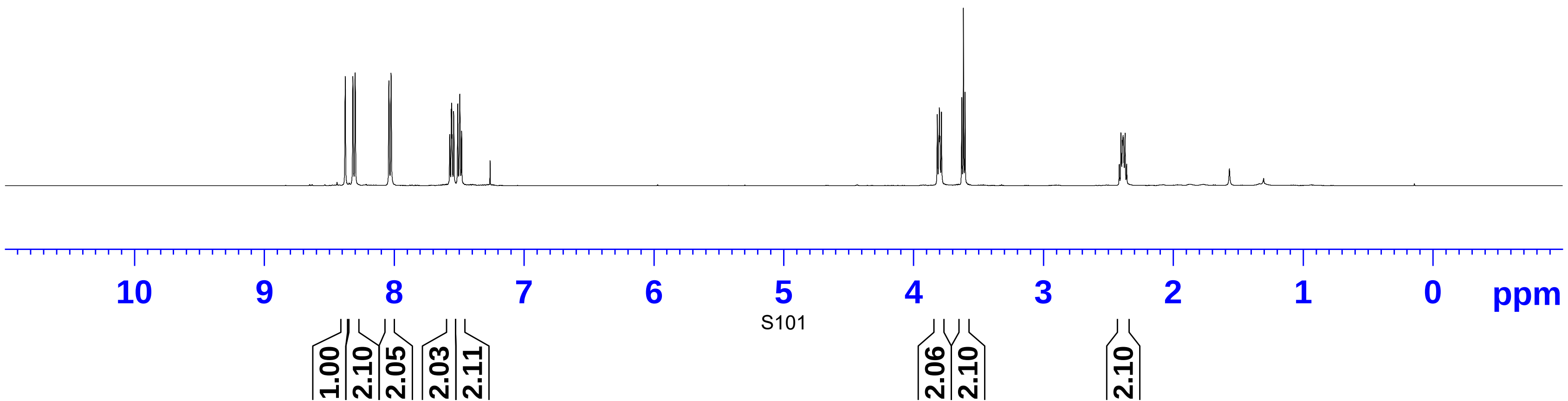

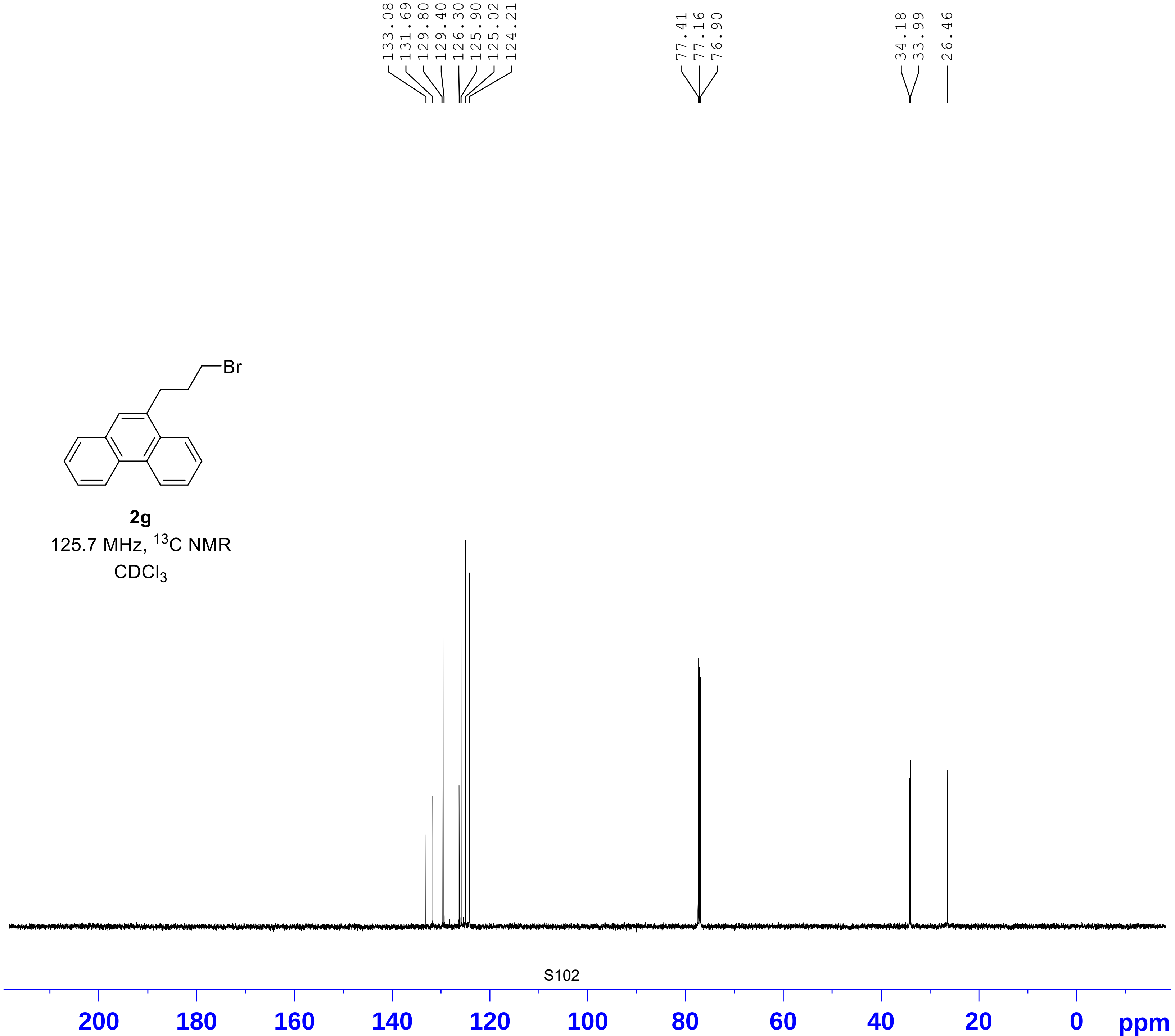

2g
125.7 MHz, ^{13}C NMR
$CDCl_3$

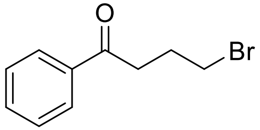

2h
500 MHz, ^1H NMR
$CDCl_3$

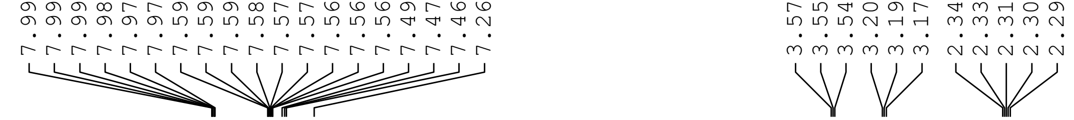

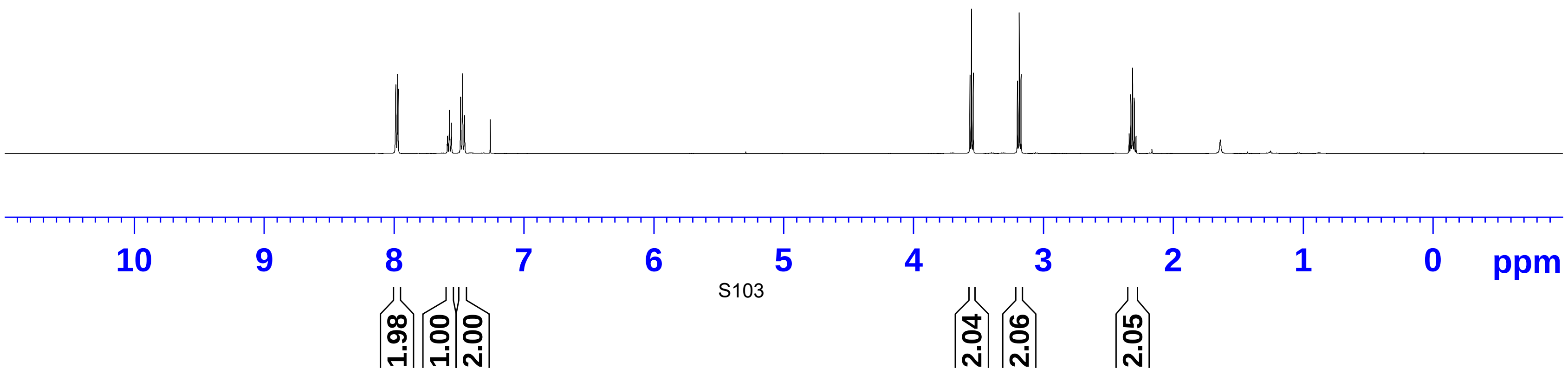

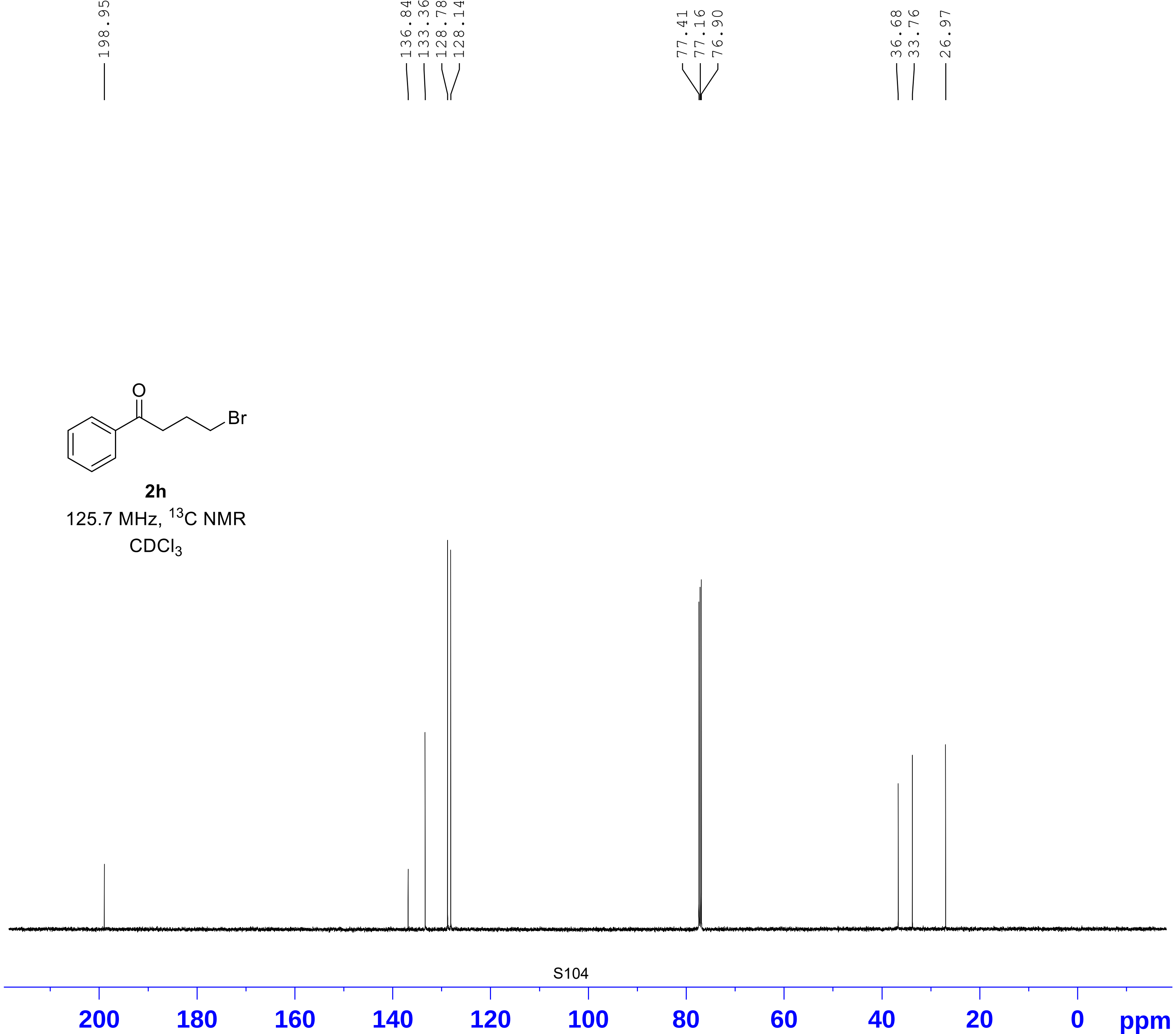
198.95
136.84
133.36
128.78
128.14
77.41
77.16
76.90
36.68
33.76
26.97
O
Br
2h
125.7 MHz, 13C NMR
CDCl3
200
180
160
140
120
100
80
60
40
20
0
ppm

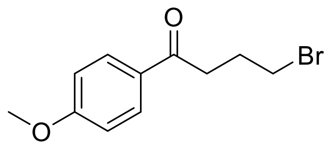

2i

500 MHz, ^{1}H NMR

$CDCl_3$

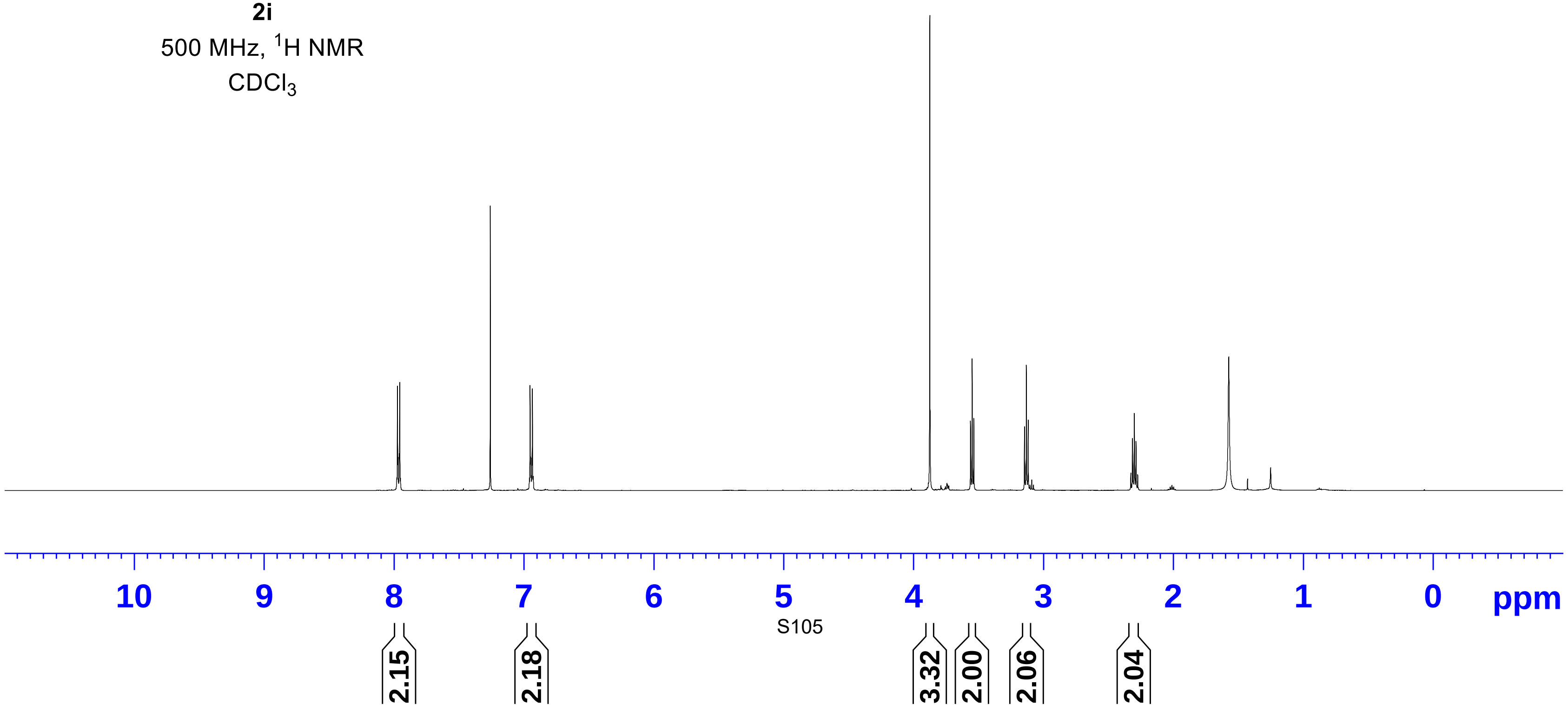

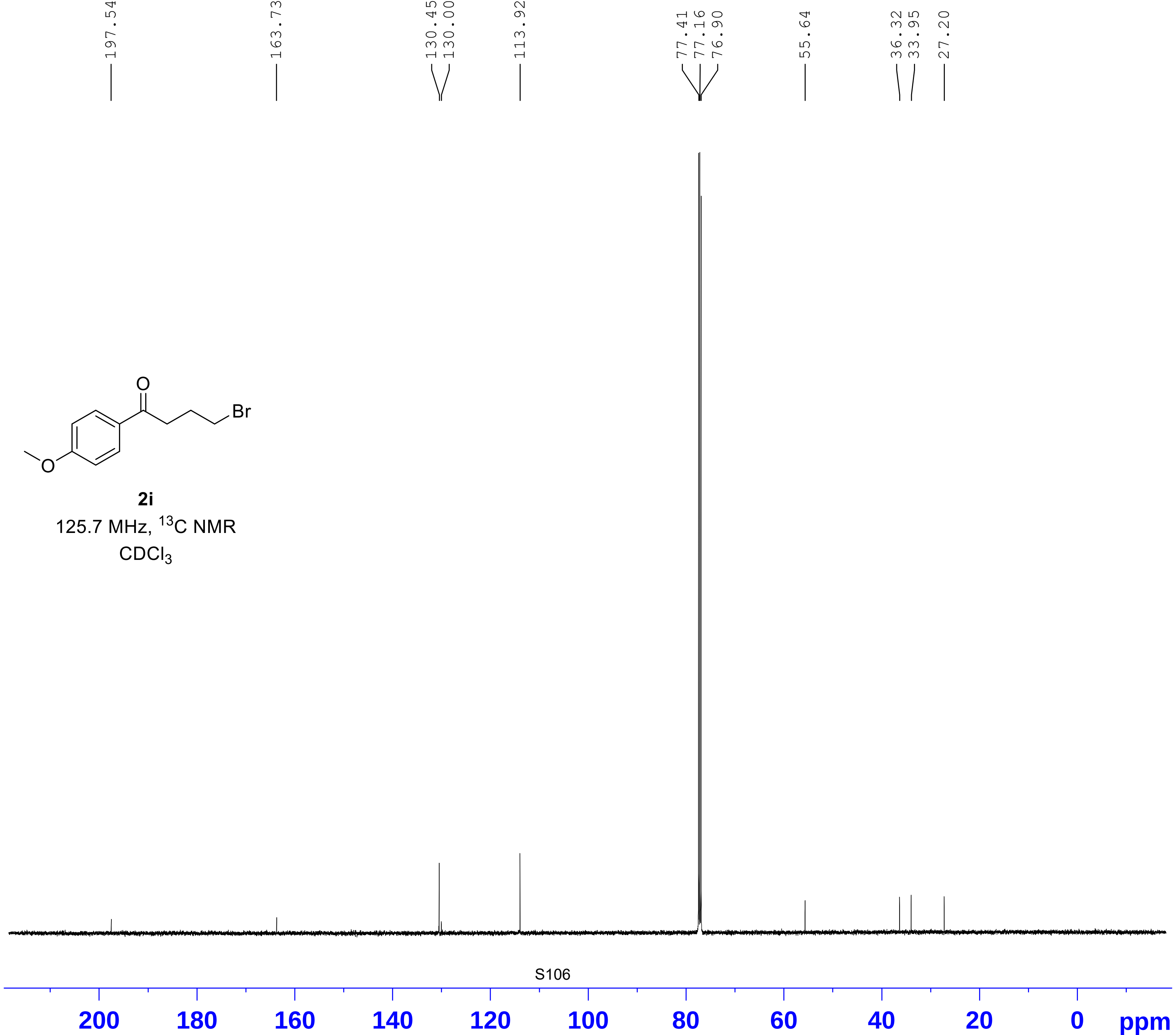

2i

125.7 MHz, ^{13}C NMR

$CDCl_3$

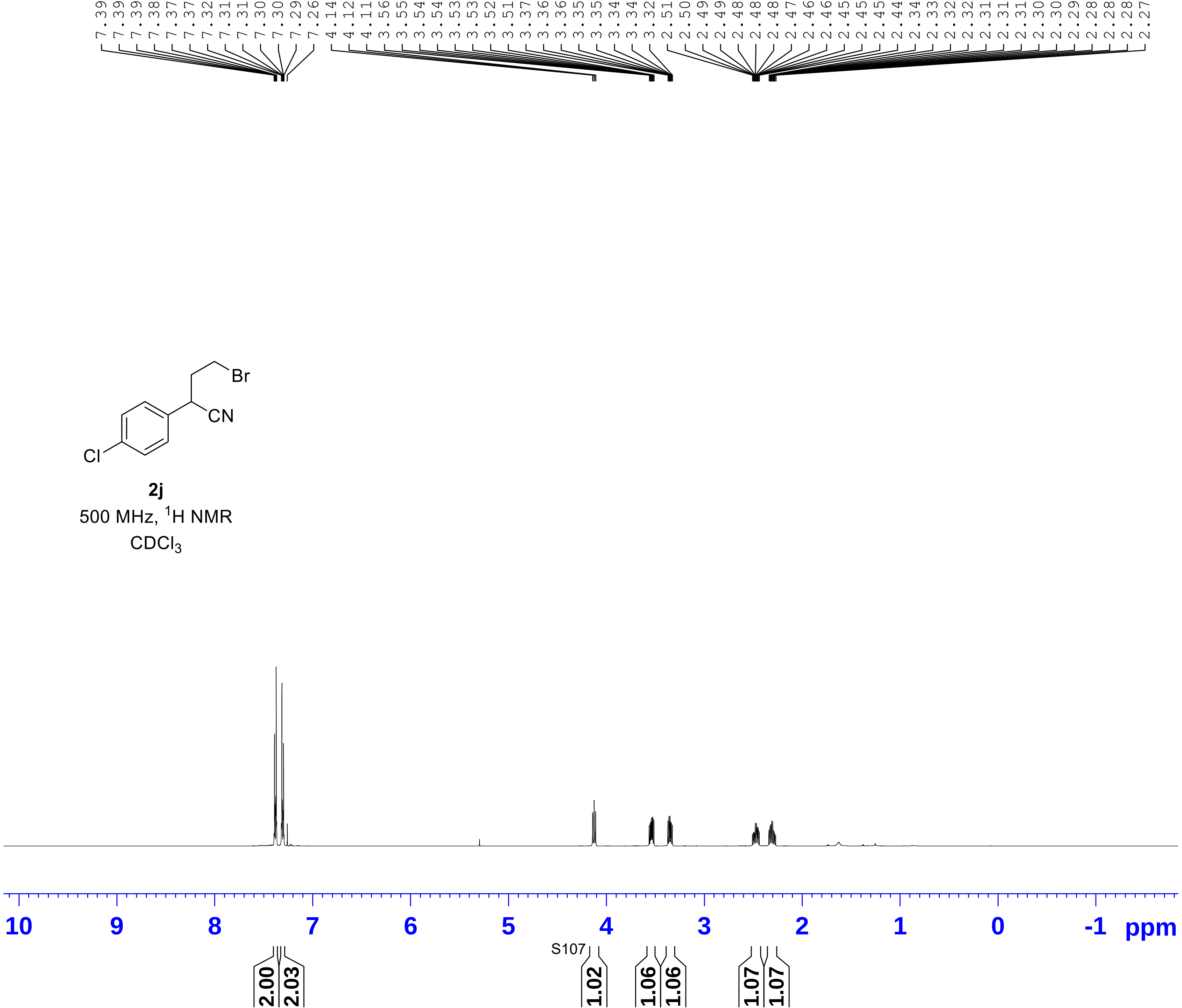

2j

500 MHz, ^{1}H NMR

$CDCl_3$

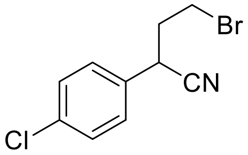

2j
125.7 MHz, ^{13}C NMR
$CDCl_3$

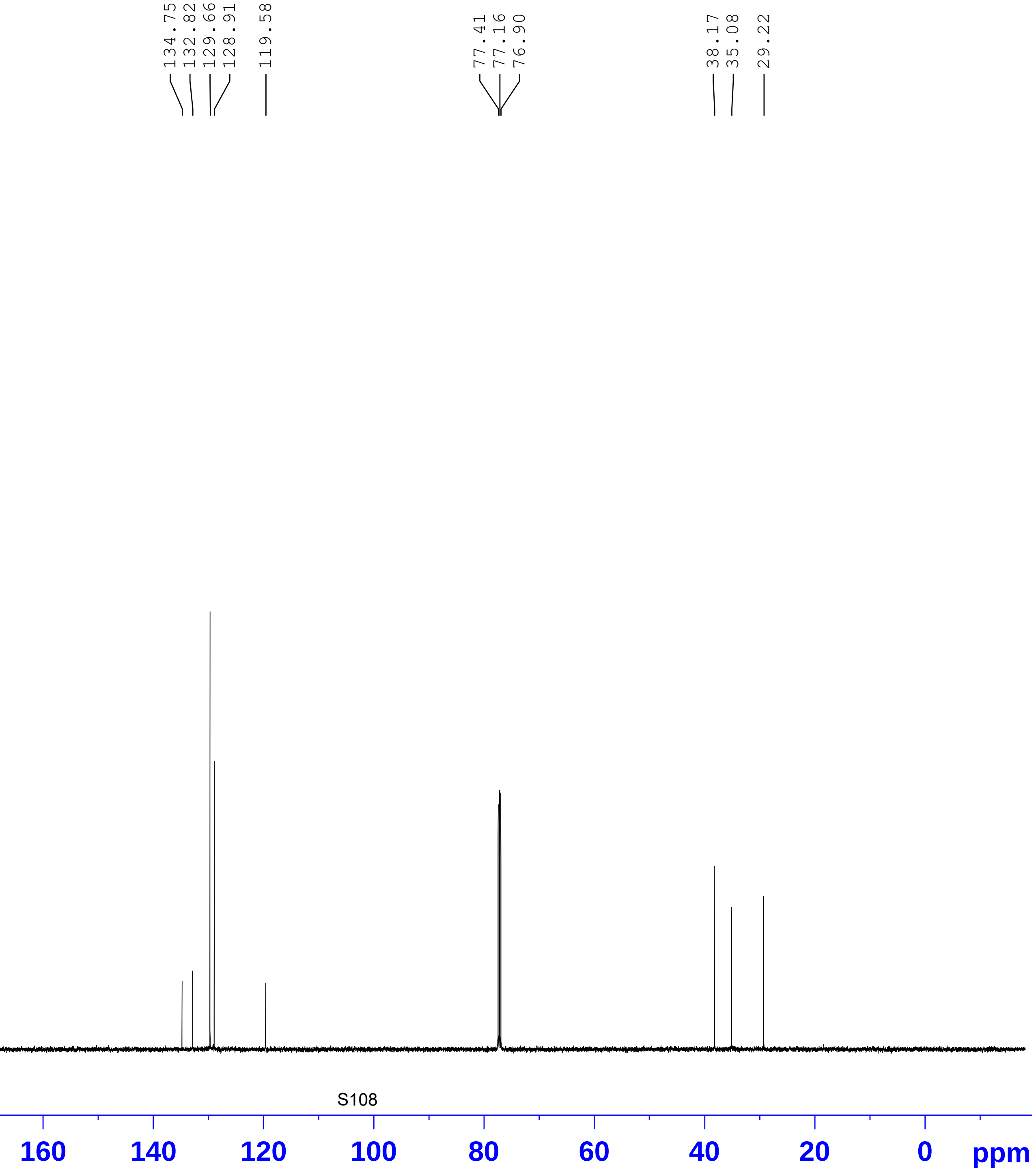

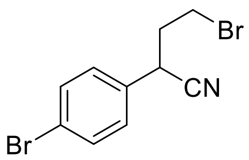

2k

500 MHz, ^1H NMR

$CDCl_3$

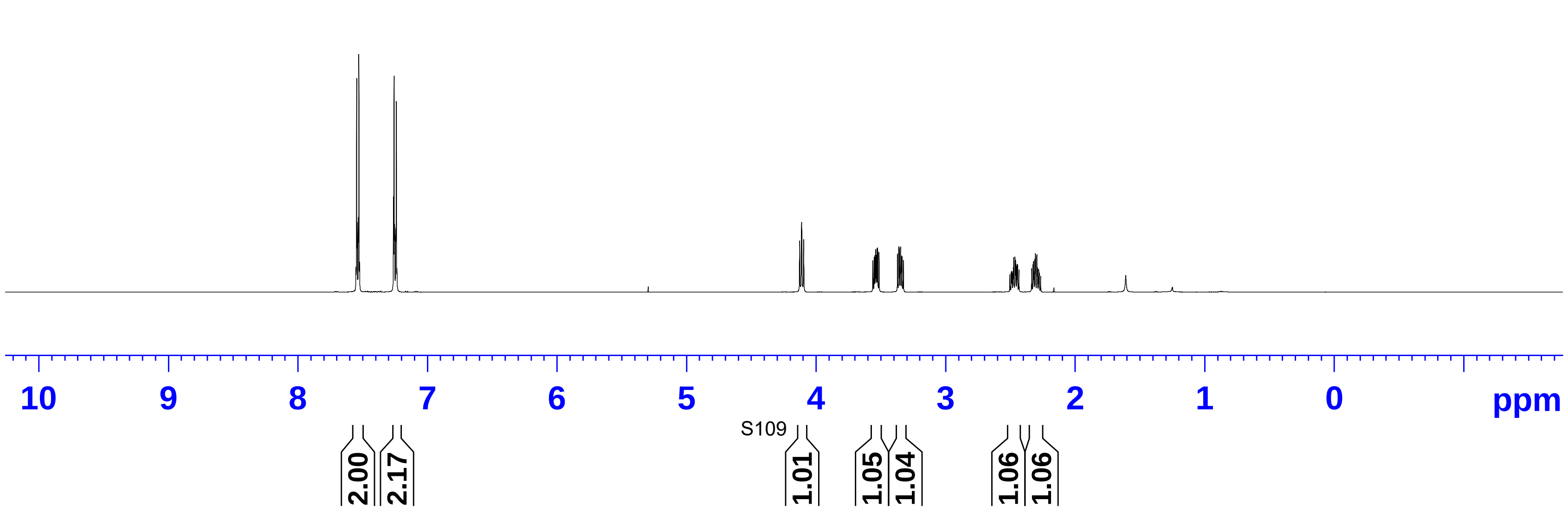

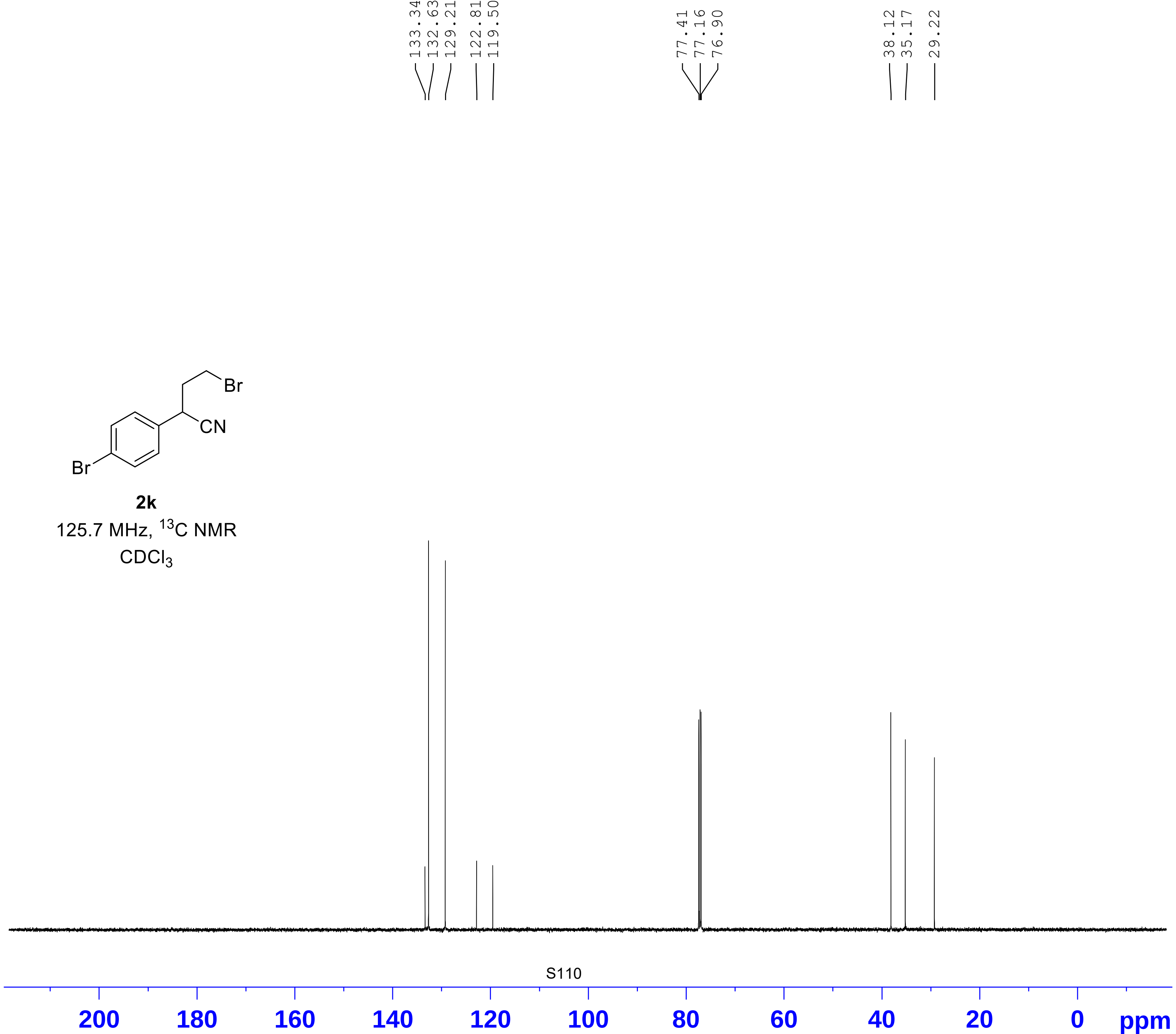

133.34
132.63
129.21
122.81
119.50
77.41
77.16
76.90
38.12
35.17
29.22
Br
CN
Br
2k
125.7 MHz, 13C NMR
CDCl3
200
180
160
140
120
100
80
60
40
20
0
ppm

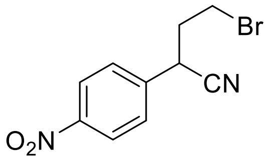

2l

500 MHz, ^{1}H NMR

$CDCl_3$

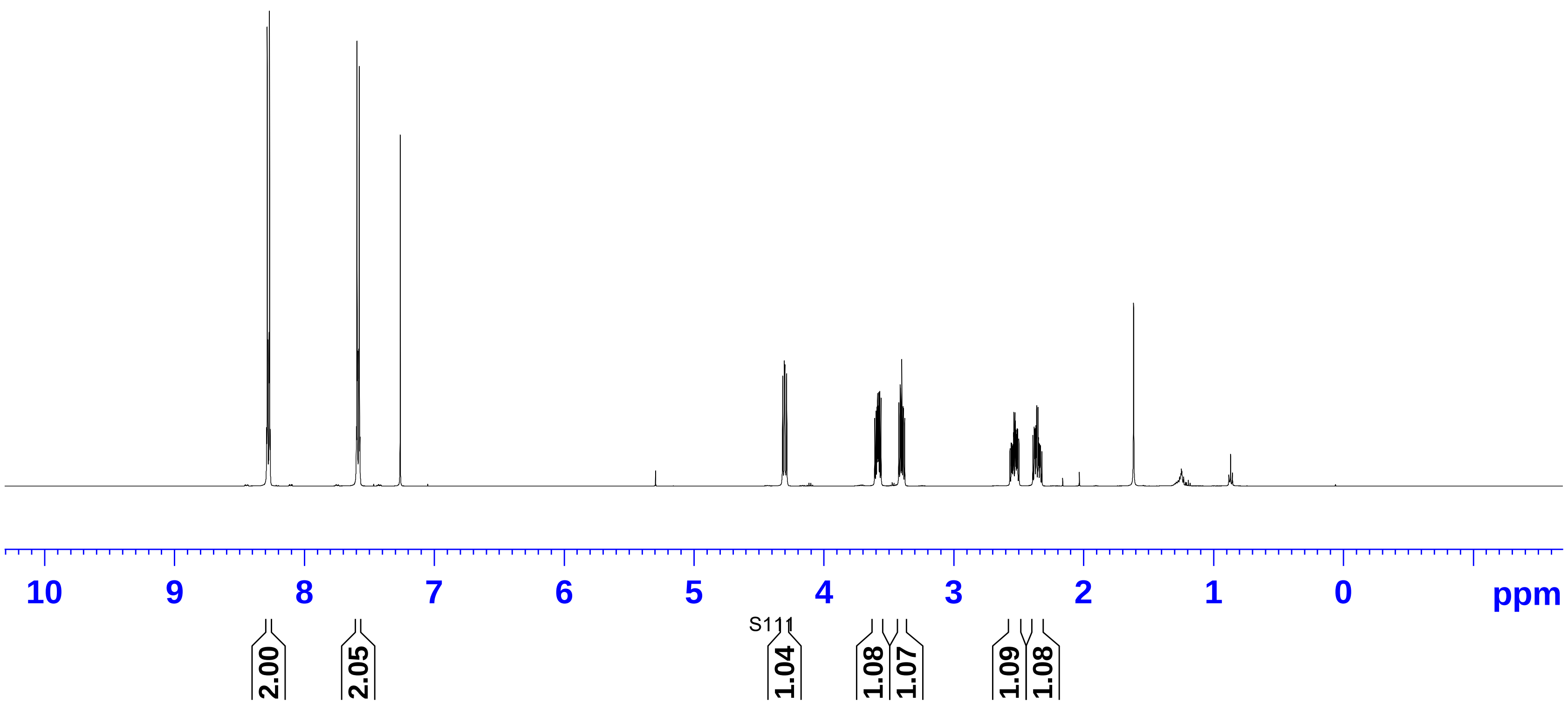

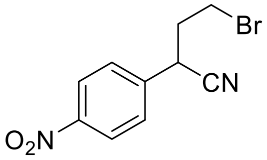

2l

125.7 MHz, ^{13}C NMR

$CDCl_3$

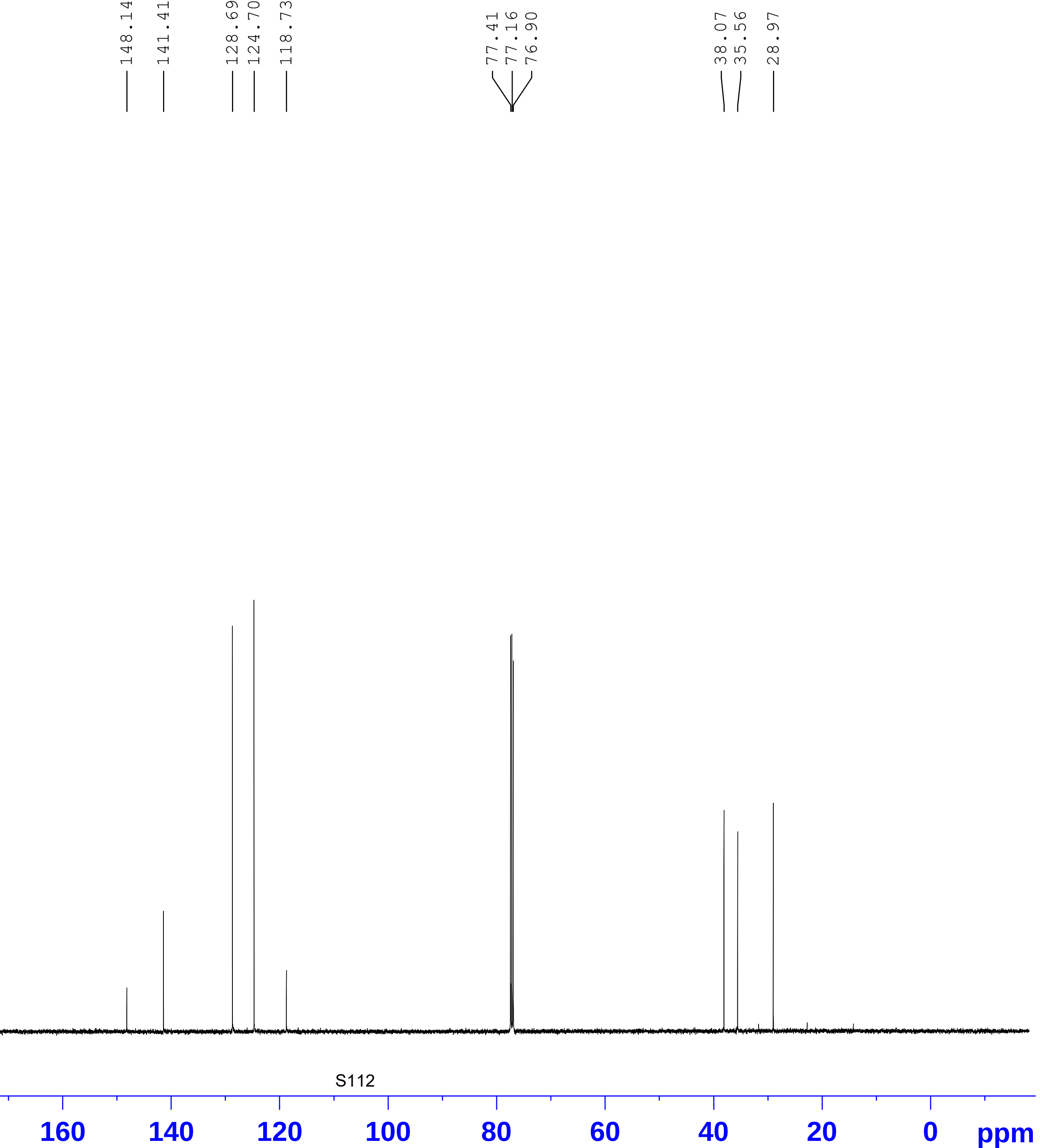

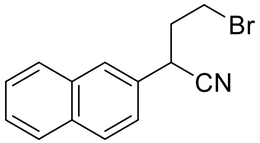

2m
500 MHz, ^{1}H NMR
$CDCl_3$

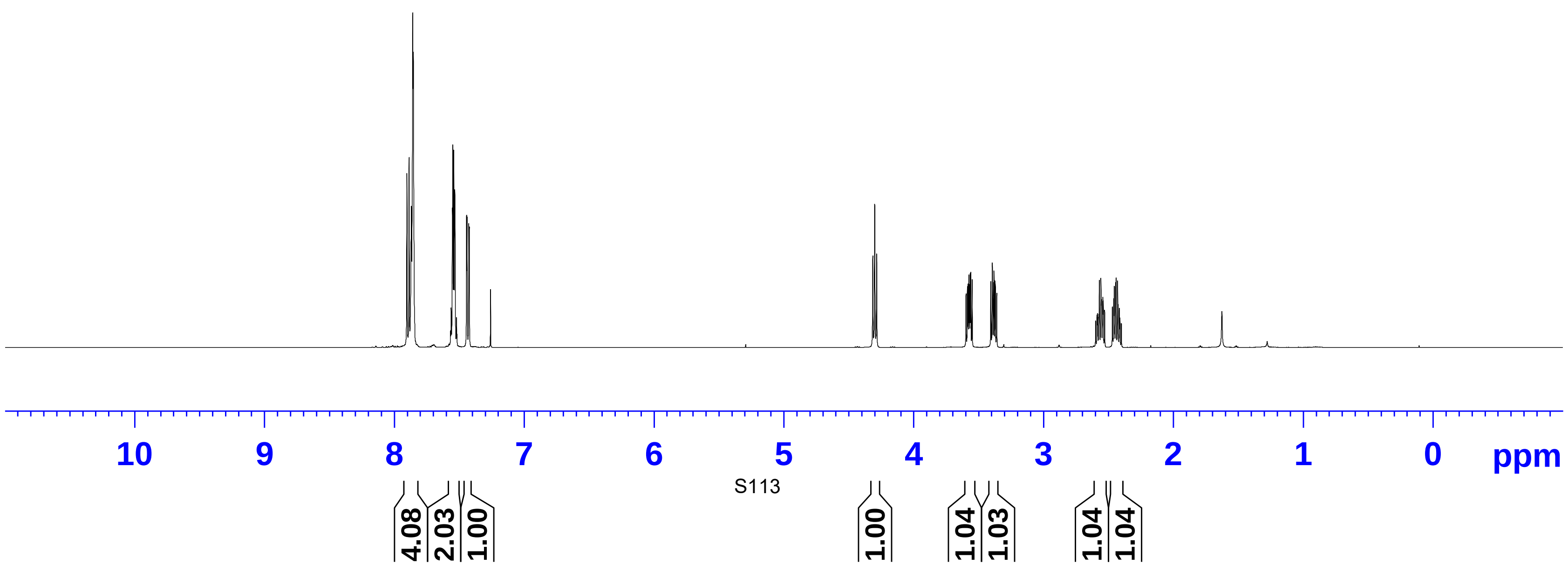

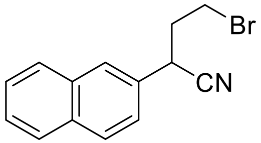

2m
125.7 MHz, ^{13}C NMR
$CDCl_3$

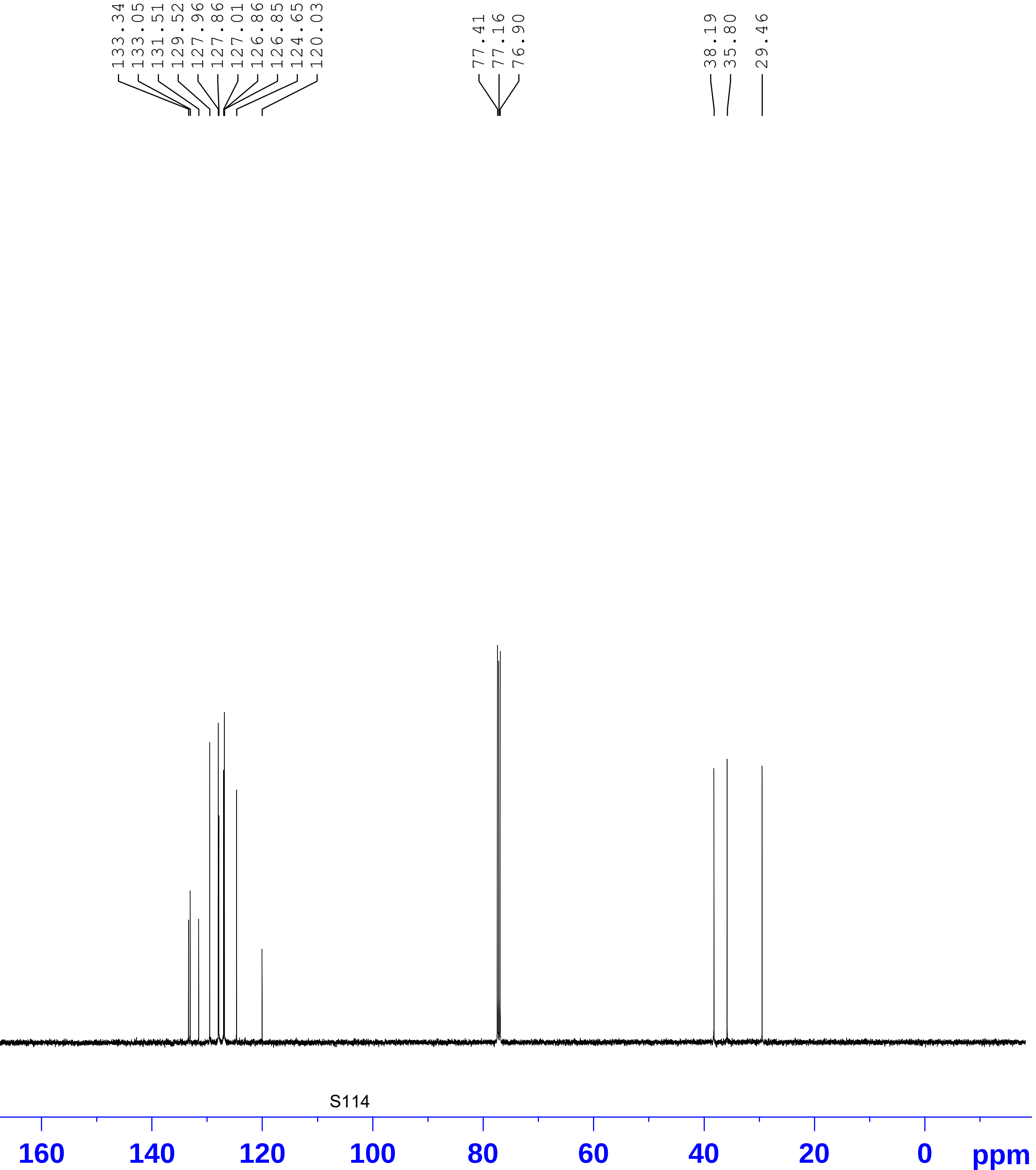

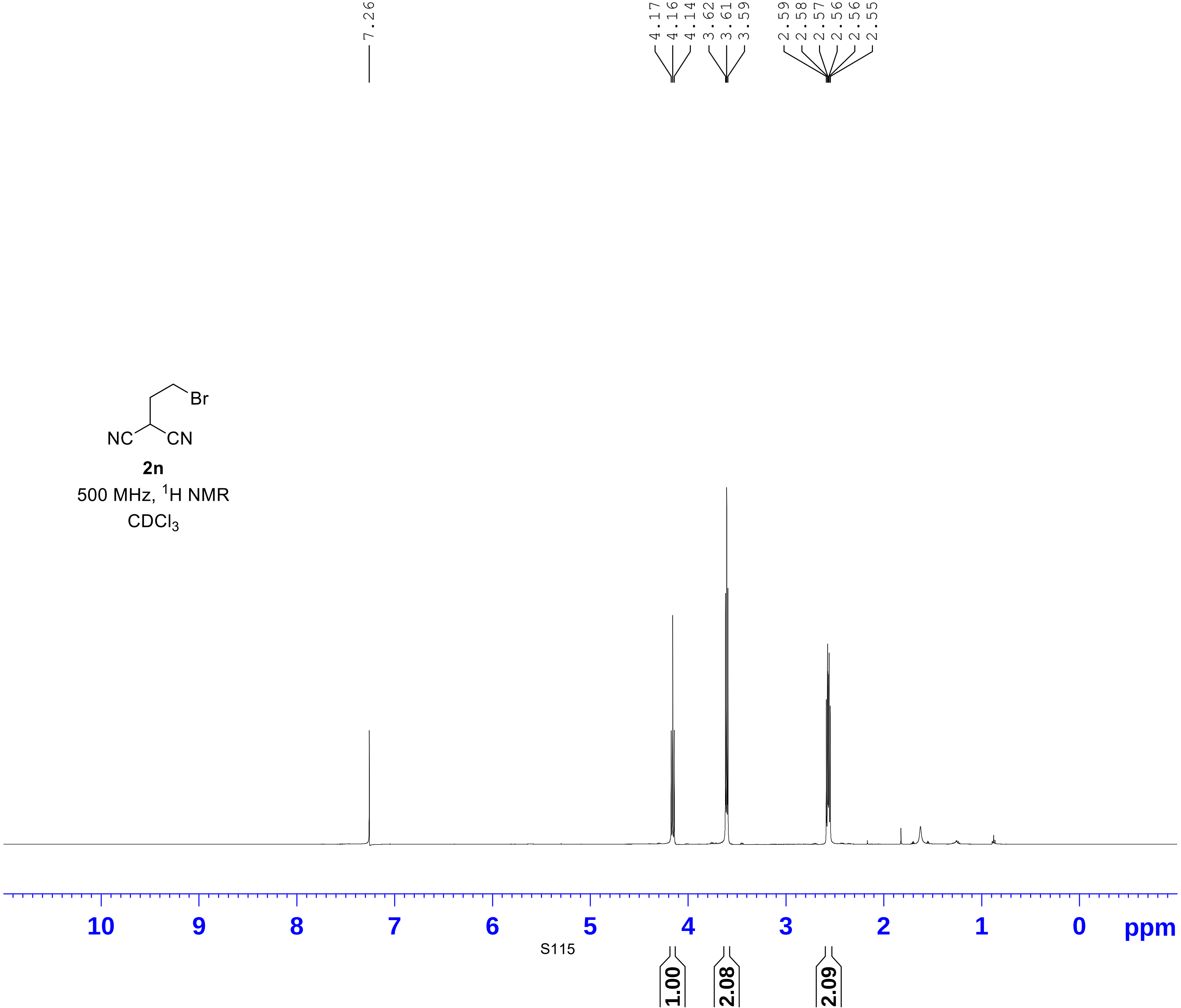
7.26
4.17
4.16
4.14
3.62
3.61
3.59
2.59
2.58
2.57
2.56
2.56
2.55
Br
NC
CN
2n
500 MHz, 1H NMR
CDCl3
10
9
8
7
6
5
4
3
2
1
0
ppm
1.00
2.08
2.09

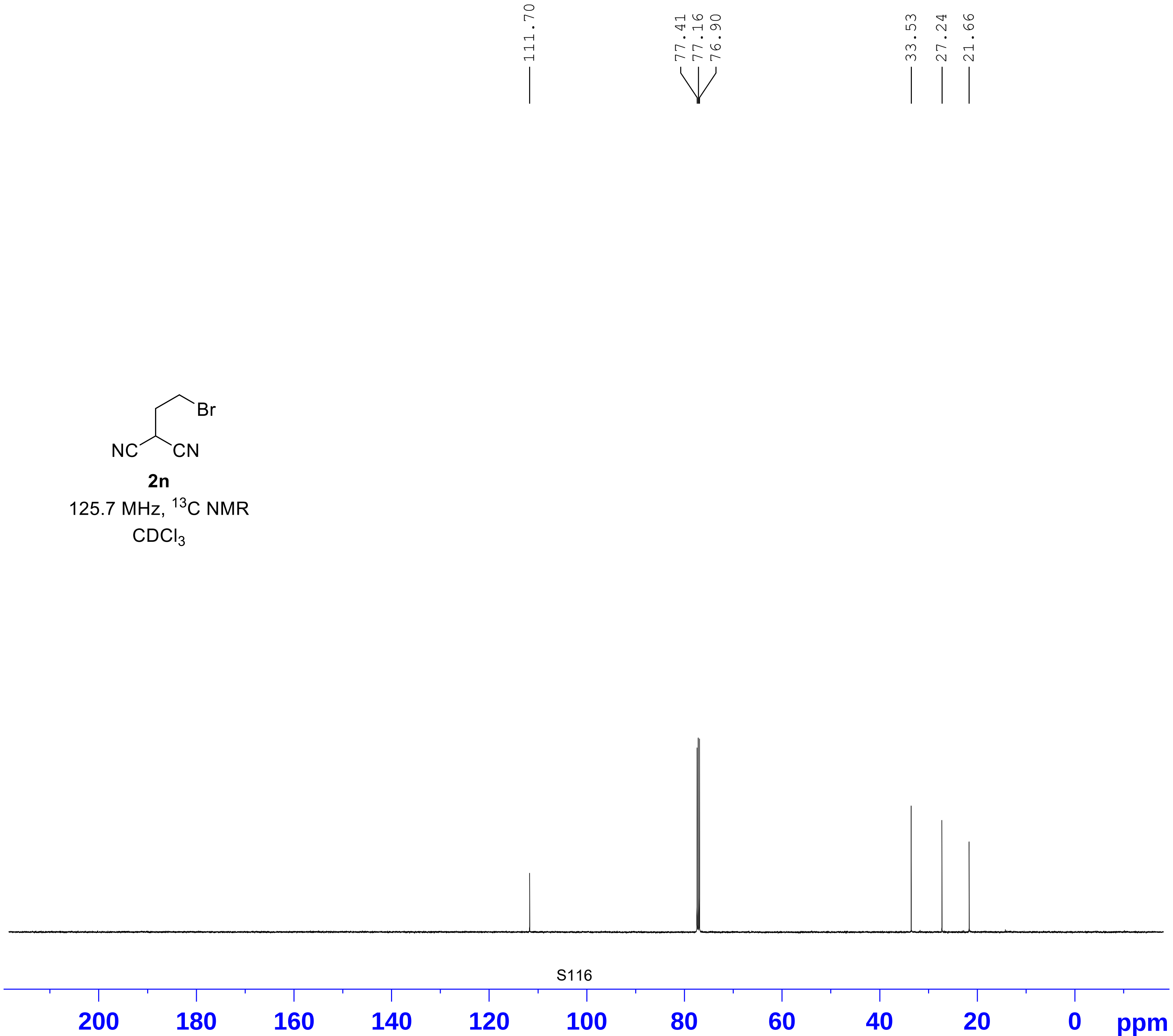

2n

125.7 MHz, ^{13}C NMR

$CDCl_3$

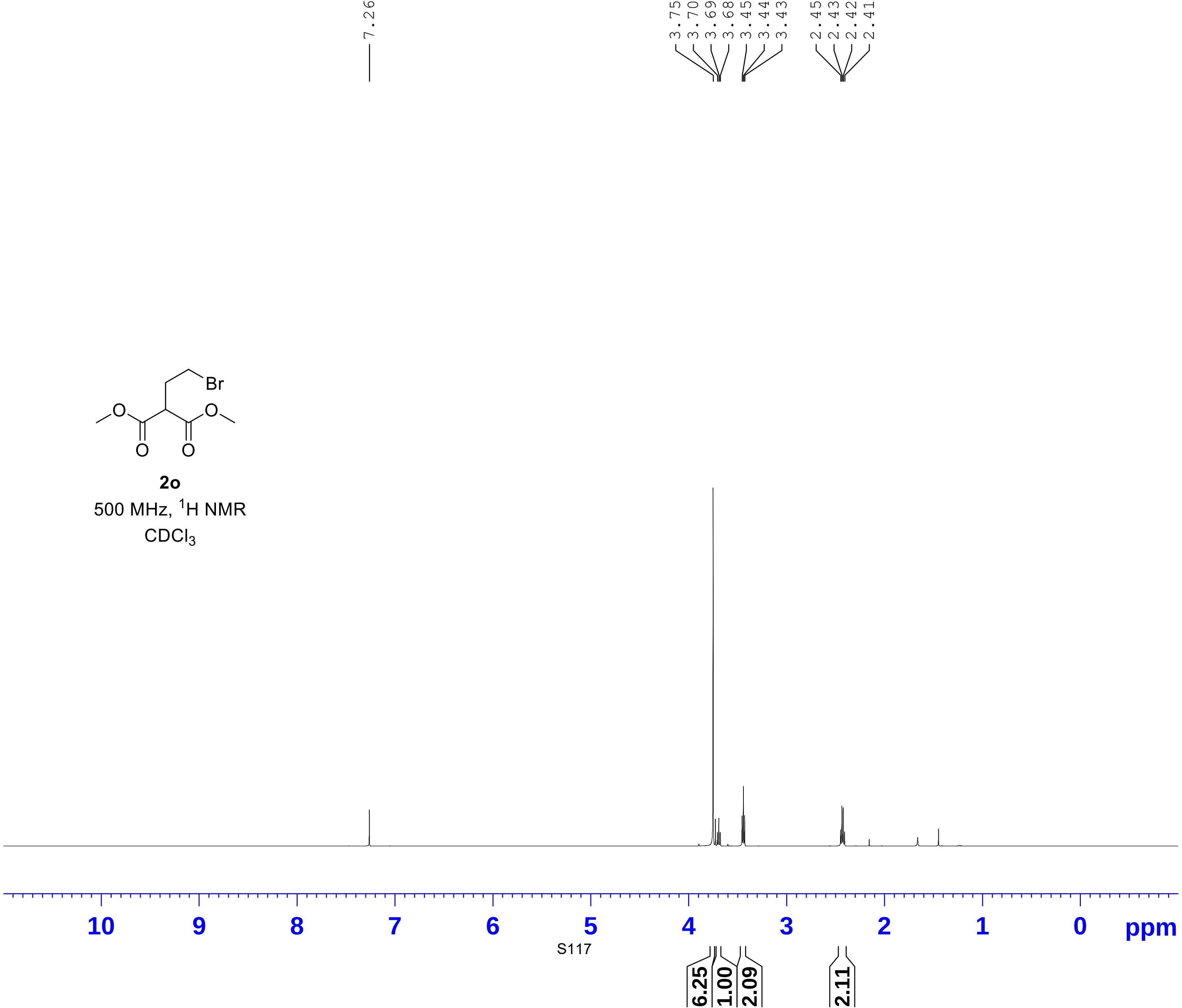

7.26
3.75
3.70
3.69
3.68
3.45
3.44
3.43
2.45
2.43
2.42
2.41
Br
O
O
O
O
2o
500 MHz, 1H NMR
CDCl3
10
9
8
7
6
5
4
3
2
1
0
ppm
6.25
1.00
2.09
2.11

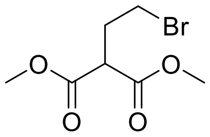

2o

125.7 MHz, ^{13}C NMR

$CDCl_3$

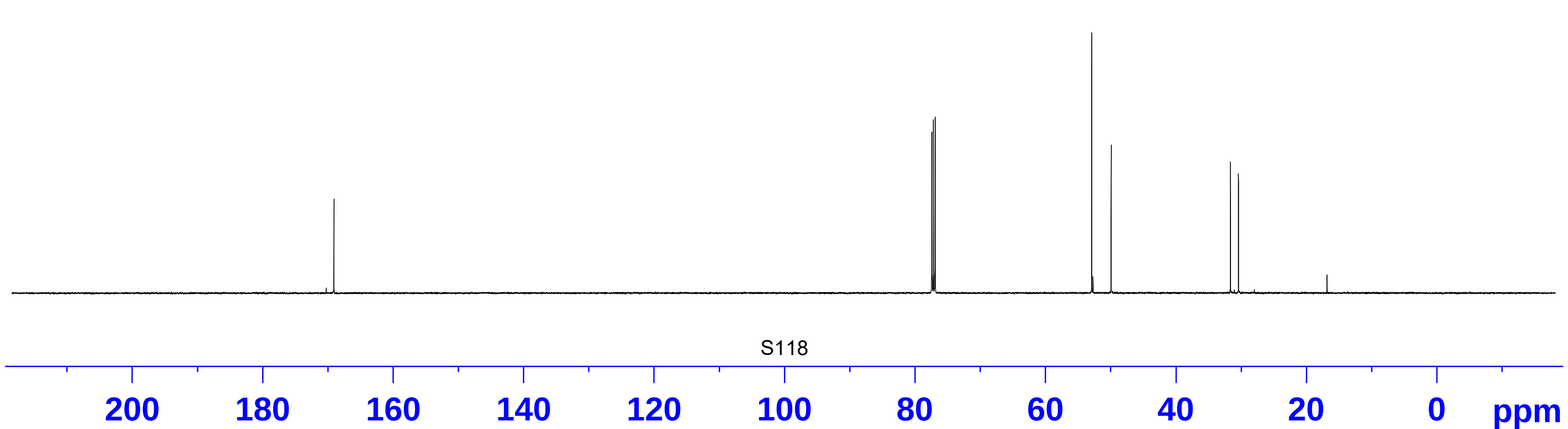

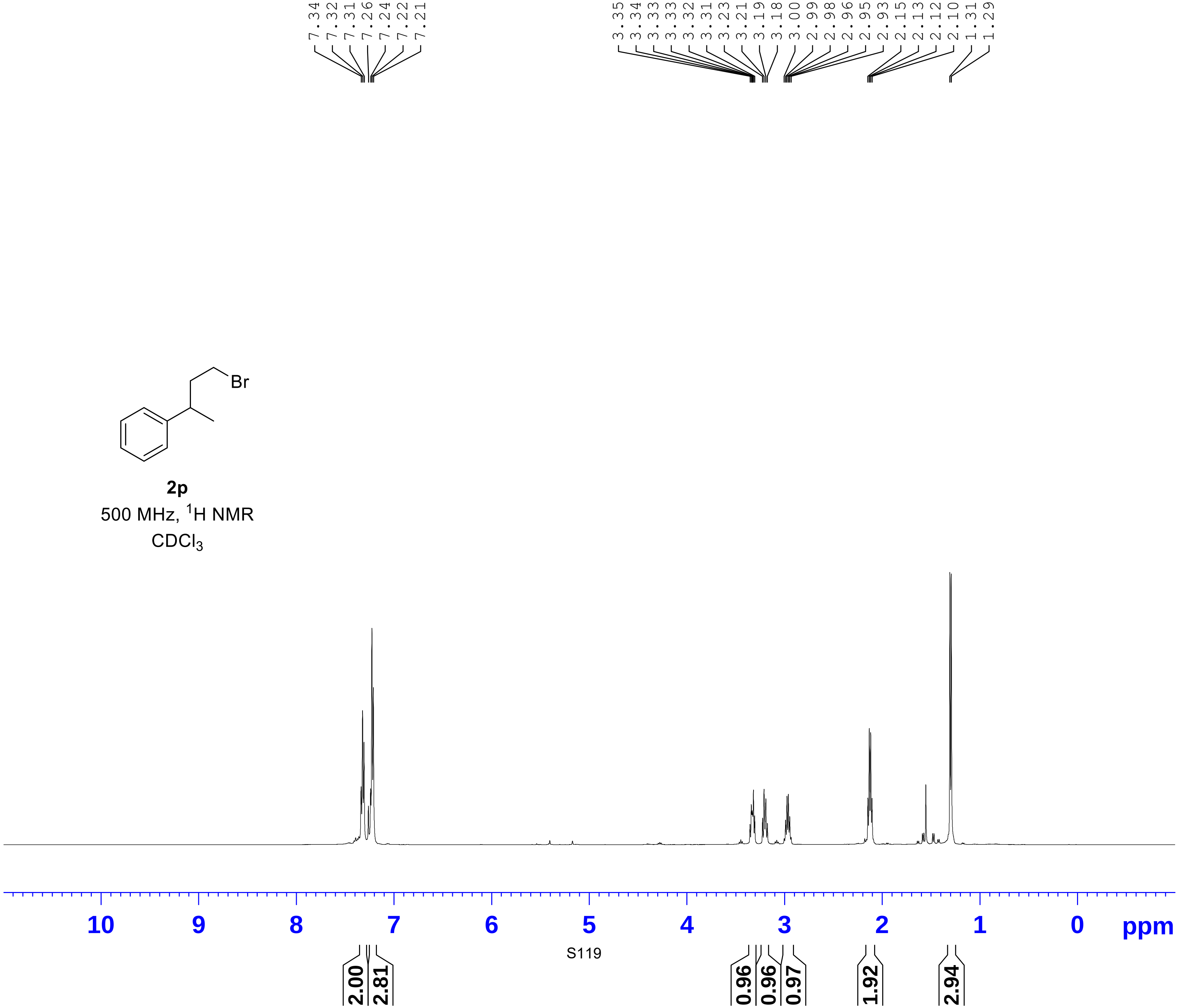
7.34
7.32
7.31
7.26
7.24
7.22
7.21
3.35
3.34
3.33
3.33
3.32
3.31
3.23
3.21
3.19
3.18
3.00
2.99
2.98
2.96
2.95
2.93
2.15
2.13
2.12
2.10
1.31
1.29
Br
2p
500 MHz, ^{1}H NMR
$CDCl_3$
10
9
8
7
6
5
4
3
2
1
0
ppm
2.00
2.81
0.96
0.96
0.97
1.92
2.94

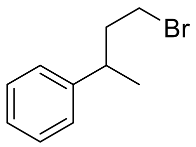

2p
125.7 MHz, ^{13}C NMR
$CDCl_3$

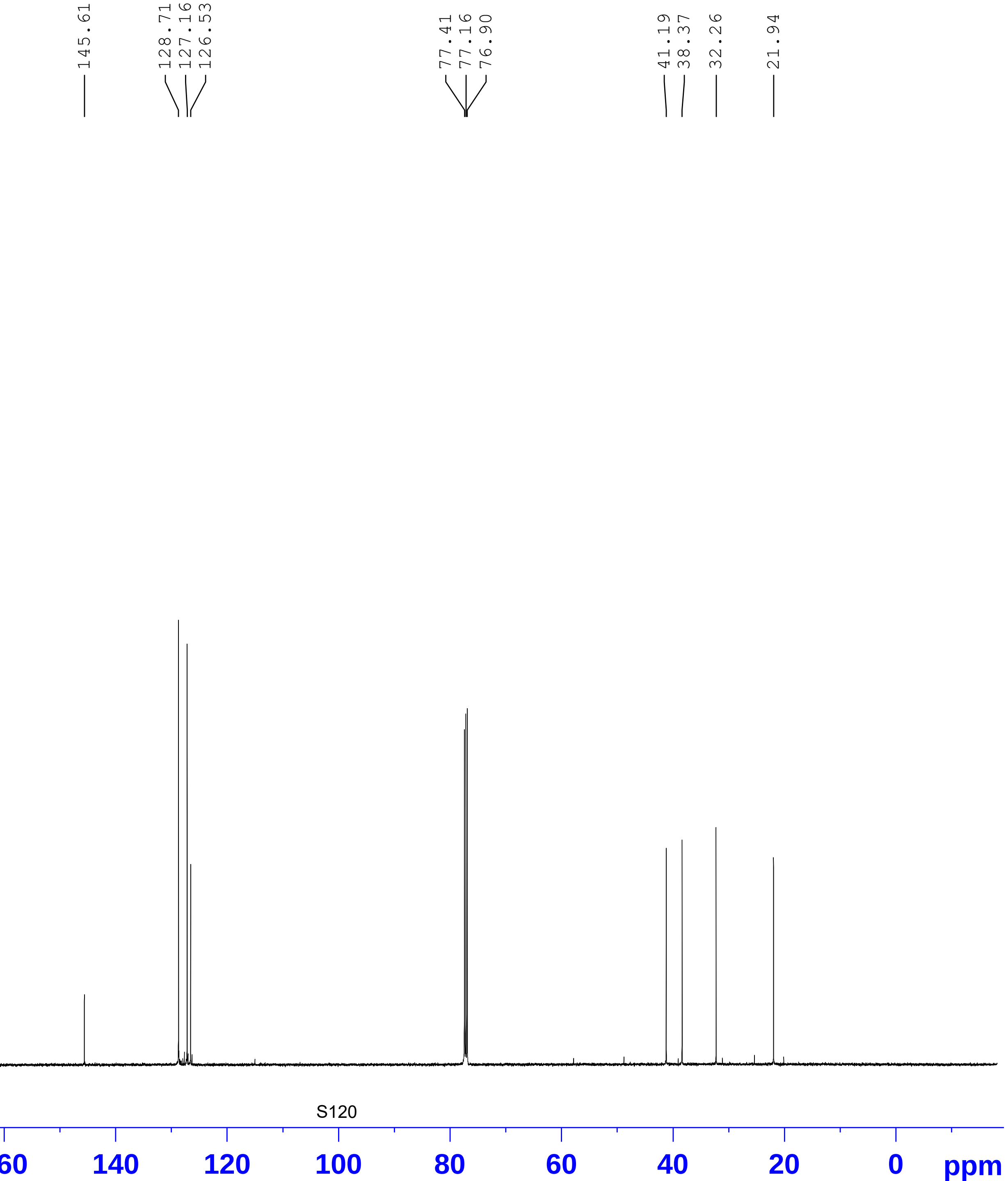

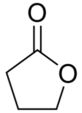

γ-butyrolactone
500 MHz, ¹H NMR
$CDCl_3$

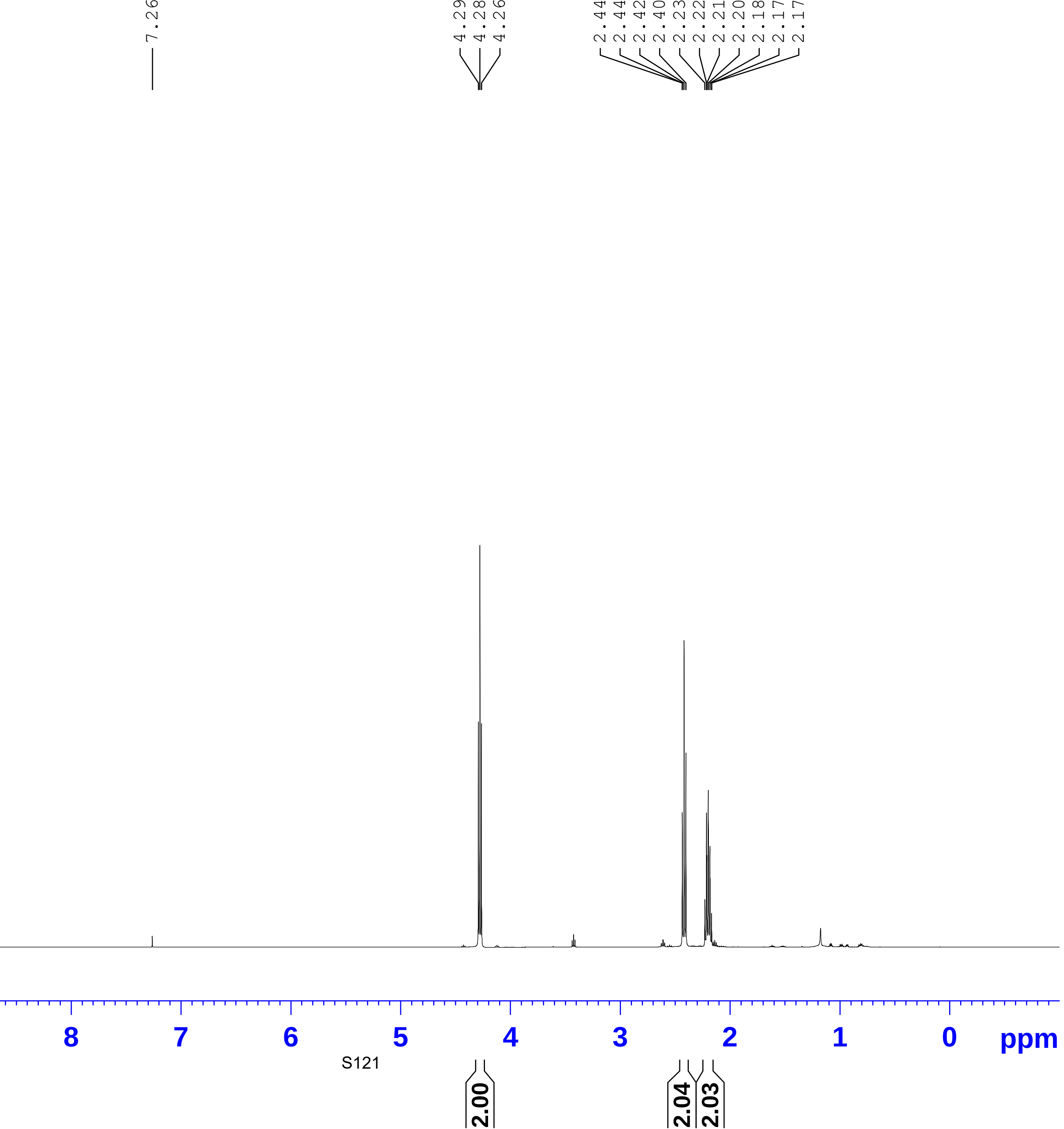

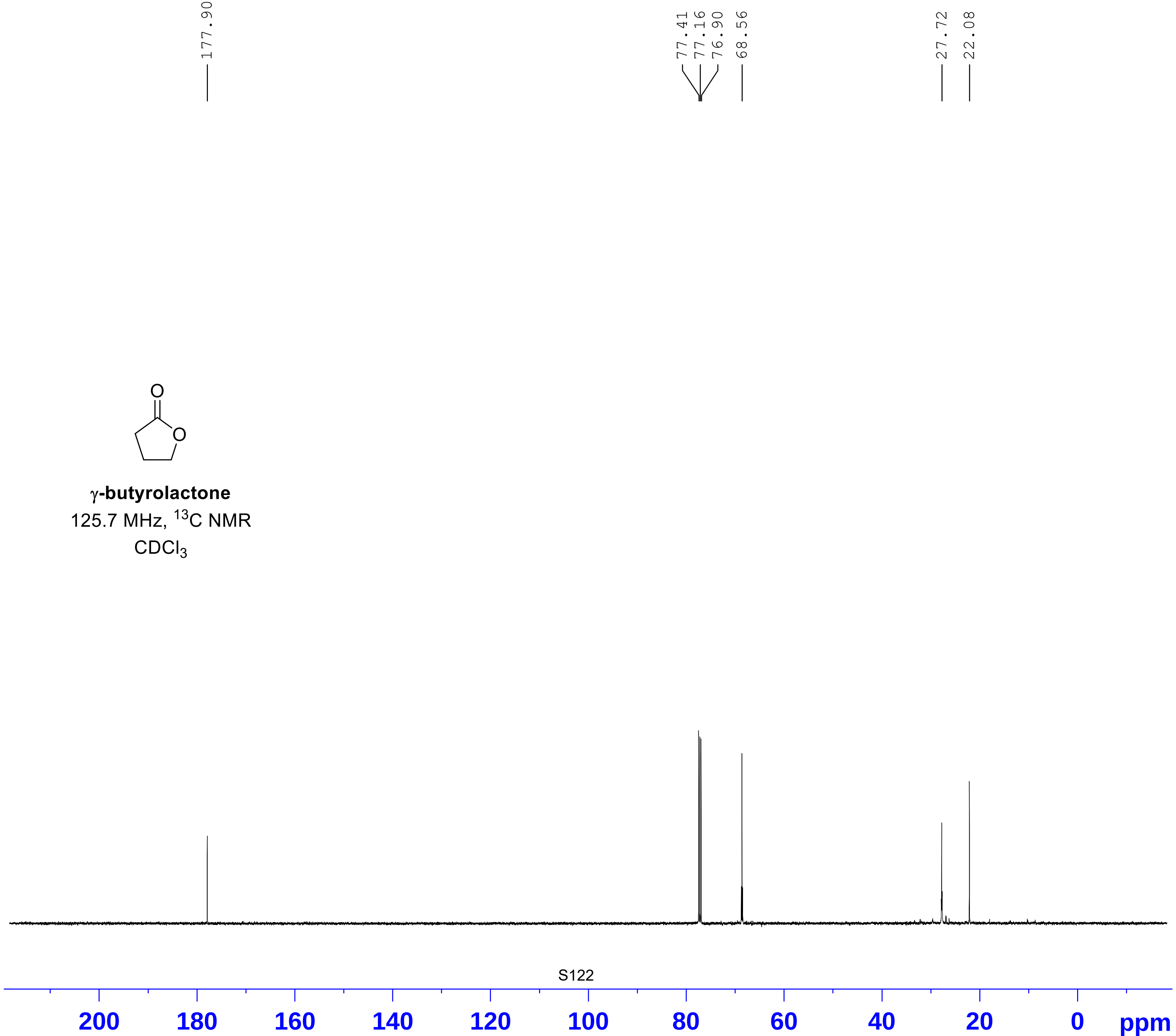
177.90
77.41
77.16
76.90
68.56
27.72
22.08
O
O
γ-butyrolactone
125.7 MHz, 13C NMR
CDCl3
200
180
160
140
120
100
80
60
40
20
0
ppm

indene-cp+BBr3-pdt

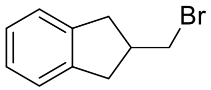

2s
500 MHz, ^{1}H NMR
$CDCl_3$

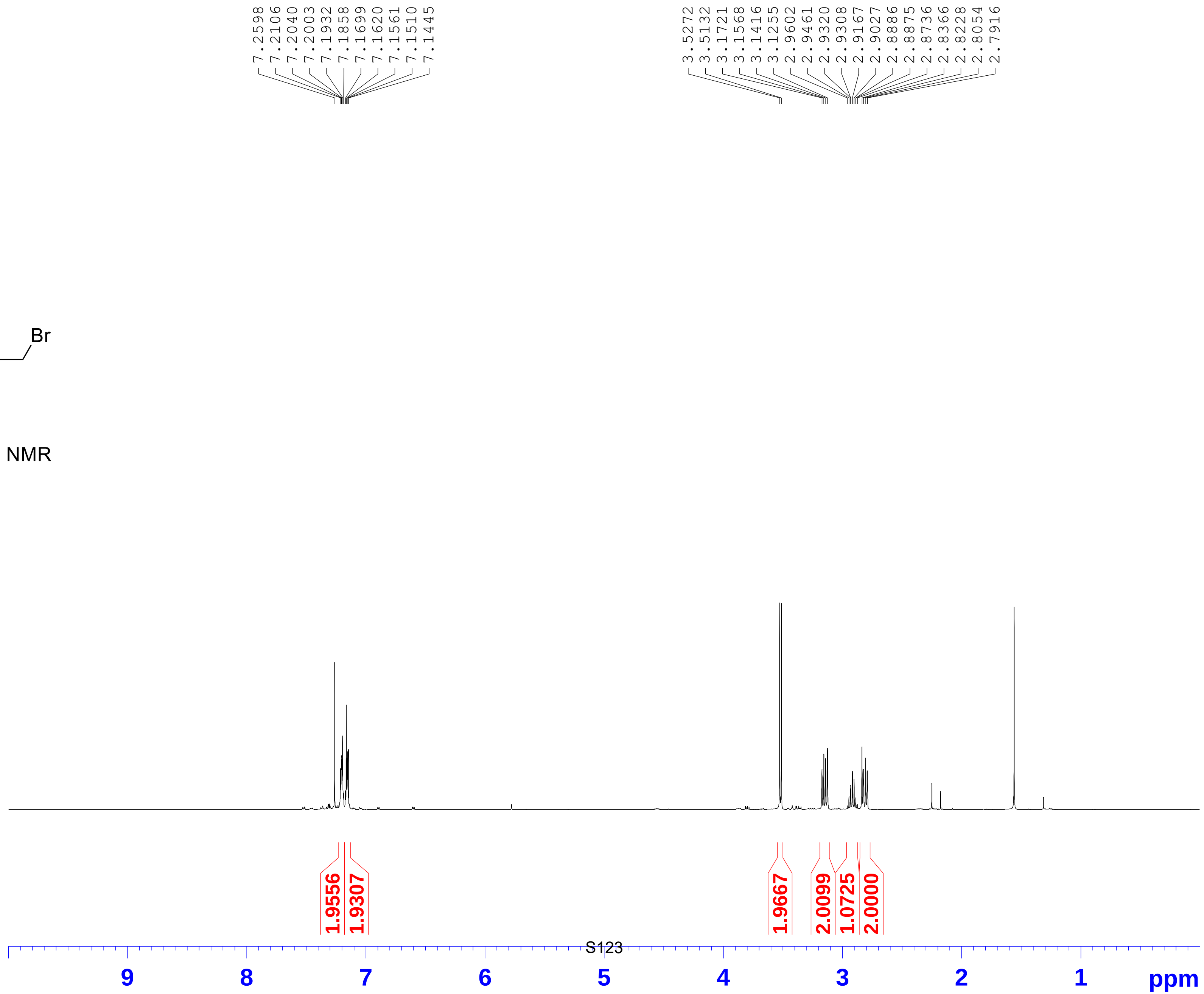

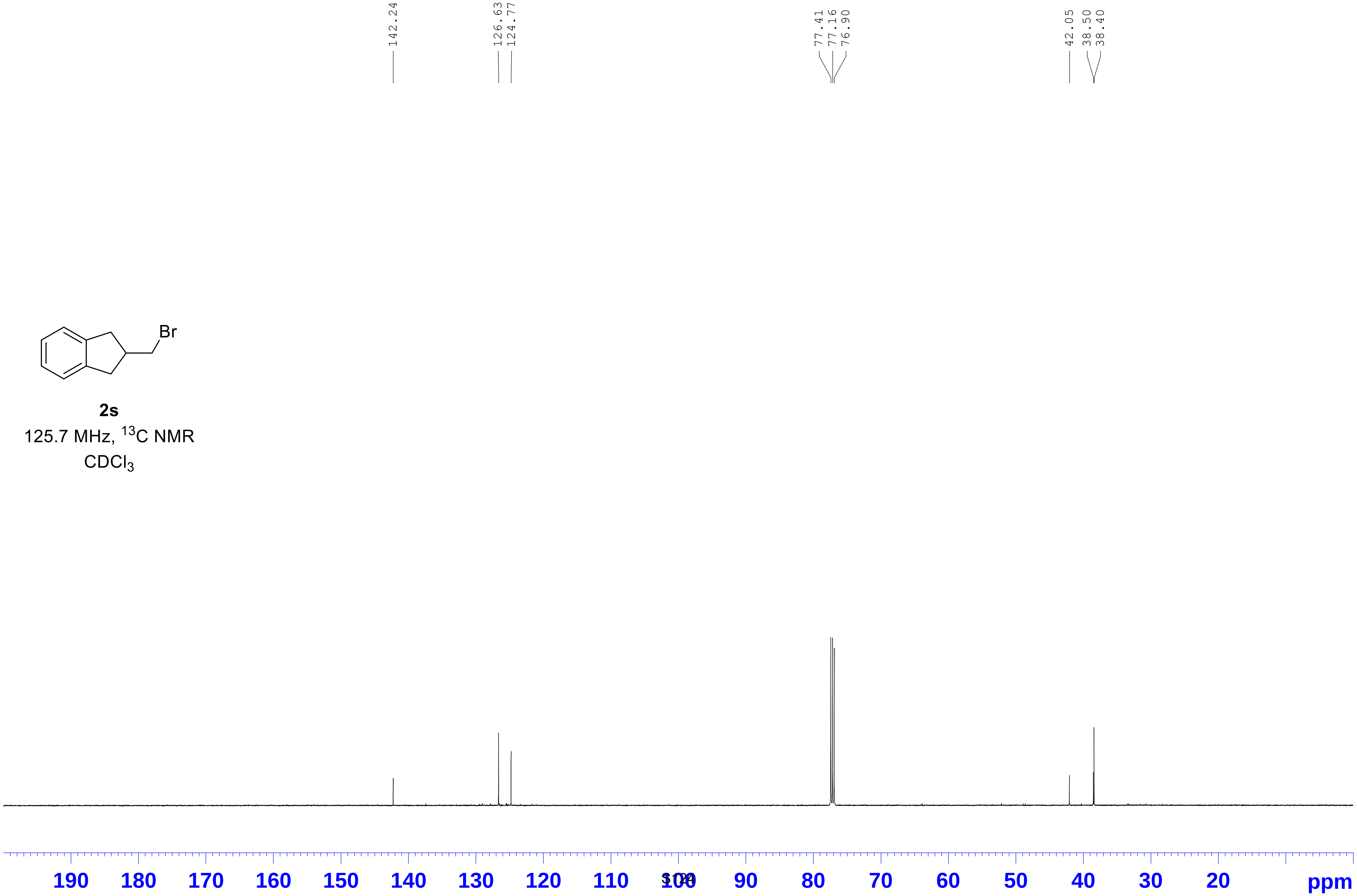

2s

125.7 MHz, ^{13}C NMR

$CDCl_3$

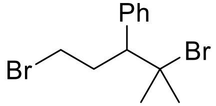

2t
500 MHz, ^{1}H NMR
$CDCl_3$

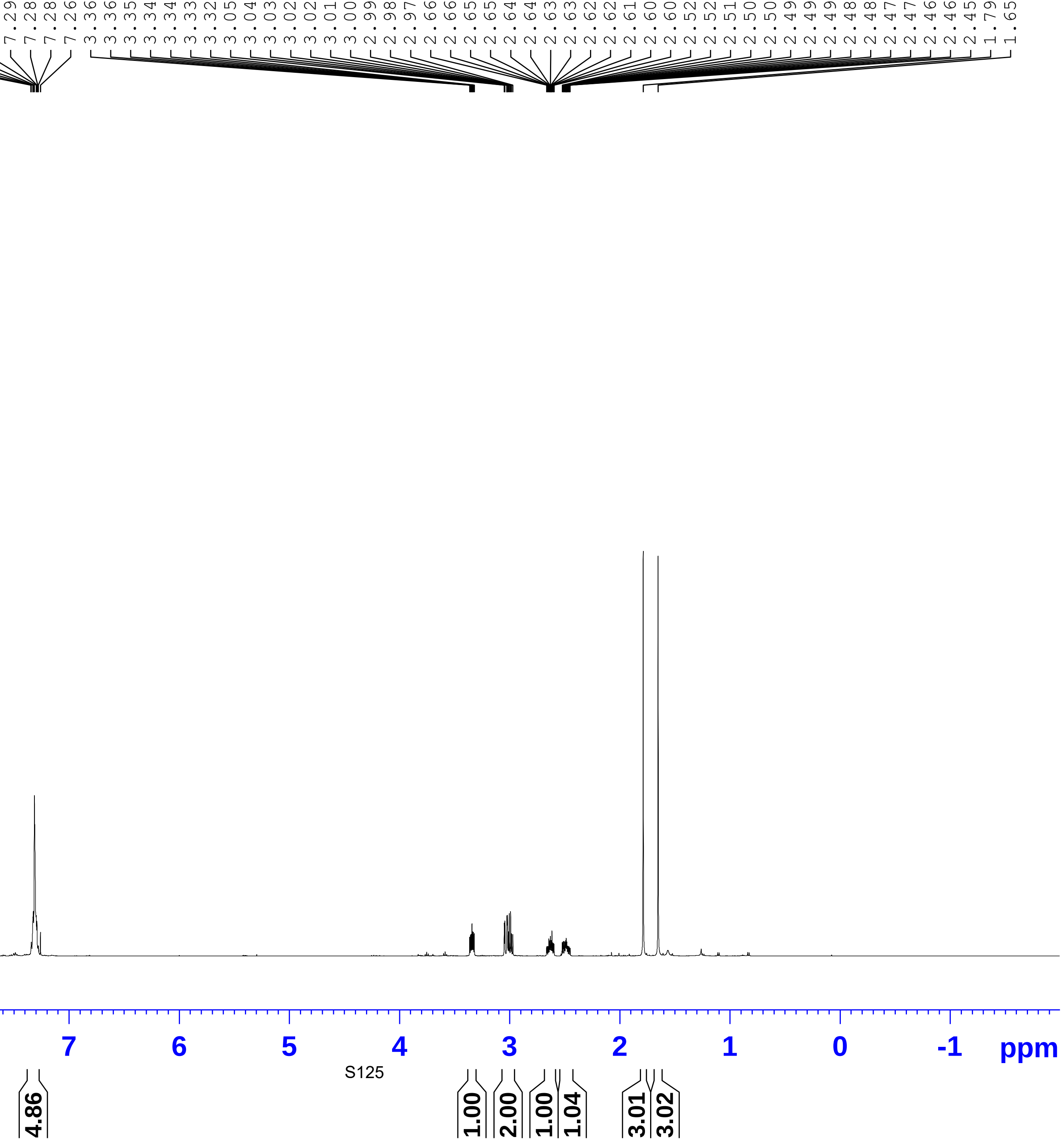

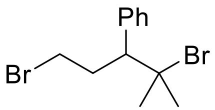

2t

125.7 MHz, ^{13}C NMR

$CDCl_3$

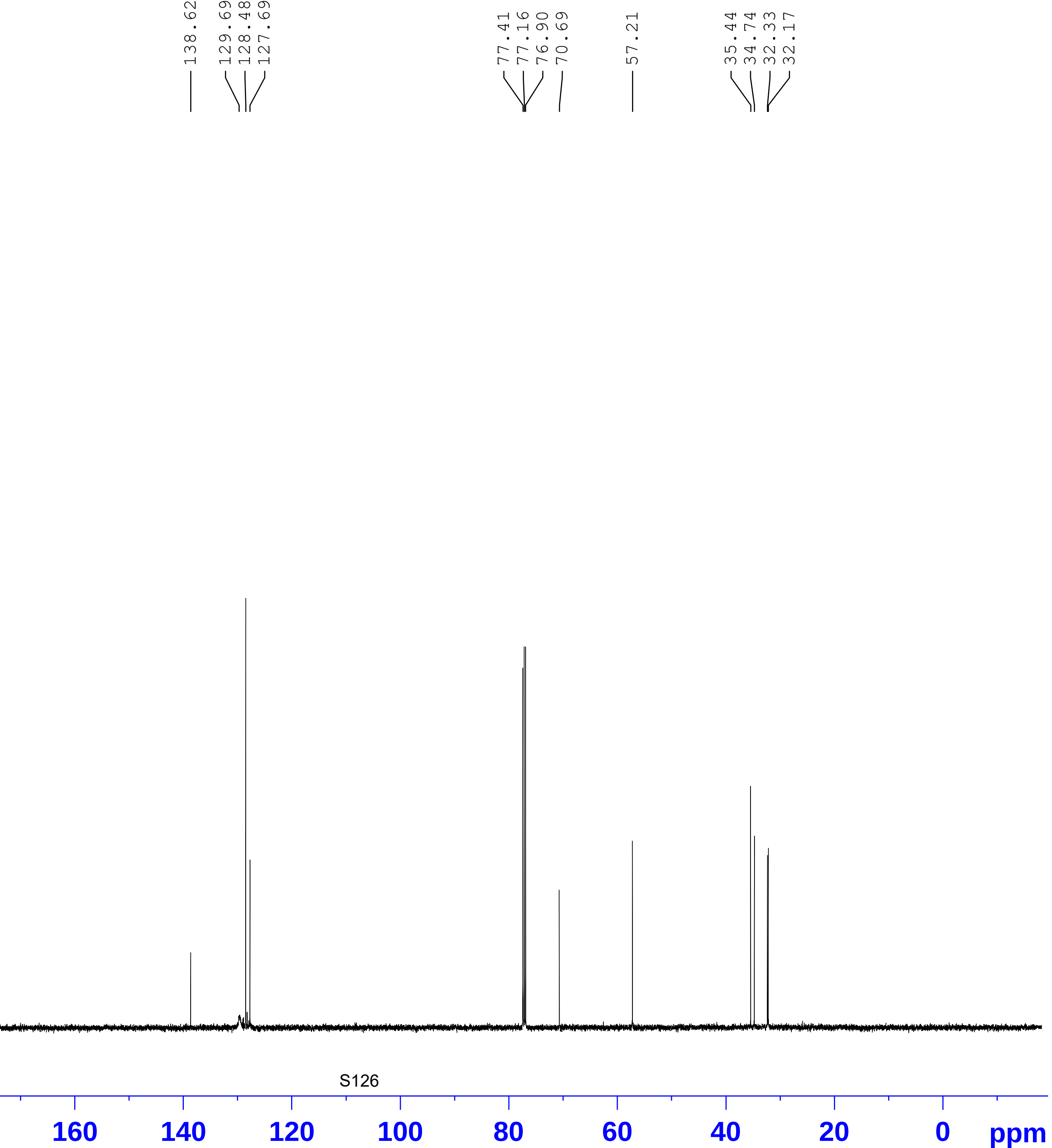

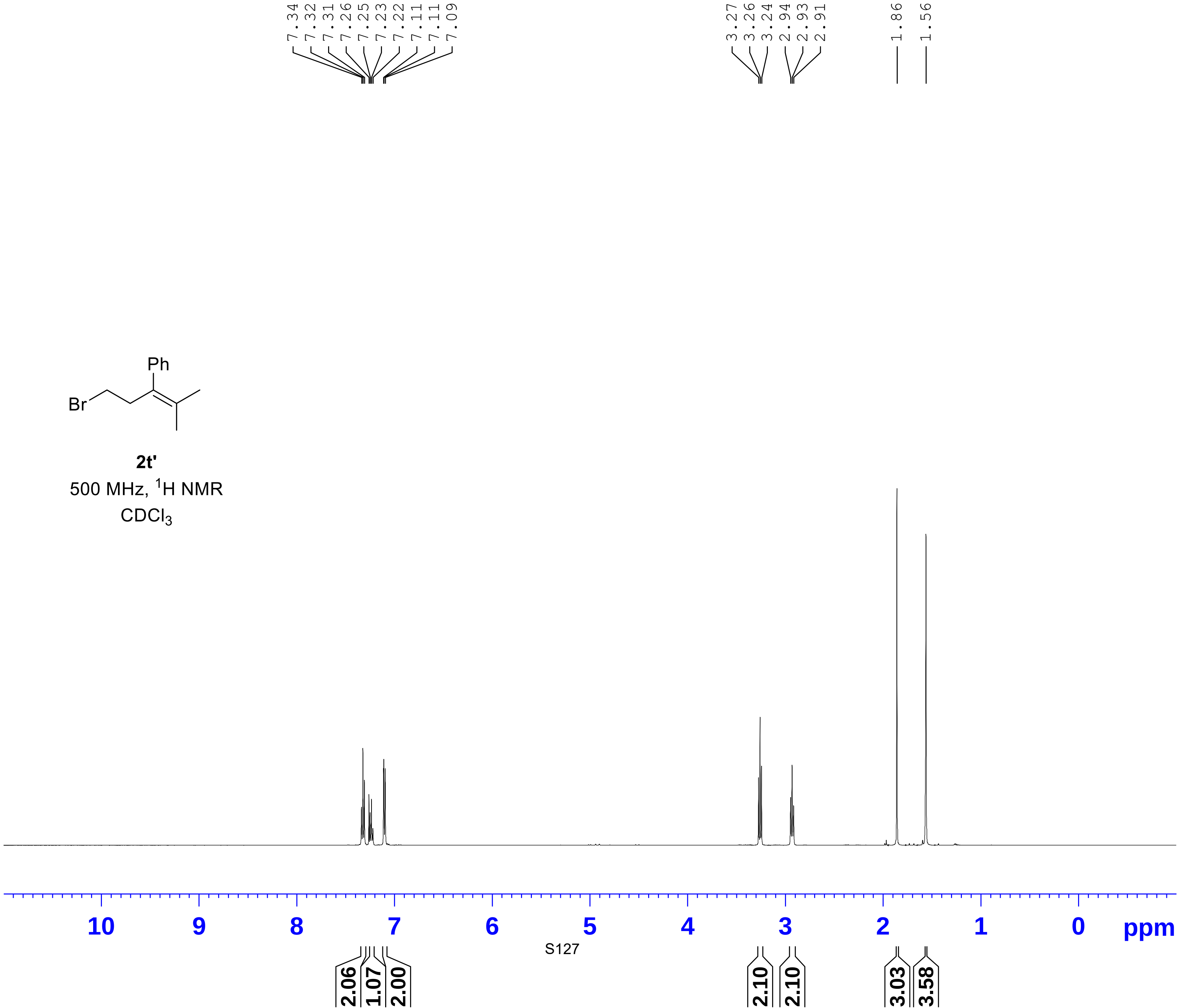

2t'

500 MHz, ^{1}H NMR

$CDCl_3$

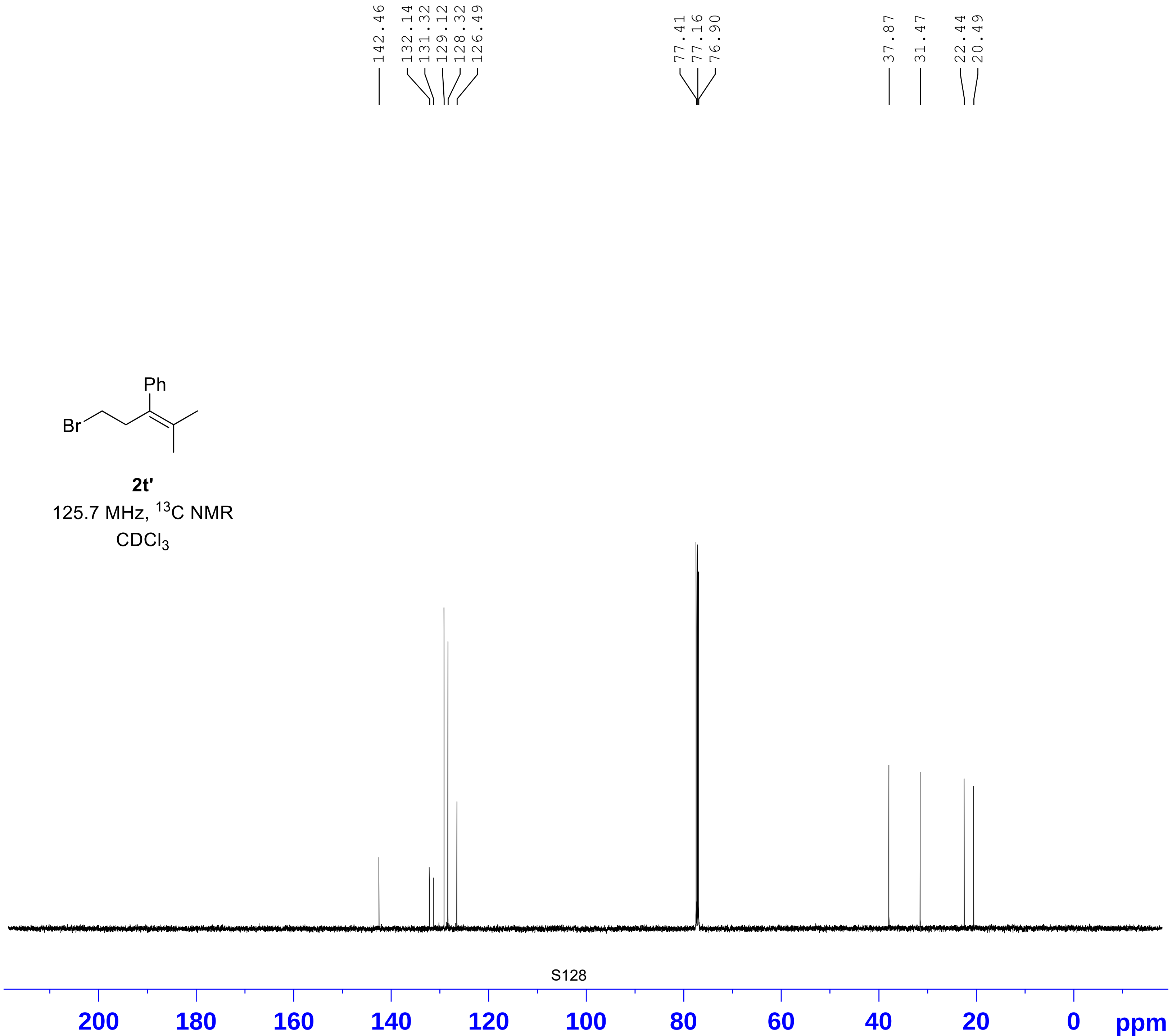
142.46
132.14
131.32
129.12
128.32
126.49
77.41
77.16
76.90
37.87
31.47
22.44
20.49
Ph
Br
2t'
125.7 MHz, ^{13}C NMR
$CDCl_3$
200
180
160
140
120
100
80
60
40
20
0
ppm

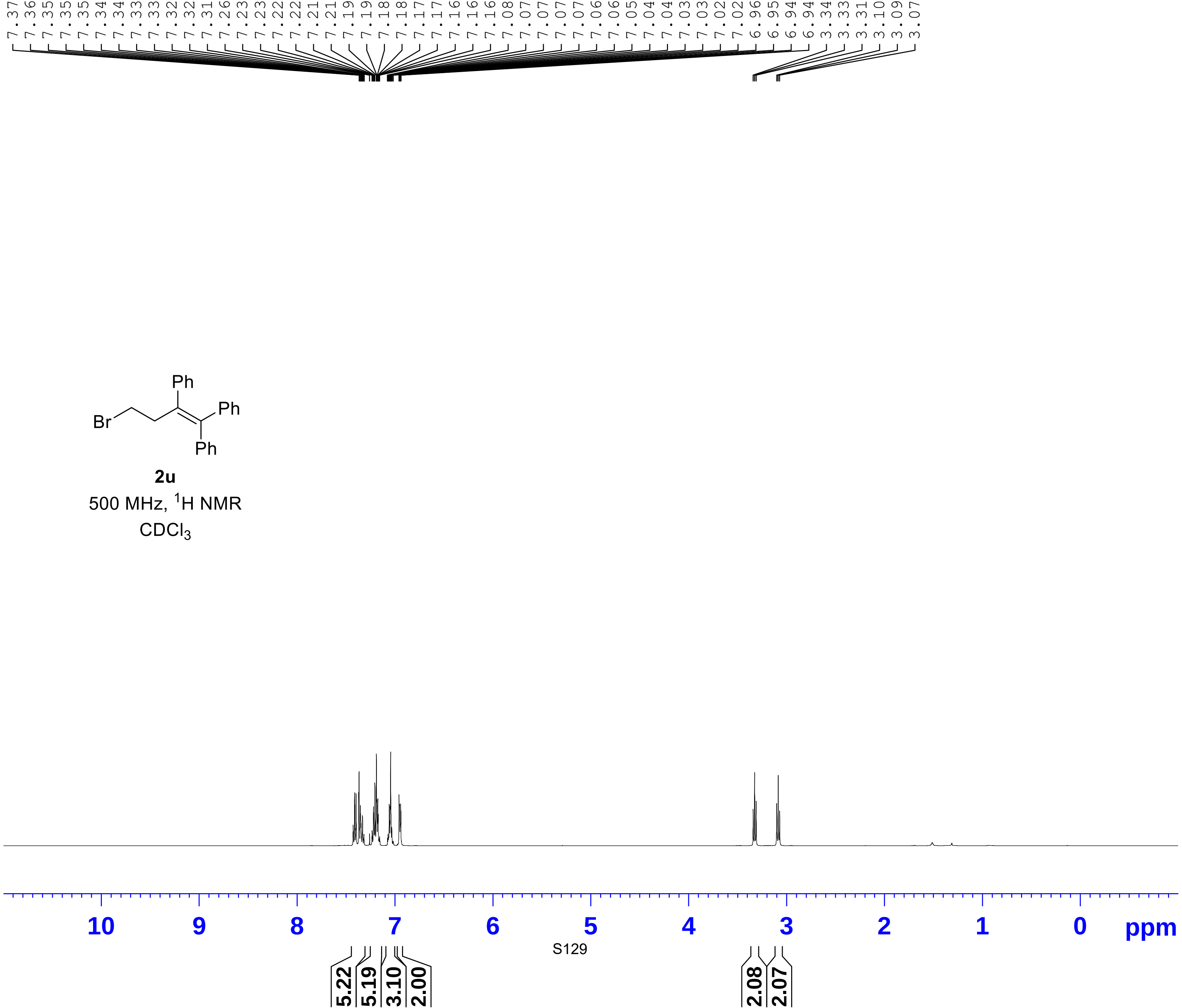

2u

500 MHz, ^{1}H NMR

$CDCl_3$

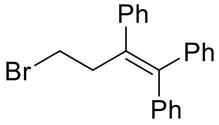

2u
125.7 MHz, ^{13}C NMR
$CDCl_3$

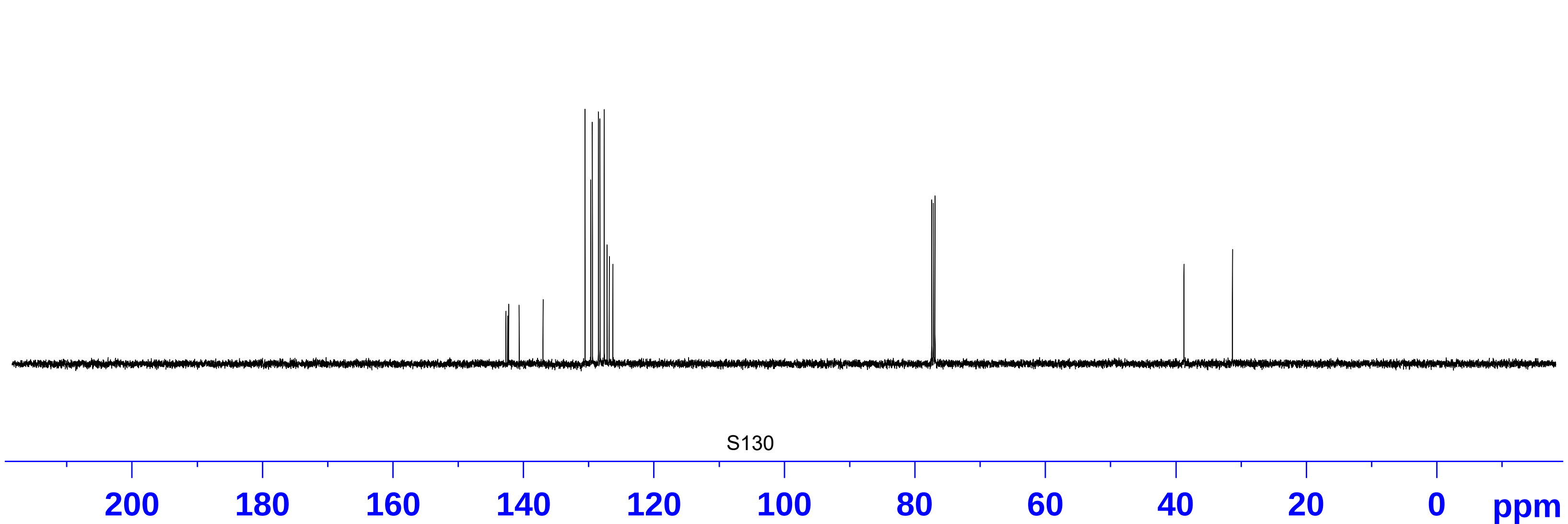

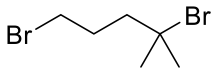

2v

500 MHz, ^{1}H NMR

$CDCl_3$

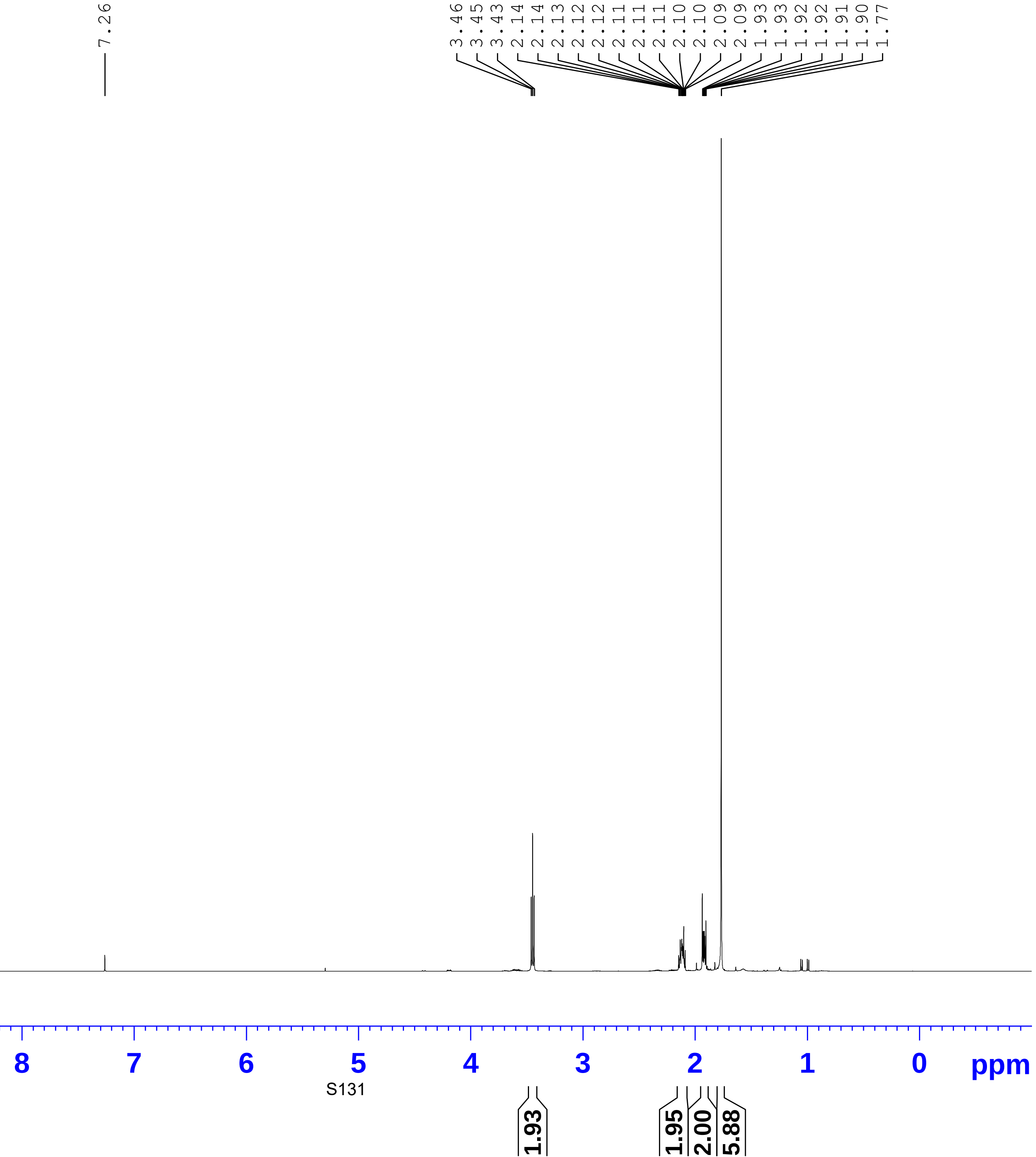

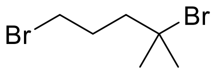

2v
125.7 MHz, ^{13}C NMR
$CDCl_3$

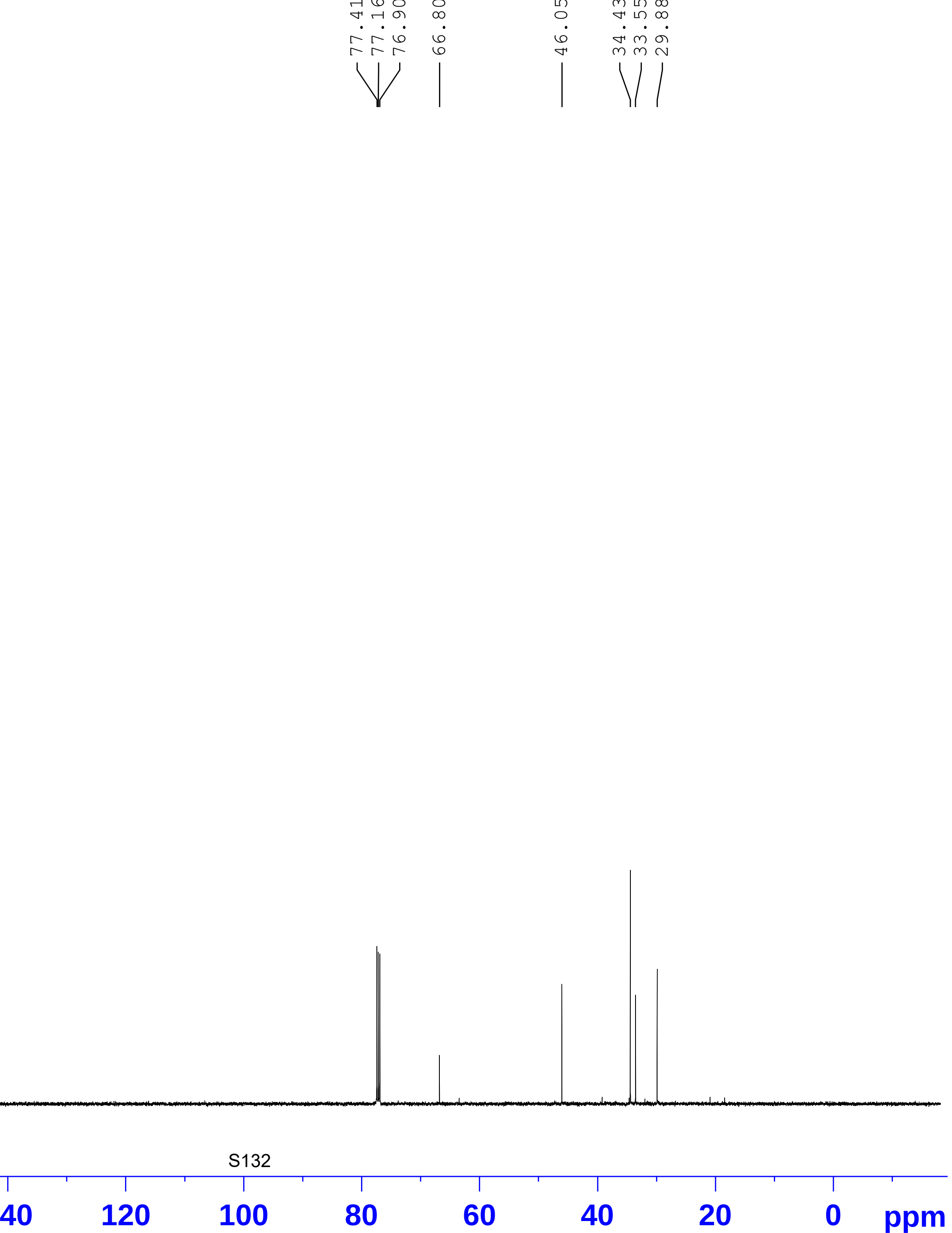

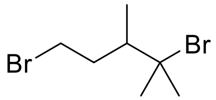

2w
500 MHz, ^{1}H NMR
$CDCl_3$

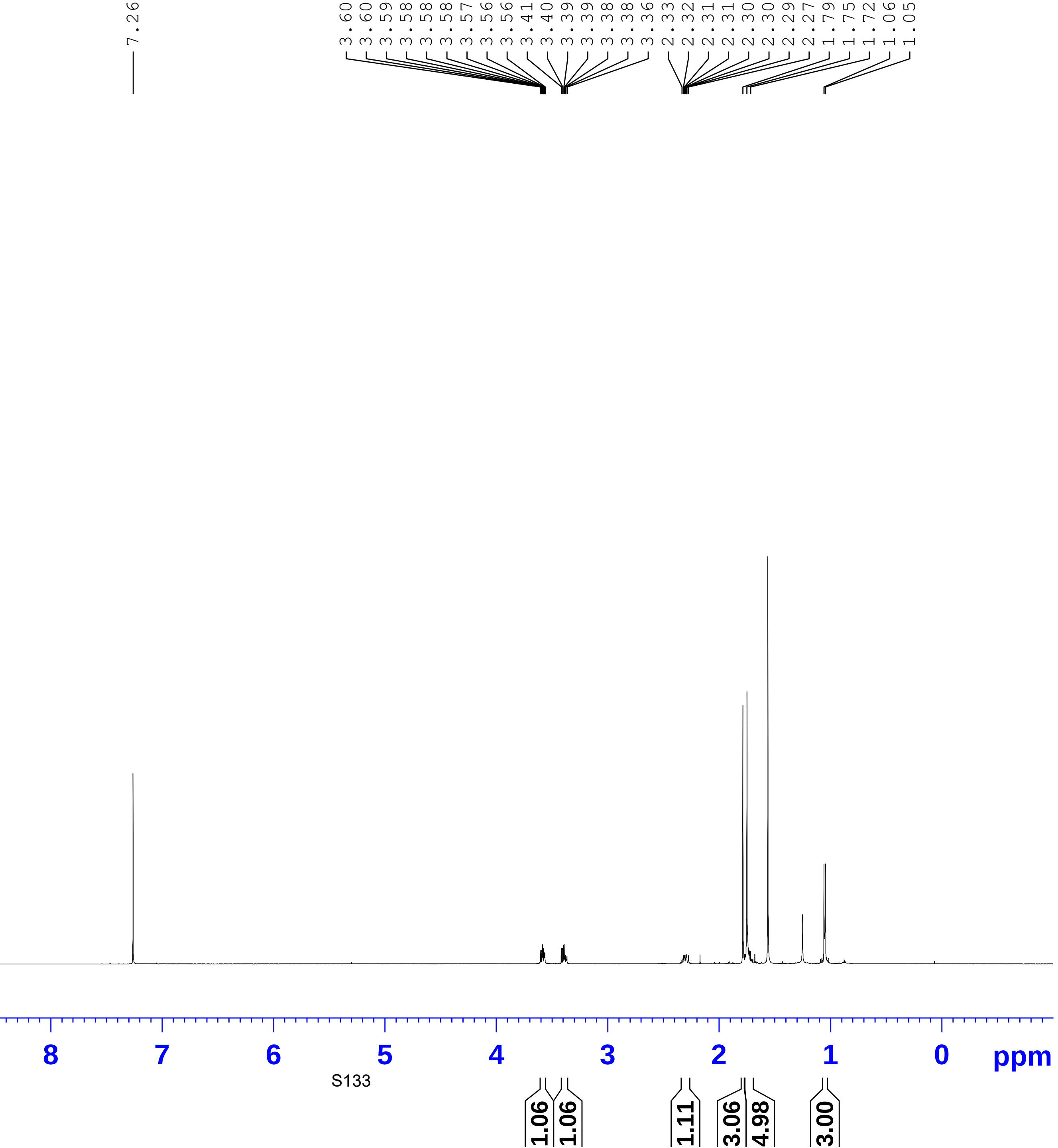

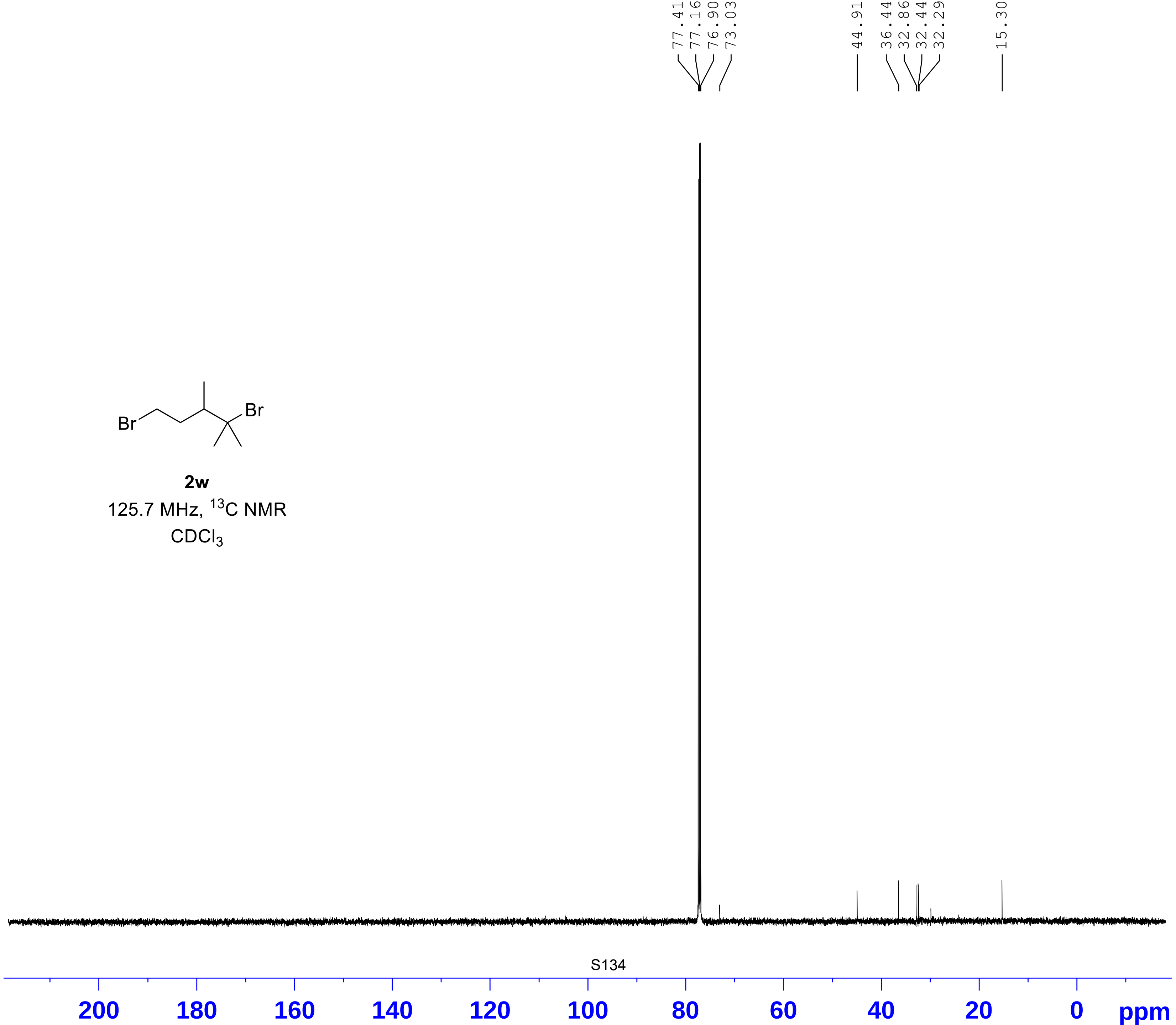

2w
125.7 MHz, ^{13}C NMR
$CDCl_3$

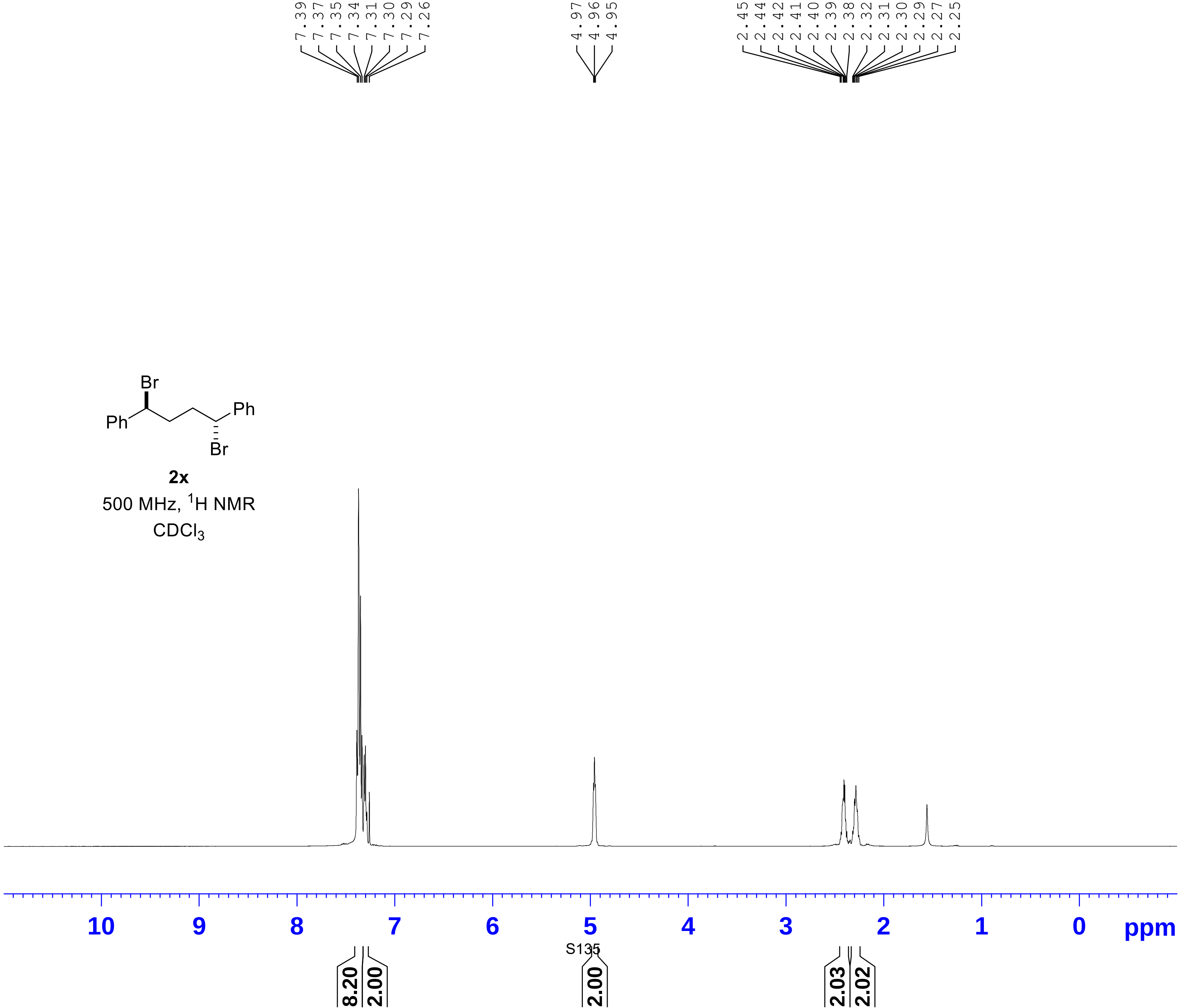
7.39
7.37
7.35
7.34
7.31
7.30
7.29
7.26
4.97
4.96
4.95
2.45
2.44
2.42
2.41
2.40
2.39
2.38
2.32
2.31
2.30
2.29
2.27
2.25
Br
Ph
Ph
Br
2x
500 MHz, 1H NMR
CDCl3
10
9
8
7
6
5
4
3
2
1
0
ppm
8.20
2.00
2.00
2.03
2.02

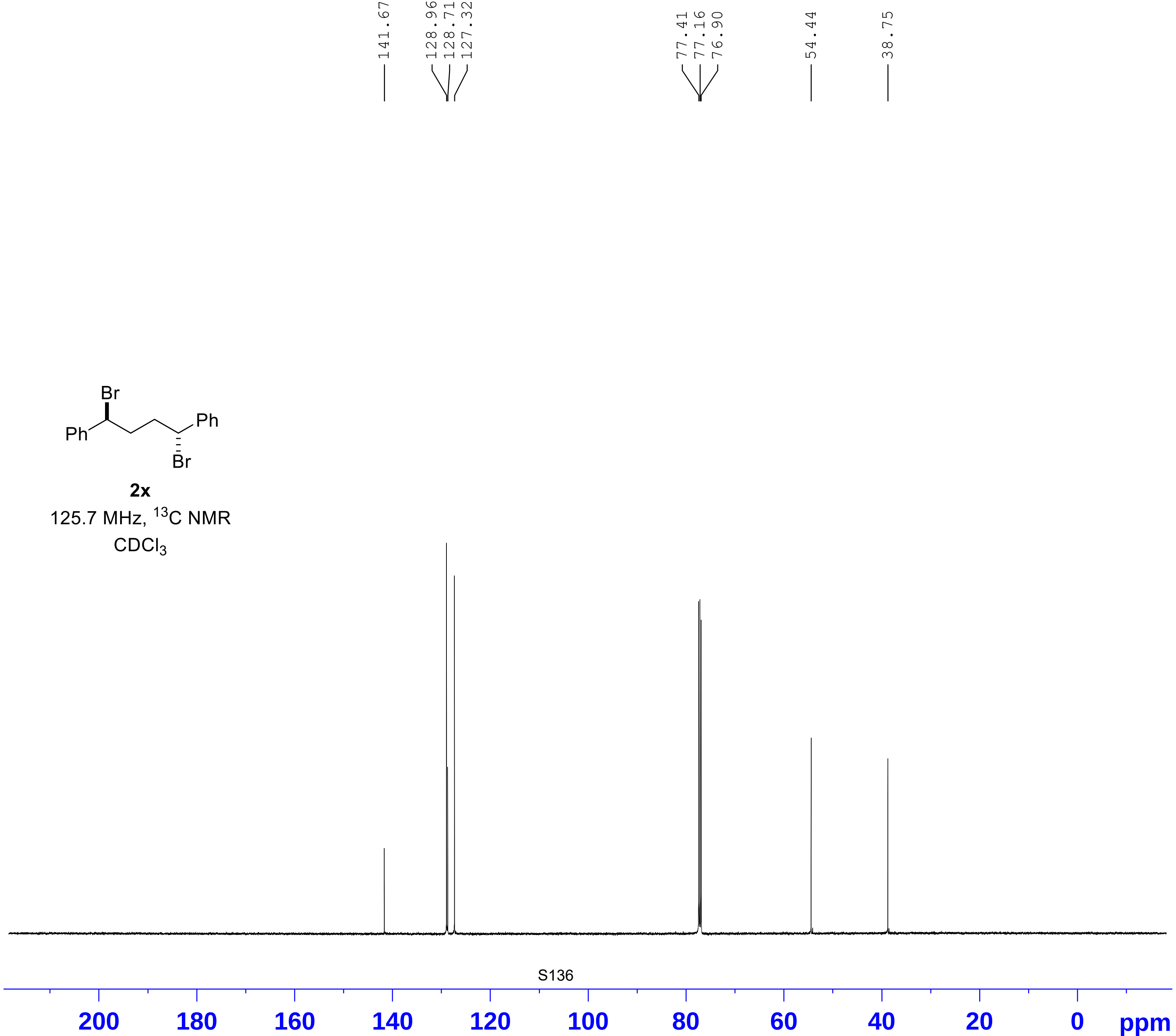

2x

125.7 MHz, ^{13}C NMR

$CDCl_3$

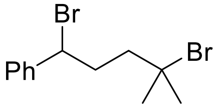

2y
500 MHz, ^{1}H NMR
$CDCl_3$

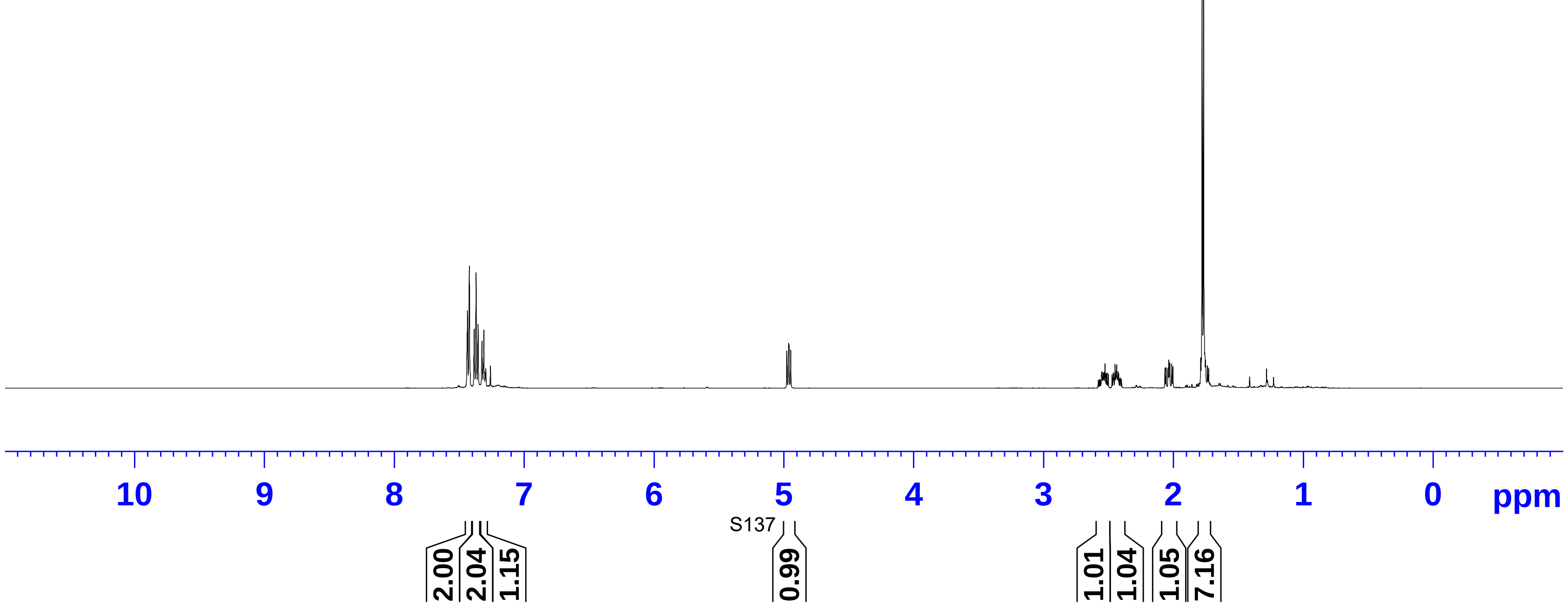

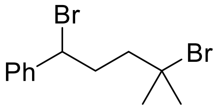

2y
125.7 MHz, ^{13}C NMR
$CDCl_3$

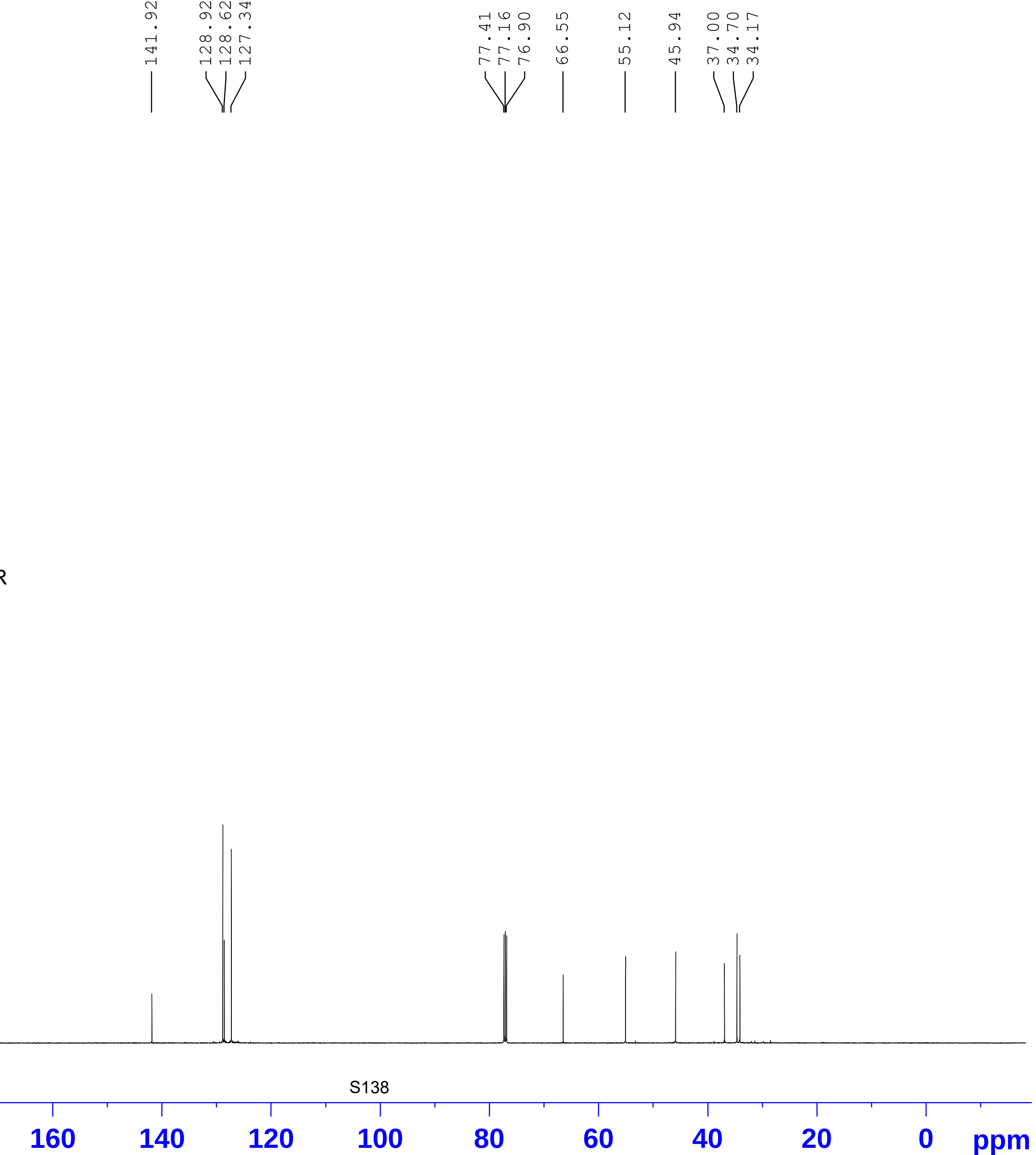

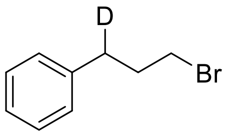

2a-*D*
500 MHz, ^{1}H NMR
$CDCl_3$

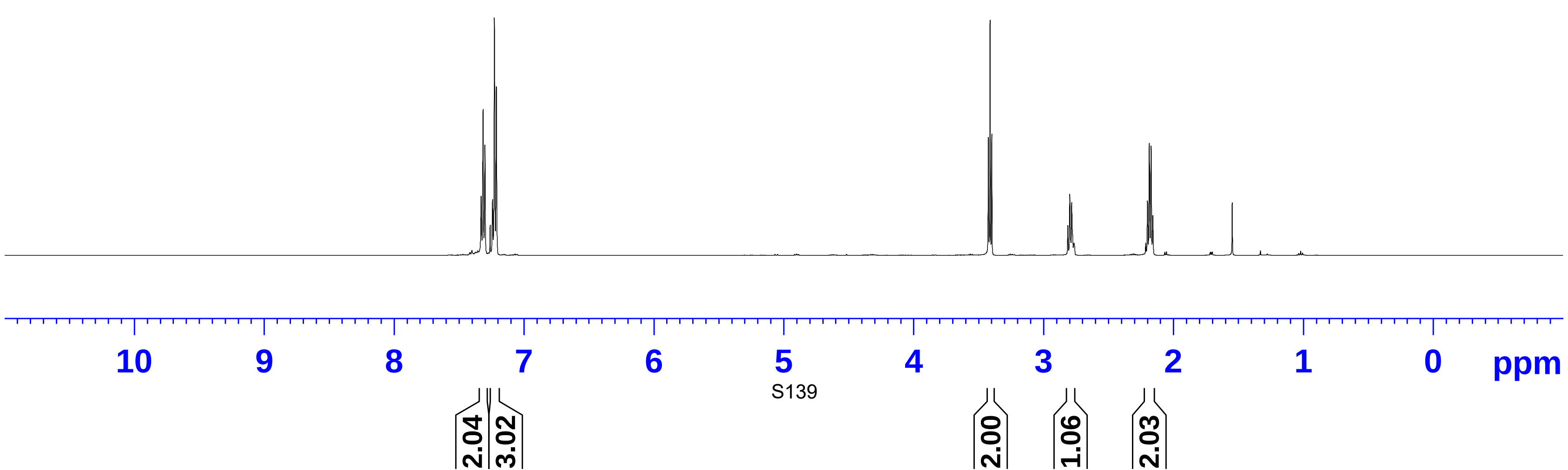

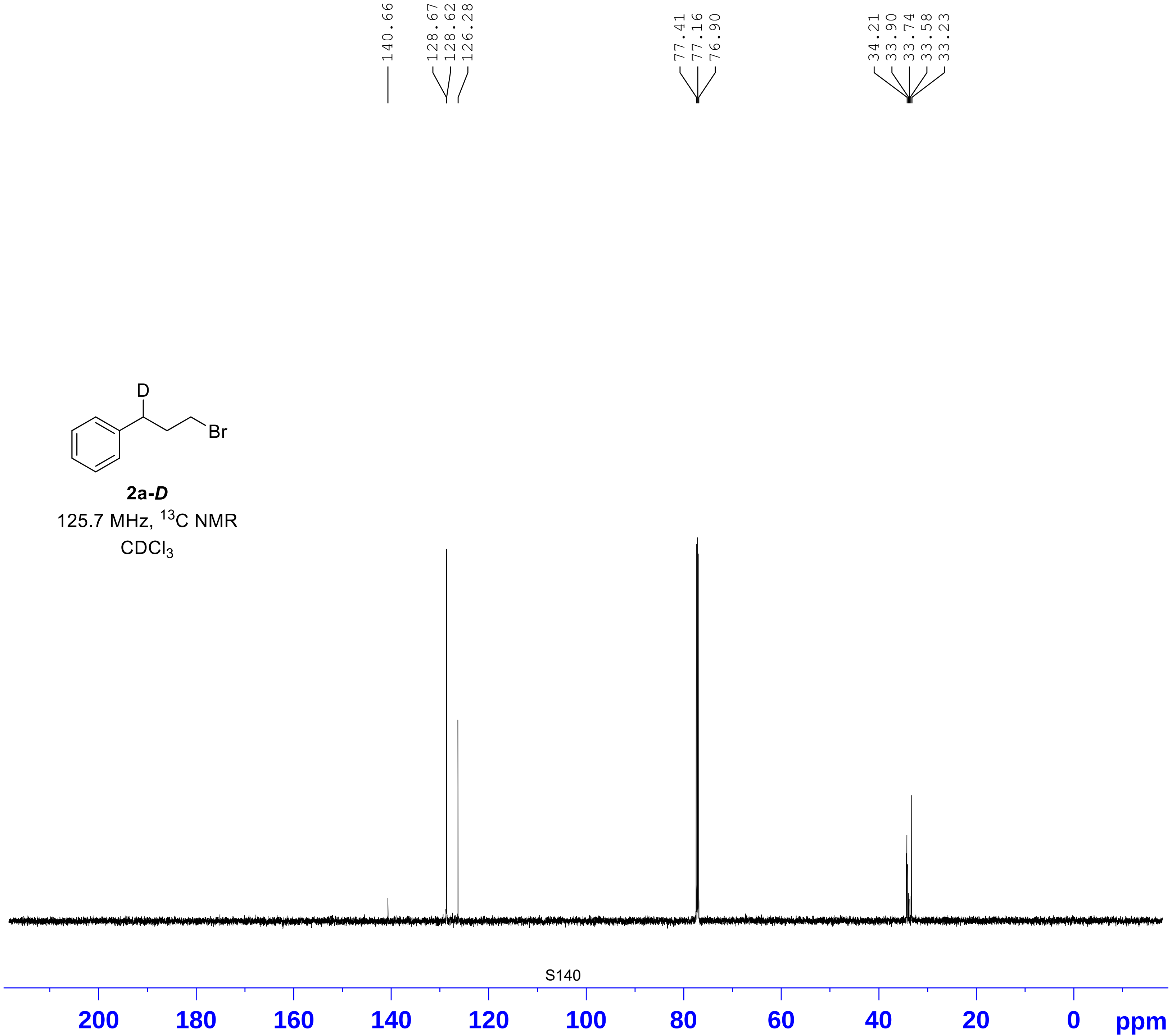

2a-*D*
125.7 MHz, ^{13}C NMR
$CDCl_3$

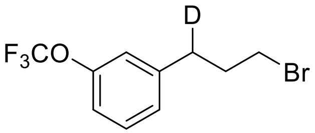

2e-*D*
500 MHz, ^1H NMR
$CDCl_3$

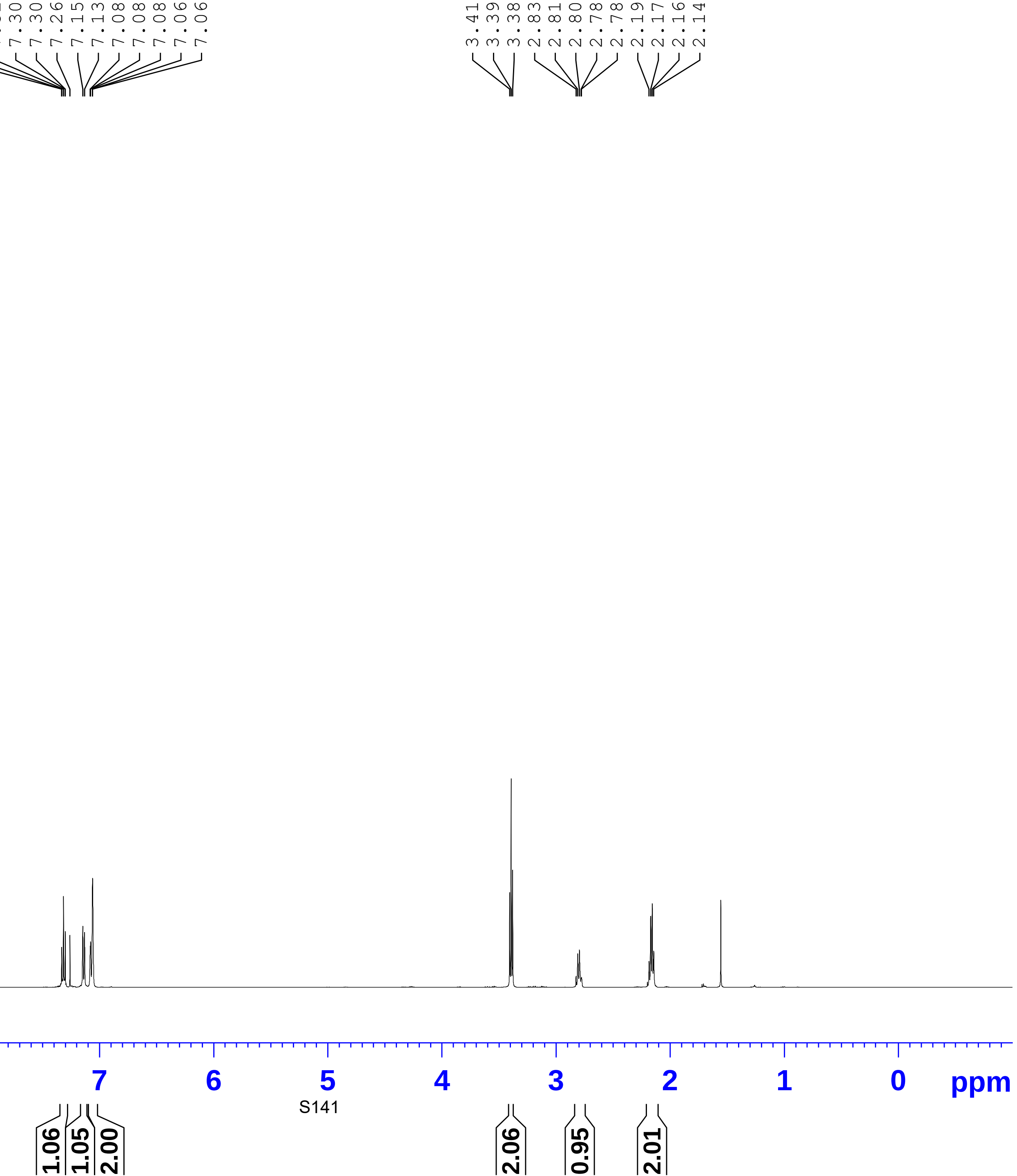

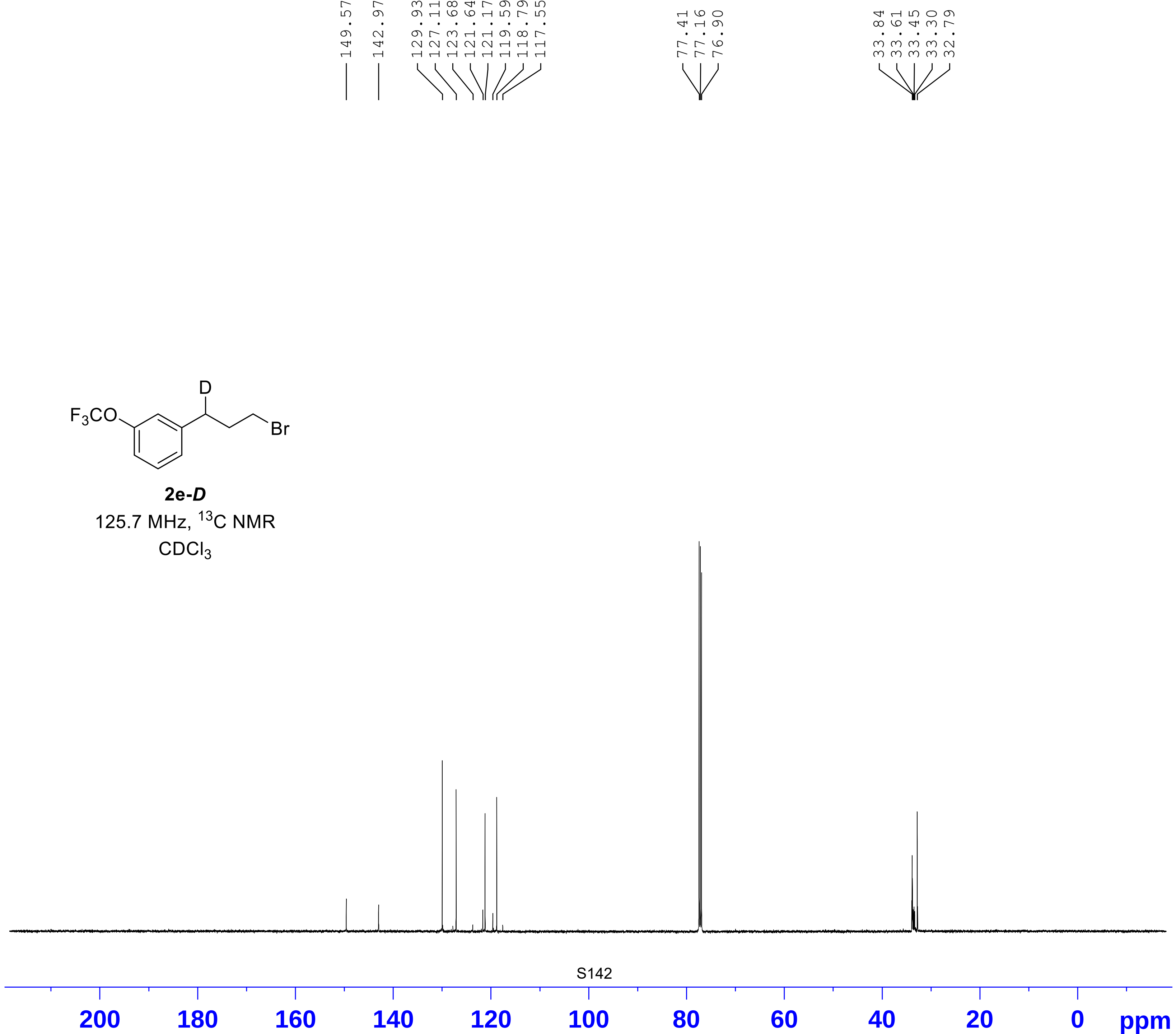
149.57
142.97
129.93
127.11
123.68
121.64
121.17
119.59
118.79
117.55
77.41
77.16
76.90
33.84
33.61
33.45
33.30
32.79
D
F3CO
Br
2e-D
125.7 MHz, 13C NMR
CDCl3
200 180 160 140 120 100 80 60 40 20 0 ppm

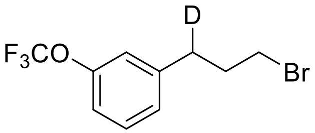

2e-*D*
470.6 MHz, ^{19}F NMR
$CDCl_3$

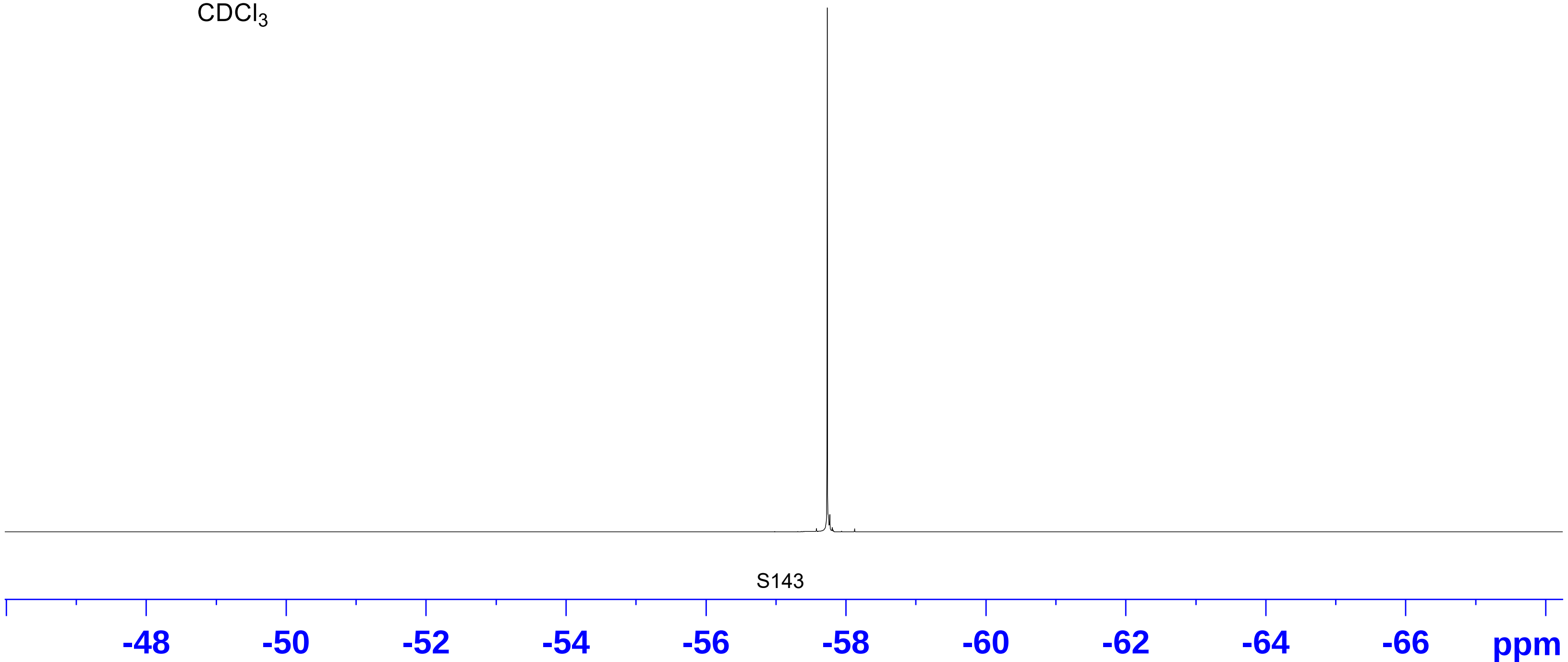

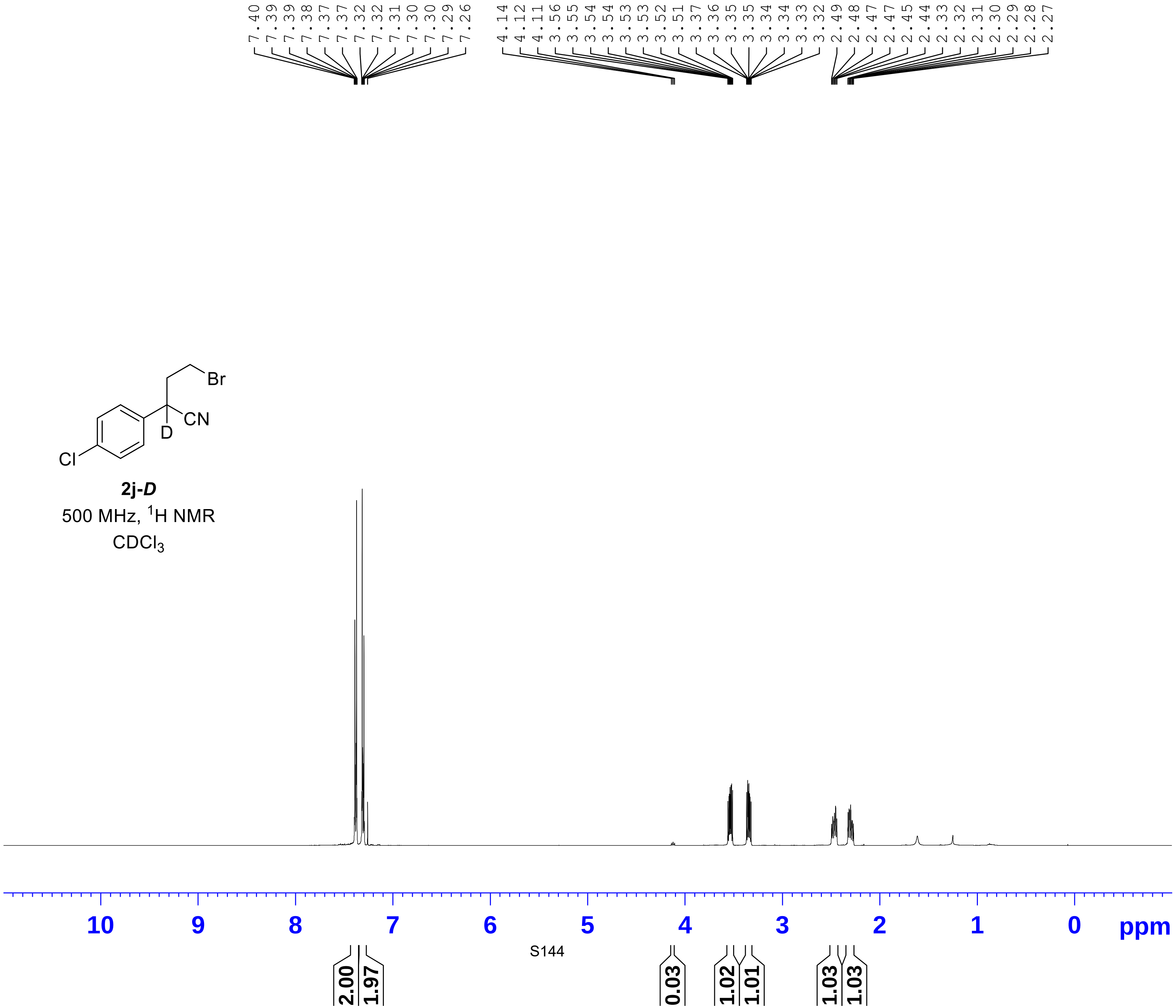
7.40 7.39 7.39 7.38 7.37 7.37 7.32 7.32 7.31 7.30 7.30 7.29 7.26
4.14 4.12 4.11 3.56 3.55 3.54 3.54 3.53 3.53 3.52 3.51 3.37 3.36 3.35 3.35 3.34 3.34 3.33 3.32
2.49 2.48 2.47 2.47 2.45 2.44 2.33 2.32 2.31 2.30 2.29 2.28 2.27
Br
CN
D
Cl
2j-D
500 MHz, 1H NMR
CDCl3
10 9 8 7 6 5 4 3 2 1 0 ppm
2.00 1.97 0.03 1.02 1.01 1.03 1.03

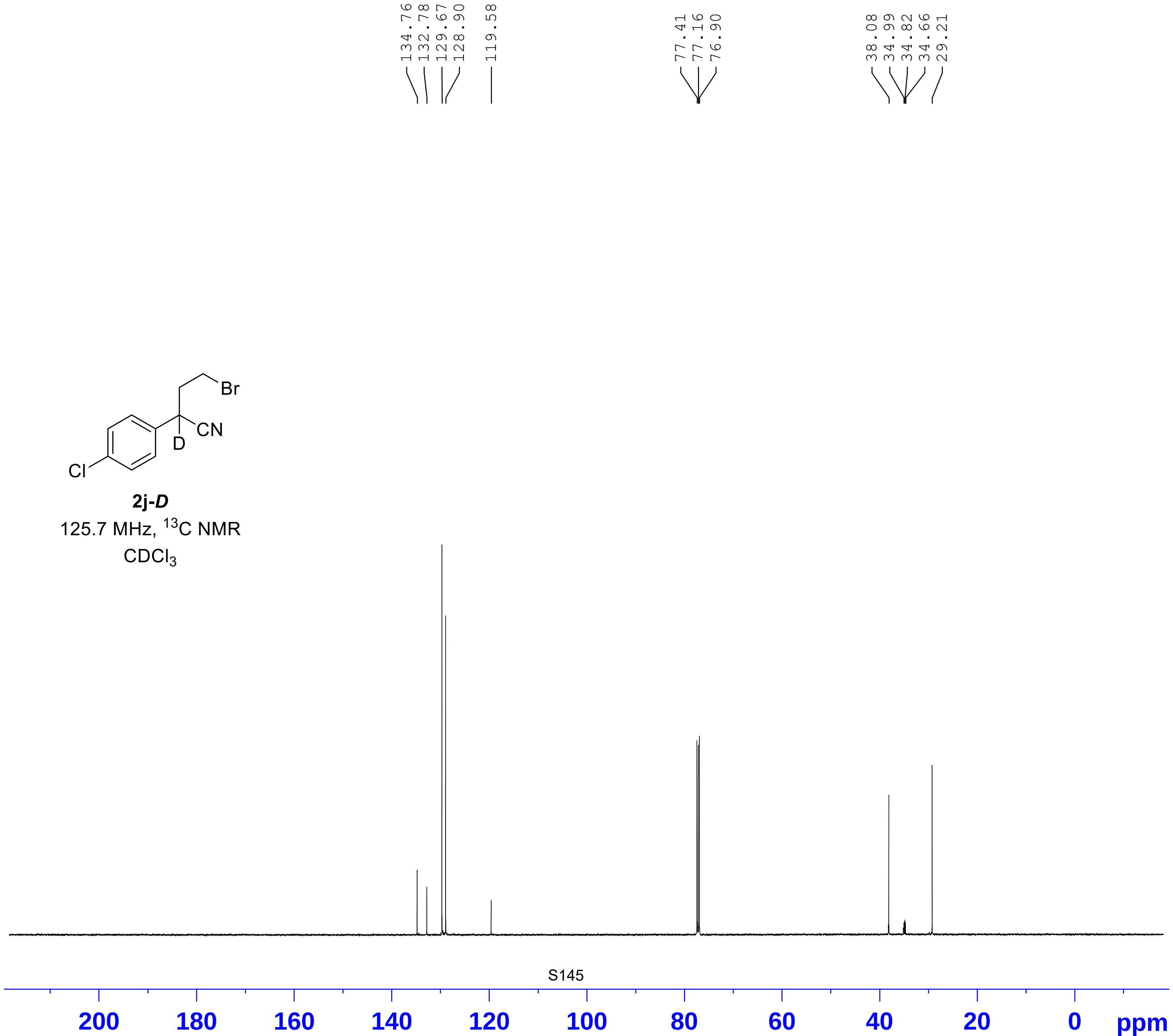

2j-*D*
125.7 MHz, ^{13}C NMR
$CDCl_3$

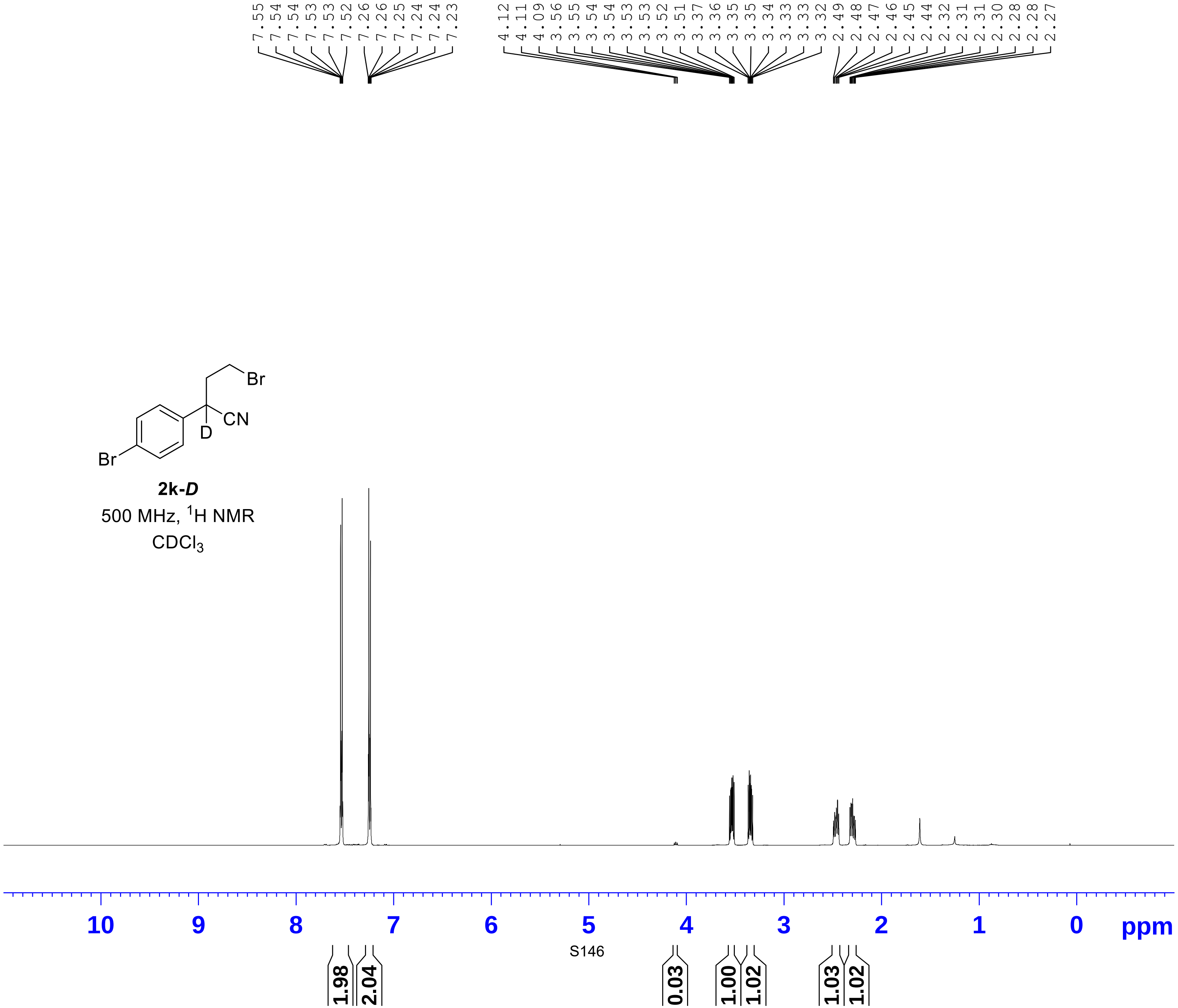
7.55
7.54
7.54
7.53
7.53
7.52
7.26
7.26
7.25
7.24
7.24
7.23
4.12
4.11
4.09
3.56
3.55
3.54
3.54
3.53
3.53
3.52
3.51
3.37
3.36
3.35
3.35
3.34
3.33
3.33
3.32
2.49
2.48
2.47
2.46
2.45
2.44
2.32
2.31
2.31
2.30
2.28
2.28
2.27
Br
CN
D
Br
2k-*D*
500 MHz, ^{1}H NMR
$CDCl_3$
10
9
8
7
6
5
4
3
2
1
0
ppm
1.98
2.04
0.03
1.00
1.02
1.03
1.02

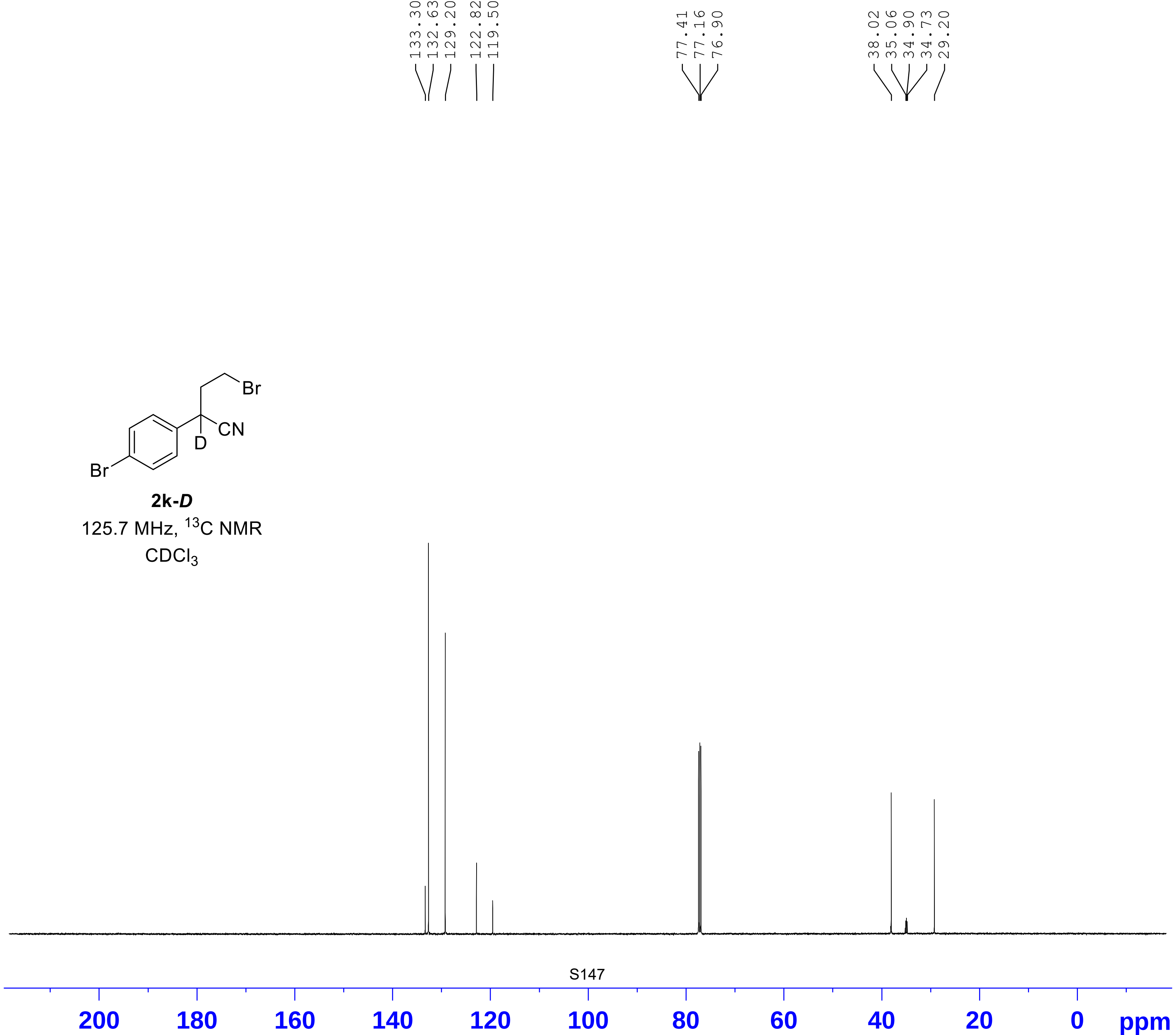

2k-*D*

125.7 MHz, ^{13}C NMR

$CDCl_3$

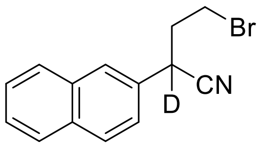

2m-*D*
500 MHz, ^1H NMR
$CDCl_3$

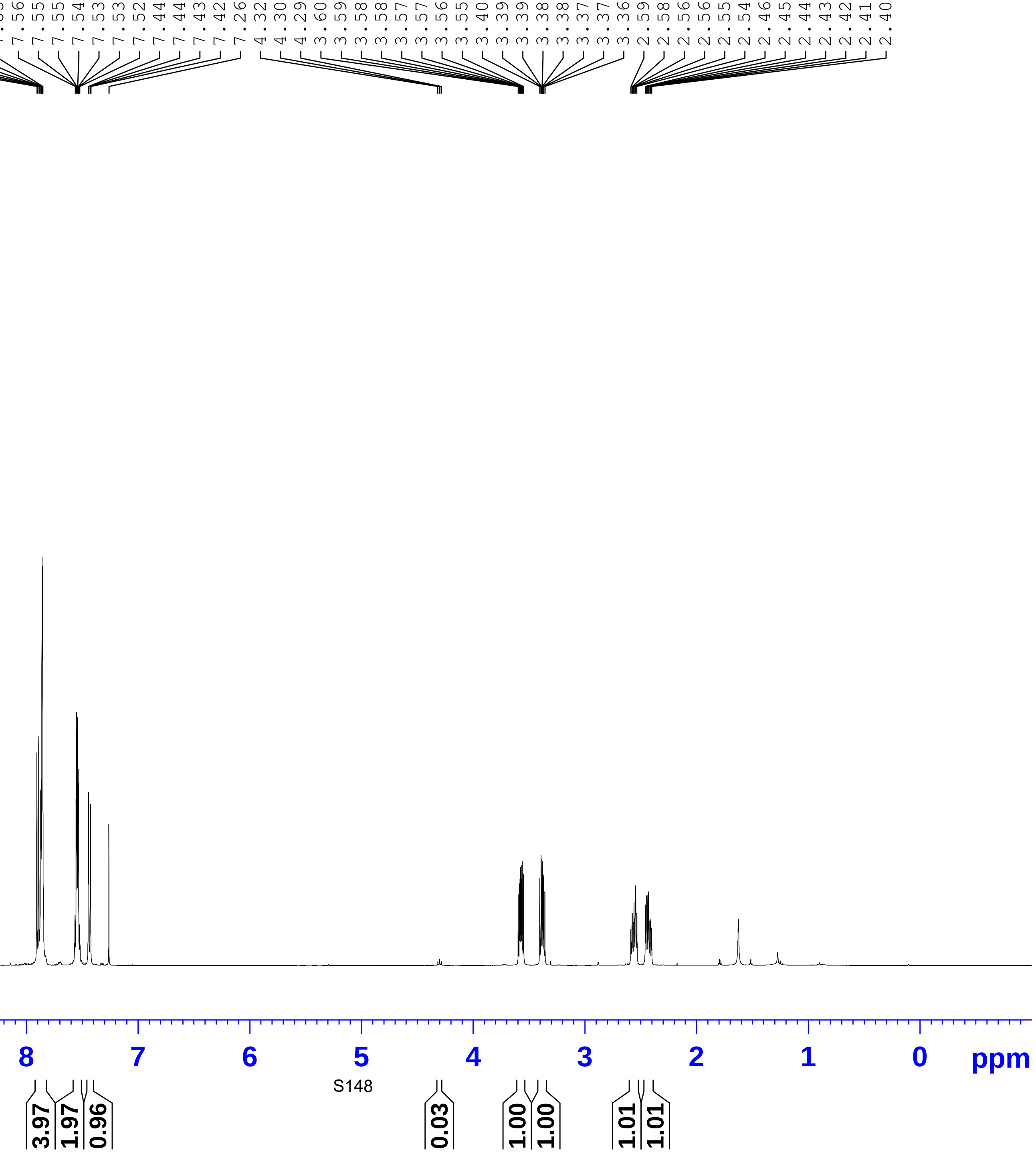

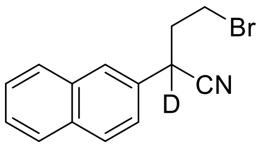

2m-*D*

125.7 MHz, ^{13}C NMR

$CDCl_3$

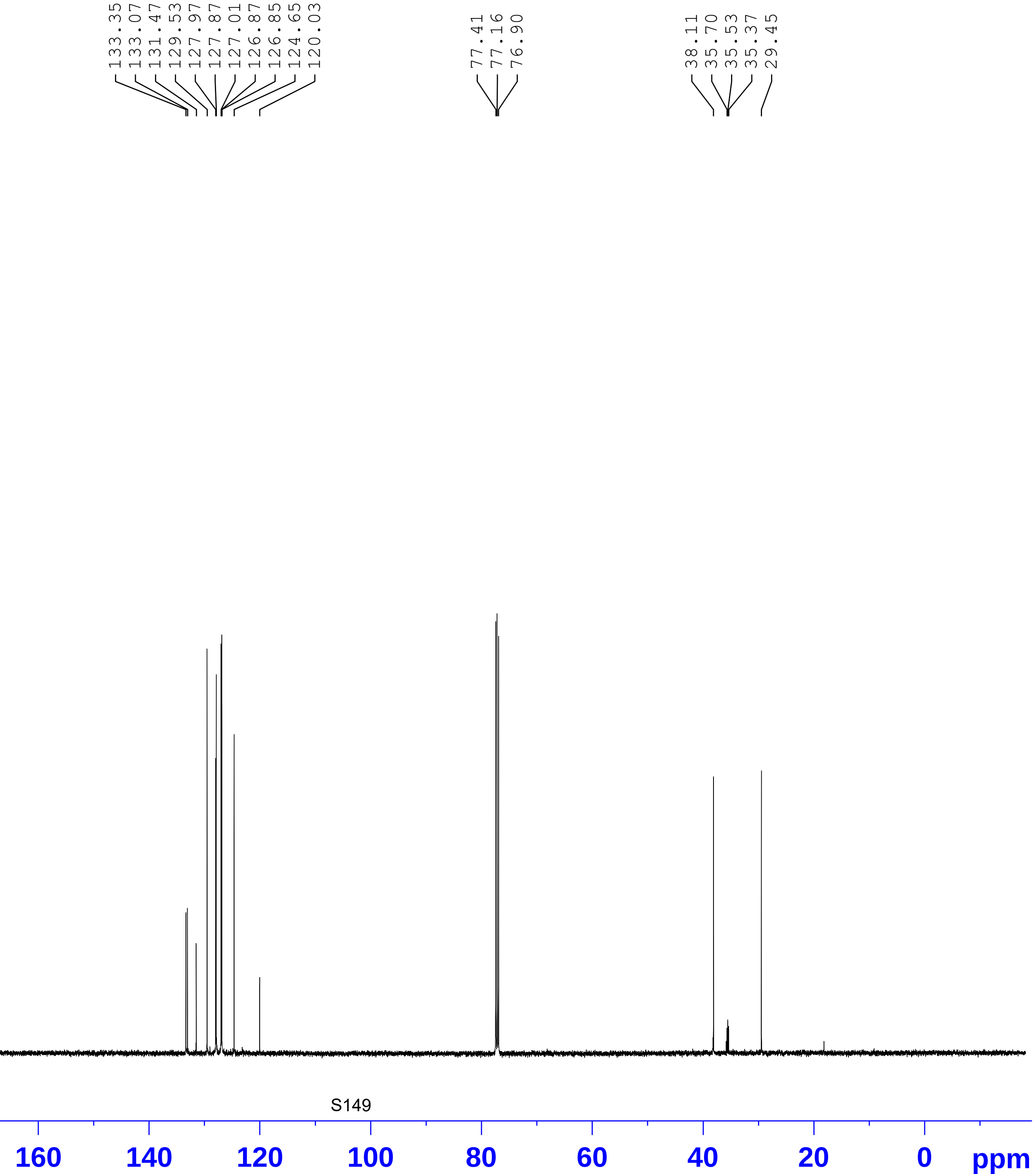

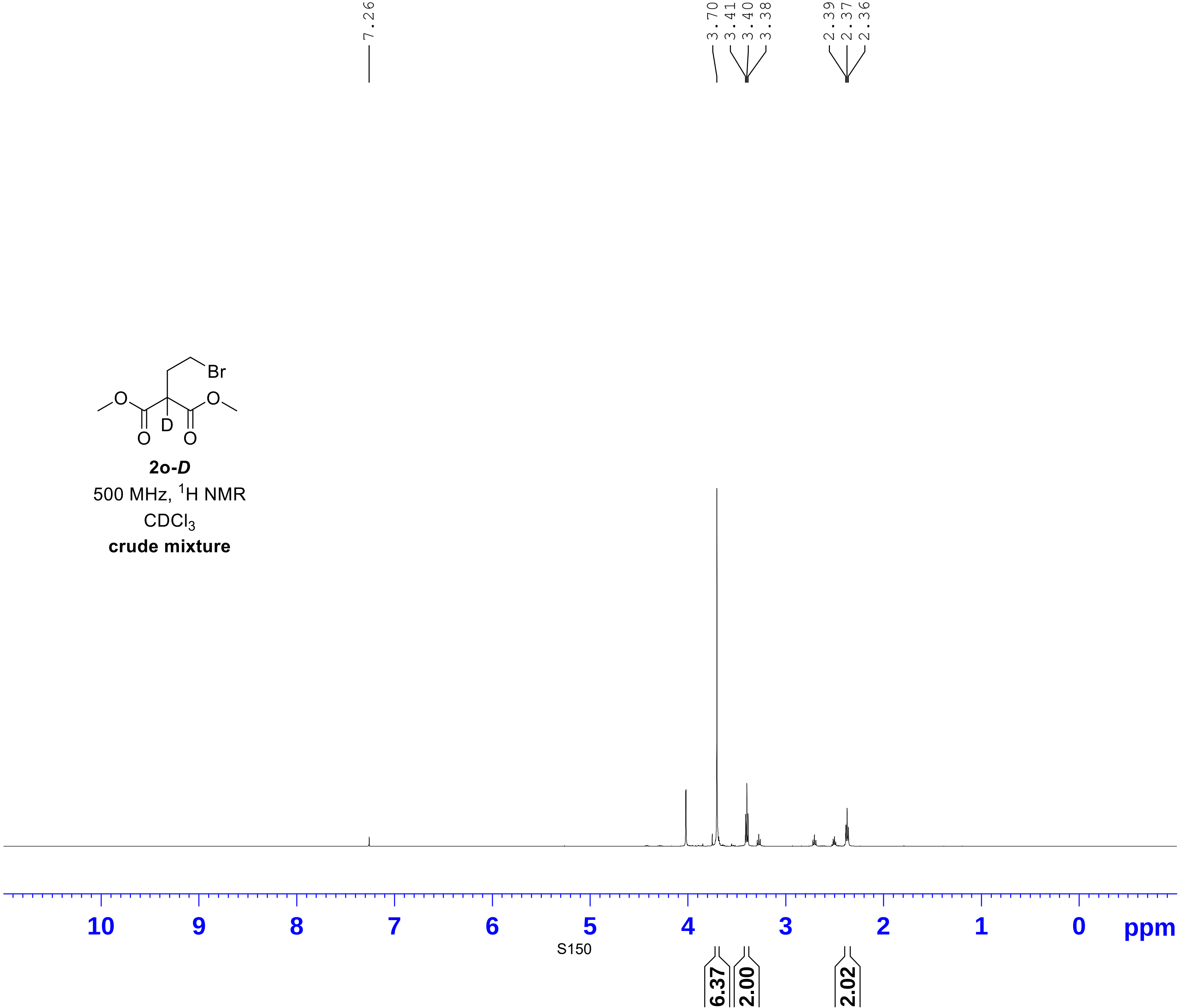
7.26
3.70
3.41
3.40
3.38
2.39
2.37
2.36
Br
O
O
O
O
D
2o-D
500 MHz, 1H NMR
CDCl3
crude mixture
10
9
8
7
6
5
4
3
2
1
0
ppm
6.37
2.00
2.02

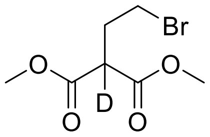

2o-*D*

125.7 MHz, ^{13}C NMR

$CDCl_3$

crude mixture

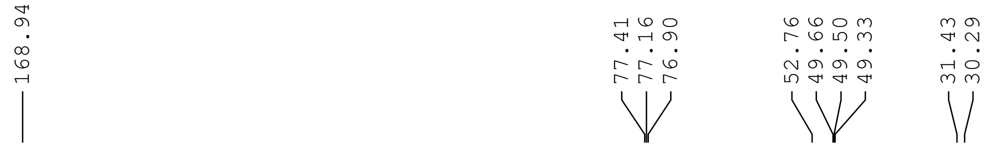

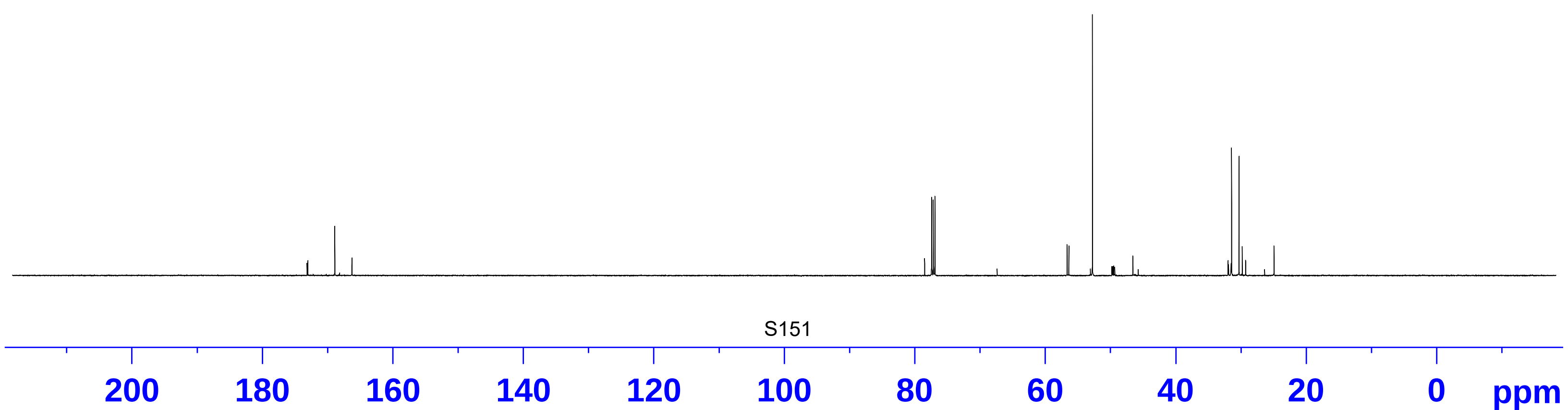

Supplement: SC-011-D0SC02567D-s001 [file SC-011-D0SC02567D-s001.pdf]
